# Diaryl-hemiindigos as visible light, pH, and heat responsive four-state switches and application in photochromic transparent polymers

**DOI:** 10.1038/s41467-023-39944-x

**Published:** 2023-07-20

**Authors:** Maximilian Sacherer, Frank Hampel, Henry Dube

**Affiliations:** Friedrich-Alexander Universität Erlangen-Nürnberg, Department of Chemistry and Pharmacy, Nikolaus-Fiebiger-Str. 10, 91058 Erlangen, Germany

**Keywords:** Photochemistry, Molecular machines and motors

## Abstract

Photoswitches are indispensable tools for responsive chemical nanosystems and are used today in almost all areas of the natural sciences. Hemiindigo (HI) derivatives have recently been introduced as potent photoswitches, but their full applicability has been hampered by the limited possibilities of their functionalization and structural modification. Here we report on a short and easy to diversify synthesis yielding diaryl-HIs bearing one additional aromatic residue at the central double bond. The resulting chromophores offer an advantageous property profile combining red-light responsiveness, high thermal bistability, strong isomer accumulations in both switching directions, strong photochromism, tunable acid responsiveness, and acid gating. With this progress, a broader structural realm becomes accessible for HI photoswitches, which can now be synthetically tailored for advanced future applications, e.g., in research on molecular machines and switches, in studies of photoisomerization mechanisms, or in the generation of smart and addressable materials. To showcase the potential of these distinct light-responsive molecular tools, we demonstrate four-state switching, chemical fueling, and reversible inscription into transparent polymers using green and red light as well as acid/base stimuli, in addition to a comprehensive photochemical study of all compounds.

## Abstract (web summary)

Hemiindigo derivatives can act as photoswitches, but their applicability is limited by synthetic challenges. Here, the authors report the synthesis of modified photoswitches and demonstrate four-state switching, chemical fueling, and reversible inscription into transparent polymers.

## Introduction

**Fig. 1:**
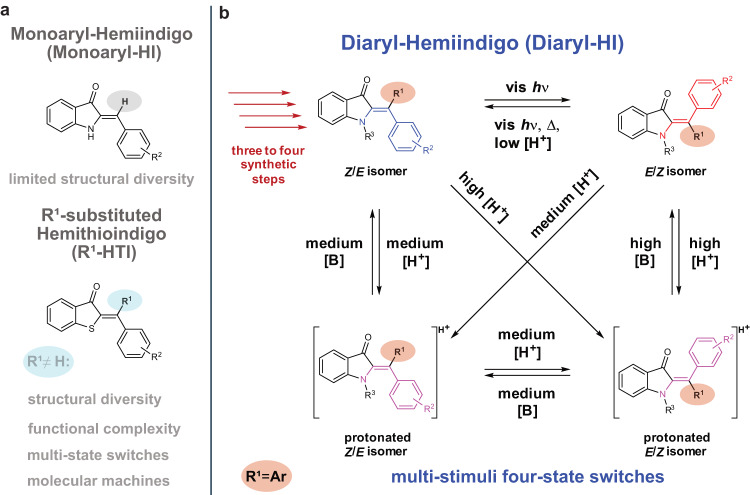
Indigoid chromophores and extended structural realm of diaryl-HIs as multi-stimuli four-state switches. **a** Monoaryl-HIs provide limited structural and functional diversity (proton highlighted in light gray) of previous works. Related higher substituted HTIs enable numerous advanced applications if the proton is replaced by R^1^ (highlighted in light blue). **b** A short and high yielding synthetic access to diaryl-HIs unlocks a plethora of advanced properties (four red arrows, H replaced by aromatic R^1^ highlighted in red), such as green and red-light responsiveness, significant photochromism and color contrasts, high isomer ratios in the photostationary state (pss), high thermal bistability, and applicability of these properties in advanced polymer materials. Aniline-based diaryl-HI chromophores possess two additional protonated switching states. This allows four state switching using the stimuli green light, red light, temperature, acid, and base. This multi-stimuli capability allows to enrich each of the four states individually. Blue-colored structures possess hypsochromic shifted absorption spectra, red-colored structures possess bathochromic shifted absorption, and purple-colored structures are related to protonated species appearing purple to the human eye. Source data are provided in a Source Data file.

Molecular photoswitches have occupied a prominent place in all chemistry-related fields as they allow facile introduction of responsiveness at the nanoscale^1,2^. The field of photoswitch research has developed rapidly especially over the past decade. In its wake the utility of photoswitches has expanded to vastly different areas of applications bringing increasingly complex phenomena under the control of light-signaling in a truly bottom-up fashion. With the aid of photoswitches it has become possible to remote-control bioactivity of drug-like molecules^3–6^, change properties of materials^7–11^, steer catalytic selectivity^12,13^, influence supramolecular assemblies^14–16^, or move molecular machines^17–20^. Such steep progress showcases the potential of this diverse class of molecular enablers. It is inevitably tied to advances at the fundamental level and consequentially unknown molecular photoswitch classes^21^, modes of geometry changes, modes of electronic alterations, and types of photoreactions are being actively researched. Recent examples are modified azobenzene^22–29^, hydrazone-^30^, Stenhouse dye-, imidazolyl-radical-^31^, or indigoid photoswitches^32–37^, each of which offering their own specific advantages. Our group has investigated indigoid chromophores intensively and transformed many of long known dyes in this class into capable photoswitching motives. A distinct advantage is the responsiveness of indigoid core-chromophores to visible light, so that no damaging UV-irradiation is needed for their operation. Additionally, geometries are typically rigid with only limited degrees of freedom facilitating precise control over shapes and spatial relations of functional groups upon switching. Hemithioindigo (HTI) is a prominent representative^38^, consisting of a thioindigo fragment and a stilbene fragment fused together via a photoisomerizable double bond (Fig. 1a). Distinct advances in applicability and photoswitching performance of HTI were made when a fourth substituent could be introduced at the double bond (termed R^1^ in Fig. 1a)^39,40^. With this substitution pattern it became possible to directly evidence a long proposed basic photoreaction, the hula twist^41^, discover a previously unknown photoreaction, the dual single bond rotation (DSBR)^42^, devise fundamentally different types of molecular motors with distinct modes of motion and working mechanisms^43,44^, build advanced multi-state photoswitches^34,42^, or establish the first light-energy powered molecular gearing system^45^.

The related HI structure features an indigo-fragment instead of the thioindigo fragment (Fig. 1a) and shows similar high potential^46^. Monoaryl-HIs bearing strong electron donors at the stilbene fragment represent nearly perfect photoswitches, delivering almost quantitative isomer conversions in both switching directions, red-light responsiveness, high extinction, strong photochromism, sizable quantum yields (QYs), and solvent-independent high thermal bistability^36^. Nitrogen substitution at the indigo fragment allows facile functionalization resulting in distinct applications e.g. as chiroptical switches with unusual electronic circular dichroism (ECD) responses^37^ or ionic chromophores for gas phase photoswitching^47^. Likewise, HI applications in biological context to photomodulate HIV-1 RNA binding^48^ or photoswitching in the presence of bovine serum albumin^49^ have been demonstrated already. For these reasons it can be stated that HIs are highly promising photoswitches, yet their full potential awaits to be explored. Currently different structural modifications of monoaryl-HIs are investigated to improve property design and applicability^50–53^. In light of the advances made with HTIs, diaryl-substitution would be a fundamentally important next step to significantly elevate the capacity of HI photoswitches (Fig. 1b). The resulting structures harbor three (or even four, if the indoxyl benzene ring is also functionalized) different substitution sites, the spatial relations of which are fully controllable during switching. The additional aryl-substitution site of the HI chromophore can then be invoked to tailor favorable electronic and photophysical properties such as photochromism or absorption ever more precisely.

In this work we disseminate the synthetic methodology for generating diaryl-HI chromophores (**1a**-**4c**), a comprehensive photophysical and photochemical analysis of their photoswitching properties, and showcase possible applications for multi-state switching and functional materials. We demonstrate how these structures can be generated in a straightforward, easy to diversify, low-step count, and versatile synthesis. We also evidence them as highly capable photoswitches possessing red-light responsiveness, high thermal bistability, strong isomer accumulations in both switching directions, strong photochromism, tunable acid responsiveness, competence for acid gating, and applicability for photoswitching within functional polymers.

## Results and discussion

### Synthesis

**Fig. 2:**
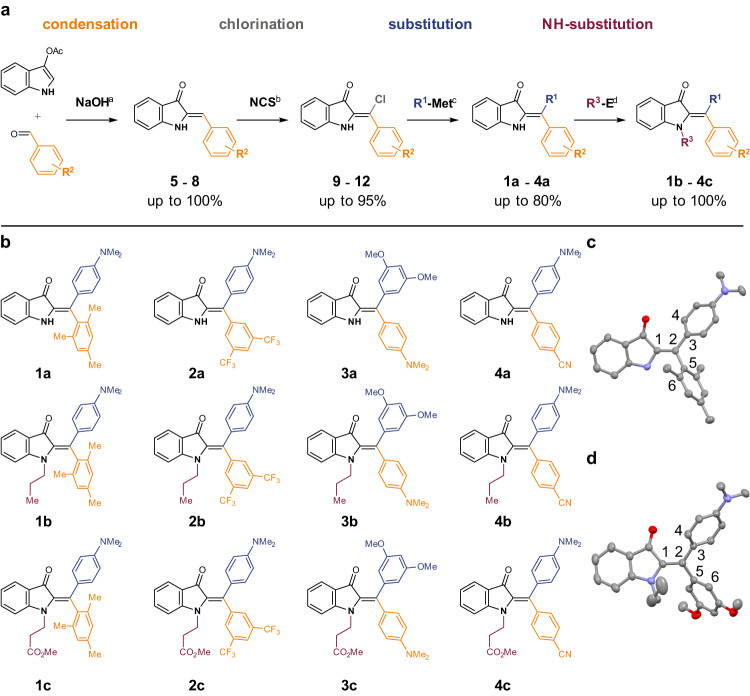
Three to four-step synthesis of highly functionalized diaryl-HI photoswitches 1a-4c. **a** Synthesis route yielding diaryl-HIs via condensation, chlorination, cross-coupling, and NH-substitution. Conditions: ^**a**^ 1,4-Dioxane, 1 h to 13 h, 0 °C or reflux depending on aldehyde. ^**b**^ Methanol (MeOH) : CH_2_Cl_2_ = 20 : 1 or tetrahydrofuran (THF), 2–7 days, 23 °C. ^**c**^ Boronic acid, CsF or CsCO_3_, [Pd^+II^], 1,4-dioxane, 8.5 h up to 48 h, 80 °C. ^**d**^ Variant 1: NaH, 1-iodopropane, *N*,*N*-dimethylformamide (DMF), 30 min up to 2 h 20 min, 23 °C. Variant 2: 1,8-diazabicyclo[5.4.0]undec-7-en (DBU), methyl acrylate, acetonitrile (MeCN), 24 h to 2.5 days, 50 °C. **b** Structures of synthesized diaryl-HI derivatives **1a**-**4c**. R^1^: aryl substituents (blue) incorporated via a Suzuki-Miyaura cross-coupling reaction with corresponding boronic acid. R^2^: aryl substituents (orange) incorporated via condensation. Chlorine (gray) incorporated at the central double bond via NCS. Indoxyl-nitrogen functionalization (purple) via S_N_2 reaction with 1-iodopropane or acrylation with methyl acrylate. **c** Structure of *Z*-**1a** in the crystalline state with a torsion angle of –31.58° between atoms 1, 2, 3, and 4 as well as −71.36° between atoms 1, 2, 5, and 6. Carbon atoms are colored in gray, oxygen atoms in red, nitrogen atoms in blue. **d** Structure of *Z*-**3b** in the crystalline state with a torsion angle of +38.00° between atoms 1, 2, 3, and 4 as well as +54.04° between atoms 1, 2, 5, and 6. Carbon atoms are colored in gray, oxygen atoms in red, nitrogen atoms in blue. Source data are provided in a Source Data file.

To develop a synthesis for diaryl-HIs, important prerequisites need to be taken into consideration, as the synthesis of diaryl-HTIs could not be adapted to access diaryl-HIs (Dieckmann-type condensations of different anthranilic acids/acid chlorides/esters followed by chlorination with oxalyl chloride were not successful and also condensations of indoxyl derivatives with ketones did not deliver the desired products). First, the parent HIs with one residual H-atom at the central photoisomerizable double bond are easily accessible in high yields by condensation of commercially available aldehydes (introducing R^2^) and indoxyl acetate (Fig. 2). Therefore, the parent HIs would be ideal starting materials for introducing a fourth substituent at the double bond (R^1^) as well as nitrogen substituents replacing the NH proton (R^3^) later. Second, introduction of a fourth substituent should be versatile and thus a common precursor allowing easy diversification by well-established broad-scope methodology was deemed preferable. Third, facile alkylation at the indoxyl nitrogen would allow to introduce functionalities for late-stage covalent connection of the HI photoswitches. For these reasons, chlorination of the double bond was chosen to enable subsequent chlorine-substitution reactions with a nucleophile (attempts for Heck reactions were equally unsuccessful as attempts of directly generating metal organic species by C–H activation at position R^1^). The envisioned chlorination was inspired by the described substitution at the Michael system double bond (R^1^) with a nucleophilic reactant such as KCN and subsequent oxidation^54^ (note: attempts at subsequent cross coupling reactions of HIs with nitriles at position R^1^ were not successful). However, employing related reactants such as KCl or KBr instead of KCN did not lead to halogenated product formation. Changing the electronic character of the reactant from a nucleophilic halide to an electrophilic halonium ion was then attempted for substituting at the desired position R^1^. However, this electrophilic reaction faces selectivity problems as either the *para* or *ortho* position to the nitrogen at the indoxyl benzene are nucleophilic. It was thus found rather expectedly that halogenation with *N*-bromosuccinimide (NBS) leads to dominant substitution of the electrophilic *para* position at the indoxyl benzene ring, similar to the literature known bromination of isatin^55^. Attempts at chemoselective bromination reactions at the *β*-position of the Michael system^56^, as well as bromination with elemental bromine also did not lead to successful functionalization at position R^1^. With *N*-chlorosuccinimide (NCS) the R^1^ position was chemoselectively accessible without side reactions at the indoxyl benzene. Because of the inherent Michael system within the HI structure, exchange of the chloride could then be accomplished by a direct nucleophilic substitution, using cross coupling chemistry, or even radical chemistry if needed. A final introduction of an ester function at the indoxyl nitrogen atom (R^3^) was deemed suitable as a versatile handle for facile implementation of HI photoswitches, e.g., in peptides, natural products, or polymers. These considerations led to the modular synthesis shown in Fig. 2 (details about methods, synthesis, and characterization are outlined in Supplementary Notes 1–3, Supplementary Table 1, and Supplementary Figs. 1–34).

The particular R^x^-groups at the diaryl-moiety were chosen first and foremost to facilitate the photoswitching properties. For proper photochromism and switchability one electron-rich aromatic residue is needed, similar to monoaryl-HIs^36,37,47,51^, HTIs^57–61^, and the recently reported diaryl-HTIs^40,62^. Thus a *p-*aniline (*p*-NMe_2_) residue was combined with a second neutral or electron-poor residue at the diaryl-moiety, which needs to be different in its electronic nature in order to distinguish *E* and *Z* isomers from each other.

As described previously, the condensation reaction between indoxyl acetate and different aldehydes leads to very high yields of the resulting parent monoaryl-HIs **5** to **8**. Chlorination of parent HIs also proceeded in good to very good yields when using NCS typically at 23 °C in tetrahydrofuran (THF), with the notable exception of the electron rich HI **7** (R^2^ = *p*-NMe_2_). In this case substantial chlorination at the α position to the *p*-NMe_2_ is observed as side reaction. In general, aprotic solvents reduce side reactions at the indoxyl benzene, and high concentrations of the HI further improves the yield. The subsequent Suzuki-Miyaura cross coupling reactions with aromatic boronic acids delivered the corresponding diaryl-HIs in moderate to high yields. A final *N*-alkylation of the indoxyl nitrogen (R^3^) using either 1-iodopropane or methyl acrylate delivered the highly functionalized HI photoswitches **1b**-**4c**. Crystals suitable for X-ray analysis were obtained for HIs **1a** and **3b** evidencing the twisted nature of the diaryl moieties as well as the constitution of the photoswitches (Fig. 2c, d, Supplementary Note 10, and Supplementary Table 12).

### Thermal behavior

To establish the switching properties of diaryl-HIs **1a**-**4c**, their thermal behavior in the dark was scrutinized first (Table 1, Supplementary Note 7, Supplementary Table 5, Supplementary Figs. 93–104). In most cases, slow isomerization takes place at elevated temperatures to establish an equilibrium with both *Z* and *E* isomers being present in significant amounts. This behavior also allows to directly measure the relative free enthalpy differences Δ*G* between isomers as summarized in Table 1. When analyzing the kinetics of thermal *E*/*Z* or *Z*/*E* isomerizations in the dark, the corresponding Gibbs energies of activation Δ*G*^‡^ could be obtained. All HIs exhibit high thermal bistability as manifested in sizable Δ*G*^‡^ values, which are >25 kcal mol^-1^ giving rise to corresponding half-live times of isomers ranging from hours to >100 years at 25 °C (linearly extrapolated from elevated temperature measurements). The only exceptions are HIs **2b** and **3c**, which are thermally more labile with corresponding half-lives in the minute to hour range, respectively. It is further observed that increasing steric hindrance at the non-*p*-NMe_2_ aryl residue of the diaryl-moiety increases the thermodynamic stability of the bathochromic isomer.

**Table 1:** Quantitative comparison of the physical and photophysical properties of HIs 1-4

diary-HI	Δ*G*^‡^_E→Z_/ Δ*G*^‡^_Z→E_ (kcal mol^−1^)	Δ*G* (kcal mol^−1^)	*E*:*Z* at thermal equilibrium (temperature)	equil. *t*_1/2_ at 25 °C^d^	isomer % in pss (LED wavelength)	*λ*_max_ (nm) and *ε*_max_ (L mol^−1^ cm^−1^) of *E*/*Z* isomers lowest energy absorption band	Δ*λ*_max_ (nm) and Δ*ε*_max_ (L mol^−1^ cm^−1^) of *E*/*Z* isomers
**1a**	25.0^a^/25.3^a^	0.3^a^	39:61^a^ (60 °C)	1.5 days^a^	47% *E* (590 nm)^a^ 94% *Z* (450 nm)^a^	477^c^; 15,300^c^/ 518^c^; 12,200^c^	41^c^; 3100^c^
**1b**	31.2^b^/31.4^b^	0.3^b^	42:58^b^ (135 °C)	118 years^b^	62% *E* (625 nm)^a^ 100% *Z* (470 nm)^a^	499^c^; 7700^c^/ 539^c^; 7700^c^	40^c^; 0^c^
**1c**	31.4^b^/31.9^b^	0.5^b^	34:66^b^ (135 °C)	199 years^b^	63% *E* (625 nm)^a^ 100% *Z* (470 nm)^a^	493^c^; 9600^c^/ 534^c^; 7600^c^	41^c^; 2000^c^
**2a**	-^e^	-^e^	- ^e^	-^e^	-^e^	-^e^	-^e^
**2b**	22.9^a^/22.5^a^	0.4^a^	67:33^a^ (25 °C)	36 min^a^	79% *E* (625 nm)^a^ 92% *Z* (490 nm)^a^	518^c^; 9600^c^/ 563^c^; 8300^c^	45^c^; 1300^c^
**2c**	29.2^a^ /27.9^a^	1.3^a^	87:13^a^ (80 °C)	291 days^a^	92% *E* (660 nm)^a^ 91% *E* (625 nm)^g^ 92% *Z* (470 nm)^a^ 81% Z (470 nm)^g^	507^c^; 6000^c^/ 562^c^; 5100^c^	55^c^; 900^c^
**3a**	-^b, f^	0.1^b, f^	47:53^b^ (0 °C)	-^b, f^	50% *E* (530 nm)^a^ 53% *Z* (450 nm)^a^	477^c^; 5900^c^/ 523^c^; 4700^c^	46^c^; 1200^c^
**3b**	27.4^a^/27.5^a^	0.1^a^	46:55^a^ (80 °C)	74 days^a^	62% *E* (590 nm)^a^ 92% *Z* (470 nm)^a^	512^c^; 9700^c^/ 542^c^; 9000^c^	30^c^; 700^c^
**3c**	24.5^a^/24.6^a^	0.1^a^	48:52^a^ (60 °C)	14 h^a^	46% *E* (550 nm)^a^ 72% *Z* (470 nm)^a^	503^c^; 8800^c^/ 541^c^;7300^c^	38^c^; 1500^c^
**4a**	-^a, e^	-^a, e^	-^a, e^	-^a, e^	-^a, e^	-^c, e^	-^c, e^
**4b**	26.5^a^/27.4^a^	0.8^a^	23:77^a^ (80 °C)	27 days^a^	89% *E* (505 nm)^a^ 81% *E* (505 nm)^g^ 87% *Z* (660 nm)^a^ 88% *Z* (625 nm)^g^	561^c^; 6500^c^/ 517^c^; 7100^c^	44^c^; 600^c^
**4c**	26.8^a^/27.5^a^	0.7^a^	26:74^a^ (80 °C)	40 days^a^	90% *E* (490 nm)^a^ 90% *E* (490 nm)^g^ 93% *Z* (660 nm)^a^ 88% *Z* (625 nm)^g^	560^c^; 3600^c^/ 507^c^; 4100^c^	53^c^; 500^c^

^a^ in toluene-*d*_8._

^b^in *o*-xylene-*d*_10._.

^c^in toluene/toluene-*d*_8_.

^d^Equilibration half-life time of thermal isomerization, linearly extrapolated to 25 °C.

^e^Values not determined due to photodegradation.

^f^No change during heating.

^g^in THF-*d*_8_.

### Photoswitching properties

**Fig. 3:**
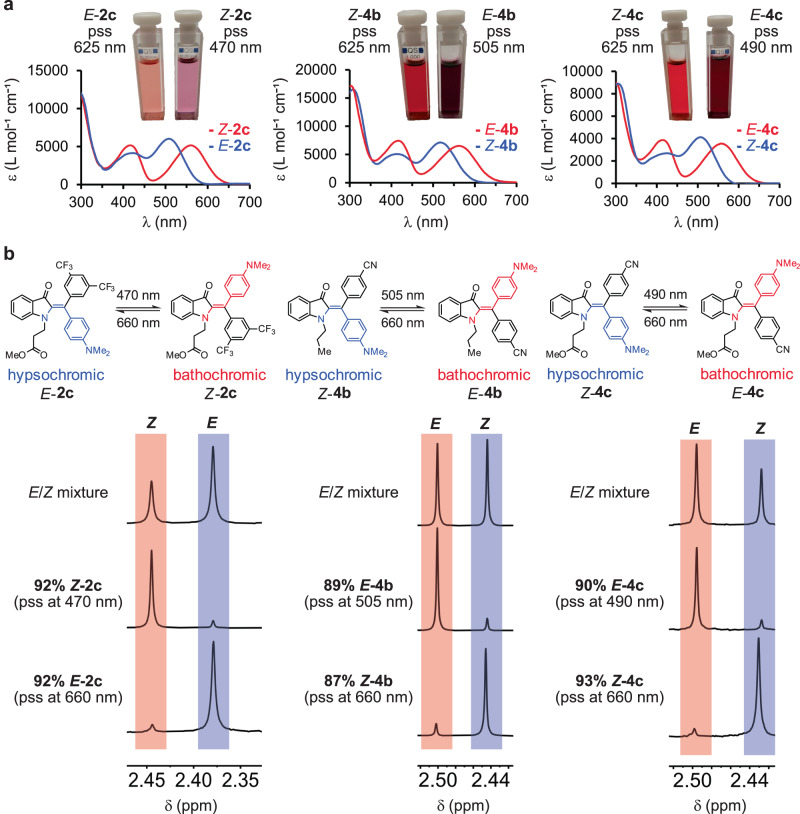
Photoswitching properties of diaryl-HIs. Hypsochromic *E*/*Z* species are highlighted in blue und bathochromic *Z*/*E* species are highlighted in red. **a** Molar absorption coefficients of pure *E* and *Z* isomers of **2c,** **4b**, and **4c** in toluene solution and corresponding photographs of solutions after irradiation with light of suitable wavelengths to the respective pss. **b** Indicative sections of ^1^H NMR spectra (400 MHz, 23 °C, toluene-*d*_8_) of **2c,** **4b**, and **4c** recorded after irradiation with light of suitable wavelengths to the respective pss. Source data are provided in a Source Data file.

With high bistability being established, the photophysical and photochromic properties of HIs **1**-**4** were scrutinized next (see Table 1 and Fig. 3). All compounds show well-separated UV/Vis absorption spectra for their *E* and *Z* isomers (Fig. 3, Supplementary Note 4, Supplementary Table 2, and Supplementary Figs. 35–44). In general, isomers in which the carbonyl group of the indigo fragment and the *p*-NMe_2_ substituent are residing at the same side of the double bond possess bathochromically shifted absorptions. They are referred to as bathochromic species in the following, and the corresponding other isomer as hypsochromic species (see “Methods: Synthesis” section). This behavior gives rise to pronounced photochromism with maxima differences reaching 40 nm (Supplementary Note 5: Supplementary Table 3, and Supplementary Figs. 46, 48–50, 52, 53, 55, 59–63, 65, 67, 68, 72–74, and 78–80). Associated with this very good absorption separation is a significant color contrast between the two isomers in solution as shown in Fig. 3 (see Supplementary Note 5 for the corresponding photographs of all derivatives in Supplementary Figs. 46, 48, 52, 55, 59, 63, 65, 67, 72, and 78). Compared to structurally related monoaryl-HIs^36^ or diaryl-HTIs^40,62^, the here presented diaryl-HIs show strongly bathochromic shifted UV/Vis absorbance bands (up to 30 nm shifted compared to the most bathochromic shifted structurally similar derivatives available so far). Also in comparison to pyrrole heterocycles fused to HTIs^63^ or electron rich monoaryl-HTIs^64^, the here presented systems exhibit more bathochromically shifted UV/Vis absorbance bands and similar very good band separation of the corresponding isomers. The comparison of UV/Vis spectra measured in solvents of different polarity show, that the absorbance maxima of the hypsochromic isomers undergo a further bathochromic shift in polar and highly polar solvents such as THF, methanol (MeOH), dimethylsulfoxide (DMSO) or DMSO/H_2_O, whereas the bathochromic species exhibits hypsochromic or bathochromic shifts (Supplementary Note 5, Supplementary Table 4, and Supplementary Figs. 49, 50, 58, 60-62, 71, 73, 74, 77–80). The molar absorption coefficients of diaryl-HIs are in the range of 4000–15,300 L mol^−1^ cm^−1^ and thus are perfectly suited for photoswitch applications (Table 1). The noticeable decrease in molar absorption coefficients in comparison to monoaryl-HIs^36^ is owed to significantly twisted aromatic substituents as a result of their mutual steric hindrance (Figs. 2 and 3, Supplementary Note 4, Supplementary Table 2, and Supplementary Figs. 35–44, corresponding crystal structures are described in Supplementary Note 10). A similar influence of molecular twisting on the molar absorption coefficients has been described, i.e., for HTI photoswitches before^60,63^. Photoisomerization reactions of the highly substituted HIs were quantified by UV/Vis and ^1^H NMR spectroscopy to cover a range of concentrations and test the robustness of photoswitching (Supplementary Note 5, Supplementary Tables 3 and 4). Photoisomerization reactions that led to enrichment of the bathochromic species resulted in nearly quantitative isomer enrichment in all cases except for HIs **3a** and **3c** for which 53% and 72% of the bathochromic species are obtained, respectively. Irradiation with light of longer wavelengths resulted in enrichment of the hypsochromic species with more variation in individual isomer yields (46% - 93%). The individual NMR experiments are shown in Supplementary Figs. 45, 47, 51, 54, 56-58, 64, 66, 69–71, and 75–77). Increasing polarity of the solvent does not hamper photoswitching abilities but slowed down photoconversion is observed in DMSO solution (Supplementary Figs. 50 and 62). Thermal stability is reduced in protic solvents for most diaryl-HIs except for **1b** and **1c** (Supplementary Figs. 49, 50, 61, 74, and 80). QYs were found to be below 1% for photoisomerization of bathochromic species to hypsochromic species of diaryl-HIs. The QYs for photoisomerization of hypsochromic species to bathochromic species were determined to be between 4% to 6% (**2b,** **2c,** **4b**, and **4c**). Individual data are presented in Supplementary Note 6, Supplementary Table 5, and Supplementary Figs. 81–92).

Taken together diaryl-HIs **2c,** **4b**, and **4c** possess the ideal combination of high thermal bistability, bathochromically shifted absorptions, strong photochromism, strong enrichment of each isomer in the respective pss, red and blue light responsiveness, highest QYs in the series, and high level of functionalization. However, also derivatives **1b,** **1c**, and **3b** deliver with quantitative isomer conversion at blue light irradiation and beyond 60% conversion with yellow and red-light irradiation. Derivatives **1b** and **1c** are interesting in their own right as they are highly bistable with half-lives in the dark beyond 100 years at 25 °C (linearly extrapolated from high temperature measurements) and are significantly resistant to acid-induced isomerization. They also appear thermally highly bistable in protic solvents such as MeOH and DMSO/H_2_O mixtures while retaining their photoswitching capacity. Looking at the photophysical and photochemical properties of diaryl-HIs **1**-**4**, the following tendencies can be observed. The stronger the donor/acceptor system at the stilbene moiety is, the more bathochromically shifted the UV/Vis absorption bands, the better the photochromism and the better the isomer enrichment in both switching directions are. The substitution of the proton at position R^3^ improves the photochemical behavior in terms of isomer content in both switching directions in the pss and photochemical stability during irradiation. There is also a tendency, that thermal bistability increases if position R^3^ is functionalized with alkyl substituents. Substituents at position R^3^ possessing a stronger *–I* effect exhibit slightly better thermal bistabilities than those possessing mostly a *+I* effect. This is interesting as the thermal bistability Δ*G*^‡^_Z→E_ of HI **1** can be improved from 25.3 kcal mol^-1^ (R^3^ = H) to 31.9 kcal mol^-1^ (R^3^ = (CH_2_)_2_C(O)OCH_3_) by making use of inductive effects. Taken the results together, it is evident that a push-pull system cross-conjugated at the central double bond in combination with the substitution of the proton at the indoxyl nitrogen are most effective for high isomer enrichment in both photoswitching directions. However, also a combination of strong and weak electron donated aromatic substituents results in proper photoswitching behavior.

### Application for photochromic polymers

**Fig. 4:**
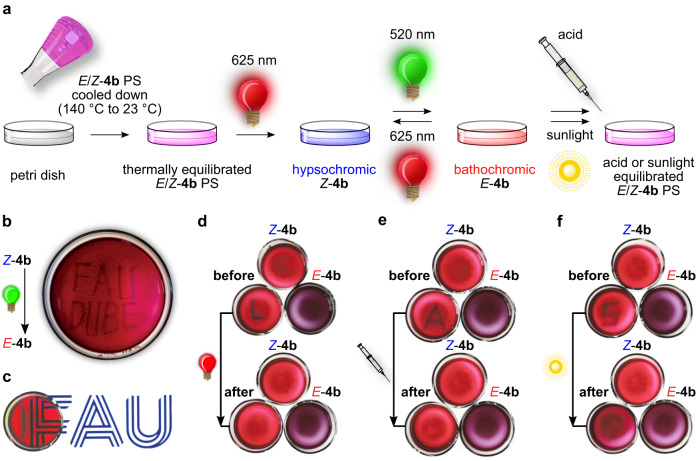
Incooperation of diaryl-HI 4b into a PS polymer and resulting photochromic behavior of the transparent material. **a** Schematic illustration of diaryl-HI functionalized polymer preparation (thermal polymerization, magenta colored flask), photowriting (520 nm light, green lamp symbol), and application of different erasing methods (625 nm Light: red lamp symbol, Acid: acid syringe symbol, Sunlight: sun symbol) within the PS matrix. Petri dishes containing thermally, sunlight or acid equilibrated *E*/*Z*-**4b** mixtures are displayed in magenta, hypsochromic *Z*-**4b** isomer in blue, and bathochromic *E*-**4b** isomer in red. **b** Local enrichment of the bathochromic *E*-**4b** isomer by using a glass-fiber guided beam of 520 nm light to write “FAU DUBE” into a prior hypsochromic *Z*-**4b** enriched PS matrix. The depicted petri dish has a diameter of 90 mm. **c** Transparency showcase of the photochromic diaryl-HI PS with one light-inscribed line. The petri dish containing the polymer was placed on a monitor displaying the FAU logo. **d** Erasing a previously written letter “L” with 625 nm Light within 5 min. **e** Erasing a previously written letter “A” by Acid addition (trifluoroacetic acid (TFA), 1 equiv., 6.5 mm in toluene) within 10 min. **f** Erasing a previously written letter “S” with Sunlight within 10 hours. Petri dishes **d**–**f** with a diameter of 25 mm were placed on a monitor displaying a white screen and are composed of PS and different isomer compositions of diaryl-HI **4b**. Reference samples photochemically enriched with either bathochromic *E*-**4b** (505 nm irradiation, purple color) or hypsochromic *Z*-**4b** (625 nm irradiation, red color) are shown for comparison.

The pronounced color contrasts between the *E* and *Z* isomers of diaryl-HIs, their high thermal isomerization barriers and moderate QYs are advantageous for applications in functional materials, which are stable under ambient conditions and even under exposure to indirect sunlight. To showcase the versatility of diaryl-HIs and translation of their green/red light responsiveness to the solid phase we have implemented derivatives **1b** and **4b** into polystyrene (PS) (for polymer application of **4b** see Fig. 4 and for **1b** and **4b** see Supplementary Note 9, and Supplementary Figs. 146 and 147). During thermal polymerization of a mixture of PS and diaryl-HIs, the latter are stable and retain their photoswitching properties (Fig. 4a). The resulting polymer is highly transparent and displays reversible photochromic properties changing from red to deep purple color upon irradiation with red or green light, respectively (Fig. 4b–d). Because of the significant photochromism of diaryl-HIs and their multi-stimuli response (see acid-base induced switching below), it is possible to draw or write with light persistently into the PS polymer (Fig. 4b and c), erase the written inscription with light of another wavelength (Fig. 4d), and also erase by adding acid to the polymer (Fig. 4e). The written areas remain stable within the polymer until several hours under exposure to indirect sunlight (Fig. 4f) and at least for several days to weeks in the dark at 23 °C.

### Acid-base induced switching

**Fig. 5:**
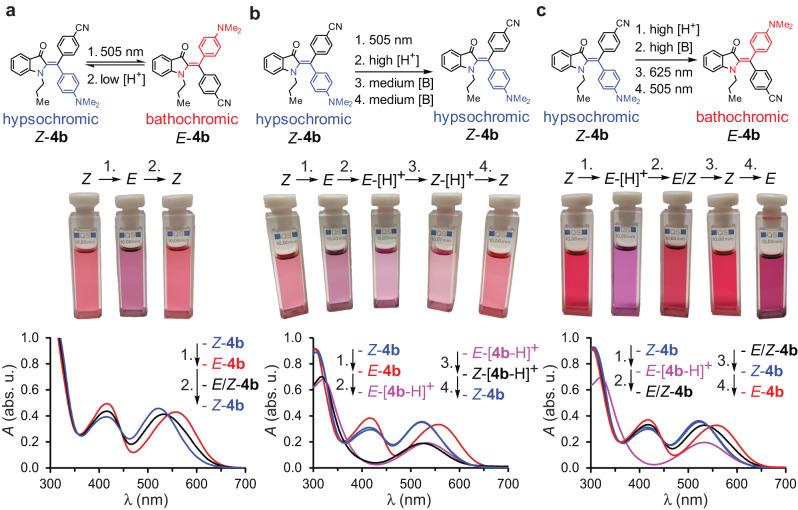
Different Acid and light induced switching properties of diaryl-HI **4b** in toluene at 23 °C, visualized by change of UV/Vis absorption spectra and corresponding solution color changes (photographs of cuvettes). Species are colored for distinction, *Z*-**4b** in blue, *E*-**4b** in red, *E*/*Z*-**4b** mixtures and *Z*-[**4b**-H]^+^ in black, *E*-[**4b**-H]^+^ in purple. The numbers 1.-4. refer to different given stimuli. **a** Dual switching mode for **4b** using acid and light as stimuli. Addition of small amounts of TFA (here: 0.25 equiv.) to *E*-**4b** leads to thermal isomerization to *Z*-**4b** within 25 min in the dark. **b** Adding excess of TFA (4000 equiv., high [H^+^]) to *E*-**4b** leads to protonated *E*-[**4b**-H]^+^ and pronounced absorption changes. Stepwise addition of NEt_3_ ([B]) leads first to formation of protonated *Z*-[**4b**-H]^**+**^ (signified by hypsochromic absorption shift) and subsequently to regeneration of *Z*-**4b**. **c** Treatment of *Z*-**4b** with high concentration of TFA (4000 equiv.) induces isomerization to *E*-[**4b**-H]^+^ and abolishes photoswitching. Subsequent addition of NEt_3_ fully recovers the photoswitching capacity. Source data are provided as a Source Data file.

**Fig. 6:**
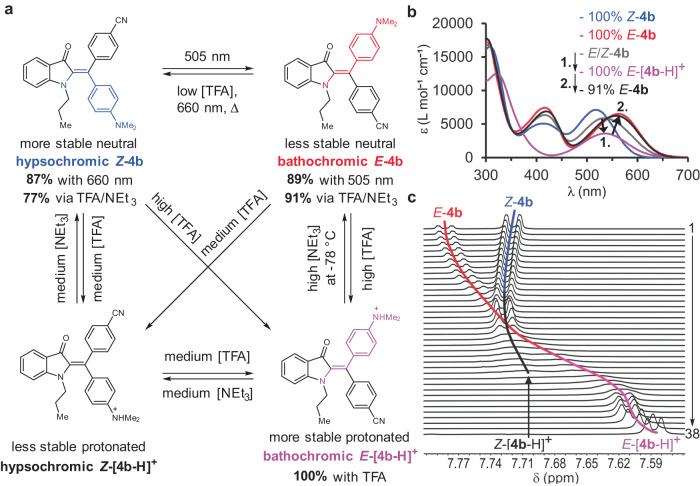
Addressability of the four states of diaryl-HI 4b. **a** Overview of the multi-stimuli addressability (visual light, temperature, acid, and base concentration) of the four states of diaryl-HI **4b**. Species are colored for distinction, *Z*-**4b** in blue, *E*-**4b** in red, *Z*-[**4b**-H]^+^ in black, *E*-[**4b**-H]^+^ in purple. **b** Enrichment of the thermodynamically less stable neutral *E*-**4b** species (black) via TFA addition and neutralization with NEt_3_ at –78 °C. Addition of large excess of TFA to a *E/Z*-**4b** mixture (gray) leads to complete conversion to *E*-[**4b**-H]^+^ (purple). Subsequent addition of an equal excess of NEt_3_ at –78 °C leads to the enrichment of 91% of thermodynamically less stable neutral *E*-**4b** (black). The molar absorption coefficients of pure *Z*-**4b** (blue) and *E*-**4b** (red) species are depicted as references. **c** ^1^H NMR titration experiment adding excess of TFA-*d*_1_ (0–647 equiv., spectrum 1–38) to a thermally equilibrated, *Z-***4b** enriched mixture (spectrum 1–38, 601 MHz, 1.12 mm in toluene-*d*_8_, 25 °C). The stepwise complete population of the *E*-isomer and corresponding depopulation of the *Z*-isomer is shown. Source data are provided in a Source Data file.

Besides photoisomerization it is also possible to isomerize the neutral metastable species of diaryl-HIs to the most stable ones by adding acid (Figs. 5a and 6a, Supplementary Note 8, Supplementary Figs. 105, 106, 113, 116, 118, 119, 122, 124–126, and 144, and Supplementary Table 7). Acid-induced isomerization is a function of acid concentration and the intrinsic electronic properties of the respective diarly-HI. The more pronounced the cross conjugation at the diaryl-fragment, i.e., by donor-acceptor (R^1^/R^2^) substitutions, the faster is the acid-induced isomerization. Fast acid-induced isomerization is also seen in cases where R^3^ = H. However, in the absence of a donor-acceptor system at the diaryl-fragment, acid-induced isomerization proceeds much slower and significant amounts of acid are needed to accelerate it (e.g., in the case of **1b** and **1c**, Supplementary Figs. 107, 110, and 112, and Supplementary Table 7). Therefore, a pH sensitive switching can be established for diaryl-HIs where acid addition serves as another stimulus that leads to facile thermal isomerization (Fig. 5a). Further, addition of excess of acid enables access of a third and also a fourth state of diaryl-HIs (Figs. 5b and 6, Supplementary Figs. 107, 108, 111, 114, 115, 120, and 121). The third state represents the protonated form of the corresponding thermodynamically more stable neutral isomer, and the fourth state represents the protonated form of the thermodynamically less stable neutral isomer (inversion of thermodynamic stabilities can be observed between neutral and protonated forms as described below).

To illustrate the multi-switching properties, derivative **4b** is discussed in more detail in the following. After adding small amounts of TFA (up to 4.0 equiv.) to a toluene solution of enriched bathochromic *E*-**4b**, the UV/Vis spectrum does not change significantly at first. However, over the course of 25 min at 23 °C the absorption spectrum transforms into the known absorption spectrum of the thermodynamically more stable hypsochromic *Z*-**4b** isomer (Fig. 5a, Supplementary Figs. 124–126, and Supplementary Table 7). In the presence of such small amount of TFA a different reversible switching mode can thus be established, in which 505 nm light leads to photoisomerization from *Z*-**4b** to *E*-**4b** and the reverse isomerization proceeds thermally activated in the dark (Fig. 5a). Multiple switching cycles in the presence of 0.25 equiv. TFA and 505 nm light are possible (Supplementary Figs. 124 and 125). However, reversible photoswitching is still possible using 625 nm light for photoisomerization from *E*-**4b** to *Z-***4b** (Supplementary Fig. 127). When adding a base like Et_3_N, high thermal stability is recovered changing the switching mode to sole reversible photoisomerizations using 505 nm and 625 nm light (Fig. 5c and Supplementary Figs. 131 and 132). The acid-induced and accelerated isomerization to hypsochromic *Z*-**4b** has also been employed in the polymer-application of this diaryl-HI to effectively erase inscribed information from the sample (Fig. 4e).

Upon addition of a large excess of TFA (up to 5000 equiv.) to bathochromic *E*-**4b** or hypsochromic *Z*-**4b** distinct changes in the absorption are observed. Most important is the missing band centered at around 420 nm, which gives the solution a distinct and lighter purple color to the human eye (Figs. 5b, c and 6c). Two distinct new absorption spectra can be obtained, which are ascribed to two new states identified as the protonated forms of the *E*- and *Z*-isomers, respectively. The third state is assigned to the *Z*-[**4b**-H]^**+**^ isomer with a hypsochromically shifted absorption and the fourth state to the *E*-[**4b**-H]^**+**^ isomer with a bathochromically shifted absorption (Figs. 5b and 6). The assignments were made because of the following observations. Mass spectroscopy experiments revealed that no twofold protonated diaryl-HI species were formed even after adding large excess of TFA (Supplementary Tables 8-11). NMR analysis revealed, that in pure TFA-*d*_1_ solution only the *E*-[**4b**-H]^+^ species is present (Supplementary Figs. 140–143). An ^1^H NMR titration experiment in toluene-*d*_8_ solution shows that increasing amounts of added TFA-*d*_1_ convert the initially 77% of *Z*-**4b** (thermodynamically most stable isomer in the neutral form) completely to the protonated *E*-[**4b**-H]^**+**^ species (after 43 equiv. TFA-*d*_1_ added to a 1.12 mm solution of **4b**, Fig. 6c and Supplementary Figs. 134–139). When first adding a large excess of TFA (ca. 5000 equiv. at UV/Vis concentrations) to a neutral *Z*-**4b** enriched toluene solution and subsequently adding an equally large excess of NEt_3_ at –78 °C, 91% of the thermodynamically less stable neutral *E*-**4b** isomer is formed (see Fig. 6b, Supplementary Fig. 133, and additionally Figs. 117 and 145 for the corresponding experiments with diaryl-HIs **2c** and **4c**, respectively, Supplementary Table 7). Cooling is important at this point, as locally remaining amounts of acid in the presence of already neutral bathochromic isomers lead to accelerated thermal isomerization to the hypsochromic neutral isomers at ambient temperatures. The sequence of acid and base addition thus establishes a chemical fueling process in which the thermodynamically less stable neutral *E*-**4b** isomer can be obtained almost quantitatively.

In a separate experiment, only up to 1000 equiv. of the NEt_3_ base were added after 4000 equiv. of TFA addition instead of completely neutralizing. In this case a different distinct change of the absorbance is observed, which is mainly manifested in a global hypsochromic shift. This new spectrum was assigned to the *Z*-[**4b**-H]^**+**^ species because subsequent complete neutralization with NEt_3_ fully recovers the absorbance spectrum of the thermodynamically more stable neutral *Z*-**4b** isomer (Fig. 5b and Supplementary Fig. 130). Therefore, switching between the two protonated states is possible when preparing the solution by addition of excess acid to the neutral species first (up to 4500 equiv. of TFA). When starting from *Z*-**4b**, first *Z*-[**4b**-H]^+^ is formed which quickly isomerizes to *E*-[**4b**-H]^+^ (Supplementary Figs. 128 and 129). Subsequent addition of 1000 equiv. of Et_3_N leads to the isomerization from *E*-[**4b**-H]^+^ to *Z*-[**4b**-H]^+^ (Fig. 5b and Supplementary Fig. 130).

To sum up the conducted experiments, acid-induced switching in both directions between the *E*-[**4b**-H]^+^ and *Z*-[**4b**-H]^+^ isomer, between the *E*-[**4b**-H]^+^ and *E*-**4b** isomer, and between the *Z*-[**4b**-H]^+^ and *Z*-**4b** isomer is possible. Additionally, acid-induced one directional switching between the *Z*-**4b** and *E*-[**4b**-H]^+^ isomer (Supplementary Fig. 131), between the *E*-**4b** and *Z*-[**4b**-H]^+^ isomer (Supplementary Fig. 132), and between the *E*-**4b** and *Z*-**4b** isomer is also possible establishing reversible four-state switching with high isomer enrichment in each case for **4b** (Fig. 6a and Supplementary Fig. 123).

For **1b** the neutral *Z*-**1b** isomer is only slightly more stable than *E*-**1b** (thermally equilibrated mixture in *o*-xylene-*d*_10_ of *E*:*Z* = 42:58) and cannot be enriched significantly by simple heating experiments. However, the analogous sequential acid/base addition experiment with **1b** at 23 °C (adding 5000 equiv. TFA and neutralization with 5000 equiv. NEt_3_) leads to an absorption spectrum signifying that the *Z*-**1b** isomer is populated to 85%. Because of the previously described resistance of diaryl-HIs **1** against acid-induced thermal isomerization (Supplementary Fig. 109) cooling is not necessary in this experiment.

A general trend is found, where protonation of the *p*-NMe_2_ substituent inverts (e.g., diaryl-HI **4b**) or at least pushes thermodynamic stabilities (e.g., diaryl-HI **1b**) towards the bathochromic isomers. This can be explained as follows. In its neutral form the hypsochromic *Z*-**4b** isomer is the thermodynamically more stable one, in which the carbonyl group of the indigo fragment and the strongest donor substituent (*p*-NMe_2_) are residing at the opposite side of the double bond. Protonation of *p*-NMe_2_ converts it into the strongest acceptor and the cyano-substituent (*p*-CN) into a (relatively) stronger donor substituent. As a result, the protonated bathochromic *E*-[**4b-**H]^**+**^ conformation becomes the thermodynamically preferred one placing again the strongest donor of the diaryl-moiety and the carbonyl at opposite sides of the double bond.

The two protonated species are thus spectroscopically distinct and represent a third and fourth reversibly accessible state of diaryl-HIs (Figs. 5b, c and 6b). In the protonated forms no photoswitching is possible, establishing acid-gating of photoresponsiveness. Neutralization with Et_3_N recovers the absorption profile of hypsochromic *Z*-**4b** (Fig. 5b) or bathochromic *E*-**4b** (chemical fueling resulting in 91% of thermodynamically stable isomer, Fig. 6b) and at the same time the photoswitching capacity (Fig. 5c), which evidences reversibility of acid-gating. The protonated states can also be elicited in all other diaryl-HI derivatives (Supplementary Note 8). However, it should be noted at this point that diaryl-HIs **1b** and **1c** are much less sensitive towards acid addition and retain their bistable photoswitching abilities even in the presence of significant amounts of TFA. Addition of large excess of TFA followed by neutralization with NEt_3_ results in 85% of the *Z*-**1b** isomer, which establishes chemical fueling also for this diaryl-HI derivative (Supplementary Fig. 109).

In summary, we report on a structurally expanded class of HI chromophores, which are diaryl substituted at the central double bond. Synthetic access to these compounds is straightforward and modular, such that a large variety of substitution pattern can be introduced not least for late-stage functionalization and covalent attachment of the photoswitches. The switching properties were quantified evidencing a suite of favorable properties such as visible and red light responsiveness in both switching directions, significant photochromism, high thermal bistability, strong enrichment of each isomer in the pss, and large yet defined geometry changes upon switching. Acid and base addition can be used as two further independent stimuli establishing a third and fourth spectrally distinct state of diaryl-HIs, which are the protonated *Z* and *E* isomers, respectively. Reversible switching and selective strong accumulation of each of the four states over up to three different pathways is possible depending on relative concentrations of acid and base. What is more, this system allows to strongly populate thermodynamically less stable isomers via acid/base additions, a process which can be related to chemically fueling. These multi-switching properties and the associated acid-gating of photoswitching capacity add further response dimensions and functional complexity. Application of diaryl-HIs in transparent and highly colored PS polymers showcases the versatility, multi-stimuli response, and intrinsic advantages, like color contrast and photostability, directly. Building on the results of this study it will now be possible to investigate a large and unexplored structural space as well as multi-stimuli and multi-state applications for this potent class of photoswitches.

## Methods

General materials and methods are outlined in Supplementary Note 1.

### Synthesis

The synthesis of the parent monoaryl-HIs **5-8** followed published procedures that have been modified in terms of solvents and temperatures used (Supplementary Table 1a)^32,36,65^. The selective chlorination with NCS at the stilbene part of the parent monoaryl-HIs **9-12** has not been described so far, but was inspired by different electrophilic functionalization reactions of similar compounds (Supplementary Table 1b)^54–56,66^. The Suzuki-Miyaura cross-coupling reactions of chlorinated monoaryl-HIs with boronic acids to yield diaryl-HIs **1a-4a** were inspired by the published synthesis of diaryl-HTIs but were modified for the needs of diaryl-HIs (Supplementary Table 1c)^40^. *N*-propylated diaryl-HIs **1b-4b** were prepared according to the published procedure of C. Petermayer et al. for the *N*-alkylation of monoaryl diaryl-HIs (Supplementary Table 1d)^36^. *N* functionalization with methyl propionate at the indoxyl part to yield diaryl-HIs **1c-4c** follows a published Aza-Michael addition (Supplementary Table 1e)^67^. Details on the syntheses are given in Supplementary Note 2. An overview of the synthesis route, substrate scope and yields is given in Supplementary Table 1. H NMR and ^13^C NMR spectra of all compounds are given in Supplementary Note 3. 1D ^1^H-^1^H NOE NMR spectra and 2D ^1^H-^1^H NOESY NMR spectra of compounds **1a-4a,** **1b-4b**, and **1c-4c** allow direct experimental assignments of *E* and *Z* isomers. This structural elucidation (Supplementary Note 3) together with the determination of molar absorption coefficients (Supplementary Note 4) establishes that isomers with the carbonyl group of the indoxyl fragment and the *p*-NMe_2_ substituent residing at the same side of the double bond possess bathochromically shifted absorptions.

### Determination of molar absorption coefficients of pure *E* and *Z* isomers

The molar absorption coefficients of pure *E* and *Z* isomers were obtained from samples containing *E* + *Z* isomer mixtures. First, the UV/Vis absorption spectra of pure *E* and *Z* isomers were obtained from the UV/Vis absorption spectra of two solutions with different *E* + *Z* mixtures, known isomeric compositions, and known total concentrations. To extract the individual absorption spectra of each isomer in this case, the sample must be composed of only two isomers, which photoisomerize without side reactions or decomposition. Details are given in Supplementary Note 4. A summary of the determined molar absorption coefficients for diaryl-HIs **1-4** are presented in Supplementary Table 2.

### Photoisomerization experiments using UV/Vis and NMR spectroscopy

Photoisomerization experiments were conducted with diaryl-HI solutions filled in NMR tubes or in 10 mm Hellma quartz cuvettes, which were irradiated from the outside with LEDs of different wavelengths. Solutions were prepared either with concentrations of 2 × 10^−2^ to 8 × 10^−2 ^mol L^−1^ in toluene-*d*_8_ or THF-*d*_8_ (NMR tubes) or with concentrations of 3 × 10^−5^ to 8 × 10^−5 ^mol L^−1^ in regular non-deuterated toluene, MeOH, DMSO or DMSO/H_2_O (spectroscopic grade, UV/Vis cuvettes). Experimental details and results are presented in Supplementary Note 5. A summary of the isomer compositions of diaryl-HIs **1-4** in the pss at NMR concentration is presented in Supplementary Table 3, the corresponding date obtained from UV/Vis measurements are summarized in Supplementary Table 4.

### Quantum yield determination

Determination of QYs for the hypsochromic to bathochromic photoisomerization followed the initial slope method. Therefore, QY determinations were carried out in UV/Vis cuvettes charged with *E* + *Z* isomer mixtures diluted to spectroscopic concentrations of 5 × 10^−5^ to 2 × 10^−4 ^mol L^−1^ in toluene. Details are outlined in Supplementary Note 6. A summary of measured values is given in Supplementary Table 5.

### Thermal stabilities of isomeric states

The thermal *E* to *Z* or *Z* to *E* isomerizations are described as two unimolecular elemental reactions, i.e., the thermal forward isomerization and the thermal backward isomerization. These can be seen as first-order kinetics with entering thermodynamic equilibria. From kinetic analysis the corresponding Gibbs energies of activation Δ*G*^‡^_E→Z_/Δ*G*^‡^_Z→E_ for the thermal equilibrations are obtained. From the isomer composition in thermal equilibrium the Gibbs free energy differences Δ*G* of the isomers are calculated. Thermal isomerization kinetics and relative isomer stabilies of diaryl-HIs **1-4** were measured at various temperatures in toluene-*d*_8_ or *o*-xylene-*d*_10_ solutions. Details are outlined in Supplementary Note 7. A summary of measured values is shown in Supplementary Table 6.

### Acid-induced isomerization

Acid-induced isomerization experiments at UV/Vis level were conducted with diaryl-HI solutions filled in 10 mm Hellma quartz cuvettes, which were irradiated from the outside with LEDs of different wavelengths. After addition of different equivalents of TFA the isomerization response (thermal and photochemical) of diaryl HIs **1**-**4** as well as the response to NEt_3_ addition was monitored by UV/Vis spectroscopy. Additionally, an acid titration experiment with TFA-*d*_1_ (0.1 to 135 equiv.) was performed with a thermally equilibrated *Z*/*E*-**4b** mixture at constant diaryl-HI concentration (1.12 mM) in toluene-*d*_8_ at 25 °C using ^1^H NMR spectroscopy for analysis. Beforehand, the proton signals of the neutral *E*-isomer and neutral *Z*-isomer of **4b** in toluene-*d*_8_ were assigned to the structure and the double bond configuration was elucidated through 1D ^1^H-^1^H NOE NMR experiments. The identity of the isomer reached at the end point of the titration was elucidated through a comprehensive NMR analysis in TFA-*d*_1_ solution. Additional, MS-APPI and MS-ESI mass experiments in the presence of different amounts of TFA were performed in toluene solution. Comprehensive experimental details are provided in Supplementary Note 8. A brief summary of the experimental results is presented in Supplementary Table 7.

### Isomerization within polymers

For each sample, 30 mL of neat styrene were heated to 140 °C for 50 min until polymerization was almost completed. Then, ca. 4 mg of diaryl-HI was added, and the liquid was mixed thoroughly. The liquid diaryl-HI containing polymer was then poured into petri dishes and allowed to cool to 23 °C. After 24 h, a solid and transparent PS polymer was obtained. Samples were irradiated with certain wavelengths to enrich the hypsochromic or bathochromic isomers, respectively. Different methods to erase the written inscriptions (light, acid, sunlight) were tested. Additional experiments are presented in Supplementary Note 9.

### Reporting summary

Further information on research design is available in the Nature Portfolio Reporting Summary linked to this article.

### Supplementary information

Supplementary Information

Reporting Summary

### Source data

Source Data

## Data Availability

Source data are provided with this paper. Crystal Structural Data were deposited in the Cambridge Crystallographic Data Centre with the following CCDC designations: 2191572 and 2191573. All data generated during this study are available from the corresponding author upon request.
