# Supplementary material for: Diaryl-hemiindigos as visible light, pH, and heat responsive four-state switches and application in photochromic transparent polymers
Source: Nat Commun. 2023 Jul 20;14:4382. doi: 10.1038/s41467-023-39944-x (PMC10359318; doi:10.1038/s41467-023-39944-x)
Supplement: Supplementary file 1 — Supplementary Information [file 41467_2023_39944_MOESM1_ESM.pdf]

Supplementary Information

Diaryl-hemiindigos as visible light, pH, and heat responsive four-state switches and application in photochromic transparent polymers

*Maximilian Sacherer, Frank Hampel, Henry Dube**

Supplementary Table of Contents

Supplementary Note 1: Materials and General Methods 6

Supplementary Note 2: Synthesis 9

(*Z*)-2-(2,4,6-trimethylbenzylidene)indolin-3-one (*Z*-**5**) 10

(*Z*)-2-(3,5-bis(trifluoromethyl)benzylidene)indolin-3-one (*Z*-**6**) 11

(*Z*)-2-(4-(dimethylamino)benzylidene)indolin-3-one (*Z*-**7**) 12

(*Z*)-4-((3-oxoindolin-2-ylidene)methyl)benzonitrile (*Z*-**8**) 13

2-(chloro(mesityl)methylene)indolin-3-one (**9**) 14

2-((3,5-bis(trifluoromethyl)phenyl)chloromethylene)indolin-3-one (**10**) 15

2-(chloro(4-(dimethylamino)phenyl)methylene)indolin-3-one (**11**) 16

4-(chloro(3-oxoindolin-2-ylidene)methyl)benzonitrile (**12**) 17

(*E*/*Z*)-2-((4-(dimethylamino)phenyl)(mesityl)methylene)indolin-3-one (*E*/*Z*-**1a**) 18

(*E*/*Z*)-2-((3,5-bis(trifluoromethyl)phenyl)(4-(dimethylamino)phenyl)- methylene)indolin-3-one (*E*/*Z*-**2a**) 20

(*E*/*Z*)-2-((3,5-dimethoxyphenyl)(4-(dimethylamino)phenyl)methylene)- indolin-3-one (*E*/*Z*-**3a**) 22

(*E*/*Z*)-4-((4-(dimethylamino)phenyl)(3-oxoindolin-2-ylidene)methyl)- benzonitrile (*E*/*Z*-**4a**) 24

(*E*/*Z*)-2-((4-(dimethylamino)phenyl)(mesityl)methylene)-1-propylindolin-3-one (*E*/*Z*-**1b**) 26

(*E*/*Z*)-2-((3,5-bis(trifluoromethyl)phenyl)(4-(dimethylamino)phenyl)- methylene)-1-propylindolin-3-one (*E*/*Z*-**2b**) 28

(*E*/*Z*)-2-((3,5-dimethoxyphenyl)(4(dimethylamino)phenyl)methylene)- indolin-3-one (*E*/*Z*-**3b**) 30

(*E*/*Z*)-4-((4-(dimethylamino)phenyl)(3-oxo-1-propylindolin-2-ylidene)methyl)benzonitrile (*E*/*Z*-**4b**) 32

Methyl (*E*/*Z*)-3-(2-((4-(dimethylamino)phenyl)(mesityl)methylene)-3-oxoindolin-1-yl)propanoate (*E*/*Z*-**1c**) 34

Methyl (*E*/*Z*)-3-(2-((3,5-bis(trifluoromethyl)phenyl)(4-(dimethylamino)-phenyl)methylene)-3-oxoindolin-1-yl)propanoate (*E*/*Z*-**2c**) 36

Methyl (*E*/*Z*)-3-(2-((3,5-dimethoxyphenyl)(4-(dimethylamino)phenyl)methylene)-3-oxoindolin-1-yl)propanoate (*E*/*Z*-**3c**) 38

Methyl (*E*/*Z*)-3-(2-((4-cyanophenyl)(4-(dimethylamino)phenyl)methylene)-3-oxoindolin-1-yl)propanoate (*E*/*Z*-**4c**) 40

Supplementary Note 3: NMR spectra 42

Supplementary Note 4: Determination of molar absorption coefficients of pure E and Z isomers 76

4.1 Molar absorption coefficient of diaryl-HI **1a** 81

4.2 Molar absorption coefficient of diaryl-HI **1b** 82

4.3 Molar absorption coefficient of diaryl-HI **1c** 83

4.4 Molar absorption coefficient of diaryl-HI **2b** 84

4.5 Molar absorption coefficient of diaryl-HI **2c** 85

4.6 Molar absorption coefficient of diaryl-HI **3a** 86

4.7 Molar absorption coefficient of diaryl-HI **3b** 87

4.8 Molar absorption coefficient of diaryl-HI **3c** 88

4.9 Molar absorption coefficient of diaryl-HI **4b** 89

4.10 Molar absorption coefficient of diaryl-HI **4c** 90

Supplementary Note 5: Photoisomerization experiments using UV/Vis and NMR spectroscopy 91

5.1 Photoisomerization of diaryl-HI **1a** 94

5.2 Photoisomerization of diaryl-HI **1b** 96

5.3 Photoisomerization of diaryl-HI **1c** 100

5.4 Photoisomerization of diaryl-HI **2a** 102

5.5 Photoisomerization of diaryl-HI **2b** 103

5.6 Photoisomerization of diaryl-HI **2c** 105

5.7 Photoisomerization of diaryl-HI **3a** 112

5.8 Photoisomerization of diaryl-HI **3b** 113

5.9 Photoisomerization of diaryl-HI **3c** at NMR concentrations 115

5.10 Photoisomerization of diaryl-HI **4a** 117

5.11 Photoisomerization of diaryl-HI **4b** 118

5.12 Photoisomerization of diaryl-HI **4c**... 124

Supplementary Note 6: Quantum yield determination 130

6.1 Quantum yield for the *E* to *Z* photoisomerization of diaryl-HI **2b** in toluene 136

6.2 Quantum yield for the E to Z photoisomerization of diaryl-HI **2c** in toluene 139

6.3 Quantum yield for the *Z* to *E* photoisomerization of diaryl-HI **4b** in toluene 142

6.4 Quantum yield for the *Z* to *E* photoisomerization of diaryl-HI **4c** in toluene............. 145

Supplementary Note 7: Thermal stabilities of isomeric states 148

7.1 Thermal isomerization of diaryl HI **1a**.. 156

7.2 Thermal isomerization of diaryl-HI **1b**.. 157

7.3 Thermal isomerization of diaryl-HI **1c** 158

7.4 Thermal isomerization of diaryl-HI **2a** 159

7.5 Thermal isomerization of diaryl-HI **2b**.. 160

7.6 Thermal isomerization of diaryl-HI **2c** 161

7.7 Thermal isomerization of diaryl-HI **3a** 162

7.8 Thermal isomerization of diaryl-HI **3b**.. 163

7.9 Thermal isomerization of diaryl-HI **3c** 164

7.10 Thermal isomerization of diaryl-HI **4a** 165

7.11 Thermal isomerization of diaryl-HI **4b**.. 166

7.12 Thermal isomerization of diaryl-HI **4c** 167

Supplementary Note 8: Acid induced isomerization of diaryl-HIs 168

8.1 Acid induced isomerization of diaryl-HI **1a** 173

8.2 Acid induced isomerization of diaryl-HI **1b** 174

8.3 Acid induced isomerization of diaryl-HI **1c**... 179

8.4 Acid induced isomerization of diaryl-HI **2b** 180

8.5 Acid induced isomerization of diaryl-HI **2c**... 183

8.6 Acid induced isomerization of diaryl-HI **3a** 185

8.7 Acid induced isomerization of diaryl-HI **3b** 186

8.8 Acid induced isomerization of diaryl-HI **3c**.. 189

8.9 Acid induced isomerization of diaryl-HI **4b** 190

8.10 Acid induced isomerization of diaryl-HI **4c**.. 217

Supplementary Note 9: Isomerization of diaryl-HIs within polymers 219

Supplementary Note 10: Crystal Structural Data 222

Supplementary References... 223

Supplementary Note 1: Materials and General Methods

Reagents and solvents were obtained from abcr, Acros Organics, Fluka, Merck or Tokyo Chemical Industry in the qualities puriss., p.a. or purum and used as received. Solvents of technical purity were further purified by distillation with a rotary evaporator (KNF LAB RC600) before use for chromatography or extraction. Anhydrous solvents purchased from Merck, Fluka and Acros were used without further purification. Reaction progress monitoring of all reactions and analysis of chromatography fractions were performed by thin-layer chromatography (TLC) using aluminum plates coated with SiO_2 (Merck, TLC silica gel 60 F_{254}), Al_2O_3 (Macherey-Nagel, ALUGRAM ALOX N / UV_{254}), SiO_2 C_{18} with fluorescent indicator (Macherey-Nagel, ALUGRAM RP-18 W/UV_{254}) or by ^1H NMR spectroscopy. Detection of TLC probes were done by irradiation with UV light (254 nm or 366 nm) in order to determine the retardation factors (R_f). Reaction progress monitoring by ^1H NMR spectroscopy was done by the removal of volatiles of the reaction mixture under reduced pressure and resolving it with deuterated solvents.

General working procedures. All reactions were carried out in Schlenk tubes or flasks under argon atmosphere. All Schlenk tubes and flasks were flame dried and flushed with argon three times before usage. Syringes and cannulas for reagents and solvents were flushed three times with argon. Solvents were subjected to an argon/air exchange by injection of an argon flux for at least 10 minutes. Solvents used for particularly air sensitive reactions have been flushed with argon for at least 1 h. After transferring solids into Schlenk tubes or flasks, they have been set alternatingly under vacuum and argon three times. Condensation reactions were carried out in three neck flasks equipped with a magnetic stirring bar, a reflux cooler, a rubber septum and a vacuum adapter. All other reactions were carried out in Schlenk flasks or tubes equipped with a magnetic stirring bar and a rubber septum.

Column chromatography was performed with SiO_2 (Merck, particle size 0.063- 0.200 mm), Al_2O_3 activated basic Brockmann I (Merck, particle size 0.040-0.160 mm) or Al_2O_3 90 standard (Merck, particle size 0.063- 0.200 mm) solid stationary phases.

Medium pressure liquid chromatography (MPLC) was performed on an Isolera™ One or an Isolera™ Selekt from Biotage. As stationary phases SiO_2 (Macherey-Nagel, M-N Silica Gel 60A, 230 – 400 mesh), Al_2O_3 (Macherey-Nagel, M-N Aluminum Oxide Neutral, 90A, 50 – 200

mesh) and SiO_2 C_{18} (Macherey-Nagel, CHROMABOND Flash RS 15 C18 ec) were used. If not stated otherwise MPLC was used as standard method for column chromatography purification.

^{1}H NMR and ^{13}C NMR spectra were recorded on a Bruker Avance NEO HD 400 MHz, Bruker Avance Neo HDX 500 MHz, or Bruker Avance Neo HDX 600 MHz with cryo probe DCH-Z^{13}C/^{1}H spectrometer. Deuterated solvents were obtained from Cambridge Isotope Laboratories, Deutero GmbH, and Eurisotop and used without further purification. Chemical shifts (δ) are reported in parts per million (ppm) relative to tetramethylsilane as external standard. Residual solvent signals in the ^{1}H and ^{13}C NMR spectra were used as internal reference. CD_2Cl_2: δ_H = 5.320 ppm, δ_C= 53.84 ppm; $CDCl_3$: δ_H = 7.260 ppm, δ_C= 77.16 ppm; toluene-d_8: δ_H = 2.080 ppm; *o*-xylene-d_{10}: δ_H = 2.220 ppm. The resonance multiplicity is reported as s (singlet), d (doublet), t (triplet), q (quartet), and m (multiplet). The chemical shifts are given in parts per million (ppm) on the delta scale (δ) and the coupling constant values (*J*) are given in Hertz (Hz). Individual atoms of *Z* isomeric diaryl-hemiindigos (diaryl-HIs) are assigned with arbitrary numbers and an apostrophe as postfix, e.g. H5' for the proton at position 5. The corresponding *E* isomers adhere to the same labelling system but without an apostrophe.

Mass spectrometry as Atmospheric Pressure Photoionization (APPI) and High Resolution Atmospheric Pressure Photoionization Mass Spectrometry (HR-MS APPI) were conducted on a MicroTOF II spectrometer. The molecular ion mass is reported as *m*/*z*.

Infrared spectra were recorded on a Perkin Elmer Spectrum BX-FT-IR instrument equipped with a Smith DuraSample IR II ATR-device and a Varian 660-IR ATR mode. Transmittance values are qualitatively described by wavenumber (cm^{-1}) as very strong (*vs*), strong (*s*), medium (*m*), weak (*w*) and very weak (*vw*).

Melting points (mp) were measured on a Büchi B-540 melting point apparatus in open capillaries.

UV/Vis spectra were measured on a Varian Cary 5000 UV/Vis-NIR and an Agilent Technologies Cary 60 UV/Vis spectrophotometer. The spectra were recorded in a quartz cuvette (pathlength 1 cm). Spectral grade solvents were obtained from VWR and Merck. Absorption wavelengths (λ) are reported in nm, the molar absorption coefficients (*ε*) in L mol^{-1} cm^{-1}, and absorbance *A* in abs. u.

Quantum yields were measured in UV/Vis cuvettes on a quantum yield set-up similar to a previously reported version by the E. Riedle group.[1]

Photoisomerization experiments. Irradiations were conducted either by irradiating NMR tubes filled with diaryl-HIs and deuterated solvents (toluene-d_8 or *o*-xylene-d_{10}) or irradiating UV/Vis cuvettes filled with diaryl-HIs and protonated solvents. Photoreactions were subsequently analyzed either by ^{1}H NMR or UV/Vis absorption spectroscopy. Irradiations of cuvettes were conducted using LEDs from Thorlabs Inc. and Roithner Lasertechnik GmbH. For irradiation of NMR tubes either Prismatix UHP LEDs, Mightex UHP LEDs, or LEDs of Thorlabs Inc. and Roithner Lasertechnik GmbH were used as light sources. When using UHP LEDs, the NMR tube was immersed in a cooling bath inside a Dewar to maintain a constant temperature during irradiation.

X-ray diffraction of single crystals were performed on a SuperNova Atlas diffractometer using Cu-K_α-radiation.

Supplementary Note 2: Synthesis

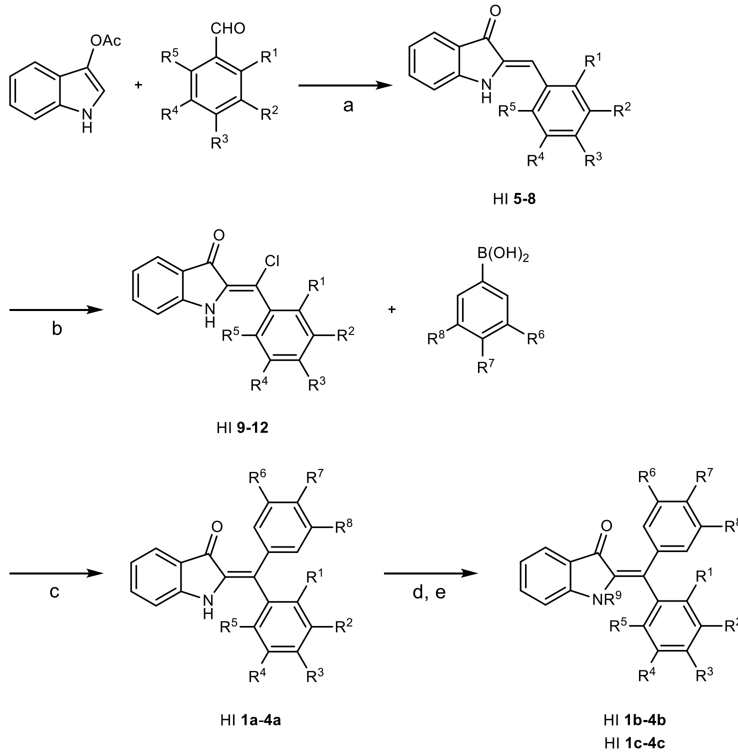

HI	R^1	R^2	R^3	R^4	R^5	R^6	R^7	R^8	R^9	Yield (%)
5	CH	H	CH_3	H	CH_3	-	-	-	-	86
6	H	CF_3	H	CF_3	H	-	-	-	-	87
7	H	H	$N(Me)_2$	H	H	-	-	-	-	91
8	H	H	CN	H	H	-	-	-	-	100
9	CH	H	CH_3	H	CH_3	-	-	-	-	95
10	H	CF_3	H	CF_3	H	-	-	-		59
11	H	H	$N(Me)_2$	H	H	-	-	-	-	25
12	H	H	CN	H	H	-	-	-	-	78
1a	CH	H	CH_3	H	CH_3	H	$N(Me)_2$	H	-	80
1b	CH	H	CH_3	H	CH_3	H	$N(Me)_2$	H	*n*-propyl	100
1c	CH	H	CH_3	H	CH_3	H	$N(Me)_2$	H	*n*-propyl-CO_2Me	73
2a	H	H	$N(Me)_2$	H	H	CF_3	H	CF_3	-	49
2b	H	H	$N(Me)_2$	H	H	CF_3	H	CF_3	*n*-propyl	64
2c	H	H	$N(Me)_2$	H	H	CF_3	H	CF_3	*n*-propyl-CO_2Me	41
3a	H	H	$N(Me)_2$	H	H	OMe	H	OMe	-	80
3b	H	H	$N(Me)_2$	H	H	OMe	H	OMe	*n*-propyl	61
3c	H	H	$N(Me)_2$	H	H	OMe	H	OMe	*n*-propyl-CO_2Me	53
4a	H	H	$N(Me)_2$	H	H	H	CN	H	-	31
4b	H	H	$N(Me)_2$	H	H	H	CN	H	*n*-propyl	76
4c	H	H	$N(Me)_2$	H	H	H	CN	H	*n*-propyl-CO_2Me	54

Supplementary Table 1: Overview of the syntheses of monoaryl-HIs **5-8**, chlorinated parent monoaryl-HIs **9-12**, diaryl-HIs **1a-4a**, and *N* functionalized diaryl-HIs **1b-4b** and **1c-4c**. **a** Aldol condensation reaction; **b** electrophilic halogenation at the stilbene fragment using NCS; **c** Suzuki-Miyaura cross coupling; **d** *N*-alkylation via S_N2 reaction using 1-iodopropane; **e** *N* alkylation via Aza-Michael addition using methyl propionate.

(Z)-2-(2,4,6-trimethylbenzylidene)indolin-3-one (Z-5)

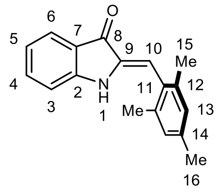

1*H*-Indol-3-yl acetate (2.0 g, 11 mmol, 1.0 equiv.) was added to a $NaOH_{aq.}$ solution (1.5 M, 62 mL, 3.7 g, 94 mmol, 8.2 equiv.). The green solution was heated under reflux for 15 min. Then, a 2,4,6-trimethylbenzaldehyde solution in 1,4-dioxane (1.0 M, 11 mL, 1.7 g, 11 mmol, 1.0 equiv.) was added. The newly formed yellow suspension was stirred under reflux for 13 h. The reaction progress was monitored by thin-layer chromatography (Al_2O_3, *i*Hex : EtOAc 12 : 1). The mixture was neutralized with a $HCl_{aq.}$ solution (1.0 M) and a saturated $NaCl_{aq.}$ solution (100 mL) was added. The aqueous phase was extracted with EtOAc (4 × 100 mL). The combined organic phases were dried over anhydrous Na_2SO_4 and the volatiles were removed *in vacuo*. The crude product was dissolved in CH_2Cl_2, adsorbed on Al_2O_3 and subsequently purified by column chromatography (ALOX N, *i*Hex : EtOAc 100 : 0 → 93 : 7) and recrystallized from MeOH/H_2O to obtain *Z*-**5** as deep orange fine powdered crystals (2.6 g, 9.9 mmol, 86%).

^{1}H NMR (400 MHz, CD_2Cl_2, 23 °C) δ (ppm) = 7.66 (ddd, $^3J_{H\text{-}H}$ = 7.7 Hz, $^4J_{H\text{-}H}$ = 1.5 Hz, $^5J_{H\text{-}H}$ = 0.8 Hz 1H, H6), 7.43 (ddd, $^3J_{H\text{-}H}$ = 8.4 Hz, $^3J_{H\text{-}H}$ = 7.2 Hz, $^4J_{H\text{-}H}$ = 1.3 Hz, 1H, H4), 6.95 – 6.89 (m, 3 H, H5, H13), 6.86 – 6.82 (m, 2H, H3, H10), 6.20 (s, 1H, H1), 2.30 (s, 3H, H16), 2.24 (s, 6H, H15); **^{13}C NMR** (151 MHz, $CDCl_3$, 23 °C) δ (ppm) = 185.6 (C8), 153.0 (C2), 138.0 (C14), 137.0 (C9), 136.9 (2C, C12), 136.4 (C4), 129.8 (C11), 128.8 (2C, C13), 125.2 (C6), 122.1 (C7), 120.2 (C5), 111.6 (C3), 111.1 (C10), 21.2 (C16), 20.6 (2C, C15); **IR**: ṽ (cm^{-1}) = 3745*vw*, 3628*vw*, 3509*vw*, 3244*s*, 3089*w*, 2952*w*, 2913*w*, 2854*w*, 2800*w*, 2728*vw*, 2661*vw*, 2585*vw*, 2514*vw*, 2447*vw*, 2386*vw*, 2313*vw*, 2257*w*, 2237*w*, 2200*w*, 2154*w*, 2131*vw*, 2099*vw*, 2071*vw*, 2045*vw*, 2018*vw*, 1998*w*, 1946*vw*, 1871*vw*, 1790*vw*, 1683*s*, 1606*vs*, 1489*s*, 1469*s*, 1404*m*, 1359*s*, 1293*s*, 1258*s*, 1195*s*, 1157*s*, 1130*vs*, 1096*s*, 1029*m*, 967*m*, 891*m*, 848*s*, 815*m*, 743*s*, 704*s*, 670*s*, 644*m*, 608*m*, 567*m*, 545*m*, 509*w*, 484*m*, 471*m*, 410*m*; **mp**: 179-181 °C; **HR-MS** (APPI$^+$, *m*/*z*): $[MH]^+$ calculated for $[C_{18}H_{18}NO]^+$: 264.1383, found 264.1378; **R_f** (Al_2O_3, *i*Hex : EtOAc 12 : 1) = 0.32.

(*Z*)-2-(3,5-bis(trifluoromethyl)benzylidene)indolin-3-one (*Z*-6)

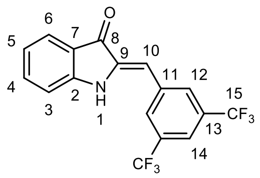

1*H*-Indol-3-yl acetate (2.0 g, 11 mmol, 1.0 equiv.) was added to a $NaOH_{aq.}$ solution (2.0 M, 47 mL, 3.7 g, 94 mmol, 8.2 equiv.). The green solution was heated under reflux for 15 min. Then, a 3,5-bis(trifluormethyl)benzaldehyde solution in 1,4-dioxane (1.0 M, 1.9 mL, 2.8 g, 11 mmol, 1.0 equiv.) was added at 0 °C and stirred for 15 min at 0 °C. The newly formed yellow suspension was stirred at 23 °C for 1.5 h. The reaction progress was monitored by thin-layer chromatography (SiO_2, *i*Hex : EtOAc 6 : 1). The mixture was neutralized with a $HCl_{aq.}$ solution (1.0 M) and a saturated $NaCl_{aq.}$ solution (100 mL) was added. The aqueous phase was extracted with EtOAc (4 × 100 mL). The combined organic phases were dried over anhydrous Na_2SO_4 and the volatiles were removed *in vacuo*. The crude product was recrystallized from $MeOH/H_2O$ to obtain *Z*-**6** as deep orange fine powdered crystals (3.6 g, 9.9 mmol, 87%).

^{1}**H NMR** (400 MHz, CD_2Cl_2, 23 °C) δ (ppm) = 7.98 (s, 2H, H12), 7.83 (s, 1H, H14), 7.71 (dd, $^{3}J_{H\text{-}H}$ = 7.7, $^{4}J_{H\text{-}H}$ = 1.3 Hz, 1H, H6), 7.54 (ddd, $^{3}J_{H\text{-}H}$ = 8.3 Hz, $^{3}J_{H\text{-}H}$ = 7.2, $^{4}J_{H\text{-}H}$ 1.4 Hz, 1H, H4), 7.10 (d, $^{3}J_{H\text{-}H}$ = 8.1 Hz, 1H, H3), 7.07 – 6.93 (m, 2H, H1, H5), 6.75 (s, 1H, H10); ^{13}**C NMR** (126 MHz, CD_2Cl_2, 23 °C) δ (ppm) = 186.5 (C8), 153.7 (C2), 137.6 (C11), 137.2 (C4), 137.3 (C9), 132.7 (2C, C13), 129.3 (2C, C12), 125.3 (C6), 123.7 (2C, C15), 121.9 (C5), 121.8 (C7), 121.7 (C14), 112.8 (C3), 106.8 (C10); **IR:** ṽ (cm^{-1}) = 3387*w*, 1693*m*, 1615*s*, 1596*s*, 1490*m*, 1471*m*, 1407*vw*, 1356*s*, 1277*vs*, 1253*m*, 1166*s*, 1117*vs*, 998*m*, 983*s*, 901*s*, 890*s*, 845*m*, 783*w*, 746*s*, 733*w*, 708*s*, 693*s*, 681*s*, 658*m*, 636*m*, 561*s*, 509*m*, 446*m*; **mp:** 150 – 153 °C; **HR-MS** (APPI^{+}, *m/z*): $[MH]^{+}$ calculated for $[C_{17}H_{10}F_6NO]^{+}$: 358.0661, found 358.0658; $\mathbf{R_f}$ (SiO_2, *i*Hex : EtOAc 6 : 1) = 0.50.

(*Z*)-2-(4-(dimethylamino)benzylidene)indolin-3-one (*Z*-7)

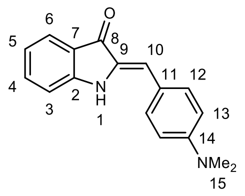

1*H*-Indol-3-yl acetate (2.0 g, 11 mmol, 1.0 equiv.) was added to a $NaOH_{aq.}$ solution (1.5 M, 62 mL, 3.7 g, 94 mmol, 8.2 equiv.). The green solution was heated under reflux for 15 min. Then, a 4-(dimethylamino)benzaldehyde solution in 1,4-dioxane (1.0 M, 1.6 mL, 1.7 g, 11 mmol, 1.0 equiv.) was added under reflux and stirred for 1 h under reflux. The newly formed red crystalline precipitate was stirred for another 1 h at 23 °C. The reaction progress was monitored by thin-layer chromatography (SiO_2, *i*Hex : EtOAc 3 : 1). The mixture was neutralized with a $HCl_{aq.}$ solution (1.0 M) and a saturated $NaCl_{aq.}$ solution (100 mL) was added. The aqueous phase was extracted with EtOAc (4 × 100 mL). The combined organic phases were dried over anhydrous Na_2SO_4 and the volatiles were removed *in vacuo*. The crude product was recrystallized from MeOH/H_2O to obtain *Z*-**7** as deep red fine powdered crystals (2.8 g, 10 mmol, 91%).

^{1}H NMR (601 MHz, CD_2Cl_2, 25 °C) δ (ppm) = 7.68 (ddt, $^3J_{H\text{-}H}$ = 7.7 Hz, $^4J_{H\text{-}H}$ = 1.4 Hz, $^5J_{H\text{-}H}$ = 0.7 Hz, 1H, H6), 7.50 – 7.48 (m, 2H, H12), 7.46 (ddd, $^3J_{H\text{-}H}$ = 8.1 Hz, $^3J_{H\text{-}H}$ = 7.2 Hz, $^4J_{H\text{-}H}$ = 1.3 Hz, 1H, H4), 7.06 (d, $^3J_{H\text{-}H}$ = 8.1 Hz, 1H, H3), 6.96 (ddd, $^3J_{H\text{-}H}$ = 7.8 Hz, $^3J_{H\text{-}H}$ = 7.2, $^4J_{H\text{-}H}$ = 0.8 Hz, 1H, H5), 6.85 (s, 1H, H1), 6.81 (s, 1H, H10), 6.79 – 6.75 (m, 2H, H13), 3.03 (s, 6H, H15); **^{13}C NMR** (151 MHz, CD_2Cl_2, 25 °C) δ (ppm) = 185.9 (C8), 153.1 (C2), 151.0 (C14), 135.6 (C4), 133.3 (C9), 131.7 (2C, C12), 124.6 (C6), 122.7 (C7), 122.4 (C11), 120.5 (C5), 113.9 (C10), 112.7 (2C, C13), 112.6 (C3), 40.3 (2C, C15); **IR**: ṽ (cm^{-1}) = 3220*m*, 2892*vw*, 16555*m*, 1603*m*, 1524*s*, 1486*m*, 1462*s*, 1434*s*, 1369*s*, 1317*s*, 1301*s*, 1261*m*, 1230*s*, 1185*s*, 1155*s*, 1123*s*, 1097*s*, 1064*s*, 947*m*, 932*m*, 892*s*, 857*m*, 828*m*, 807*m*, 790*s*, 753*s*, 727*s*, 707*s*, 687*s*, 561*s*, 595*s*, 520*s*, 50*s*, 488*s*, 471*s*; **mp**: 237 – 238 °C; **HR-MS** ($APPI^+$, *m*/*z*): $[MH]^+$ calculated for $[C_{17}H_{17}N_2O]^+$: 265.1335, found 265.1335; **R_f** (SiO_2, *i*Hex : EtOAc 3 : 1) = 0.25.

(Z)-4-((3-oxoindolin-2-ylidene)methyl)benzonitrile (Z-8)

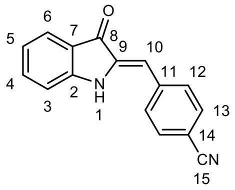

1*H*-Indol-3-yl acetate (0.50 g, 2.9 mmol, 1.0 equiv.) was added to a $NaOH_{aq.}$ solution (1.5 M, 16 mL, 0.94 g, 23 mmol, 8.2 equiv.). The green solution was heated under reflux for 15 min. Then, a 4-formylbenzonitrile solution in 1,4-dioxane (1.0 M, 0.37 g, 2.9 mmol, 1.0 equiv.) was added at 0 °C. The newly formed clay red precipitate was stirred for 12 h at 23 °C. The reaction progress was monitored by thin-layer chromatography (SiO_2, *i*Hex : EtOAc 5 : 1). The mixture was neutralized with a $HCl_{aq.}$ solution (1.0 M) and a saturated $NaCl_{aq.}$ solution (50 mL) was added. The aqueous phase was extracted with EtOAc (4 × 50 mL). The combined organic layers were dried over anhydrous Na_2SO_4 and the volatiles were removed *in vacuo*. The crude product was recrystallized from MeOH/H_2O to obtain *Z*-**8** as clay red fine powdered crystals (0.70 g, 2.9 mmol, quant.).

^{1}H NMR (601 MHz, $CDCl_3$, 25 °C) δ (ppm) = 7.76 (ddt, $^3J_{H-H}$ = 7.6 Hz, 1H, H6), 7.73 – 7.70 (m, 2H, H13), 7.63 – 7.60 (m, 2H, H12), 7.52 (ddd, $^3J_{H-H}$ = 8.0 Hz, $^3J_{H-H}$ = 7.3 Hz, $^4J_{H-H}$ = 1.3 Hz, 1H, H4), 7.04 – 7.00 (m, 2H, H3, H5), 6.85 (bs, 1H, H1), 6.77 (s, 1H, H10); **^{13}C NMR** (151 MHz, $CDCl_3$, 25 °C) δ (ppm) = 186.4 (C8), 153.2 (C2), 139.7 (C15), 136.9 (C9), 136.8 (C4), 133.0 (2C, C13), 129.8 (2C, C12), 125.5 (C6), 121.6 (C7), 121.6 (C5), 118.7 (C11), 112.2 (C3), 111.4 (C14), 108.3 (C10); **IR**: ṽ (cm^{-1}) = 3360*m*, 2220*m*, 1930*vw*, 1756*w*, 1686*s*, 1592*vs*, 1539*m*, 1506*m*, 1485*s*, 1463*s*, 1418*m*, 1380*m*, 1313*s*, 1293*s*, 1252*s*, 1211*m*, 1191*m*, 1157*m*, 1130*s*, 1097*s*, 1077*s*, 1011*m*, 962*m*, 922*w*, 891*w*, 879*w*, 860*m*, 828*m*, 749*vs*, 709*s*, 682*s*, 634*w*, 573*s*, 508*s*, 463*s*, 457*s*, 452*s*, 439*s*, 434*s*, 429*s*, 424*s*; **mp**: 241 – 243 °C; **HR-MS** ($APPI^+$, *m*/*z*): $[MH]^+$ calculated for $[C_{16}H_{11}N_2O]^+$: 247.0866, found 247.0871; **R$_f$** (SiO_2, *i*Hex : EtOAc 5 : 1) = 0.15.

2-(chloro(mesityl)methylene)indolin-3-one (9)

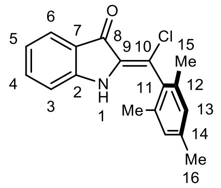

NCS (0.15 g, 1.2 mmol, 1.2 equiv.) was added to a solution of *Z*-**5** (0.25 g, 0.96 mmol, 1.0 equiv.) in MeOH : CH_2Cl_2 (29 mM, 34 mL, 20 : 1). The solution was stirred for 4 d at 23 °C. The reaction progress was monitored by ^{1}H NMR spectroscopy. The reaction was stopped by addition of H_2O (50 mL) and the product was extracted with CH_2Cl_2 (4 × 50 mL). The combined organic phases were washed with a saturated $NaHCO_3$ $_{aq.}$ solution (50 mL), dried over anhydrous Na_2SO_4 and the volatiles were removed *in vacuo*. The crude product was dissolved in CH_2Cl_2, adsorbed on Celite and subsequently purified by column chromatography (SiO_2, *i*Hex : EtOAc + 1 % NEt_3 100 : 0 → 87 : 13) to obtain **9** as an orange solid (0.27 g, 0.91 mmol, 95%).

^{1}H NMR (400 MHz, CD_2Cl_2, 23 °C) δ (ppm) = 7.53 (ddd, $^3J_{H\text{-}H}$ = 7.7 Hz, $^4J_{H\text{-}H}$ = 1.5 Hz, $^5J_{H\text{-}H}$ = 0.7 Hz, 1H, H6), 7.49 (ddd, $^3J_{H\text{-}H}$ = 8.4 Hz, $^3J_{H\text{-}H}$ = 7.3 Hz, $^4J_{H\text{-}H}$ = 1.3 Hz, 1H, H4), 7.02 (dt, $^3J_{H\text{-}H}$ = 8.2 Hz, $^4J_{H\text{-}H}$ = 0.8 Hz, 1H, H3), 6.96 (s, 2H, H13), 6.92 (dt, $^3J_{H\text{-}H}$ = 7.5 Hz, $^4J_{H\text{-}H}$ = 0.8 Hz, 1H, H5), 6.87 (s, 1H, H1), 2.33 (s, 3H, H6), 2.20 (s, 6H, H15); **^{13}C NMR** (151 MHz, CD_2Cl_2, 23 °C) δ (ppm) = 182.0 (C8), 152.0 (C2), 139.7 (C14), 137.5 (2C, C12), 136.7 (C4), 135.0 (C9), 132.0 (C11), 128.9 (2C, C13), 125.1 (C6), 123.0 (C7), 121.8 (C10), 120.7 (C5), 111.9 (C3), 21.4 (C16), 19.6 (2C, C15); **IR**: ṽ (cm^{-1}) = 3283*m*, 3063*vw*, 2974*vw*, 2915*vw*, 2855*vw*, 2730*vw*, 2671*vw*, 2549*vw*, 2458*vw*, 2415*vw*, 2279*vw*, 2232*vw*, 2144*vw*, 2043*vw*, 2025*vw*, 1955*vw*, 1888*vw*, 1849*vw*, 1811*vw*, 1696*s*, 1626*s*, 1598*vs*, 1487*s*, 1463*s*, 1390*m*, 1375*m*, 1303*s*, 1270*m*, 1229*m*, 1208*vs*, 1140*s*, 1095*s*, 1030*m*, 1015*m*, 986*m*, 956*m*, 903*s*, 846*s*, 797*w*, 744*w*, 750*vs*, 734*s*, 712*s*, 677*m*, 638*m*, 576*m*, 534*m*, 505*m*, 485*m*, 424*m*; **mp**: 236 - 238 °C; **HR-MS** (APPI$^+$, *m/z*): [MH]$^+$ calculated for [$C_{18}H_{17}ClNO$]+ : 298.0999, found 298.0997; **R$_f$** (SiO_2, *i*Hex : EtOAc 10 : 1) = 0.20.

2-((3,5-bis(trifluoromethyl)phenyl)chloromethylene)indolin-3-one (10)

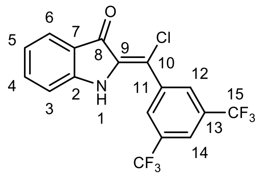

NCS (0.67 g, 5.0 mmol, 1.2 equiv.) was added to a solution of *Z*-**6** (1.5 g, 4.2 mmol, 1.0 equiv.) in THF (0.4 M, 10 mL). The solution was stirred for 3 d at 23 °C. The reaction progress was monitored by thin-layer chromatography (ALOX N, *i*Hex : EtOAc 12 : 1). The reaction was stopped by addition of H_2O (100 mL) and the product was extracted with EtOAc (4 × 100 mL). The combined organic phases were washed with a saturated $NaHCO_3$ aq. solution (100 mL), dried over anhydrous Na_2SO_4 and the volatiles were removed *in vacuo*. The crude product was dissolved in EtOAc, adsorbed on Al_2O_3 basic and subsequently purified by column chromatography (Al_2O_3 basic, *i*Hex : EtOAc 100 : 00 → 0 : 100) to obtain **10** as red fine powdered crystals (0.97 g, 2.5 mmol, 59%).

^{1}H NMR (601 MHz, CD_2Cl_2, 25 °C) δ (ppm) = 8.13 (s, 2H, H12), 7.92 (s, 1H, H14), 7.61 (ddd, $^3J_{H-H}$ = 7.7 Hz, $^4J_{H-H}$ = 1.4 Hz, $^5J_{H-H}$ = 0.7 Hz, 1H, H6), 7.52 (ddd, $^3J_{H-H}$ = 8.1, $^3J_{H-H}$ = 7.3, $^4J_{H-H}$ = 1.3 Hz, 1H, H4), 7.02 (dt, $^3J_{H-H}$ = 8.1, $^4J_{H-H}$ = 0.8 Hz, 1H, H3), 7.02 (bs, 1H, H1), 6.97 (dt, $^3J_{H-H}$ = 7.5 Hz, $^4J_{H-H}$ = 0.8 Hz, 1H, H5); **^{13}C NMR** (151 MHz, CD_2Cl_2, 25 °C) δ (ppm) = 181.8 (C8), 151.5 (C2), 137.4 (C4), 136.6 (C11), 135.0 (C9), 131.4 (q, $^2J_{C-F}$ = 33.5 Hz, 2C, C13), 130.5 (q, $^3J_{C-F}$ = 3.9 Hz, 2C, C12), 125.6 (C6), 123.7 (q, $^1J_{C-F}$ = 272.6 Hz, 2C, C15), 123.5 (p, $^4J_{C-F}$ = 3.9 Hz, C14), 122.9 (C7), 121.5 (C5), 118.3 (C10), 112.1 (C3); **IR**: ṽ (cm^{-1}) =3331*w*, 1680*s*, 1623*m*, 1570*m*, 1485*m*, 1467*m*, 1368*m*, 1318*w*, 1274*s*, 1216*m*, 1173*s*, 1142*s*, 1122*s*, 1097*s*, 1016*m*, 1000*m*, 970*m*, 910*m*, 895*m*, 877*m*, 846*m*, 786*m*, 751*s*, 721*m*, 709*m*, 696*m*, 681*m*, 663*m*, 648*m*, 533*m*, 499*s*, 460*m*; **mp**: 159 – 160 °C; **HR-MS** (APPI$^+$, *m*/*z*): [MH]$^+$ calculated for $[C_{17}H_9ClF_6NO]^+$: 392.0271, found 392.0271; **R$_f$** (ALOX N, *i*Hex : EtOAc 12 : 1) = 0.27.

2-(chloro(4-(dimethylamino)phenyl)methylene)indolin-3-one (11)

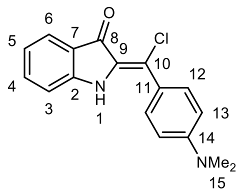

NCS (1.5 g, 11 mmol, 1.2 equiv.) was added to a solution of *Z*-**7** (2.5 g, 9.5 mmol, 1.0 equiv.) in THF (0.5 M, 19 mL). The solution was stirred for 7 d at 23 °C. The reaction progress was monitored by thin-layer chromatography (SiO_2, *i*Hex : EtOAc 5 : 1). The reaction was stopped by addition of H_2O (100 mL) and the product was extracted with CH_2Cl_2 (4 × 100 mL). The combined organic phases were washed with a saturated $NaHCO_{3\ aq.}$ solution (100 mL), dried over anhydrous Na_2SO_4 and the volatiles were removed *in vacuo*. The crude product was dissolved in CH_2Cl_2, adsorbed on Celite, subsequently purified by column chromatography (SiO_2, *i*Hex : EtOAc 85 : 15 → 60 : 40) and recrystallized from CH_2Cl_2/heptane to obtain **11** as red fine powdered crystals (0.70 g, 2.3 mmol, 25%).

^{1}H NMR (601 MHz, CD_2Cl_2, 25 °C) δ (ppm) = 7.61 – 7.57 (m, 3H, H6, H12), 7.45 (ddd, $^3J_{H\text{-}H}$ = 8.3 Hz, $^3J_{H\text{-}H}$ = 7.2 Hz, $^4J_{H\text{-}H}$ = 1.3 Hz, 1H, H4), 6.99 (d, $^3J_{H\text{-}H}$ = 8.2 Hz, 1H, H3), 6.94 (bs, 1H, H1), 6.90 (ddd, $^3J_{H\text{-}H}$ = 7.9 Hz, $^3J_{H\text{-}H}$ = 7.2 Hz, $^4J_{H\text{-}H}$ = 0.8 Hz, 1H, H5), 6.72 (m, 2H, H13), 3.03 (s, 6H, H15). **^{13}C NMR** (151 MHz, CD_2Cl_2, 25 °C) δ (ppm) = 181.3 (C8), 151.1 (C14), 150.9 (C2), 136.0 (C4), 132.2 (C9), 131.4 (2C, C12), 130.6 (C10), 125.0 (C6), 123.7 (C7), 122.7 (C11), 120.3 (C5), 111.8 (C3), 111.3 (2C, C13), 40.52 (2C, C15). **IR**: ṽ (cm^{-1}) = 3303*w*, 2893*vw*, 2172*vw*, 2157*vw*, 1967*vw*, 1658*m*, 1595*s*, 1547*s*, 1522*s*, 1484*s*, 1463*s*, 1437*w*, 1389*w*, 1365*s*, 1314*m*, 1298*m*, 1278*m*, 12229*m*, 1248*m*, 1187*s*, 1141*s*, 1095s, 1061*s*, 1015*w*, 977*m*, 950*m*, 918*m*, 865m, 814*s*, 770*s*, 746*s*, 724*s*, 708*s*, 647*m*, 627*m*, 590*s*, 552*s*, 523*s*, 507*s*, 432*s*; **mp**: 145 – 147 °C; **HR-MS** ($APPI^+$, *m/z*): $[MH]^+$ calculated for $[C_{17}H_{16}ClN_2O]^+$: 299.0946, found 299.0945. **R_f** (SiO_2, *i*Hex : EtOAc 5 : 1) = 0.30.

4-(chloro(3-oxoindolin-2-ylidene)methyl)benzonitrile (12)

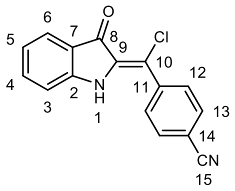

N-Chlorosuccinimide (NCS, 0.85 g, 6.3 mmol, 1.2 equiv.) was added to a solution of *Z*-**8** (1.3 g, 5.3 mmol, 1.0 equiv.) in THF (0.2 M, 26 mL). The solution was stirred for 48 h at 23 °C. The reaction progress was monitored by thin-layer chromatography (SiO_2, *i*Hex : EtOAc 5 : 1). The reaction was stopped by addition of H_2O (50 mL) and the product was extracted with CH_2Cl_2 (4 × 50 mL). The combined organic phases were washed with a saturated $NaHCO_{3\,aq.}$ solution (50 mL), dried over anhydrous Na_2SO_4 and the volatiles were removed *in vacuo*. The crude product was dissolved in CH_2Cl_2, adsorbed on Celite, subsequently purified by column chromatography (SiO_2, *i*Hex : EtOAc 100 : 0 → 83 : 17) and recrystallized from MeOH/H_2O to obtain **12** as an orange solid (1.2 g, 4.2 mmol, 78%).

^{1}H NMR (601 MHz, CD_2Cl_2, 25 °C) δ (ppm) = 7.78 – 7.74 (m, 2H, H12), 7.72 – 7.68 (m, 2H, H13), 7.58 (d, $^3J_{H\text{-}H}$ = 7.7 Hz, 1H, H6), 7.53 – 7.49 (m, 1H, H4), 7.04 – 6.99 (m, 2H, H3, H1), 6.96 (dt, $^3J_{H\text{-}H}$ = 7.5 Hz, $^4J_{H\text{-}H}$ = 0.8 Hz, 1H, H5). **^{13}C NMR** (151 MHz, CD_2Cl_2, 25 °C) δ (ppm) = 181.8 (C8), 151.5 (C2), 139.1 (C15), 137.2 (C4), 134.8 (C9), 132.1 (2C, C13), 130.8 (2C, C12), 125.5 (C6), 123.0 (C7), 121.3 (C5), 120.1 (C10), 118.9 (C11), 113.4 (C14), 112.0 (C3). **IR**: ṽ (cm^{-1}) = 3397*w*, 3277*w*, 2222*w*, 1969*vw*, 1708*m*, 1685*m*, 1613*s*, 1596*s*, 1571*s*, 1484*s*, 1464*s*, 1402*m*, 1402*m*, 1311*s*, 1296*s*, 1240*m*, 1198*s*, 1155*m*, 1142*m*, 1120*m*, 1100*m*, 1068*m*, 1005*m*, 983*m*, 924*m*, 872*m*, 798*m*, 777*m*, 752*s*, 712*s*, 660*m*, 638*s*, 588*m*, 549*m*, 525*m*, 492*m*, 462*m*; **mp**: 164 – 167 °C; **HR-MS** (APPI$^+$, *m/z*): $[MH]^+$ calculated for $[C_{16}H_{10}ClN_2O]^+$: 281.0476, found 281.0500; **R$_f$** (SiO_2, *i*Hex : EtOAc 5 : 1) = 0.26.

(*E*/*Z*)-2-((4-(dimethylamino)phenyl)(mesityl)methylene)indolin-3-one (*E*/*Z*-1a)

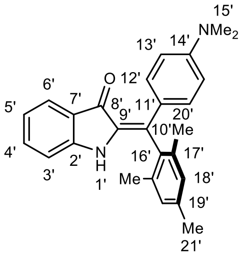

E-**9** (0.20 g, 0.67 mmol, 1.0 equiv.) was dissolved in 1,4-dioxane (0.67 M, 1.0 mL). Subsequently, (4-(dimethylamino)phenyl)boronic acid (0.17 g, 1.0 mmol, 1.5 equiv.), Cs_2CO_3 (0.44 g, 1.3 mmol, 2.0 equiv.) and SPhos Pd G2 (24 mg, 0.34 mmol) were added. The resulting red reaction mixture was stirred for 8.5 h at 80 °C. The reaction progress was monitored by thin-layer chromatography (SiO_2, *i*Hex : EtOAc 7 : 1). To the reaction mixture a solution of saturated $NaHCO_{3aq.}$ (5 mL) was added and the aqueous phase was extracted with CH_2Cl_2 (3 × 50 mL). The combined organic phases were dried over anhydrous Na_2SO_4 and the volatiles were removed *in vacuo*. The crude product was dissolved in CH_2Cl_2, adsorbed on Celite, purified two times by column chromatography (SiO_2, iHex : EtOAc + 1% Et_3N 100 : 0 → 88 : 12) and recrystallized from CH_2Cl_2/heptane to obtain *E*/*Z*-**1a** as red fine powdered crystals (107 mg, 0.27 mmol, 80%).

***E* isomer**: **^{1}H NMR** (601 MHz, CD_2Cl_2, 25 °C) δ (ppm) = 7.53 (ddt, $^3J_{H\text{-}H}$ = 7.7 Hz, $^4J_{H\text{-}H}$ = 1.4 Hz, $^4J_{H\text{-}H}$ = 0.7 Hz, 1H, H6), 7.42 (ddd, $^3J_{H\text{-}H}$ = 8.3 Hz, $^3J_{H\text{-}H}$ = 7.2 Hz, $^4J_{H\text{-}H}$ = 1.3 Hz, 1H, H4), 7.31 (m, 2H, H12), 7.09 (bs, 1H, H1), 6.93 (ds, $^4J_{H\text{-}H}$ = 0.7 Hz, 2H, H18), 6.87 (ddd, $^3J_{H\text{-}H}$ = 7.8 Hz, $^3J_{H\text{-}H}$ = 7.2 Hz, $^4J_{H\text{-}H}$ = 0.8 Hz, 1H, H5), 6.71 (m, 2H, H13), 2.99 (s, 6H, H15), 2.34 (s, 3H, H21), 2.02 (s, 6H, H20); **^{13}C NMR** (151 MHz, CD_2Cl_2, 25 °C) δ (ppm) = 184.5 (C8), 152.2 (C2), 150.6 (C14), 136.8 (C19), 136.3 (2C, C17), 135.9 (C16), 135.2 (C4), 132.0 (C9), 130.9 (2C, C12), 128.7 (2C, C18), 128.4 (C20), 125.4 (C11), 124.5 (C6), 123.1 (C7), 119.6 (C5), 112.5 (2C, C13), 111.8 (C3), 40.3 (2C, C15), 21.2 (1C, C21), 20.0 (2C, C20); ***Z* isomer**: **^{1}H NMR** (601 MHz, CD_2Cl_2, 25 °C) δ (ppm) = 7.6 (ddt, $^3J_{H\text{-}H}$ = 7.7 Hz, $^4J_{H\text{-}H}$ = 1.4 Hz, $^5J_{H\text{-}H}$ = 0.7 Hz, 1H, H6'), 7.47 (m, 2H, H12'), 7.35 (ddd, $^3J_{H\text{-}H}$ = 8.3 Hz, $^3J_{H\text{-}H}$ = 7.1 Hz, $^4J_{H\text{-}H}$ = 1.3 Hz, 1H, H4'), 6.98 (d, $^4J_{H\text{-}H}$ = 0.8 Hz, 2H, H18'), 6.83 (ddd, $^3J_{H\text{-}H}$ = 7.8 Hz, $^3J_{H\text{-}H}$ = 7.1 Hz, $^4J_{H\text{-}H}$ = 0.9 Hz, 1H, H5'), 6.75 (dt, $^3J_{H\text{-}H}$ = 8.1 Hz, $^4J_{H\text{-}H}$ = 0.8 Hz, 1H, H3'), 6.61 (m, 2H, H13'), 6.00 (bs, 1H, H1'), 2.99 (s, 6H, H15'), 2.34 (s, 3H, H21'), 2.07 (s, 6H, H20'); **^{13}C NMR** (151 MHz, CD_2Cl_2, 25 °C) δ (ppm) = 183.0 (C8'), 151.3 (C14'), 150.5 (C2'), 138.1 (C19'), 136.4 (2C,

C17'), 136.3 (C16'), 135.2 (C4'), 132.5 (C10'), 131.8 (C9'), 131.6 (2C, C12'), 129.1 (2C, C18'), 124.5 (C6'), 123.7 (C7'), 123.3 (C11'), 119.1 (C5'), 111.2 (C3'), 111.1 (2C, C13'), 40.3 (2C, C15'), 21.2 (C21'), 19.8 (2C, C20'); **IR**: ṽ (cm^{-1}) = 3211*m*, 3083*vw*, 3043*vw*, 2912*w*, 2849*w*, 1797*w*, 2361*s*, 2337*s*, 2209*vw*, 2145*w*, 2110*w*, 2065*w*, 1793*vw*, 1734*w*, 1699*vw*, 1664*s*, 1603*vs*, 1559*vs*, 1544*s*, 1520*s*, 1485*s*, 1466*s*, 1441*m*, 1395*m*, 1365*m*, 1317*s*, 1290*m*, 1263*m*, 1196*vs*, 1166*s*, 1138*s*, 1096*s*, 1062*w*, 1011*w*, 983*m*, 958*m*, 942*m*, 911*m*, 879*w*, 843*m*, 816*s*, 751*s*, 735*m*, 711*m*, 667*m*, 625*w*, 596*m*, 575*w*, 556*m*, 512*m*, 490*w*, 435*m*, 417*vw*; **mp**: 242 – 243 °C; **HR-MS** ($APPI^+$, *m/z*): $[MH]^+$ calculated for $[C_{26}H_{27}N_2O]+$: 383.2118, found 383.2114; $\mathbf{R_f}$ (SiO_2, *i*Hex : EtOAc 10 : 1) = 0.13.

(*E*/*Z*)-2-((3,5-bis(trifluoromethyl)phenyl)(4-(dimethylamino)phenyl)-methylene)indolin-3-one (*E*/*Z*-2a)

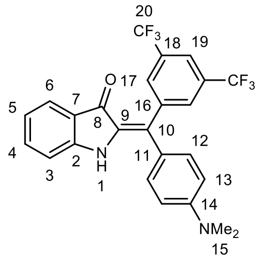

E-**10** (0.80 g, 2.0 mmol, 1.0 equiv.) was dissolved in 1,4-dioxane (0.5 M, 2.0 mL). Subsequently, (4-(Dimethylamino)phenyl)boronic acid (0.51 g, 3.1 mmol, 1.5 equiv.), CsF (0.62 g, 4.1 mmol, 2.0 equiv.), John Phos (0.12 g, 0.41 mmol) and $Pd(OAc)_2$ (46 mg, 0.2 mmol) were added. The resulting red reaction mixture was stirred for 21 h at 80 °C. The reaction progress was monitored by thin-layer chromatography (SiO_2, *i*Hex : EtOAc 3 : 1). To the reaction mixture a solution of saturated $NaHCO_{3aq}$ (5 mL) was added and the aqueous phase was extracted with EtOAc (3 × 50 mL). The combined organic phases were dried over anhydrous Na_2SO_4 and the volatiles were removed *in vacuo*. The crude product was dissolved in EtOAc, adsorbed on Celite, purified by column chromatography (SiO_2, *i*Hex : EtOAc 84 : 16 → 75 : 25), adsorbed on Al_2O_3 basic Brockmann I and purified by reversed-phase column chromatography (SiO_2 C18, acetonitrile : H_2O 0 : 100 → 100 : 0) to obtain *E*/*Z*-**2a** as a red solid (0.48 g, 0.98 mmol, 49%).

***E* isomer:** **^{1}H NMR** (601 MHz, CD_2Cl_2, 25 °C) δ (ppm) = 7.88 – 7.86 (bs, 1H, H19), 7.81 – 7.78 (bs, 2H, H17), 7.58 (d, $^3J_{H-H}$ = 7.6 Hz, 1H, H6), 7.44 (ddd, $^3J_{H-H}$ = 8.4, $^3J_{H-H}$ = 7.2 Hz, $^4J_{H-H}$ = 1.3 Hz, 1H, H4), 7.22 (m, 2H, H12), 6.93 (d, $^3J_{H-H}$ = 8.1, 1H, H3), 6.90 (ddd, $^3J_{H-H}$ = 7.9, $^3J_{H-H}$ = 7.2, $^4J_{H-H}$ 0.9 Hz, 1H, H5), 6.86 (bs, 1H, H1), 6.75 (m, 2H, H13), 3.02 (s, 6H, H15); **^{13}C NMR** (151 MHz, CD_2Cl_2, 25 °C) δ (ppm) = 184.5 (C8), 151.9 (C2), 151.0 (C14), 141.4 (C9), 136.0 (C4), 131,8 (C12), 131.7 (C17 or C17′), 131.7 (C17 or C17′), 131.3 (C18), 127.1 (C10), 126.7 (C16), 125.6 (C11), 124.9 (C6), 123.1 (C20), 123.0 (C7), 121.8 (p, $^4J_{C-F}$ = 3.9 Hz, C19), 120.1 (C5), 112.5 (C13), 111.7 (C3), 40.3 (C15);

Z isomer: **^{1}H NMR** (601 MHz, CD_2Cl_2, 25 °C) δ (ppm) = 7.91 (bs, 1H, H19‘), 7.88 – 7.86 (bs, 2H, H17‘), 7.63 (d, $^3J_{H-H}$ = 7.7, 1H, H6‘), 7.46 – 7.41 (m, 1H, H4‘), 7.19 (m, 2H, H12‘), 6.95 – 6.87 (m, 2H, H5‘, H3‘), 6.66 (m, 2H, H13‘), 6.26 (bs, 1H, H1‘), 3.02 (s, 6H, H15‘); **^{13}C NMR** (151 MHz, CD_2Cl_2, 25 °C) δ (ppm) = 184.0 (C8‘), 151.5 (C14‘), 151.2 (C2‘), 143.6 (C9‘), 135.8 (C4‘), 132.7 (C12‘), 131.3 (C17‘), 129.0 (C10‘), 124.8 (C6‘), 123.7 (C11‘), 122.3 (C19‘), 120.2

(C5‘), 111.8 (C3‘), 111.4 (C13‘), 40.3 (C15‘), C7‘, C16‘, C18‘, C20‘ not assigned; **IR**: ṽ (cm^{-1}) = 3263*w*, 2979*w*, 1663*m*, 1622*m*, 1603*s*, 1557*m*, 1522*m*, 1486*m*, 1467*m*, 1444*w*, 1364*s*, 1311*m*, 1274*s*, 1168*s*, 1123*s*, 1017*w*, 1001*m*, 965*m*, 945*w*, 881*m*, 896*m*, 845*m*, 820*m*, 750*s*, 733*w*, 713*w*, 703*w*, 682*s*, 657*w*, 571*m*, 546*w*, 530*w*, 506*w*, 459*w*; **mp**: 206 – 208 °C; **HR-MS** ($APPI^+$, *m*/*z*): $[MH]^+$ calculated for $[C_{25}H_{19}F_6N_2O]^+$: 477.1396, found 477.1408; $\mathbf{R_f}$ (SiO_2, *i*Hex : EtOAc 3 : 1) = 0.32.

(*E*/*Z*)-2-((3,5-dimethoxyphenyl)(4-(dimethylamino)phenyl)methylene)-indolin-3-one (*E*/*Z*-3a)

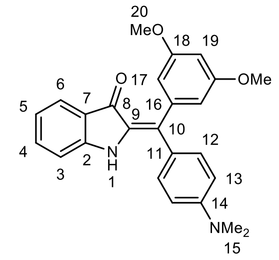

E-**11** (100 mg, 0.33 mmol, 1.0 equiv.) was dissolved in 1,4-dioxane (0.18 M, 1.9 mL). Subsequently, (3,5-dimethoxyphenyl)boronic acid (91 mg, 0.50 mmol, 1.5 equiv.), CsF (102 mg, 0.67 mmol, 2.0 equiv.), and Pd(dppf)Cl_2 (19 mg, 0.033 mmol) were added. The resulting red reaction mixture was stirred for 48 h at 80 °C. The reaction progress was monitored by thin-layer chromatography (SiO_2, *i*Hex : EtOAc 3 : 1). To the reaction mixture a solution of saturated $NaHCO_{3\ aq.}$ (5 mL) was added and the aqueous phase was extracted with CH_2Cl_2 (3 × 50 mL). The combined organic phases were dried over anhydrous Na_2SO_4 and the volatiles were removed *in vacuo*. The crude product was dissolved in CH_2Cl_2, adsorbed on Al_2O_3, purified two times by column chromatography (ALOX N, *i*Hex : EtOAc 100 : 0 → 80 : 20) and recrystallized from MeOH/H_2O to obtain *E*/*Z*-**3a** as red fine powdered crystals (107 mg, 0.27 mmol, 80%).

***E* isomer: ^{1}H NMR** (601 MHz, CD_2Cl_2, 25 °C) δ (ppm) = 7.60 (d, $^3J_{H\text{-}H}$ = 7.7 Hz, 1H, H6), 7.38c (ddd, $^3J_{H\text{-}H}$ = 8.2 Hz, 7.2 Hz, $^4J_{H\text{-}H}$ = 1.4 Hz, 1H, H4), 7.27 (m, 2H, H12), 6.91 (d, $^3J_{H\text{-}H}$ = 8.2 Hz, 1H, H3), 6.85 (dt, $^3J_{H\text{-}H}$ = 7.6, $^4J_{H\text{-}H}$ = 0.8 Hz, 1H, H5), 6.72 (m, 3H, H1, H13), 6.49-6.46 (m, 1H, H19), 6.43 (d, $^4J_{H\text{-}H}$ = 2.3 Hz, 2H, H17), 3.75 (s, 6H, H20), 3.01 (s, 6H, H15); **^{13}C NMR** (151 MHz, CD_2Cl_2, 25 °C) δ (ppm) = 183.5 (C8), 160.5 (C18), 150.5 (C2), 150.4 (C14), 140.9 (C9), 135.97 or 134.96 (C4), 131.6 (C16), 131.5 (C12), 130.8 (C10), 126.5 (C22), 124.2 (C6), 123.1 (C7), 118.9 (C5), 111.9 (C11), 111.2 (C3), 108.8 (C17), 99.8 (C19), 55.3 (C20), 40.0 (C15); ***Z* isomer: ^{1}H NMR** (601 MHz, CD_2Cl_2, 25 °C) δ (ppm) = 7.58 (d, $^3J_{H\text{-}H}$ = 7.7 Hz, 1H, H6’), 7.41 (ddd, $^3J_{H\text{-}H}$ = 8.2 Hz, 7.2 Hz, $^4J_{H\text{-}H}$ = 1.36 Hz, 1H, H4’), 7.29 (m, H12’), 6.87 (dt, $^3J_{H\text{-}H}$ = 7.5 Hz, $^4J_{H\text{-}H}$ = 0.9 Hz, 1H, H5’), 6.83 (d, $^3J_{H\text{-}H}$ = 7.2 Hz, 1H, H3’), 6.64 (m, 2H, H13’), 6.49-6.46 (m, 1H, H19’), 6.48 (d, $^4J_{H\text{-}H}$ = 2.1 Hz, 2H, H17’), 3.77 (s, 6H, H20’), 3.01 (s, 6H, H15’); ^{13}C NMR (151 MHz, CD_2Cl_2, 25 °C) δ (ppm) = 183.8 (C8’), 161.1 (C18’), 151.3 (C2’), 150.9 (C14’), 142.7 (C9’), 135.97 or 134.96 (C4’), 132.6 (C10’), 132.2 (C12’), 131.8 (C16’), 124.4 (C13’), 124.3 (C6’), 122.9 (C7’), 119.2 (C5’), 111.0 (C3’), 110.8 (C11’), 108.4 (C17’), 100.1 (C19’), 55.4 (C20’), 40.0 (C15’); **IR**: ṽ (cm^{-1}) = 3258*w*, 2923*w*, 1662*m*, 1601*s*, 1556*s*,

1519*s*, 1486*s*, 1465*s*, 1421*m*, 1334*s*, 1317*s*, 1256*m*, 1191*s*, 1149*s*, 1135*s*, 1096*s*, 1058*s*, 1001*s*, 965*s*, 941*s*, 885*s*, 841*s*, 817*s*, 747*s*, 715*s*, 700*s*, 685*s*, 660*s*, 569*s*, 511*s*; **mp**: 157 – 161 °C; **HR-MS** (APPI$^+$, *m*/*z*): [MH]$^+$ calculated for $[C_{25}H_{25}N_2O_3]^+$: 401.1860, found 401.1865; $\mathbf{R_f}$ (SiO_2, *i*Hex : EtOAc 3 : 1) = 0.24.

(*E*/*Z*)-4-((4-(dimethylamino)phenyl)(3-oxoindolin-2-ylidene)methyl)-benzonitrile (*E*/*Z*-4a)

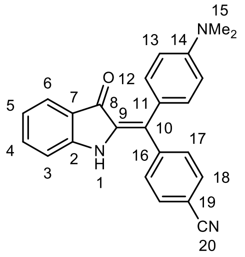

E/*Z*-**12** (0.25 g, 0.89 mmol, 1.0 equiv.) was dissolved in 1,4-dioxane (0.5 M, 1.8 mL). Subsequently, (4-(dimethylamino)phenyl)boronic acid (0.22 g, 1.3 mmol, 1.5 equiv.), CsF (0.28 g, 1.8 mmol, 2.0 equiv.), John Phos (53 mg, 0.18 mmol), and $Pd(OAc)_2$ (20 mg, 0.089 mmol) were added. The resulting red reaction mixture was stirred for 48 h at 80 °C. The reaction progress was monitored by thin-layer chromatography (SiO_2, *i*Hex : EtOAc 3 : 1). To the reaction mixture a solution of saturated $NaHCO_{3\ aq.}$ (5 mL) was added and the aqueous phase was extracted with CH_2Cl_2 (3 × 50 mL). The combined organic phases were dried over anhydrous Na_2SO_4 and the volatiles were removed *in vacuo*. The crude product was dissolved in CH_2Cl_2, adsorbed on Celite or Al_2O_3, and purified two times by column chromatography (SiO_2, *i*Hex : EtOAc 100 : 0 → 0 : 100 and Al_2O_3, *i*Hex : EtOAc 100 : 0 → 70 : 30), followed by a recrystallization from MeOH/H_2O to obtain *E*/*Z*-**4a** as red fine powdered crystals (101 mg, 0.28 mmol, 31%).

***E* isomer:** ^{1}**H NMR** (601 MHz, CD_2Cl_2, 25 °C) δ (ppm) = 7.72 (m, 2H, H18), 7.61 (d, $^{3}J_{H\text{-}H}$ = 7.8 Hz, 1H, H6), 7.50 (m, 2H, H17), 7.45 – 7.40 (m, 1H, H4), 7.22 – 7.17 (m, 2H, H12), 6.91 – 6.88 (m, 1H, H5), 6.86 (d, $^{3}J_{H\text{-}H}$ = 8.2 Hz, 1H, H3), 6.65 (m, 2H, H13), 6.37 (bs, 1H, H1), 3.01 (s, 6H, H15); ^{13}**C NMR** (151 MHz, CD_2Cl_2, 25 °C) δ (ppm) = 184.1 (C8), 151.4 (C14), 151.2 (C2), 135.8 (C4), 132.9 (C18), 132.7 (C12), 131.8 (C17), 130.2 (C10), 124.8 (C6), 124.3 (C11), 123.2 (C7), 120.0 (C5), 119.0 (C20), 112.1 (C19), 111.6 (C3), 111.4 (C13), 40.4 (C15), C9 and C16 not found; ***Z* isomer:** ^{1}**H NMR** (601 MHz, CD_2Cl_2, 25 °C) δ (ppm) = 7.64 (m, 2H, H18'), 7.56 (d, $^{3}J_{H\text{-}H}$ = 7.8 Hz, 1H, H6'), 7.43 (ddd, $^{3}J_{H\text{-}H}$ = 8.4 Hz, $^{3}J_{H\text{-}H}$ = 7.2 Hz, $^{4}J_{H\text{-}H}$ = 1.3 Hz. 1H, H4'), 7.41 (m, 2H, H17'), 7.20 (m, 2H, H12'), 6.92 (d, $^{3}J_{H\text{-}H}$ = 8.2 Hz, 1H, H3'), 6.89 (dd, $^{3}J_{H\text{-}H}$ = 7.4 Hz, $^{4}J_{H\text{-}H}$ = 0.8 Hz, 1H, H5'), 6.81 (bs, 1H, H1'), 6.73 (m, 2H, H13'), 3.01 (s, 6H, H15'); ^{13}**C NMR** (151 MHz, CD_2Cl_2, 25 °C) δ (ppm) = 184.5 (C8'), 151.9 (C2'), 150.9 (C14'), 144.4 (C16'), 135.9 (C4'), 132.6 (C9'), 132.1 (C18'), 132.0 (C17'), 131.9 (C12'), 128.9 (C10'), 126.1 (C11'), 124.8 (C6'), 123.1 (C7'), 120.1 (C5'), 119.5 (C20'), 112.4 (C13'), 111.8 (C3'), 111.6 (C19'), 40.4 (C15'); **IR**: ṽ (cm^{-1}) = 2224*w*, 1667*w*, 1602*m*, 1557*w*, 1521*w*, 1484*w*,

1465*w*, 1363*m*, 1319*m*, 1060*s*, 982*s*, 926*s*, 818*m*, 150*m*, 708*m*, 572*m*, 432*s*; **mp**: > 400 °C; **HR-MS** (APPI$^+$, *m*/*z*): [MH]$^+$ calculated for [$C_{24}H_{20}N_3O$]$^+$: 366.1601, found 366.1608; **R$_f$** (SiO_2, *i*Hex : EtOAc 3 : 1) = 0.25.

(E/Z)-2-((4-(dimethylamino)phenyl)(mesityl)methylene)-1-propylindolin-3-one (E/Z-1b)

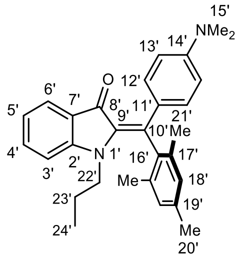

NaH (16 mg, 0.39 mmol, 60 %m/m suspension in paraffin oil, 1.5 equiv.) was added to a solution of *E*/*Z*-**1a** (100 mg, 0.26 mmol, 1.0 equiv.) in DMF (0.13 M, 2.0 mL). The reaction mixture was stirred for 15 min at 23 °C while the color changed from red to blue. Then, 1-iodopropane (31 µL, 53 mg, 0.31 mmol, 1.75 g mL^{-1}, 1.2 equiv.) was added dropwise, while the color turned red again. The reaction progress was monitored by thin-layer chromatography (SiO_2, *i*Hex : EtOAc 7 : 1). After 2 h the reaction was stopped by adding a saturated $NH_4Cl_{aq.}$ solution (10 mL). The aqueous phase was extracted with EtOAc (3 × 50 mL) and the combined organic phases were washed with a saturated $NaHCO_{3\ aq.}$ solution (3 × 100 mL), H_2O (3 × 100 mL) and a saturated $NaCl_{aq.}$ (3 × 100 mL) solution. The organic phase was dried over anhydrous Na_2SO_4 and the volatiles were removed *in vacuo*. The crude product was dissolved in CH_2Cl_2, adsorbed on Celite, purified two times by column chromatography (SiO_2, iHex : EtOAc 100 : 0 → 88 : 12 and SiO_2, iHex : EtOAc 100:0 → 92 : 8) and recrystallized from MeOH/H_2O to obtain *E*/*Z*-**1b** as a red solid (111 mg, 0.26 mmol, quant.).

***E* isomer: ^{1}H NMR** (601 MHz, CD_2Cl_2, 25 °C) δ (ppm) = 7.51 (ddd, $^3J_{H\text{-}H}$ = 7.6 Hz, $^4J_{H\text{-}H}$ = 1.4 Hz, $^5J_{H\text{-}H}$ = 0.7 Hz, 1H, H6), 7.46 (ddd, $^3J_{H\text{-}H}$ = 8.5 Hz, $^3J_{H\text{-}H}$ = 7.2 Hz, $^4J_{H\text{-}H}$ = 1.4 Hz, 1H, H4), 7.17 (m, 2H, H12), 7.08 (d, $^3J_{H\text{-}H}$ = 8.3 Hz, 1H, H3), 6.92 (bs, 2H, H18), 6.85 – 6.79 (m, 1H, H5), 6.65 (m, 2H, H13), 3.61 (t, $^3J_{H\text{-}H}$ = 7.2 Hz, 2H, H22), 3.10 – 2.87 (m, 6H, H15), 2.33 (s, 3H, H20), 2.00 (s, 6H, H21), 1.40 (sx, $^3J_{H\text{-}H}$ = 7.1 Hz, 2H, H23), 0.60 (t, $^3J_{H\text{-}H}$ = 7.4 Hz, 3H, H24); **^{13}C NMR** (151 MHz, CD_2Cl_2, 25 °C) δ (ppm) = 185.8 (C8), 155.5 (C2), 150.5 (C14), 125.7 (C11), 124.3 (C6), 137.3 (C16), 135.0 (C4), 132.4 (C12), 131.4 (C10), 128.9 (C18), 123.8 (C7) 119.4 (C5), 111.8 (C3), 111.6 (C13), 48.0 (C22), 40.3 (C15), 21.3 (C20), 20.7 (C23), 20.4 (C21), 11.5 (C24), C9, C17, C19 not assumed;
***Z* isomer: ^{1}H NMR** (601 MHz, CD_2Cl_2, 25 °C) δ (ppm) = 7.56 (ddd, $^3J_{H\text{-}H}$ = 7.6, $^4J_{H\text{-}H}$ = 1.4, $^5J_{H\text{-}H}$ = 0.7 Hz, 1H, H6’), 7.41 (ddd, $^3J_{H\text{-}H}$ = 8.4 Hz, $^3J_{H\text{-}H}$ = 7.1 Hz, $^4J_{H\text{-}H}$ = 1.4 Hz, 1H, H4’), 7.14 (m, 2H, H12’), 6.93 (bs, 2H, H18’), 6.89 (d, $^3J_{H\text{-}H}$ = 12.1 Hz, 1H, H3’), 6.85 (dt, $^3J_{H\text{-}H}$ =

11.1 Hz, $^{4}J_{H\text{-}H}$ = 0.9 Hz, 1H, H5'), 6.60 (m, 2H, H13'), 3.10 – 2.87 (m, 8H, H15, H22'), 2.31 (s, 3H, H20'), 1.99 (s, 6H, H21'), 1.33 – 1.24 (m, 3H, H23'), 0.56 (t, $^{3}J_{H\text{-}H}$ = 7.4 Hz, 3H, H24'); **^{13}C NMR** (151 MHz, CD_2Cl_2, 25 °C) δ (ppm) = 184.0 (C8'), 153.7 (C2'), 151.3 (C14'), 124.4 (C6'), 138.2 (C16'), 135.1 (C4'), 133.9 (C10'), 132.8 (C12'), 129.1 (C18'), 123.3 (C7'), 119.1 (C5'), 111.1 (C13'), 110.4 (C3'), 47.5 (C22'), 40.4 (C15'), 21.2 (C20'), 21.0 (C23'), 20.4 (C21'), 11.1 (C24'), C11, C9, C17, C19 not assumed; **IR**: ṽ (cm^{-1}) = 3266*w*, 2920*m*, 2850*m*, 2176*vw*, 1992*vw*, 1664*m*, 1599*s*, 1554*m*, 1518*s*, 1646*s*, 1421*m*, 1347*s*, 1316*s*, 1256*m*, 1227*s*, 1185*s*, 1156*s*, 1126*s*, 1095*s*, 1057*s*, 1000*m*, 964*m*, 943*m*, 922*m*, 885*m*, 849*s*, 815*s*, 747*s*, 715*m*, 675*s*, 569*m*, 553*m*, 511*m*, 450*m*, 424*m*; **mp**: 94 – 96 °C; **HR-MS** ($APPI^+$, *m*/*z*): $[MH]^+$ calculated for $[C_{29}H_{33}N_2O]^+$: 425.2587, found 425.2595; **R_f** (SiO_2, *i*Hex : EtOAc 7 : 1) = 0.32.

(*E*/*Z*)-2-((3,5-bis(trifluoromethyl)phenyl)(4-(dimethylamino)phenyl)-methylene)-1-propylindolin-3-one (*E*/*Z*-2b)

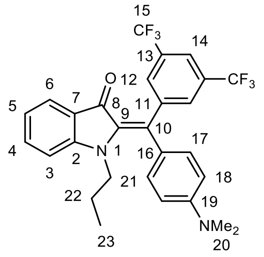

NaH (11 mg, 0.28 mmol, 60 %m/m suspension in paraffin oil, 1.5 equiv.) was added to a solution of *E*/*Z*-**2a** (90 mg, 0.19 mmol, 1.0 equiv.) in DMF (0.13 M, 1.5 mL). The reaction mixture was stirred for 15 min at 23 °C while the color changed from red to blue. Then, 1-iodopropane (22 µL, 39 mg, 0.23 mmol, 1.75 g mL^{-1}, 1.2 equiv.) was added dropwise, while the color turned red again. The reaction progress was monitored by thin-layer chromatography (SiO_2, *i*Hex : EtOAc 5 : 1). After 2 h 20 min the reaction was stopped by adding a saturated $NH_4Cl_{aq.}$ solution (10 mL). The aqueous phase was extracted with EtOAc (3 × 50 mL) and the combined organic phases were washed with a saturated $NaHCO_{3\,aq.}$ solution (3 × 100 mL), H_2O (3 × 100 mL) and a saturated $NaCl_{aq.}$ (3 × 100 mL) solution. The organic phase was dried over anhydrous Na_2SO_4 and the volatiles were removed *in vacuo*. The crude product was recrystallized from MeOH/H_2O, purified by reversed-phase column chromatography (SiO_2 C_{18}, MeOH : H_2O 0 : 100 → 100 : 0) and again recrystallized from MeOH/H_2O to obtain *E*/*Z*-**2b** as red fine powdered crystals (63 mg, 0.12 mmol, 64%).

***E* isomer: ^{1}H NMR** (601 MHz, CD_2Cl_2, 25 °C) δ (ppm) = 7.88 (s, 1H, H14), 7.70 (d, $^4J_{H\text{-}H}$ = 1.7 Hz, 2H, H12), 7.55 (d, $^3J_{H\text{-}H}$ = 7.5 Hz, 1H, H6), 7.49 (t, $^3J_{H\text{-}H}$ = 7.7 Hz, 1H, H4), 7.08 (m, 2H, H17), 7.04 (d, $^3J_{H\text{-}H}$ = 8.3 Hz, 1H, H3), 6.90 (t, $^3J_{H\text{-}H}$ = 7.4 Hz, 1H, H5), 6.71 (m, 2H, H18), 3.41 (t, $^3J_{H\text{-}H}$ = 7.3 Hz, 2H, H21), 3.03 (s, 6H, H20), 1.41 (q, $^3J_{H\text{-}H}$ = 7.4 Hz, 2H, H22), 0.62 (t, $^3J_{H\text{-}H}$ = 7.4 Hz, 3H, H23); **^{13}C NMR** (151 MHz, CD_2Cl_2, 25 °C) δ (ppm) = 185.8 (C8), 155.1 (C2), 151.1 (C19), 143.4 (C11), 136.0 (C10), 135.7 (C4), 132.9 (2C, C17), 132.2 (d, $^3J_{C\text{-}F}$ = 3.10 Hz, 2C, C12), 131.2 (q, $^1J_{C\text{-}F}$ = 33.0 Hz, 2C, C13), 130.1 (C10), 126.1 (C16), 124.7 (C6), 124.0 (q, $^1J_{C\text{-}F}$ = 272.8 Hz, 2C, C15), 123.4 (C7), 121.9 (p, $^3J_{C\text{-}F}$ = 3.9 Hz, 1H, H14), 120.0 (C5), 111.9 (2C, C18), 111.5 (C3), 47.0 (C21), 40.3 (2C, C20), 20.7 (C22), 11.5 (C23); ***Z* isomer: ^{1}H NMR** (601 MHz, CD_2Cl_2, 25 °C) δ (ppm) = 7.89 (s, 1H, H14'), 7.78 (d, $^4J_{H\text{-}H}$ = 1.7 Hz, 2H, H12'), 7.60 (d, $^3J_{H\text{-}H}$ = 7.5 Hz, 1H, H6'), 7.51–7.46 (m, 1H, H4'), 7.10 (m, 2H, H17'), 7.00 (d,

$^3J_{H\text{-}H}$ = 8.2 Hz, 1H, H3'), 6.94 (t, *J* = 7.4 Hz, 1H, H5'), 6.68 (m, 2H, H18'), 3.07 (t, $^3J_{H\text{-}H}$ = 7.3 Hz, 2H, H21'), 3.05 (s, 6H, H20'), 0.88 (t, $^3J_{H\text{-}H}$ = 6.9 Hz, 2H, H22'), 0.5 (t, $^3J_{H\text{-}H}$ = 7.4 Hz, 3H, H23'); **^{13}C NMR** (151 MHz, CD_2Cl_2, 25 °C) δ (ppm) = 185.0 (C8'), 154.2 (C2'), 151.9 (C19'), 144.6 (C11'), 136.6 (C10'), 135.4 (C4'), 133.7 (2C, C17'), 131.9 (q, $^2J_{C\text{-}F}$ = 33.0 Hz, 2C, C13'), 131.8 (s, 2C, C12'), 130.6 (C10'), 125.7 (C16'), 124.6 (C6'), 123.7 (q, $^1J_{C\text{-}F}$ = 272.8 Hz, 2C, C15'), 123.4 (C7'), 122.1 (p, $^3J_{C\text{-}F}$ = 3.9 Hz, C14'), 120.2 (C5'), 111.6 (2C, C18'), 111.5 (C3'), 47.8 (C21'), 40.4 (2C, C20'), 20.6 (C22'), 11.1 (C23'); **IR**: ṽ (cm^{-1}) = 2920*w*, 2849*w*, 1673*s*, 1600*s*, 1556*s*, 1522*s*, 1477*s*, 1464*s*, 1441*m*, 1384*m*, 1366*s*, 1317*s*, 1274*s*, 1167*s*, 1124*s*, 1096*s*, 1064*s*, 1014*s*, 965*s*, 948*s*, 908*s*, 891*s*, 868*s*, 846*s*, 835*s* 817*s*, 780*s*, 750*s*, 730*s*, 714*s*, 702*s*, 683*s*, 671*s*, 626*w*, 584*s*, 570*s*, 546*s*, 519*w*, 507*w*, 473*w*, 429*w*; **mp**: 111 – 113 °C; **HR-MS** (APPI$^+$, *m*/*z*): $[MH]^+$ calculated for $[C_{28}H_{25}F_6N_2O]^+$: 519.1866, found 519.1879; **R$_f$** (SiO_2, *i*Hex : EtOAc 5 : 1) = 0.43.

(*E*/*Z*)-2-((3,5-dimethoxyphenyl)(4(dimethylamino)phenyl)methylene)-indolin-3-one (*E*/*Z*-3b)

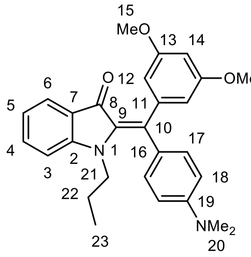

NaH (13 mg, 0.34 mmol, 60 %m/m suspension in paraffin oil, 1.5 equiv.) was added to a solution of *E*/*Z*-**3a** (90 mg, 0.22 mmol, 1.0 equiv.) in DMF (0.13 M, 2.0 mL). The reaction mixture was stirred for 15 min at 23 °C while the color changed from red to blue. Then, 1-iodopropane (26 µL, 46 mg, 0.27 mmol, 1.75 g mL^{-1}, 1.2 equiv.) was added dropwise, while the color turned red again. The reaction progress was monitored by thin-layer chromatography (SiO_2, *i*Hex : EtOAc 5 : 1). After 2 h the reaction was stopped by adding a saturated $NH_4Cl_{aq.}$ solution (10 mL). The aqueous phase was extracted with EtOAc (3 × 50 mL) and the combined organic phases were washed with a saturated $NaHCO_{3\ aq.}$ solution (3 × 100 mL), H_2O (3 × 100 mL) and a saturated $NaCl_{aq.}$ (3 × 100 mL) solution. The organic phase was dried over anhydrous Na_2SO_4 and the volatiles were removed *in vacuo*. The crude product was recrystallized from MeOH/H_2O, purified by column chromatography (SiO_2, *i*Hex : EtOAc 100 : 0 → 70 : 30) to obtain *E*/*Z*-**3b** as a red solid (60 mg, 0.14 mmol, 61%).

***E* isomer:** **^{1}H NMR** (601 MHz, CD_2Cl_2, 25 °C) δ (ppm) = 7.58 – 7.54 (m, 1H, H6), 7.45 (ddd, $^3J_{H-H}$ = 8.4 Hz, $^3J_{H-H}$ = 7.7 Hz, $^4J_{H-H}$ = 1.4 Hz, H4), 7.12 (m, 2H, H17), 7.03 (d, $^3J_{H-H}$ = 8.3 Hz, 1H, H3), 6.87 (dt, $^3J_{H-H}$ = 7.5 Hz, $^4J_{H-H}$ = 0.8 Hz, 1H, H5), 6.68 (m, 2H, H18), 6.50 (t, $^4J_{H-H}$ = 2.3 Hz, 1H, H14), 6.38 (d, $^4J_{H-H}$ = 2.3 Hz, 2H, H12), 3.75 (s, 6H, H15), 3.37 (t, $^3J_{H-H}$ = 7.2 Hz, 2H, H21), 3.01 (s, 6H, H20), 1.42 – 1.34 (m, 4H, H22), 0.58 (t, $^3J_{H-H}$ = 7.4 Hz, 3H, H23); **^{13}C NMR** (151 MHz, CD_2Cl_2, 25 °C) δ (ppm) = 185.5 (C8), 160.8 (C13), 154.8 (C2), 150.9 (C19), 143.4 (C9), 135.5 (C11), 135.0 (C4), 133.0 (C17), 127.4 (C16), 124.4 (C6), 123.9 (C7), 119.5 (C5), 111.5 (C3), 111.3 (C18), 110.1 (C12), 100.3 (C14), 55.7 (C15), 47.1 (C21), 40.4 (C20), 20.6 (C22), 11.5 (C23); ***Z* isomer:** **^{1}H NMR** (601 MHz, CD_2Cl_2, 25 °C) δ (ppm) = 7.58 – 7.54 (m, 1H, H6'), 7.43 (ddd, $^3J_{H-H}$ = 8.4 Hz, $^3J_{H-H}$ = 7.7 Hz, $^4J_{H-H}$ = 1.4 Hz, H4'), 7.15 (m, 2H, H17'), 6.97 (d, $^3J_{H-H}$ = 8.3 Hz, 1H, H3'), 6.86 (dt, $^3J_{H-H}$ = 7.5 Hz, $^4J_{H-H}$ = 0.8 Hz, 1H, H5'), 6.64 (m, 2H, H18'), 6.48 (t, $^4J_{H-H}$ = 2.3 Hz, 1H, H14'), 6.42 (d, $^4J_{H-H}$ = 2.3 Hz, 2H, H12'), 3.74 (s,

6H, H15’), 3.17 (t, $^3J_{H\text{-}H}$ = 7.2 Hz, 2H, H21’), 3.02 (s, 6H, H20’), 1.42 – 1.34 (m, 4H, H22’), 0.59 (t, $^3J_{H\text{-}H}$ = 7.4 Hz, 3H, H23’); **^{13}C NMR** (151 MHz, CD_2Cl_2, 25 °C) δ (ppm) = 184.9 (C8’), 161.0 (C13’), 154.1 (C2’), 151.6 (C19’), 144.0 (C9’), 135.4 (C10’), 135.3 (C11’), 134.9 (C4’), 133.7 (C17’), 126.9 (C16’), 124.4 (C6’), 123.5 (C7’), 119.3 (C5’), 111.7 (C18’), 111.1 (C3’), 109.7 (C12’), 100.9 (C14’), 55.9 (C15’), 47.0 (C21’), 40.4 (C20’), 20.9 (C22’), 11.4 (C23’); **IR**: ṽ (cm^{-1}) = 2955*w*, 1664*s*, 1598*s*, 1517*s*, 1474*s*, 1452*s*, 1419*s*, 1361*m*, 1316*s*, 1229*s*, 1185*s*, 1151*s*, 1124*s*, 1096*s*, 1061*s*, 1045s, 1007*s*, 969*m*, 941*m*, 830*s*, 817*m*, 748*s*, 732*m*, 704*m*, 673*w*, 642*w*, 598*m*, 554*m*, 552*w*, 466*w*, 431*m*; **mp**: 186 – 187 °C; **HR-MS** (APPI$^+$, *m*/*z*): $[MH]^+$ calculated for $[C_{28}H_{31}N_2O_3]^+$: 443.2329, found 443.2338; **R_f** (SiO_2, *i*Hex : EtOAc 5 : 1) = 0.1.

(*E*/*Z*)-4-((4-(dimethylamino)phenyl)(3-oxo-1-propylindolin-2-ylidene)methyl)benzonitrile (*E*/*Z*-4b)

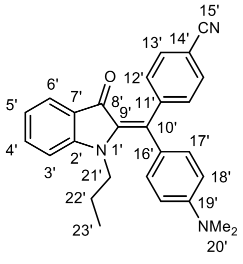

NaH (12 mg, 0.29 mmol, 60 %m/m suspension in paraffin oil, 1.5 equiv.) was added to a solution of *E*/*Z*-**4a** (70 mg, 0.19 mmol, 1.0 equiv.) in DMF (0.13 M, 1.5 mL). The reaction mixture was stirred for 15 min at 23 °C while the color changed from red to blue. Then, 1-iodopropane (22 µL, 39 mg, 0.23 mmol, 1.75 g mL^{-1}, 1.2 equiv.) was added dropwise, while the color of the mixture turned red again. The reaction progress was monitored by thin-layer chromatography (SiO_2, *i*Hex : EtOAc 3 : 1). After 30 min the reaction was stopped by adding a saturated $NH_4Cl_{aq.}$ solution (10 mL). The aqueous phase was extracted with EtOAc (3 × 50 mL) and the combined organic phases were washed with a saturated $NaHCO_{3\ aq.}$ solution (3 × 100 mL), H_2O (3 × 100 mL) and a saturated $NaCl_{aq.}$ (3 × 100 mL) solution. The organic phase was dried over anhydrous Na_2SO_4 and the volatiles were removed *in vacuo*. The crude product was dissolved in CH_2Cl_2, adsorbed on Celite, purified two times by column chromatography (SiO_2, *i*Hex : EtOAc 100 : 0 → 75 : 25) and recrystallized from MeOH/H_2O to obtain *E*/*Z*-**4b** as red fine powdered crystals (0.59 g, 0.15 mmol, 76%).

***E* isomer:** **^{1}H NMR** (601 MHz, CD_2Cl_2, 25 °C) δ (ppm) = 7.68 (m, 2H, H13), 7.58 (ddd, $^3J_{H\text{-}H}$ = 7.6, $^4J_{H\text{-}H}$ = 1.3, $^5J_{H\text{-}H}$ = 0.6 Hz, 1H, H6), 7.48 – 7.45 (m, *1*H, H4), 7.40 (m, 2H, H12), 7.09 (m, 2H, H17), 6.98 (d, $^3J_{H\text{-}H}$ = 8.2 Hz, 1H, H3), 6.93 – 6.88 (m, 1H, H5), 6.65 (m, 2H, H18), 3.10 (t, $^3J_{H\text{-}H}$ = 7.20 Hz, 2H, H21), 3.03 (s, 6H, H20), 1.42 – 1.32 (m, 2H, H22), 0.56 (t, $^3J_{H\text{-}H}$ = 7.4 Hz, 3H, H23); **^{13}C NMR** (151 MHz, CD_2Cl_2, 25 °C) δ (ppm) = 185.0 (C8), 154.2 (C2), 151.7 (C19), 147.0 (C11), 136.2 (C9), 135.3 (C4), 133.7 (2C, C17), 132.4 (2C, C12), 132.4 (2C, C13), 132.2 (C10), 126.2 (C16), 124.6 (C6), 123.4 (C7), 120.0 (C5), 119.1 (C15), 112.0 (C14), 111.5 (C18), 111.3 (C3), 47.6 (C21), 40.4 (2C, C20), 20.7 (C22), 11.3 (C23); ***Z* isomer:** **^{1}H NMR** (601 MHz, CD_2Cl_2, 25 °C) δ (ppm) = 7.62 (m, 2H, H13'), 7.53 (ddd, $^3J_{H\text{-}H}$ = 7.6, $^4J_{H\text{-}H}$ = 1.4, $^5J_{H\text{-}H}$ = 0.7 Hz, 1H, H6'), 7.48 (ddd, $^3J_{H\text{-}H}$ = 8.4, $^3J_{H\text{-}H}$ = 7.2, $^4J_{H\text{-}H}$ = 1.4 Hz, 1H, H4'), 7.35 (m, 2H, H12'), 7.06 (m, 2H, H17'), 7.04 (d, $^3J_{H\text{-}H}$ = 8.3 Hz, 1H, H3'), 6.89 (dd, $^3J_{H\text{-}H}$ = 7.4 Hz, $^4J_{H\text{-}H}$ = 0.7 Hz, 1H, H5'), 6.69 (m, 2H, H18'), 3.40 (t, $^3J_{H\text{-}H}$ = 7.20 Hz, 2H, H21'), 3.01 (s, 6H, H20'), 1.42 – 1.32 (m, 2H, H22'), 0.60 (t, $^3J_{H\text{-}H}$ = 7.4 Hz, 3H, H23'); **^{13}C NMR** (151 MHz,

CD_2Cl_2, 25 °C) δ (ppm) = 185.7 (C8'), 155.0 (C2'), 151.0 (C19'), 146.4 (C11'), 136.0 (C9'), 135.5 (C4'), 133.0 (2C, C17'), 132.6 (2C, C12'), 131.9 (2C, C13'), 131.8 (C10'), 126.6 (C16'), 124.5 (C6'), 123.4 (C7'), 119.9 (C5'), 119.5 (C15'), 111.8 (2C, C18'), 111.7 (C14'), 111.5 (C3'), 47.0 (C21'), 40.3 (2C, C20'), 20.7 (C22'), 11.5 (C23'); **IR**: ṽ (cm^{-1}) = 3047*vw*, 2924*w*, 2220*m*, 1662*s*, 1615*s*, 1602*s*, 1551*s*, 1521*s*, 1400*s*, 1476*s*, 1462*s*, 1439*s*, 1370*s*, 1350*s*, 1300*s*, 1284*s*, 1226*s*, 1189*s*, 1166*s*, 1130*s*, 1100*s*, 1047*s*, 1019*s*, 988*s*, 959*s*, 946*s*, 931*s*, 843*s*, 833*s*, 816*s*, 751*s*, 737*s*, 720*s*, 708*s*, 671*s*, 638*s*, 617*s*, 589*s*, 570*s*, 555*s*, 538*s*, 518*s*, 474*s*, 451*s*; **mp**: 168 – 169 °C; **HR-MS** ($APPI^+$, *m*/*z*): $[MH]^+$ calculated for $[C_{27}H_{26}N_3O]^+$: 408.2070, found 408.2078; $\mathbf{R_f}$ (SiO_2, *i*Hex : EtOAc 3 : 1) = 0.43.

Methyl (*E*/*Z*)-3-(2-((4-(dimethylamino)phenyl)(mesityl)methylene)-3-oxoindolin-1-yl)propanoate (*E*/*Z*-1c)

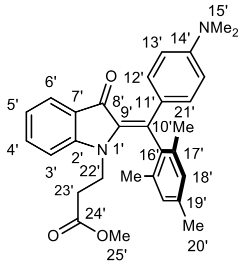

DBU (30 µL, 30 mg, 0.20 mmol, 1.02 g · mL^{-1}, 1.0 equiv.) was added to a solution of *E*/*Z*-**1a** (76 mg, 0.20 mmol, 1.0 equiv.) and methyl acrylate (54 µL, 51 mg, 0.60 mmol, 0.95 g mL^{-1}, 3.0 equiv.) in acetonitrile (0.50 M, 0.78 mL, dry). The resulting red reaction mixture was stirred for 2 d at 50 °C. The reaction progress was monitored by thin-layer chromatography (SiO_2, *i*Hex : EtOAc 5 : 1). The reaction was stopped by the addition of H_2O (50 mL) and the aqueous phase was extracted with EtOAc (3 × 50 mL). The combined organic layers were washed with saturated $KHSO_{4aq.}$ solution (3 × 100 mL), dried over anhydrous Na_2SO_4 and the volatiles were removed *in vacuo*. The crude product was dissolved in CH_2Cl_2, adsorbed on Celite, purified by column chromatography (SiO_2, *i*Hex : EtOAc 100 : 0 → 85 : 15) and recrystallized from MeOH/H_2O to obtain *E*/*Z*-**1c** as fine powdered red crystals (68 mg, 0.14 mmol, 73%).

***E* isomer:** ^{1}**H NMR** (601 MHz, CD_2Cl_2, 25 °C) δ (ppm) = 7.53 (d, $^3J_{H\text{-}H}$ = 7.6 Hz, 1H, H6), 7.50 (ddd, $^3J_{H\text{-}H}$ = 8.4, $^3J_{H\text{-}H}$ = 7.2, $^4J_{H\text{-}H}$ = 1.4 Hz, 1H, H4), 7.19 (m, 2H, H12), 7.17 – 7.12 (m, 1H, H3), 6.94 – 6.87 (m, 3H, H5, H18), 6.65 (d, $^3J_{H\text{-}H}$ = 9.0 Hz, 2H, H13), 4.04 – 3.99 (m, 2H, H13), 3.45 – 3.39 (m, 4H, H25), 2.99 (s, 6H, H15), 2.34 – 2.29 (m, 2H, H22), 2.33 (s, 3H, H20), 2.01 (s, 6H, H21); ^{13}**C NMR** (151 MHz, CD_2Cl_2, 25 °C) δ (ppm) = 185.6 (C8), 172.1 (C24), not assumed (C2), 150.7 (C14), 136.1 (C16), 137.0 (C17), 135.2 (C4), 132.5 (C12), 128.9 (C18), 125.0 (C11), 124.3 (C6), 120.2 (C7), 112.0 (C3), 111.6 (C13), 51.8 (C25), 41.9 (C23), 40.3 (C15), 31.9 (C22), 21.3 (C20), 20.5 (C21); ***Z* Isomer:** ^{1}**H NMR** (601 MHz, CD_2Cl_2, 25 °C) δ (ppm) = 7.59 (ddd, $^3J_{H\text{-}H}$ = 7.6, $^4J_{H\text{-}H}$ = 1.4, $^5J_{H\text{-}H}$ = 0.7 Hz, 1H, H6’), 7.44 (ddd, J = $^3J_{H\text{-}H}$ = 8.4, $^3J_{H\text{-}H}$ = 7.2, $^4J_{H\text{-}H}$ = 1.4 Hz, 1H, H4’), 7.17 – 7.12 (m, 2H, H12’), 6.94 – 6.87 (m, 4H, H3, H5, H18’), 6.60 (m, 2H, H13’), 3.51 (s, 3H, H25’), 3.45 – 3.39 (m, 2H, H23’), 3.00 (s, 6H, H15’), 2.31 (s, 3H, H20’), 2.19 – 2.14 (m, 2H, H22’), 1.99 (s, 6H, H21’); ^{13}**C NMR** (151 MHz, CD_2Cl_2, 25 °C) δ (ppm) = 183.5 (C8’), 171.9 (C24’), 153.3 (C2’), 151.5 (C14’), 138.5 (C17’), 137.3 (C16’), 135.8 (C19’), 135.3 (C4’), 134.9 (C10’), 132.8 (C12’), 129.2 (C18’), 125.3

(C11'), 124.5 (C6'), 123.6 (C7'), 119.8 (C5'), 111.1 (C13'), 110.4 (C3'), 42.3 (C23'), 51.8 (C25'), 40.4 (C15'), 32.3 (C22'), 21.2 (C20'), 20.4 (C21'); **IR**: ṽ (cm^{-1}) = 2912*w*, 1732*m*, 1673*m*, 1600*s*, 1556*m*, 1519*s*, 1474*s*, 1435*m*, 1355*s*, 1315*s*, 1164*s*, 1136*s*, 1096*s*, 1068*s*, 1045*s*, 978*s*, 945*s*, 918*s*, 751*s*, 709*s*, 675*m*, 589*m*, 553*s*, 540*m*, 443*s*; **mp**: 107 – 110 °C; **HR-MS** ($APPI^+$, *m/z*): $[MH]^+$ calculated for$[C_{30}H_{33}N_2O_3]^+$: 470.2519, found 470.2520; $\mathbf{R_f}$ (SiO_2, *i*Hex : EtOAc 5 : 1) = 0.15.

Methyl (*E*/*Z*)-3-(2-((3,5-bis(trifluoromethyl)phenyl)(4-(dimethylamino)-phenyl)methylene)-3-oxoindolin-1-yl)propanoate (*E*/*Z*-2c)

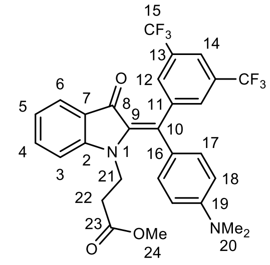

DBU (56 µL, 58 mg, 0.38 mmol, 1.02 g · mL^{-1}, 2.0 equiv.) was added to a solution of *E*/*Z*-**2a** (90 mg, 0.19 mmol, 1.0 equiv.) and methyl acrylate (52 µL, 28 mg, 0.56 mmol, 0.95 g mL^{-1}, 3.0 equiv.) in acetonitrile (0.50 M, 0.38 mL, dry). The resulting red reaction mixture was stirred for 24 h at 50 °C. The reaction progress was monitored by thin-layer chromatography (SiO_2, *i*Hex : EtOAc 3 : 1). The reaction was stopped by addition of H_2O (50 mL) and the aqueous phase was extracted with EtOAc (3 × 50 mL). The combined organic layers were washed with saturated $KHSO_{4aq.}$ solution (3 × 100 mL), dried over anhydrous Na_2SO_4 and the volatiles were removed *in vacuo*. The crude product was dissolved in CH_2Cl_2, adsorbed on Celite, purified by column chromatography (SiO_2, *i*Hex : EtOAc 100 : 0 → 80 : 20), recrystallized from MeOH/H_2O, dissolved in CH_2Cl_2, purified by reversed-phase column chromatography (C_{18} SiO_2, MeOH/H_2O 10 : 90 → 75 : 25) and recrystallized in MeOH/H_2O to obtain *E*/*Z*-**2c** as red fine powdered crystals (43 g, 0.077 mmol, 41%).

***E* isomer: ^{1}H NMR** (500 MHz, CD_2Cl_2, 25 °C) δ (ppm) = 7.90 (s, 1H, H14), 7.74 (s, 2H, H12), 7.56 (ddd, $^3J_{H\text{-}H}$ = 7.6 Hz, $^4J_{H\text{-}H}$ = 1.4 Hz, $^5J_{H\text{-}H}$ = 0.7 Hz, 1H, H6), 7.52 (ddd, $^3J_{H\text{-}H}$ = 8.4 Hz, $^3J_{H\text{-}H}$ = 7.2 Hz, $^4J_{H\text{-}H}$ = 1.4 Hz, 1H, H4), 7.13 – 7.07 (m, 3H, H17, H3), 6.95 (t, $^3J_{H\text{-}H}$ = 7.40 Hz, 1H, H5), 6.74 – 6.69 (m, 2H, H18), 3.83 (t, $^3J_{H\text{-}H}$ = 6.9 Hz, 2H, H21), 3.43 (s, 3H, H24), 3.04 (s, 6H, H20), 2.35 (t, $^3J_{H\text{-}H}$ = 6.9 Hz, 2H, H22); **^{13}C NMR** (126 MHz, CD_2Cl_2, 25 °C) δ (ppm) = 185.5 (C8), 171.9 (C23), 154.4 (C2), 151.2 (C19), 135.8 (C4), 135.3 (C9), 133.8 (C11), 133.1 (2C, C17), 132.3 (2C, C12), 132.0 (C13), 131.2 (C10), 125.5 (C16), 124.7 (C6), 124.0 (C7), 123.8 (C15), 122.1 (C14), 120.7 (C5), 111.9 (2C, C18), 111.8 (C3), 51.9 (C24), 41.3 (C21), 40.3 (2C, C20), 32.1 (C22); ***Z* isomer: ^{1}H NMR** (500 MHz, CD_2Cl_2, 25 °C) δ (ppm) = 7.91 (s, 1H, H14'), 7.81 (s, 2H, H12'), 7.67 (t, $^3J_{H\text{-}H}$ = 7.6 Hz), 7.62 (d, $^3J_{H\text{-}H}$ = 7.6 Hz, 1H, H6'), 7.13 – 7.07 (m, 2H, H17'), 7.04 (d, $^3J_{H\text{-}H}$ = 8.3 Hz, 1H, H3'), 6.98 (t, $^3J_{H\text{-}H}$ = 7.40 Hz, 1H, H5'), 6.69 – 6.66 (m, 2H, H18'), 3.47 (t, $^3J_{H\text{-}H}$ = 6.9 Hz, 2H, H21'), 3.44 (s, 3H, H24'), 3.05 (s, 6H, H20'),

2.31 (t, $^{3}J_{H\text{-}H}$ = 7.1 Hz, 2H, H22'); **^{13}C NMR** (126 MHz, CD_2Cl_2, 25 °C) δ (ppm) = 171.9 (C23'),153.4 (C2'), 152.0 (C19'), 132.0 (2C, C12'), 131.0 (C10'), 125.3 (C16'), 120.8 (C5'), 111.5 (C3'), 52.0 (C24'), 42.0 (C21'), 40.3 (2C, C20'), 32.3 (C22'), C4', C6', C7', C8', C9', C11', C13', C14', C15', C17', C18', C24' were not found; **IR**: ṽ (cm^{-1}) = 2917*w*, 2849*w*, 1735*m*, 1676*m*, 1600*s*, 1520*s*, 1475*m*, 1444*m*, 1365*m*, 13614*m*, 1274*s*, 1164*s*, 1122*vs*, 1097*vs*, 1067*s*, 966*s*, 894*s*, 845*m*, 817*s*, 751*s*, 713*m*, 703*m*, 682*s*, 670*m*, 586*m*, 548*m*, 443*s*; **mp**: 103 – 105 °C; HR-MS (APPI^{+}, *m*/*z*): $[MH]^{+}$ calculated for $[C_{29}H_{25}F_6N_2O_3]^{+}$: 563.1764, found 563.1770; **R$_f$** (SiO_2, *i*Hex : EtOAc 3 : 1) = 0.37.

Methyl (*E*/*Z*)-3-(2-((3,5-dimethoxyphenyl)(4-(dimethylamino)phenyl)methylene)-3-oxoindolin-1-yl)propanoate (*E*/*Z*-3c)

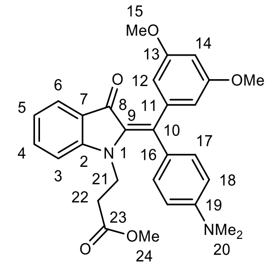

DBU (34 µL, 34 mg, 0.22 mmol, 1.02 g mL^{-1}, 1.0 equiv.) was added to a solution of *E*/*Z*-**3a** (90 mg, 0.23 mmol, 1.0 equiv.) and methyl acrylate (61 µL, 58 mg, 0.68 mmol, 0.95 g mL^{-1}, 3.0 equiv.) in acetonitrile (0.50 M, 0.45 mL, dry). The resulting red reaction mixture was stirred for 61 h at 50 °C. The reaction progress was monitored by thin-layer chromatography (SiO_2, *i*Hex : EtOAc 2 : 1). The reaction was stopped by the addition of H_2O (50 mL) and the aqueous phase was extracted with EtOAc (3 × 50 mL). The combined organic layers were washed with saturated $KHSO_{4\,aq.}$ solution (3 × 100 mL), dried over anhydrous Na_2SO_4 and the volatiles were removed *in vacuo*. The crude product was recrystallized from MeOH/H_2O, dissolved in CH_2Cl_2, adsorbed on Celite, purified by column chromatography (SiO_2, *i*Hex : EtOAc 100 : 0 → 70 : 30) and recrystallized from MeOH/H_2O to obtain *E*/*Z*-**3c** as red fine powdered crystals (58 mg, 0.12 mmol, 53%).

***E* isomer: ^{1}H NMR** (601 MHz, CD_2Cl_2, 25 °C) δ (ppm) = 7.57 (d, $^3J_{H\text{-}H}$ = 7.6 Hz, 1H, H6), 7.48 (ddd, $^3J_{H\text{-}H}$ = 8.4 Hz, $^3J_{H\text{-}H}$ = 7.3 Hz, $^3J_{H\text{-}H}$ = 1.4 Hz, 1H, H4), 7.15 (m, 2H, H17), 7.08 (d, $^3J_{H\text{-}H}$ = 8.3, 1H, H3), 6.92 (dt, $^3J_{H\text{-}H}$ = 7.3 Hz, $^4J_{H\text{-}H}$ = 0.8 Hz, 1H, H5), 6.68 (m, 2H, H18), 6.51 (t, $^4J_{H\text{-}H}$ = 2.3 Hz, 1H, H14), 6.41 (d, $^4J_{H\text{-}H}$ = 2.3 Hz, 2H, H12), 3.78 (t, $^3J_{H\text{-}H}$ = 7.0 Hz, 2H, H21), 3.76 (s, 6H, H15), 3.40 (s, 3H, H24), 3.02 (s, 6H, H20), 2.31 – 2.27 (m, 2H, H22); **^{13}C NMR** (151 MHz, CD_2Cl_2, 25 °C) δ (ppm) = 185.2 (C8), 172.1 (C23), 160.8 (C13), 154.1 (C2), 151.0 (C19), 143.6 (C9), 135.3 (C10), 135.1 (C4), 134.6 (C11), 133.2 (C17), 126.8 (C16), 124.4 (C6), 124.4 (C7), 120.2 (C5), 111.7 (C3), 111.7 (C18), 110.1 (C12), 100.6 (C14), 55.7 (C15), 51.8 (C24), 41.5 (C21), 40.3 (C20), 32.0 (C22);
***Z* isomer: ^{1}H NMR** (601 MHz, CD_2Cl_2, 25 °C) δ (ppm) = 7.59 (d, $^3J_{H\text{-}H}$ = 7.6 Hz, 1H, H6'), 7.46 (ddd, $^3J_{H\text{-}H}$ = 8.4 Hz, $^3J_{H\text{-}H}$ = 7.2 Hz, $^4J_{H\text{-}H}$ = 1.4 Hz, 1H, H4'), 7.17 (m, 2H, H17'), 7.01 (d, $^3J_{H\text{-}H}$ = 8.3, 1H, H3'), 6.91 (dt, $^3J_{H\text{-}H}$ = 7.3 Hz, $^4J_{H\text{-}H}$ = 0.8 Hz, 1H, H5'), 6.65 (m, 2H, H18'), 6.49 (t, $^4J_{H\text{-}H}$ = 2.3 Hz, 1H, H14'), 6.43 (d, $^4J_{H\text{-}H}$ = 2.3 Hz, 2H, H12'), 3.75 (s, 6H, H15'), 3.58

– 3.54 (m, 2H, H21’), 3.44 (s, 3H, H24’), 3.03 (s, 6H, H20’), 2.35 – 2.31 (m, 2H, H22’); **^{13}C NMR** (151 MHz, CD_2Cl_2, 25 °C) δ (ppm) = 184.7 (C8’), 171.9 (C23’), 161.1 (C13’), 153.5 (C2’) 151.8 (C19’), 143.0 (C9’), 136.4 (C10’), 135.0 (C4’), 134.6 (C11’), 133.9 (C17’), 126.4 (C16’), 124.4 (C6’), 124.0 (C7’), 119.9 (C5’), 111.2 (C18’), 111.2 (C3’), 109.7 (C12’), 101.1 (C14’), 55.9 (C15’), 51.8 (C24’), 41.5 (C21’), 40.4 (C20’), 32.2 (C22’); **IR**: ṽ (cm^{-1}) = 2932*w*, 1070*vw*, 1731*s*, 1670*m*, 1597*vs*, 1550*m*, 1517*s*, 1474*s*, 1420*s*, 1313*s*, 1251*m*, 1191*s*, 1149*vs*, 1097*s*, 1060*s*, 1042*s*, 1006*m*, 970*m*, 942*m*, 817*s*, 750*s*, 733*s*, 705*m*, 672*m*, 551*m*, 518*m*, 436*m*; **mp**: 94 – 96 °C; HR-MS ($APPI^+$, *m*/*z*): $[MH]^+$ calculated for $[C_{29}H_{31}N_2O_5]^+$: 487.2227, found 487.2234; **R_f** (SiO_2, *i*Hex : EtOAc 2 : 1) = 0.28.

Methyl (*E*/*Z*)-3-(2-((4-cyanophenyl)(4-(dimethylamino)phenyl)methylene)-3-oxoindolin-1-yl)propanoate (*E*/*Z*-4c)

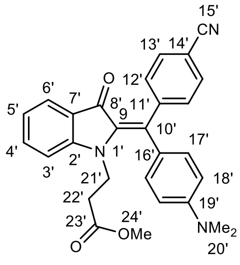

1,8-Diazabicyclo(5.4.0)undec-7-ene (DBU, 33 µL, 33 mg, 0.22 mmol, 1.02 g mL^{-1}, 1.0 equiv.) was added to a solution of *E*/*Z*-**4a** (80 mg, 0.22 mmol, 1.0 equiv.) and methyl acrylate (40 µL, 38 mg, 0.44 mmol, 0.95 g mL^{-1}, 2.0 equiv.) in acetonitrile (0.50 M, 0.44 mL, dry). The resulting red reaction mixture was stirred for 24 h at 50 °C. The reaction progress was monitored by thin-layer chromatography (SiO_2, *i*Hex : EtOAc 5 : 1). The reaction was stopped by addition of H_2O (50 mL) and the aqueous phase was extracted with EtOAc (3 × 50 mL). The combined organic phases were washed with a saturated $KHSO_{4\,aq.}$ solution (3 × 100 mL), dried over anhydrous Na_2SO_4 and the volatiles were removed *in vacuo*. The crude product was dissolved in CH_2Cl_2, adsorbed on Celite, purified by column chromatography (SiO_2, *i*Hex : EtOAc 100 : 0 → 70 : 30) and recrystallized from MeOH/H_2O to obtain *E*/*Z*-**4c** as red fine powdered crystals (0.53 g, 0.12 mmol, 54%).

***E* isomer:** ^{1}**H NMR** (601 MHz, CD_2Cl_2, 25 °C) δ (ppm) = 7.69 (m, 2H, H13), 7.60 (ddd, $^3J_{H\text{-}H}$ = 7.6 Hz, $^4J_{H\text{-}H}$ = 1.4 Hz, $^5J_{H\text{-}H}$ = 0.7 Hz, 1H, H6), 7.52–7.48 (m, 1H, H4), 7.43 (m, 2H, H12), 7.11–7.07 (m, 2H, H17), 7.02 (d, $^3J_{H\text{-}H}$ = 8.3 Hz, 1H, H3), 6.96 (t, $^3J_{H\text{-}H}$ = 7.4 Hz, 1H, H5), 6.66 (m, 2H, H18), 3.50 (t, $^3J_{H\text{-}H}$ = 7.00 Hz, 2H, H21), 3.44 (s, 3H, H24), 3.04 (s, 6H, H20), 2.29 (t, $^3J_{H\text{-}H}$ = 6.90 Hz 7.0 Hz, 2H, H22); ^{13}**C NMR** (151 MHz, CD_2Cl_2, 25 °C) δ (ppm) = 184.7 (C8), 171.6 (C23), 153.5 (C2), 151.9 (C19), 146.6 (C11), 135.4 (C4), 135.3 (C9), 133.9 (2C, C17), 133.3 (C10), 132.5 (2C, C12), 132.5 (2C, C13), 125.7 (C16), 124.6 (C6), 123.9 (C7), 120.6 (C5), 119.0 (C15), 112.3 (C14), 111.5 (C3), 111.4 (2C, C18), 51.9 (C24), 41.9 (C21), 40.4 (2C, C20), 32.0 (C22); ***Z* isomer:** ^{1}**H NMR** (601 MHz, CD_2Cl_2, 25 °C) δ (ppm) = 7.63 (m, 2H, H13'), 7.55 (ddd, $^3J_{H\text{-}H}$ = 7.6 Hz, $^4J_{H\text{-}H}$ = 1.4 Hz, $^5J_{H\text{-}H}$ = 0.7 Hz, 1H, H6'), 7.52–7.48 (m, 1H, H4'), 7.37 (m, 2H, H12'), 7.11–7.07 (m, 3H, H3,17'), 6.94 (t, $^3J_{H\text{-}H}$ = 7.4 Hz, 1H, H5'), 6.69 (m, 2H, H18'), 3.80 (t, $^3J_{H\text{-}H}$ = 7.00 Hz, 2H, H21'), 3.40 (s, 3H, H24'), 3.02 (s, 6H, H20'), 2.31 (t, $^3J_{H\text{-}H}$ = 6.90 Hz 7.0 Hz, 2H, H22'); ^{13}**C NMR** (151 MHz, CD_2Cl_2, 25 °C) δ (ppm) = 185.5 (C8'), 171.9 (C23'), 154.3 (C2'), 151.1 (C19'), 146.0 (C11'), 135.6 (C4'), 135.1 (C9'), 133.1 (2C, C17'), 132.7 (C10'), 132.6 (2C, C12'), 132.0 (2C, C13'), 126.0 (C16'), 124.5 (C6'),

123.9 (C7'), 120.6 (C5'), 119.5 (C15'), 111.9 (C14'), 111.8 (2C, C18'), 111.7 (C3'), 51.9 (C24'), 41.3 (C21'), 40.3 (2C, C20'), 32.0 (C22'); **IR**: ṽ (cm^{-1}) = 2222*w*, 1724*w*, 1670*w*, 1602*m*, 1519*w*, 1476*w*, 1365*m*, 1316*m*, 1046*s*, 925*s*, 917*s*, 751*s*, 672*s*, 424*vs*; **mp**: 138 – 140 °C; **HR-MS** ($APPI^+$, *m/z*): $[MH]^+$ calculated for $[C_{27}H_{26}N_3O]^+$: 452.1969, found 452.1968; **R_f** (SiO_2, *i*Hex : EtOAc 5 : 1) = 0.1.

Supplementary Note 3: NMR spectra

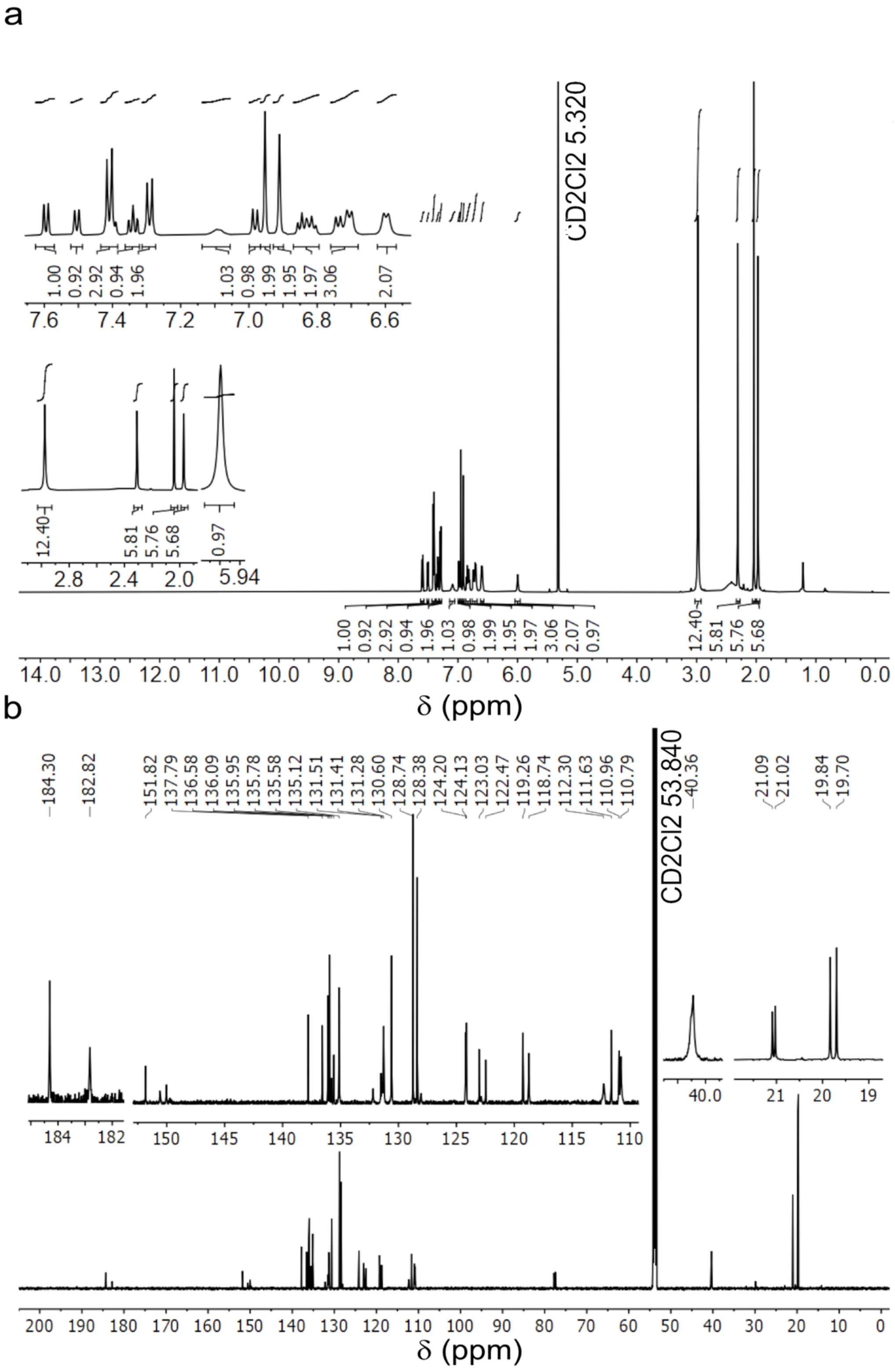

Supplementary Figure 1: NMR spectra of **1a** with isomer ratio of $E : Z = 1 : 1.1$. **a** ^{1}H NMR spectrum (601 MHz, CD_2Cl_2, 25 °C). **b** ^{13}C NMR spectrum (151 MHz, CD_2Cl_2, 25 °C). Source data are provided as Source Data File.

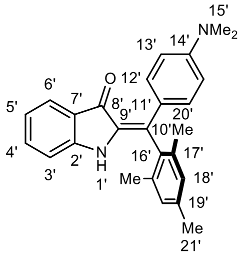

Z-**1a**

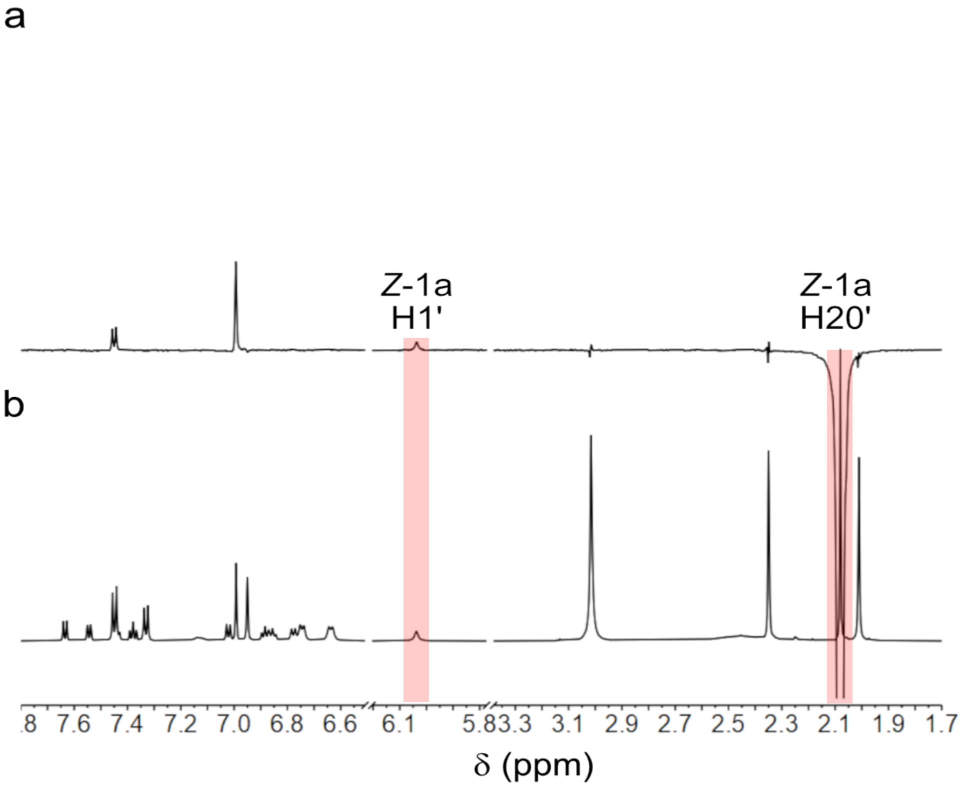

Supplementary Figure 2: Partial 1D ^{1}H-^{1}H NOE NMR experiment with **1a**. Irradiating the protons at 2.08 ppm (H20' of *Z*-**1a**) of a *E*/*Z*-**1a** mixture (*E* : *Z* = 1 : 1.1) leads to magnetization transfer to the signal at 6.04 ppm (H1' of *Z*-**1a**). This confirms that the thermally more stable isomer in the *E*/*Z* mixture (determined by heating experiments) is the bathochromic *Z* configured isomer. **a** 1D ^{1}H-^{1}H NOE NMR spectrum (601 MHz, CD_2Cl_2, –20 °C). **b** ^{1}H NMR spectrum (601 MHz, CD_2Cl_2, 25 °C). Source data are provided as Source Data File.

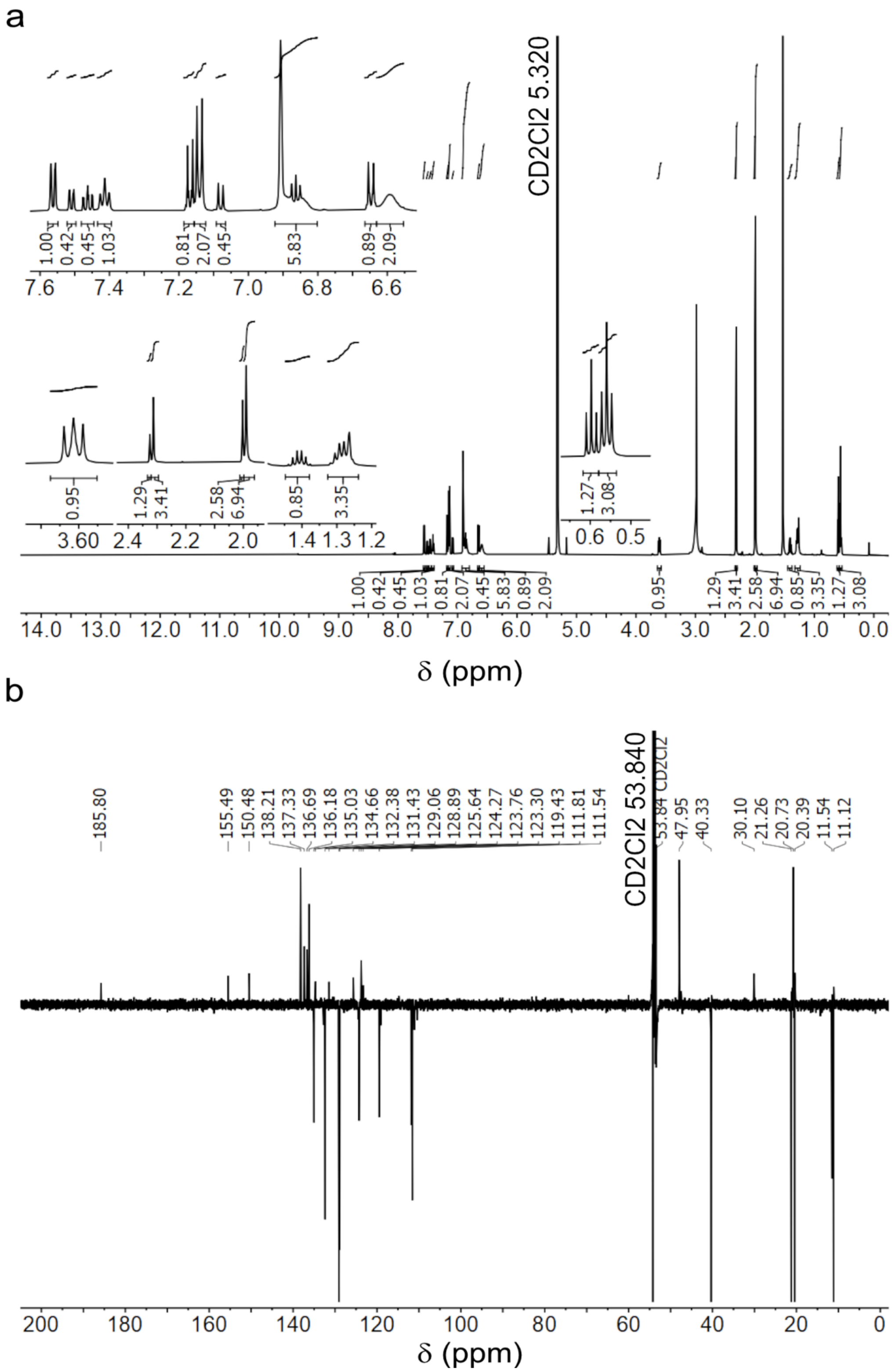

Supplementary Figure 3: NMR spectra of **1b** with isomer ratio of $E : Z = 1 : 2.4$. **a** ^{1}H NMR spectrum (601 MHz, CD_2Cl_2, 25 °C). **b** ^{13}C(DEPTq135) NMR spectrum (151 MHz, CD_2Cl_2, 25 °C). Source data are provided as Source Data File.

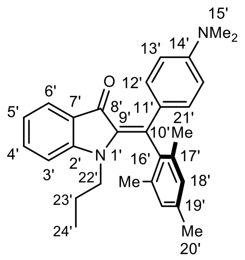

***Z*-1b**

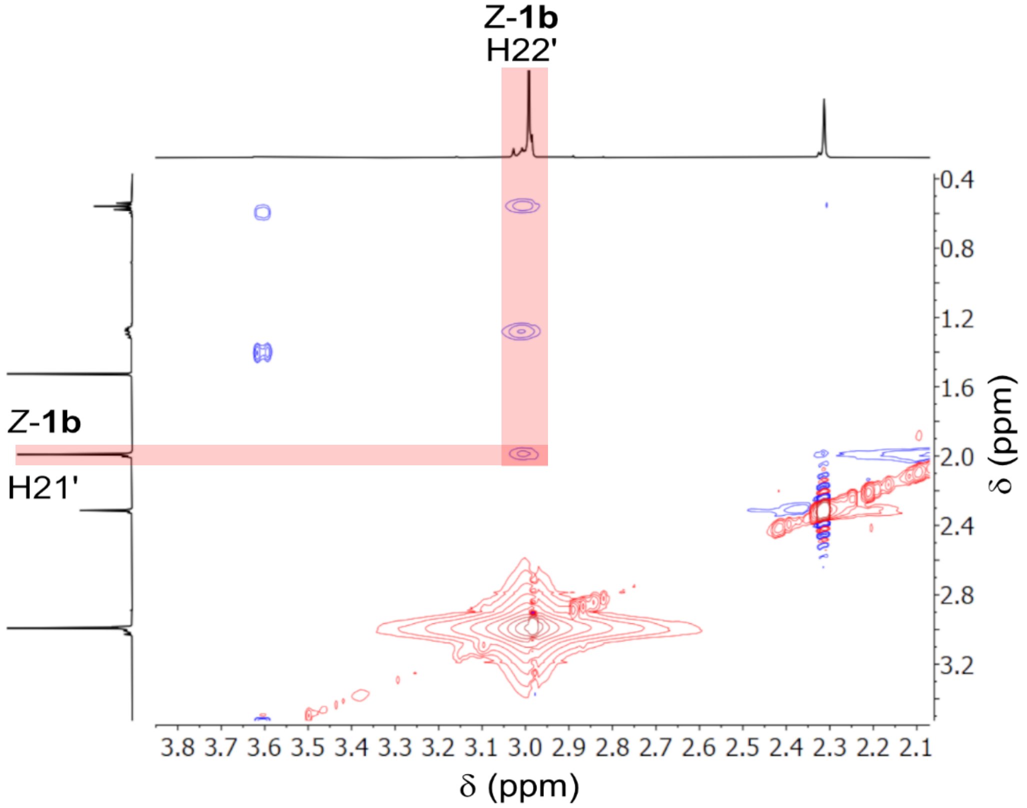

Supplementary Figure 4: Partial 2D ^{1}H-^{1}H NOESY NMR spectrum (601 MHz, CD_2Cl_2, 25 °C) of a *E*/*Z*-isomeric mixture (*E* : *Z* = 1 : 2.4) of **1b**. The cross signals between the protons at 3.10 – 2.87 ppm (H22' of *Z*-**1b**) and 1.99 ppm (H21' of *Z*-**1b**) confirm that the thermally more stable isomer is the bathochromic *Z* isomer. Source data are provided as Source Data File.

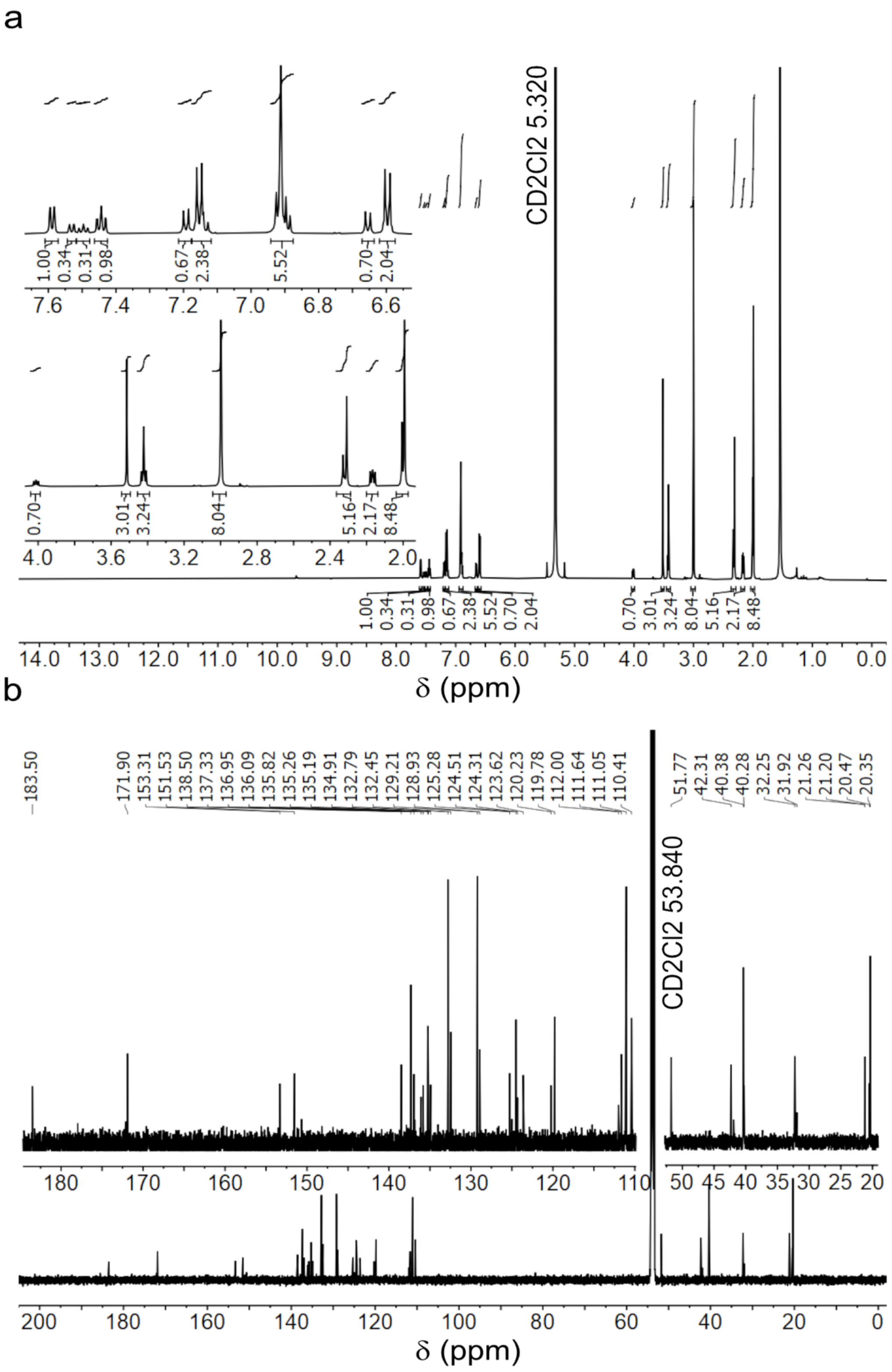

Supplementary Figure 5: NMR spectra of **1c** with isomer ratio of *E* : *Z* = 1 : 2.9. **a** ^{1}H NMR spectrum (601 MHz, CD_2Cl_2, 25 °C). **b** ^{13}C NMR spectrum (151 MHz, CD_2Cl_2, 25 °C). Source data are provided as Source Data File.

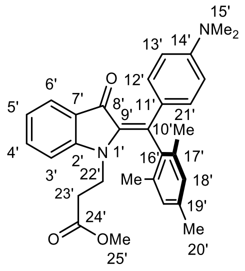

Z-**1c**

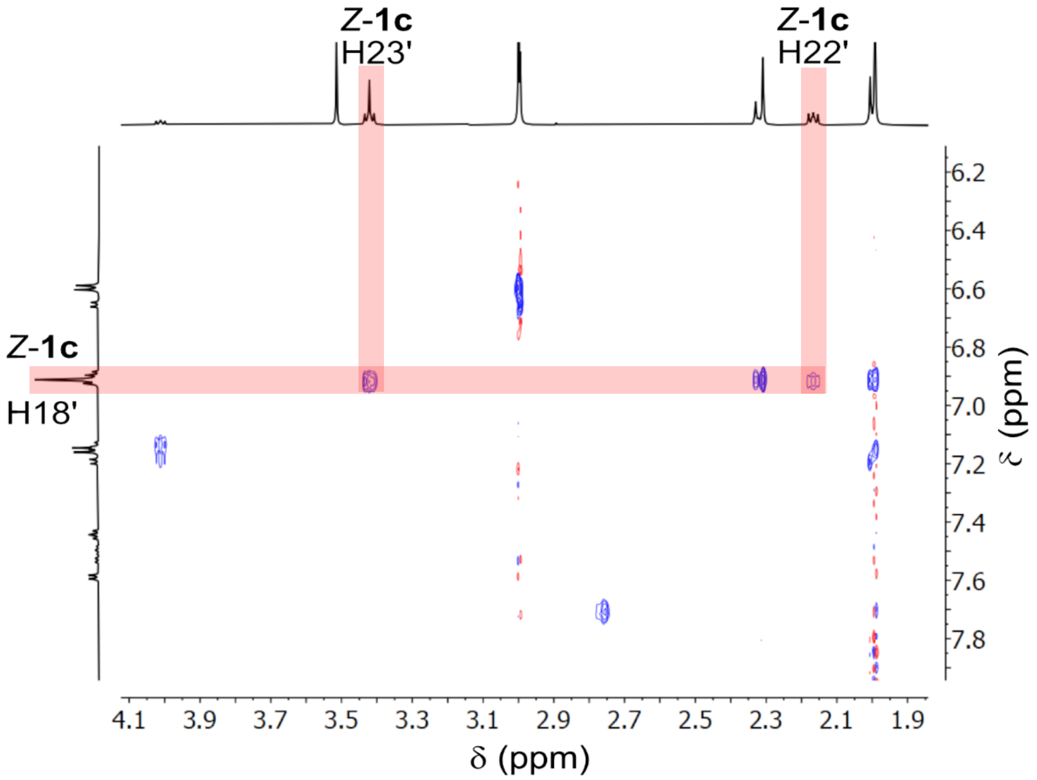

Supplementary Figure 6: Partial 2D ^{1}H-^{1}H NOESY NMR spectrum (601 MHz, CD_2Cl_2, 25 °C) of a *E*/*Z*-isomeric mixture (*E* : *Z* = 1 : 2.9) of **1c**. The cross signals between the protons at 2.19 – 2.14 ppm (H22' of *Z*-**1c**) and 3.45 – 3.39 (H23' of *Z*-**1c**) confirm that the thermally more stable isomer is the bathochromic *Z* isomer. Source data are provided as Source Data File.

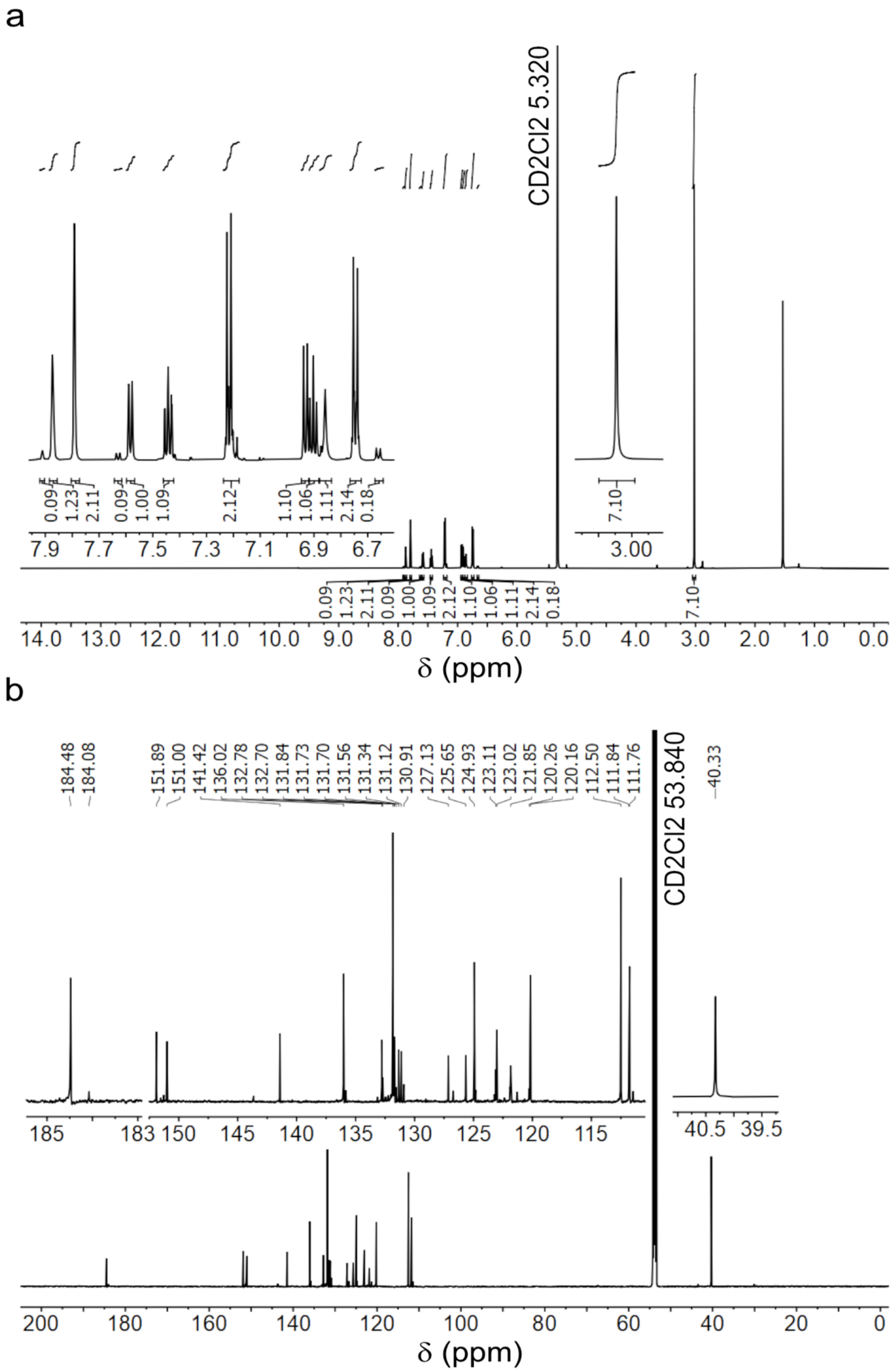

Supplementary Figure 7: NMR spectra of **2a** with isomer ratio of $E : Z = 10 : 1$. **a** ^{1}H NMR spectrum (601 MHz, CD_2Cl_2, 25 °C). **b** ^{13}C NMR spectrum (151 MHz, CD_2Cl_2, 25 °C). Source data are provided as Source Data File.

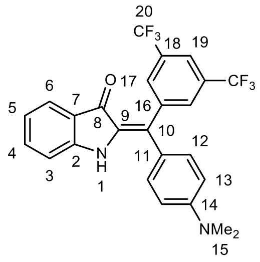

E-**2a**

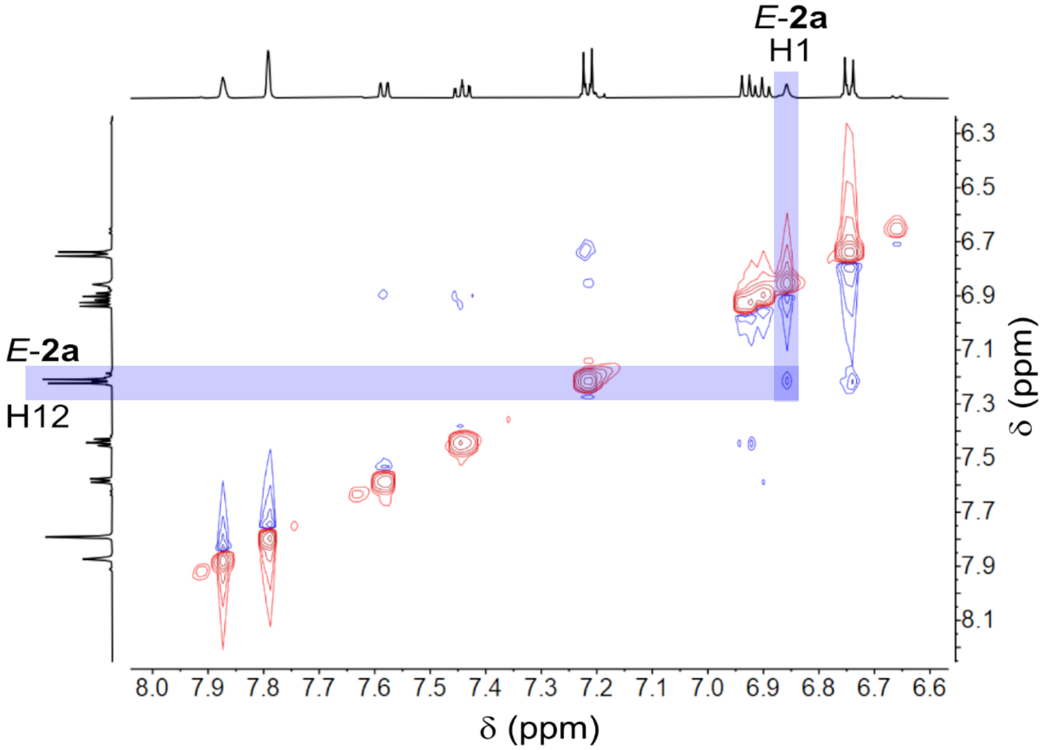

Supplementary Figure 8: Partial 2D ^{1}H-^{1}H NOESY NMR spectrum (601 MHz, CD_2Cl_2, 25 °C) of a *E*/*Z*-isomeric mixture (*E* : *Z* = 10 : 1) of **2a**. The cross signal between the protons at 7.22 ppm (H12 of *E*-**2a**) and 6.86 ppm (H1 of *E*-**2a**) confirms that the thermally more stable isomer is the hypsochromic *E* isomer. Source data are provided as Source Data File.

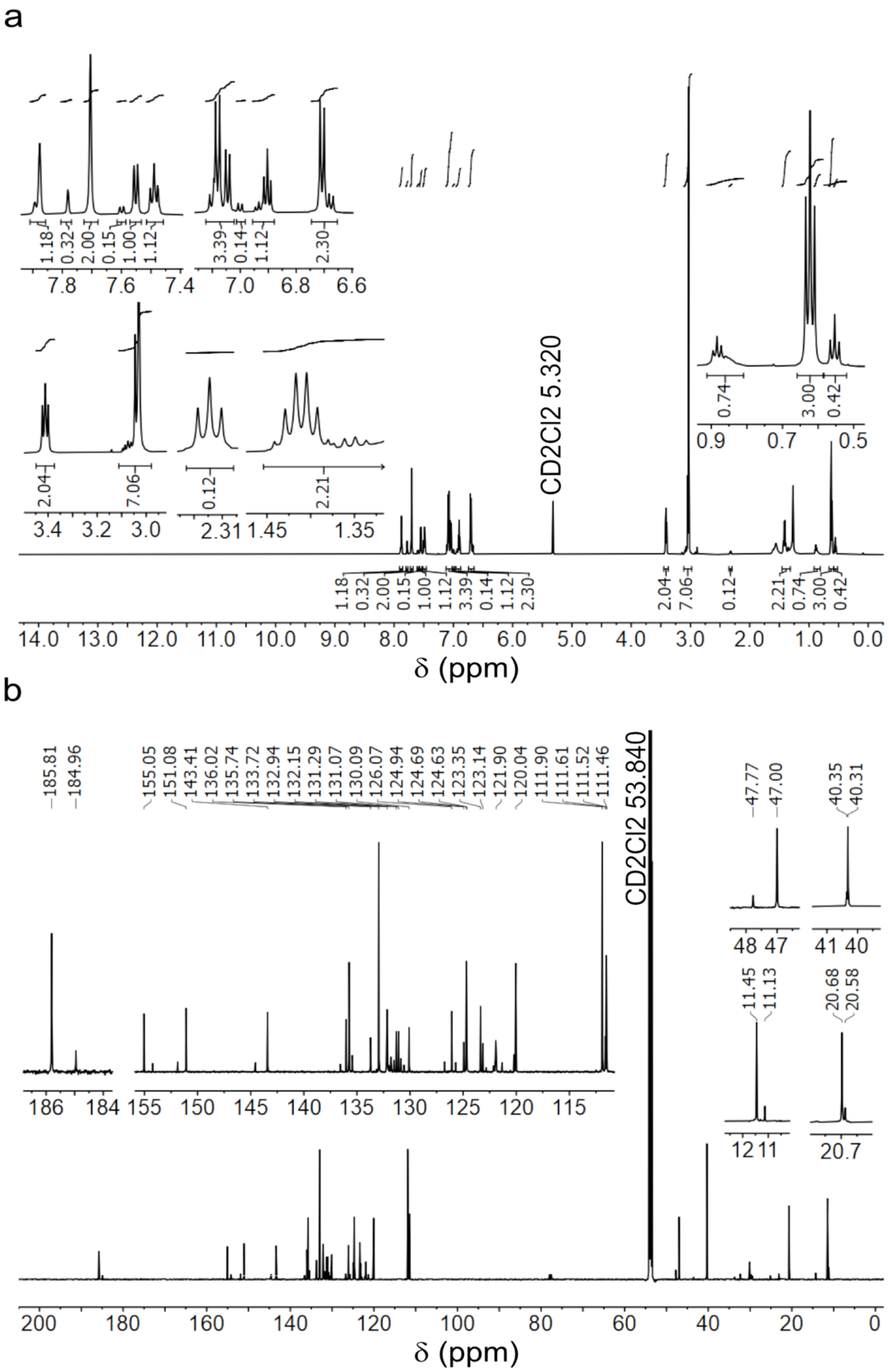

Supplementary Figure 9: NMR spectra of **2b** with isomer ratio of *E* : *Z* = 6.3 : 1. **a** ^{1}H NMR spectrum (601 MHz, CD_2Cl_2, 25 °C). **b** ^{13}C NMR spectrum (151 MHz, CD_2Cl_2, 25 °C). Source data are provided as Source Data File.

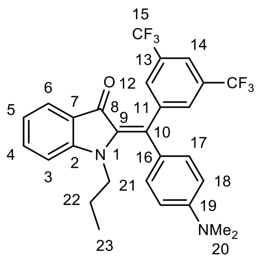

E-**2b**

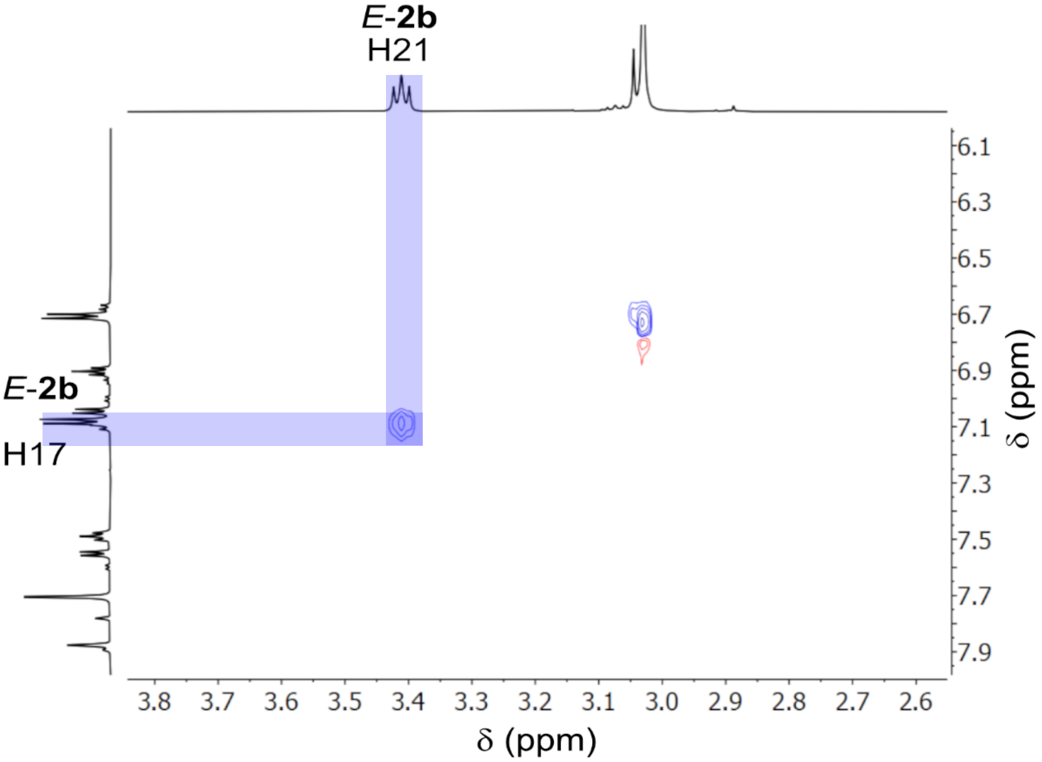

Supplementary Figure 10: Partial 2D ^{1}H-^{1}H NOESY NMR spectrum (601 MHz, CD_2Cl_2, 25 °C) of a *E*/*Z*-isomeric mixture (*E* : *Z* = 6.3 : 1) of **2b**. The cross signal between the protons at 7.08 ppm (H17 of *E*-**2b**) and 3.41 ppm (H21 of *E*-**2b**) confirms that the thermally more stable isomer is the hypsochromic *E* isomer. Source data are provided as Source Data File.

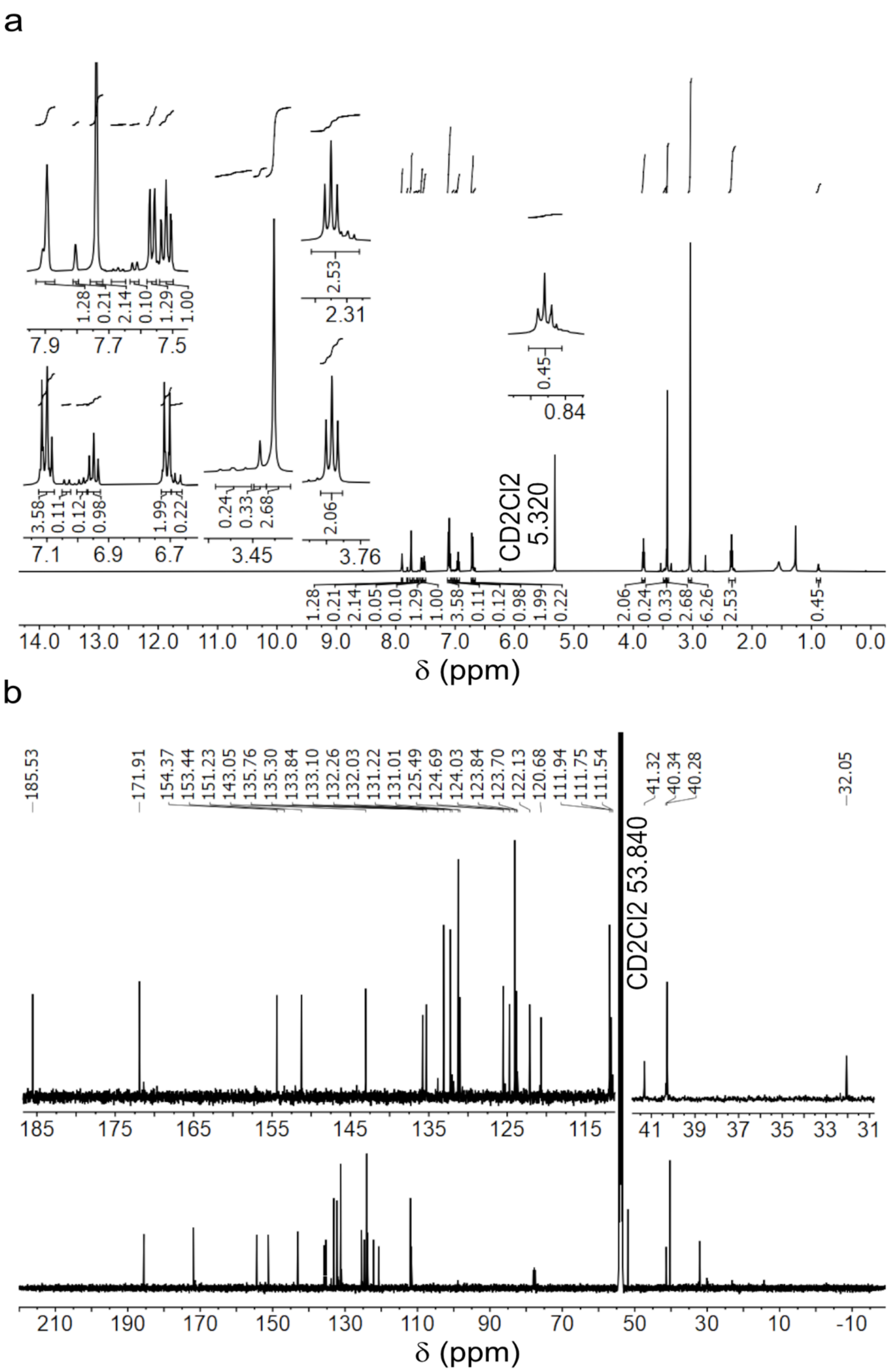

Supplementary Figure 11: NMR spectra of **2c** with isomer ratio of *E* : *Z* = 13 : 1. **a** ^{1}H NMR spectrum (500 MHz, CD_2Cl_2, 25 °C). **b** ^{13}C NMR spectrum (126 MHz, CD_2Cl_2, 25 °C). Source data are provided as Source Data File.

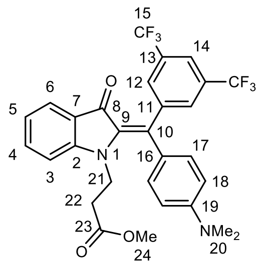

E-**2c**

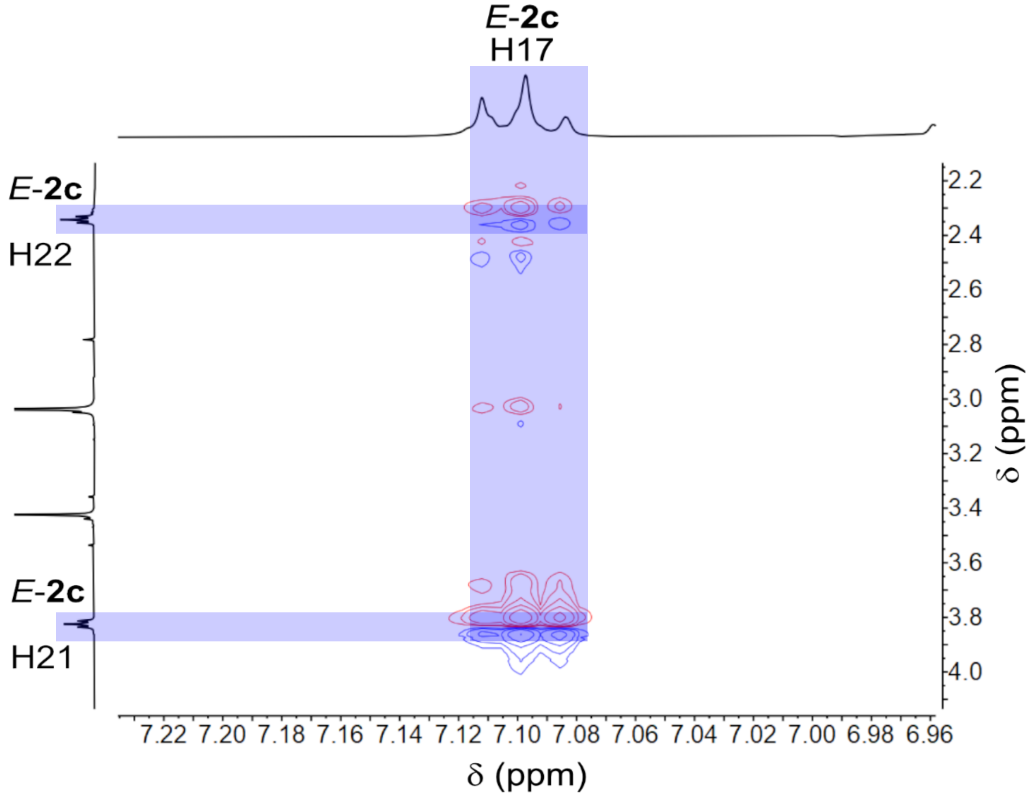

Supplementary Figure 12: Partial 2D ^{1}H-^{1}H NOESY NMR spectrum (601 MHz, CD_2Cl_2, 25 °C) of a *E*/*Z*-isomeric mixture (*E* : *Z* = 16 : 1) of **2c**. The cross signals between the protons at 7.13 – 7.07 ppm (H17 of *E*-**2c**) with those at 3.83 ppm (H21 of *E*-**2c**) and 2.35 (H22 of *E*-**2c**) confirm that the thermally more stable isomer is the hypsochromic *E* isomer. Source data are provided as Source Data File.

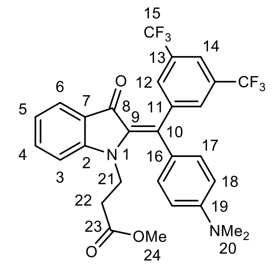

E-**2c**

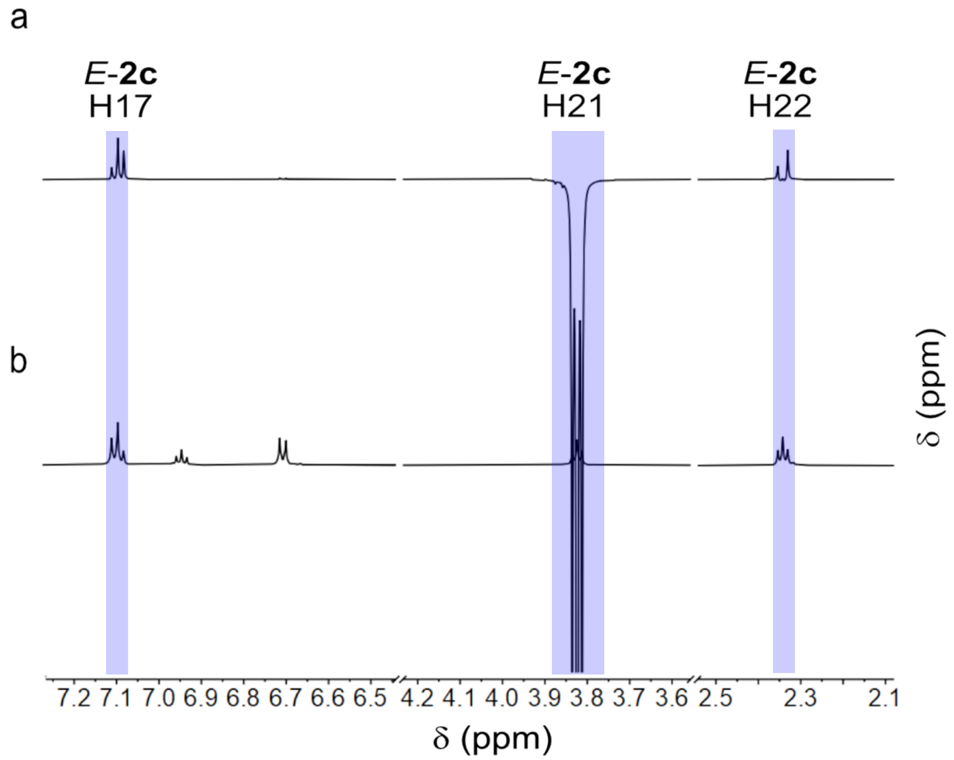

Supplementary Figure 13: 1D ^{1}H-^{1}H NOE NMR experiment of a *E*/*Z*-isomeric mixture (*E* : *Z* = 16 : 1) of **2c**. Irradiating the protons at 3.83 ppm (H21 of *E*-**2c**) leads to signal increase at 7.13 – 7.07 ppm (H17 of *E*-**2c**) and 2.35 (H22 of *E*-**2c**). This confirms that the thermally more stable isomer is the hypsochromic *E* isomer. **a** 1D ^{1}H-^{1}H NOE NMR spectrum (601 MHz, CD_2Cl_2, 25 °C). **b** ^{1}H NMR spectrum (601 MHz, CD_2Cl_2, 25 °C). Source data are provided as Source Data File.

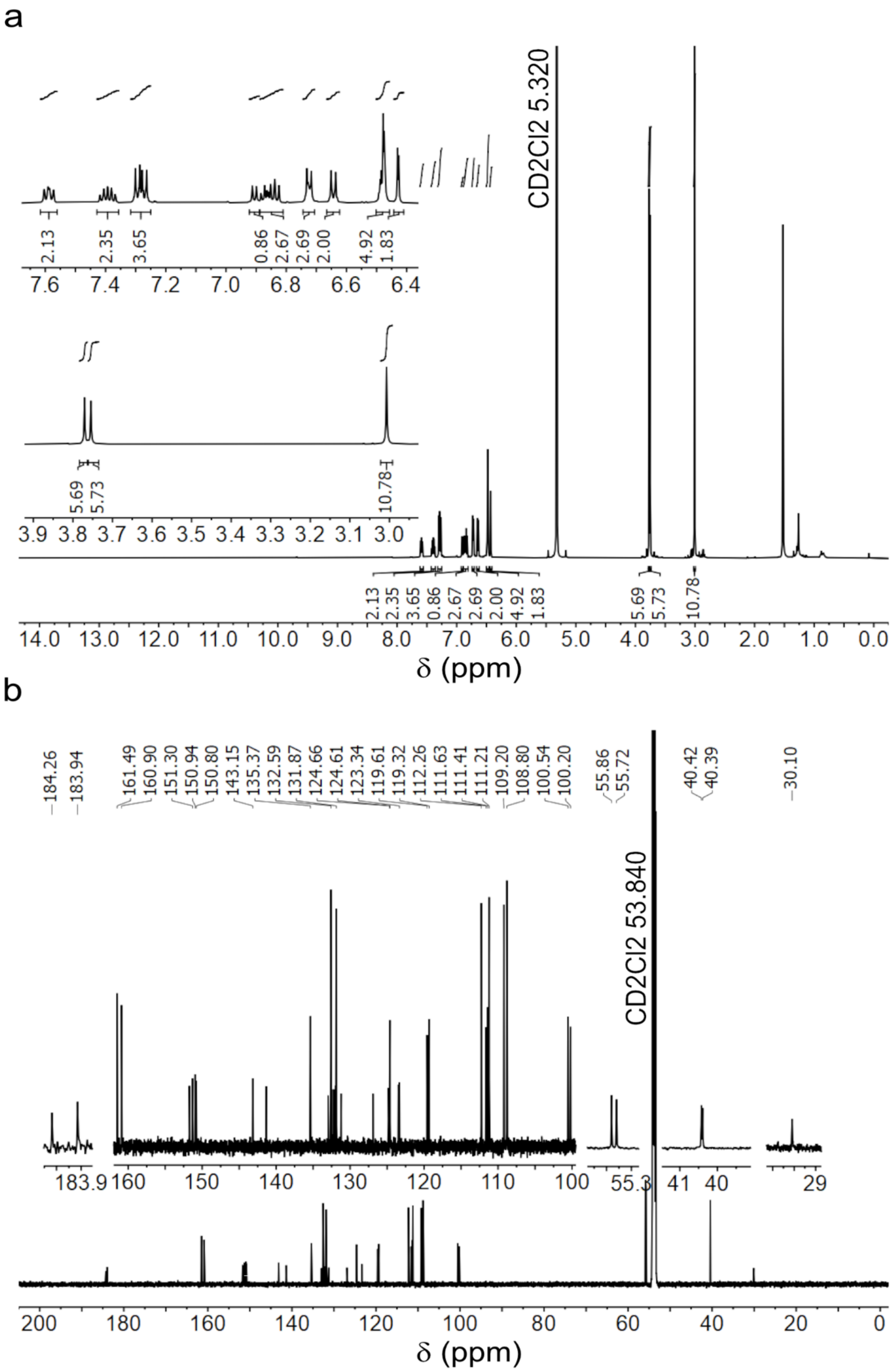

Supplementary Figure 14: NMR spectra of **3a** with isomer ratio of almost *E* : *Z* = 1 : 1. **a** ^{1}H NMR spectrum (601 MHz, CD_2Cl_2, 25 °C). **b** ^{13}C NMR spectrum (151 MHz, CD_2Cl_2, 25 °C). Source data are provided as Source Data File.

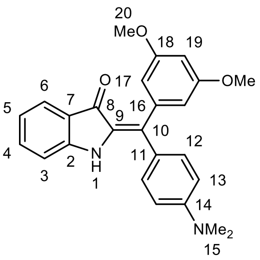

E-**3a**

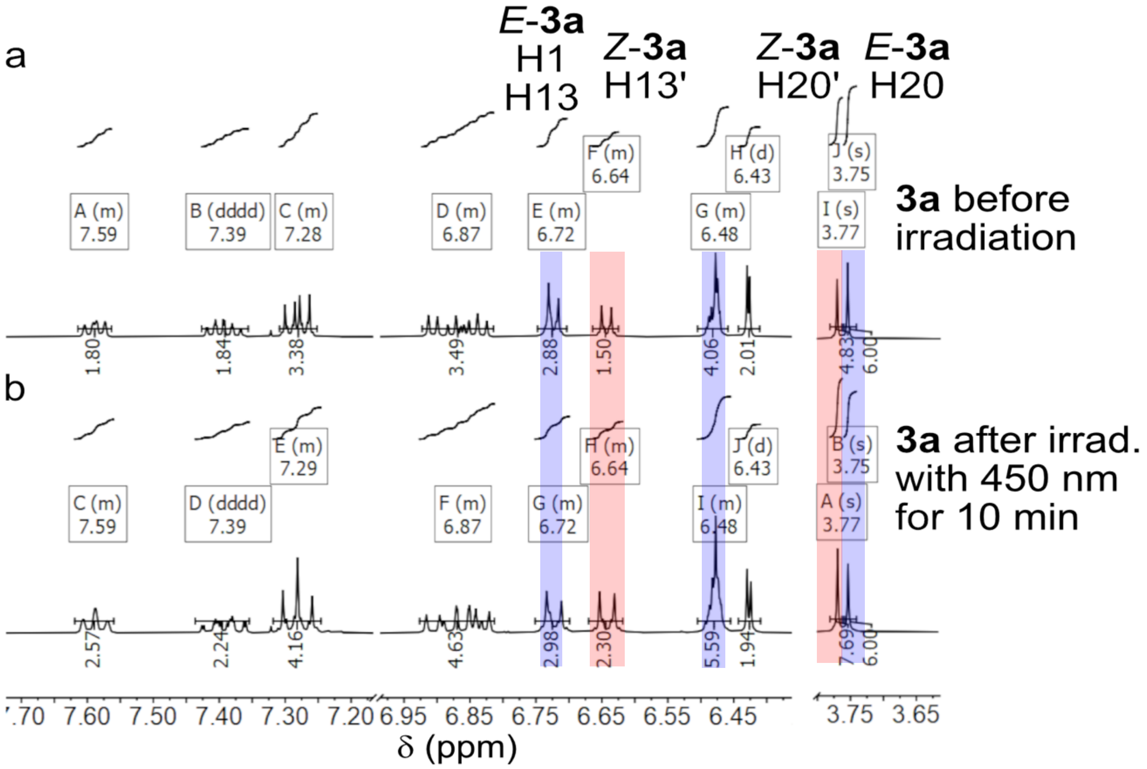

Supplementary Figure 15: Partial ^{1}H NMR spectra of **3a** before (601 MHz, CD_2Cl_2, 25 °C) and after irradiation with 450 nm light (400 MHz, CD_2Cl_2, 25 °C). **a** ^{1}H NMR spectrum of a thermally equilibrated *E*/*Z* isomer mixture of **3a** shows an isomer ratio of 1.24 : 1 (H20 : H20’). The multiplet at 6.72 ppm shows three proton signals that belong to the hypsochromic isomer (see b). **b** ^{1}H NMR spectrum obtained after irradiation of **3a** with 450 nm light for 10 min. The isomer ratio changes to 1: 1.28 (H20 : H20’). When comparing spectra before (a) and after irradiation (b)) the distinction between the two signal sets can be made. The bathochromic isomer is now enriched. In a thermally equilibrated mixture of **3a**, the hypsochromic isomer is the major one. Integration of the multiplet at 6.72 ppm reveals, that it contains the signals of three protons. All three protons have to belong to the hypsochromic isomer, due to the value of the integral. The broad shape of the signal at 6.72 ppm differs from the sharp signals that is expected for a *para*-substituted aromatic compound. Therefore, it is estimated, that the H1 proton of the hypsochromic isomer is the third proton contributing to the signal at 6.72 ppm. Source data are provided as Source Data File.

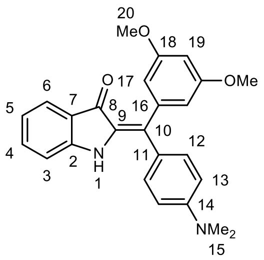

E-**3a**

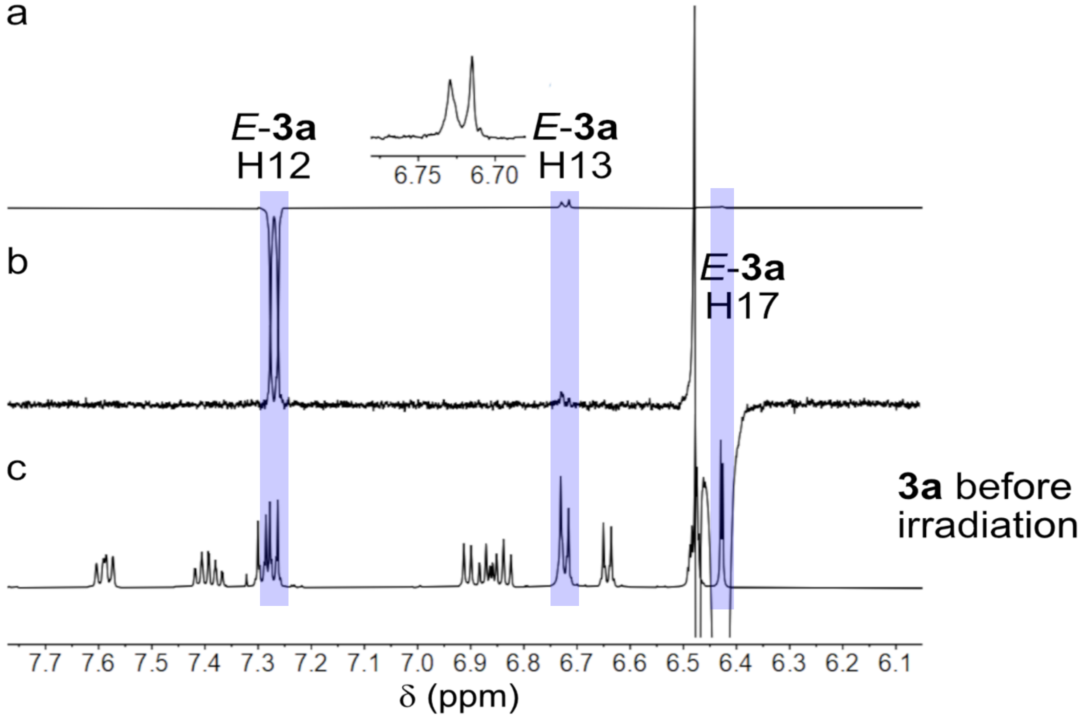

Supplementary Figure 16: 1D ^{1}H-^{1}H NOE NMR (601 MHz, CD_2Cl_2, 25 °C) experiment. **a** 1D ^{1}H-^{1}H NOE NMR spectrum showing that irradiating the H12 proton signal of the hypsochromic isomer at 7.28 ppm leads to signal increase of the H13 and H1 proton signals at 6.72 ppm. **b** 1D ^{1}H-^{1}H NOE NMR specrum showing that irradiating the H17 protons of the hypsochromic isomer at 6.43 ppm leads to signal increase of the H1 proton of the hypsochromic isomer at 6.72 ppm. The shape of the arising signal excludes the H13 proton signal. Taking the observations together, it can therefore be concluded that the hypsochromic isomer is the *E*-**3a** isomer. **c** ^{1}H NMR spectrum of a thermally equilibrated *E*/*Z* isomer mixture of **3a** shows an isomer ratio of 1.24 : 1 (H20 : H20’). Source data are provided as Source Data File.

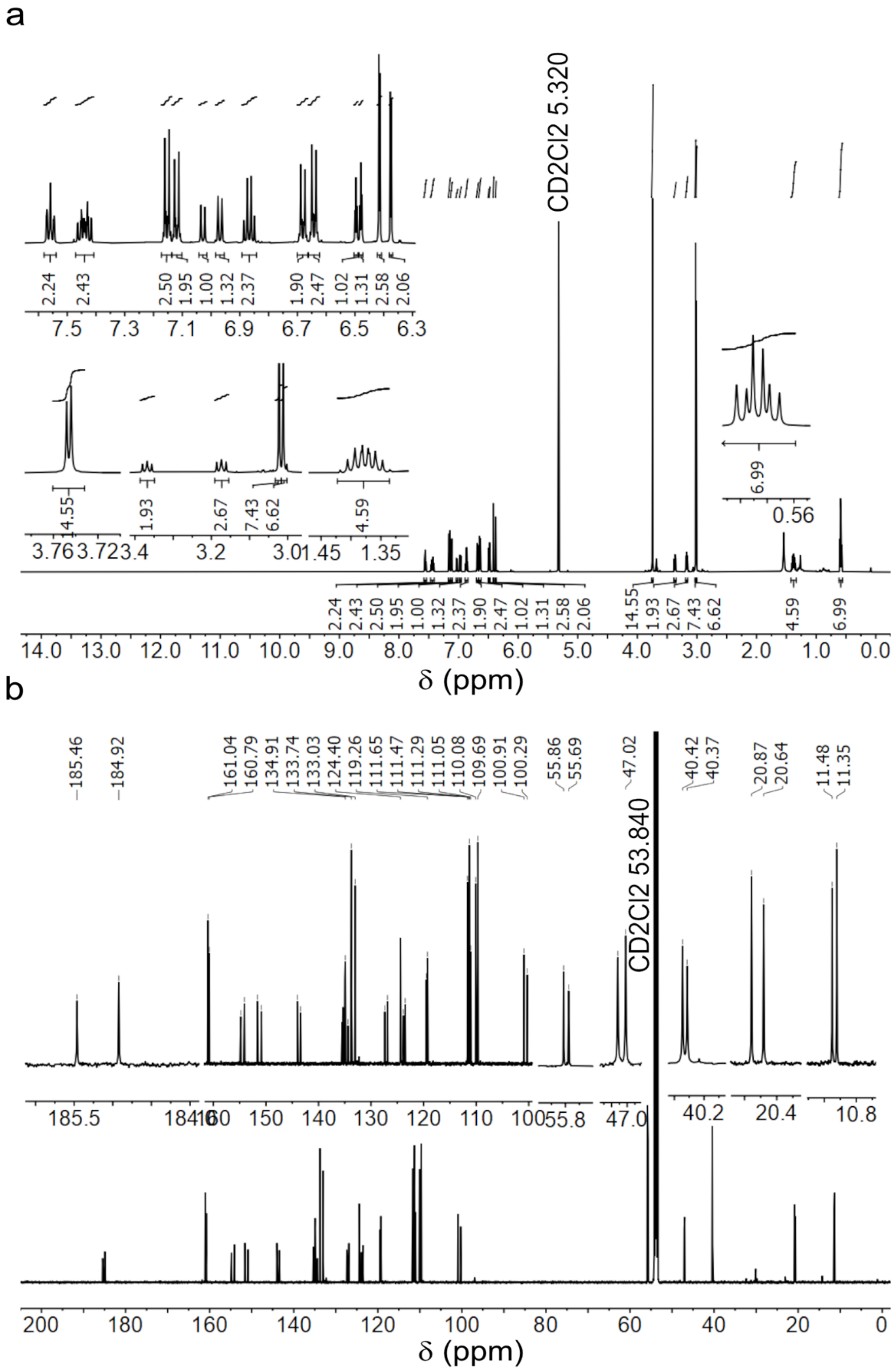

Supplementary Figure 17: NMR spectra of **3b** with isomer ratio of $E : Z = 1 : 1.3$. **a** ^{1}H NMR spectrum (601 MHz, CD_2Cl_2, 25 °C). **b** ^{13}C NMR spectrum (151 MHz, CD_2Cl_2, 25 °C). Source data are provided as Source Data File.

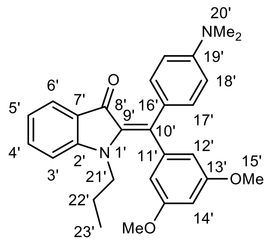

Z-**3b**

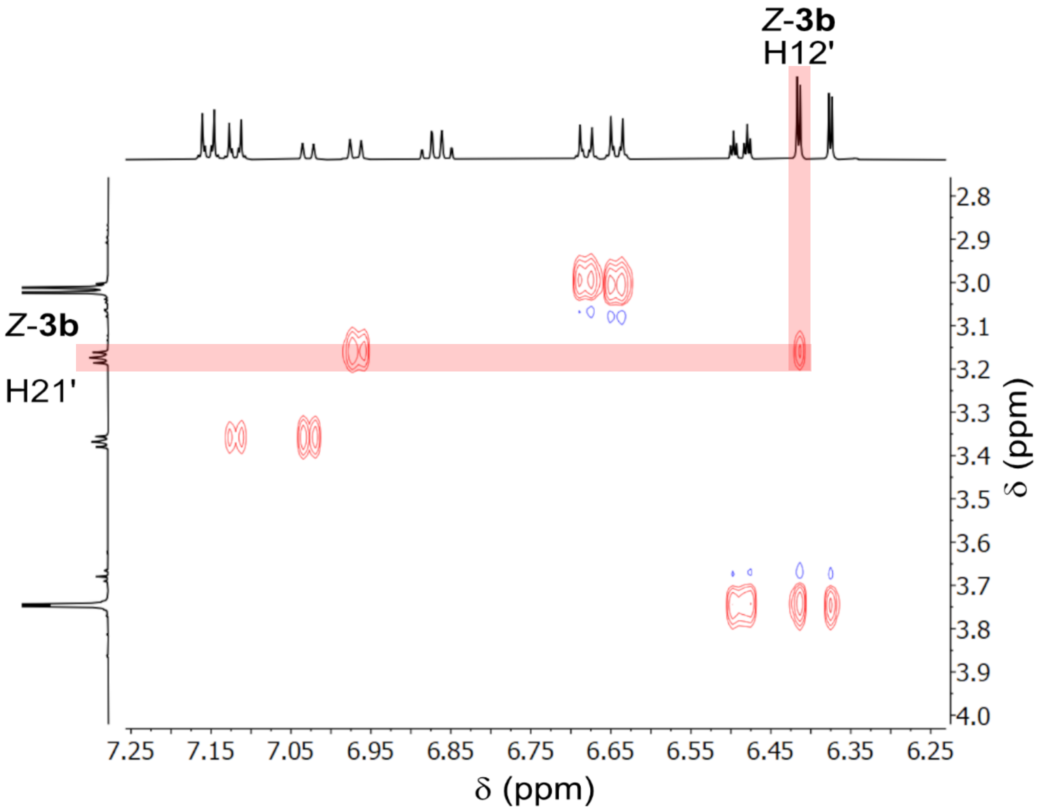

Supplementary Figure 18: Partial 2D ^{1}H-^{1}H NOESY NMR spectrum (601 MHz, CD_2Cl_2, 25 °C) of a *E*/*Z*-isomeric mixture (*E* : *Z* = 1 : 1.3) of **3b**. The cross signal between the protons at 6.42 ppm (H12' of *Z*-**3b**) and 3.17 ppm (H21' of *Z*-**3b**) confirms that the thermally more stable isomer is the bathochromic *Z* isomer. Source data are provided as Source Data File.

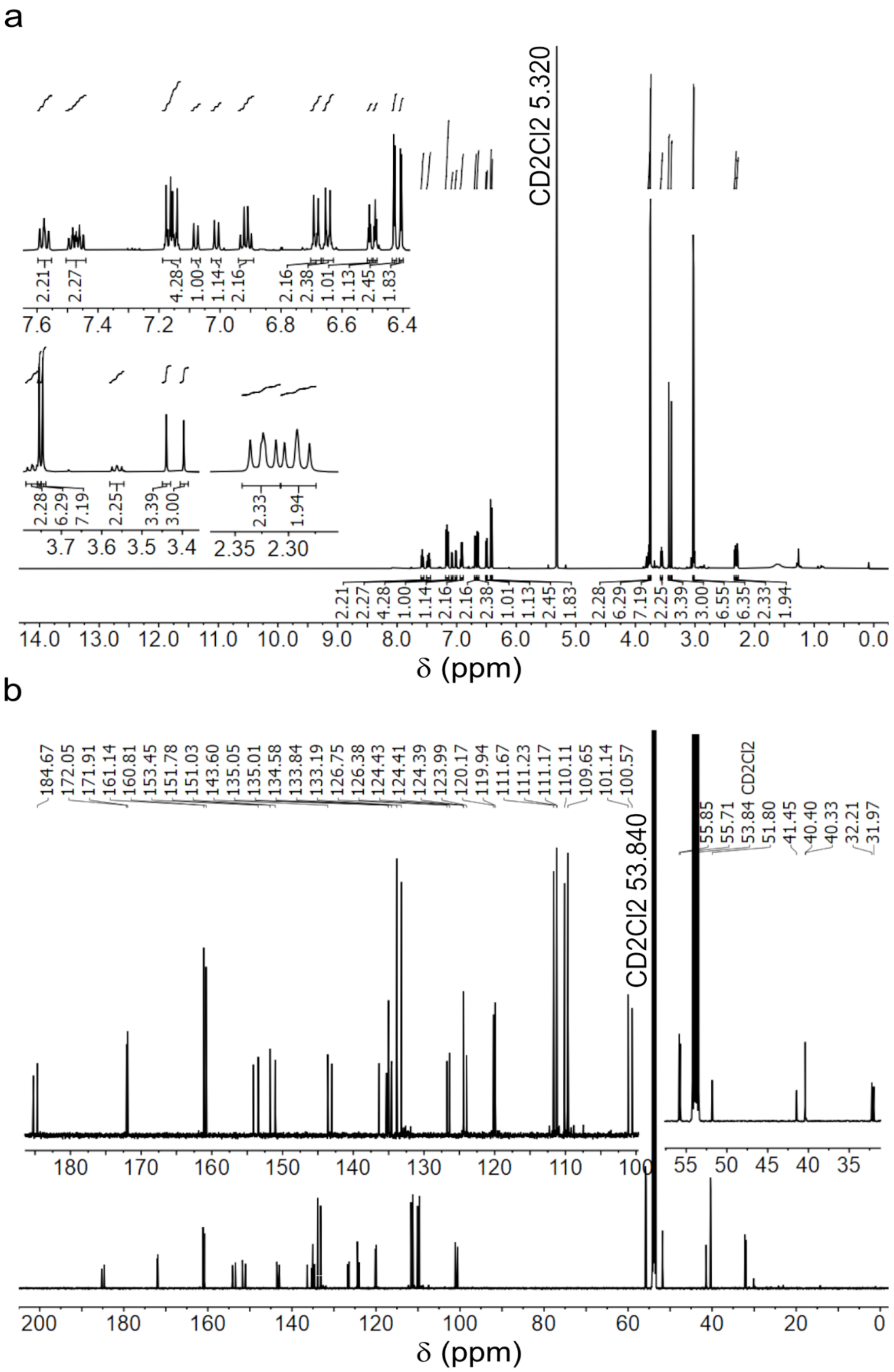

Supplementary Figure 19: NMR spectra of **3c** with isomer ratio of E : Z = 1 : 1. **a** ^{1}H NMR spectrum (601 MHz, CD_2Cl_2, 25 °C). **b** ^{13}C NMR spectrum (151 MHz, CD_2Cl_2, 25 °C). Source data are provided as Source Data File.

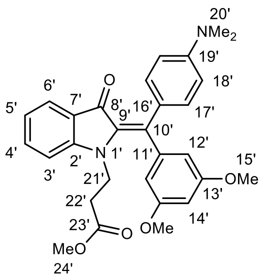

Z-**3c**

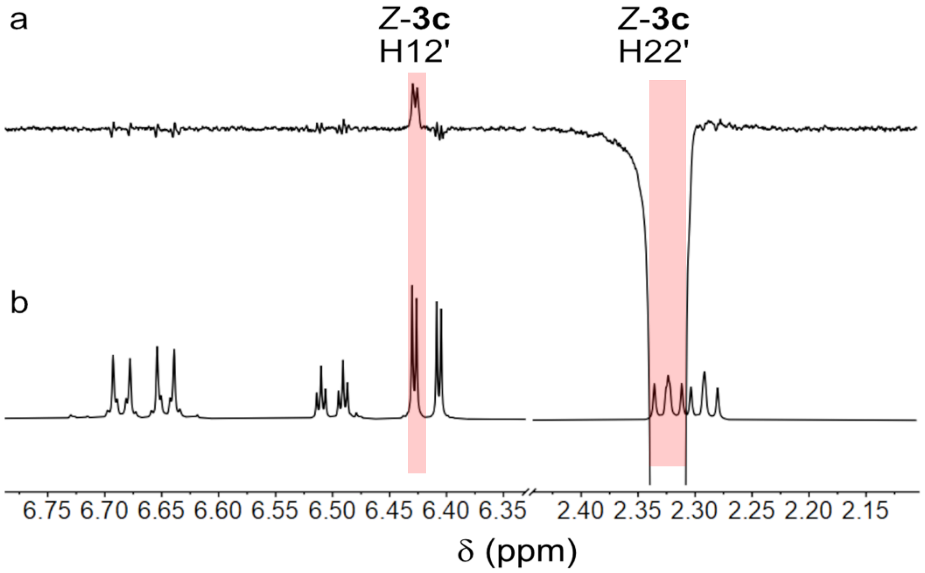

Supplementary Figure 20: 1D ^{1}H-^{1}H NOE NMR experiment of a *E*/*Z*-isomeric mixture (*E* : *Z* = 1 : 1.25) of **3c**. Irradiating the protons at 2.35 – 2.31 ppm (H22' of *Z*-**3c**) leads to signal increase at 6.43 ppm (H12' of *Z*-**3c**). This confirms that the thermally more stable isomer is the hypsochromic *Z* isomer. **a** 1D ^{1}H-^{1}H NOE NMR spectrum (601 MHz, CD_2Cl_2, 25 °C). **b** ^{1}H NMR spectrum (601 MHz, CD_2Cl_2, 25 °C). Source data are provided as Source Data File.

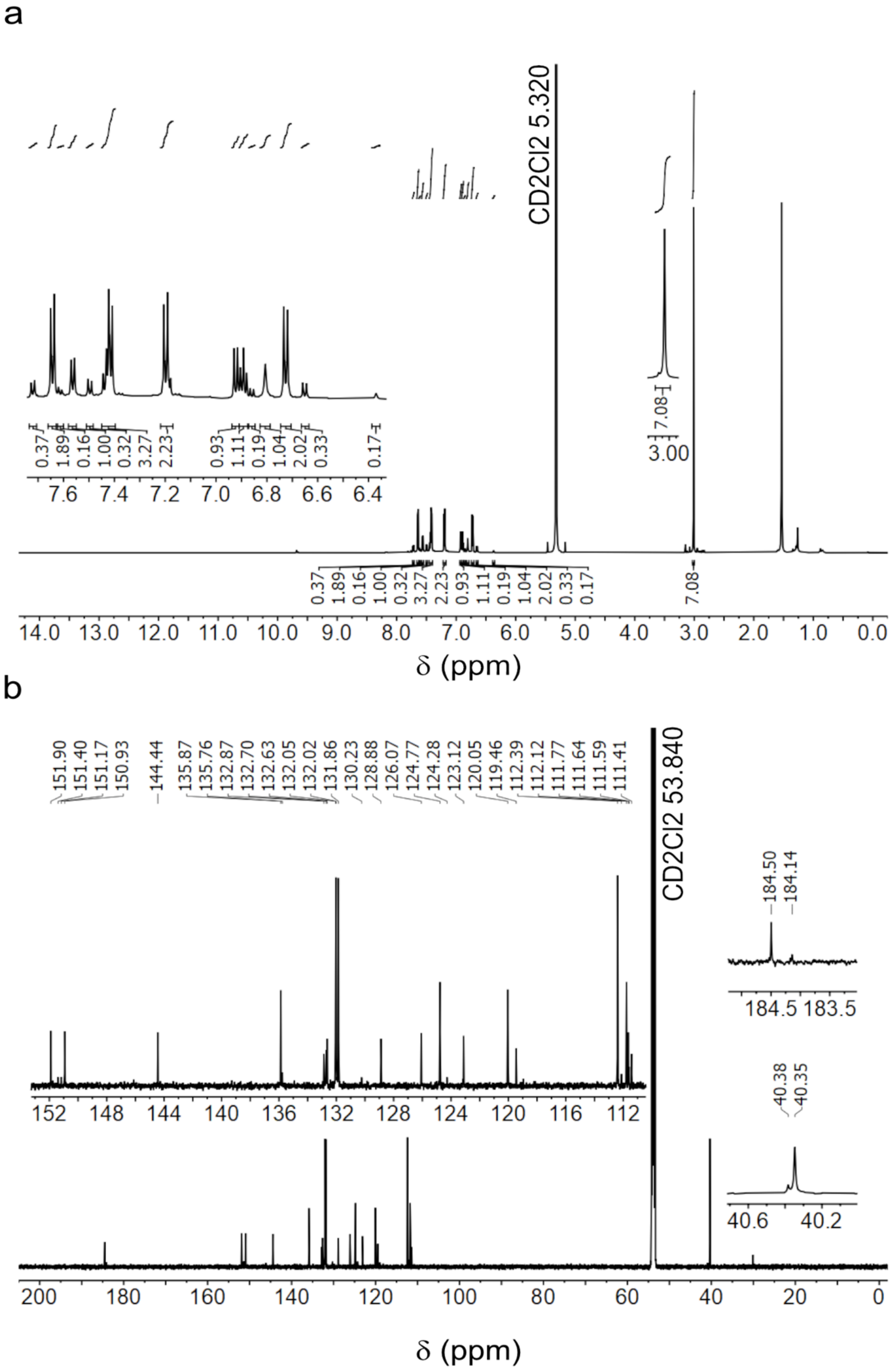

Supplementary Figure 21: NMR spectra of **4a** with isomer ratio of $E : Z = 1 : 5.1$. **a** ^{1}H NMR spectrum (601 MHz, CD_2Cl_2, 25 °C). **b** ^{13}C NMR spectrum (151 MHz, CD_2Cl_2, 25 °C). Source data are provided as Source Data File.

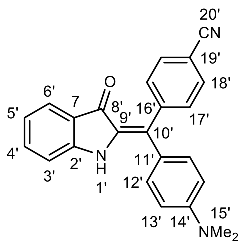

Z-**4a**

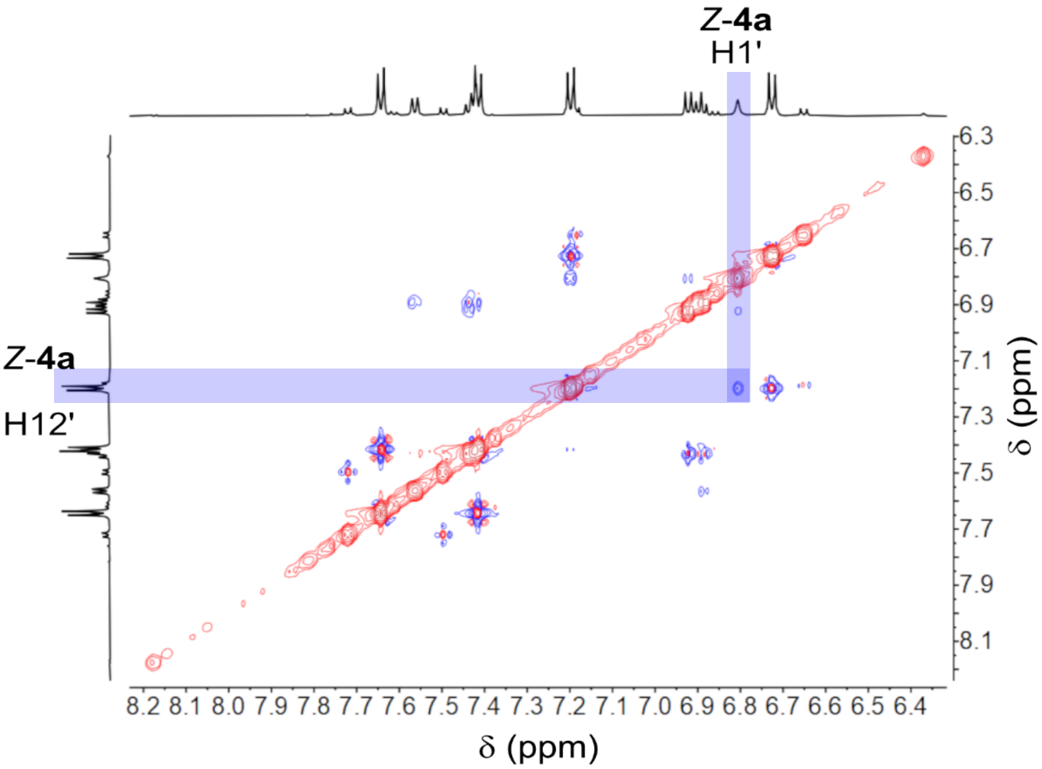

Supplementary Figure 22: Partial 2D ^{1}H-^{1}H NOESY NMR spectrum (601 MHz, CD_2Cl_2, 25 °C) of a *E*/*Z*-isomeric mixture (*E* : *Z* = 1 : 5.1) of **4a**. The cross signal between the protons at 7.20 ppm (H12' of *Z*-**4a**) and 6.81 ppm (H1' of *Z*-**4a**) confirms that the thermally more stable isomer is the hypsochromic *Z* isomer. Source data are provided as Source Data File.

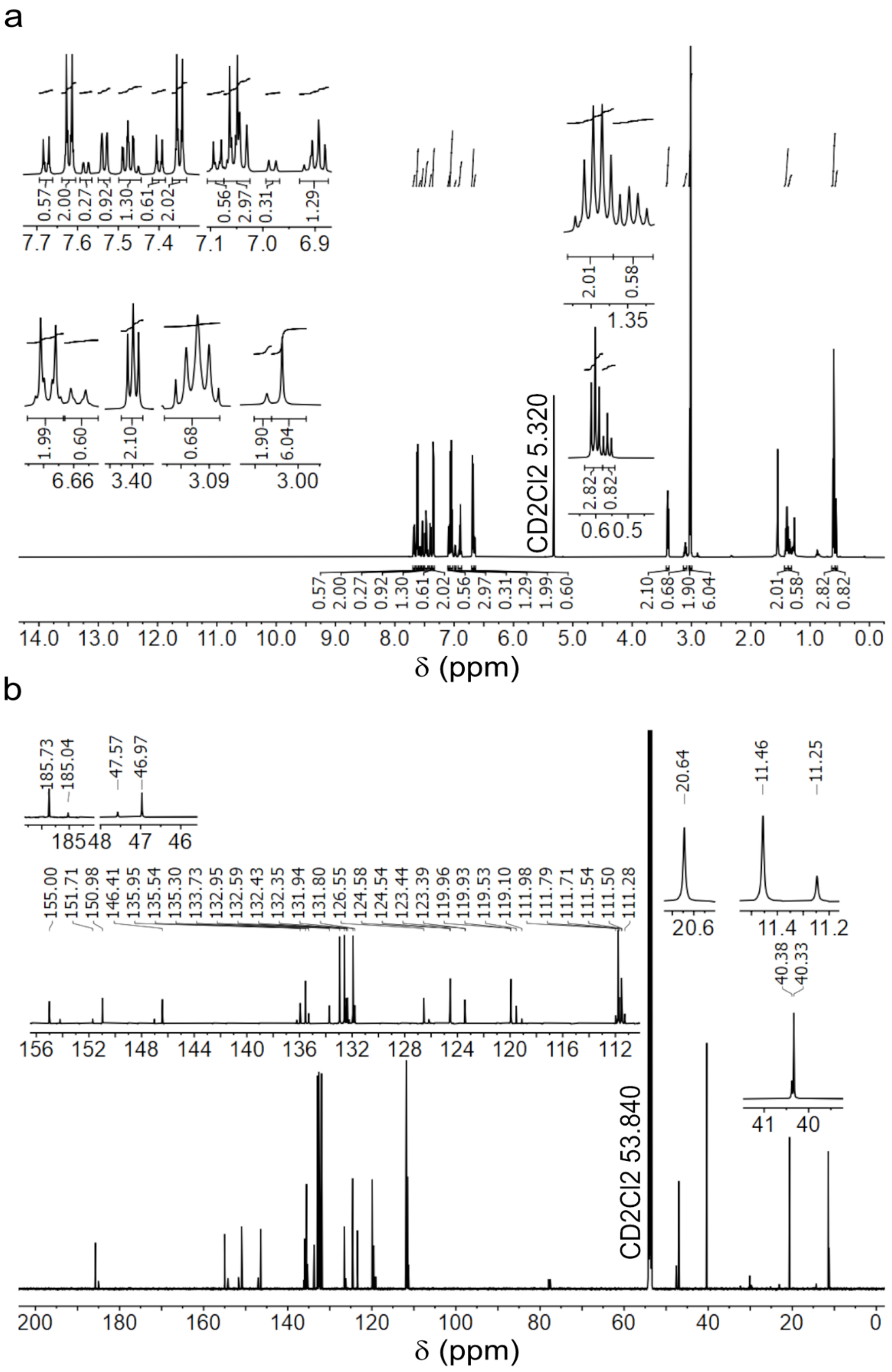

Supplementary Figure 23: NMR spectra of **4b** with isomer ratio of *E* : *Z* = 1 : 3.5. **a** ^{1}H NMR spectrum (601 MHz, CD_2Cl_2, 25 °C). **b** ^{13}C NMR spectrum (151 MHz, CD_2Cl_2, 25 °C). Source data are provided as Source Data File.

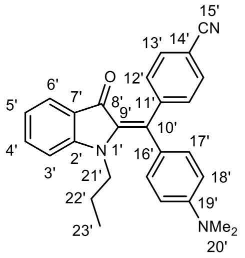

Z-**4b**

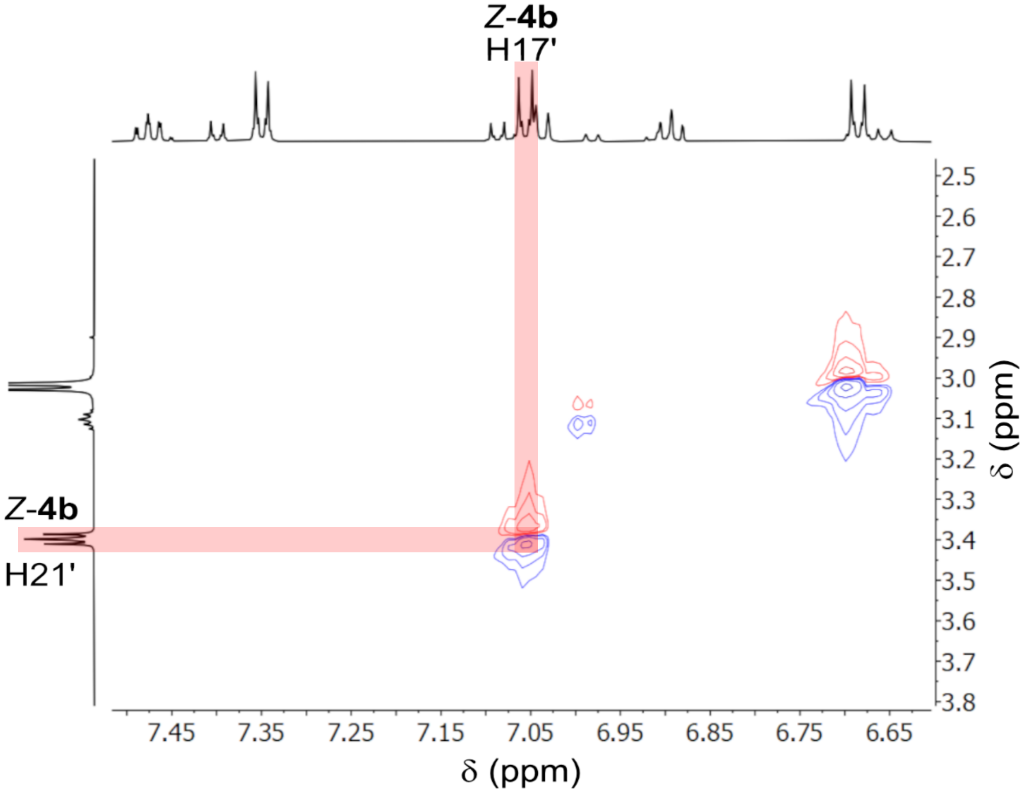

Supplementary Figure 24: Partial 2D ^{1}H-^{1}H NOESY NMR spectrum (601 MHz, CD_2Cl_2, 25 °C) of a *E*/*Z*-isomeric mixture (*E* : *Z* = 1 : 3.5) of **4b**. The cross signal between the protons at 7.06 ppm (H17' of *Z*-**4b**) and 3.40 ppm (H21' of *Z*-**4b**) confirms that the thermally more stable isomer is the bathochromic *Z* isomer. Source data are provided as Source Data File.

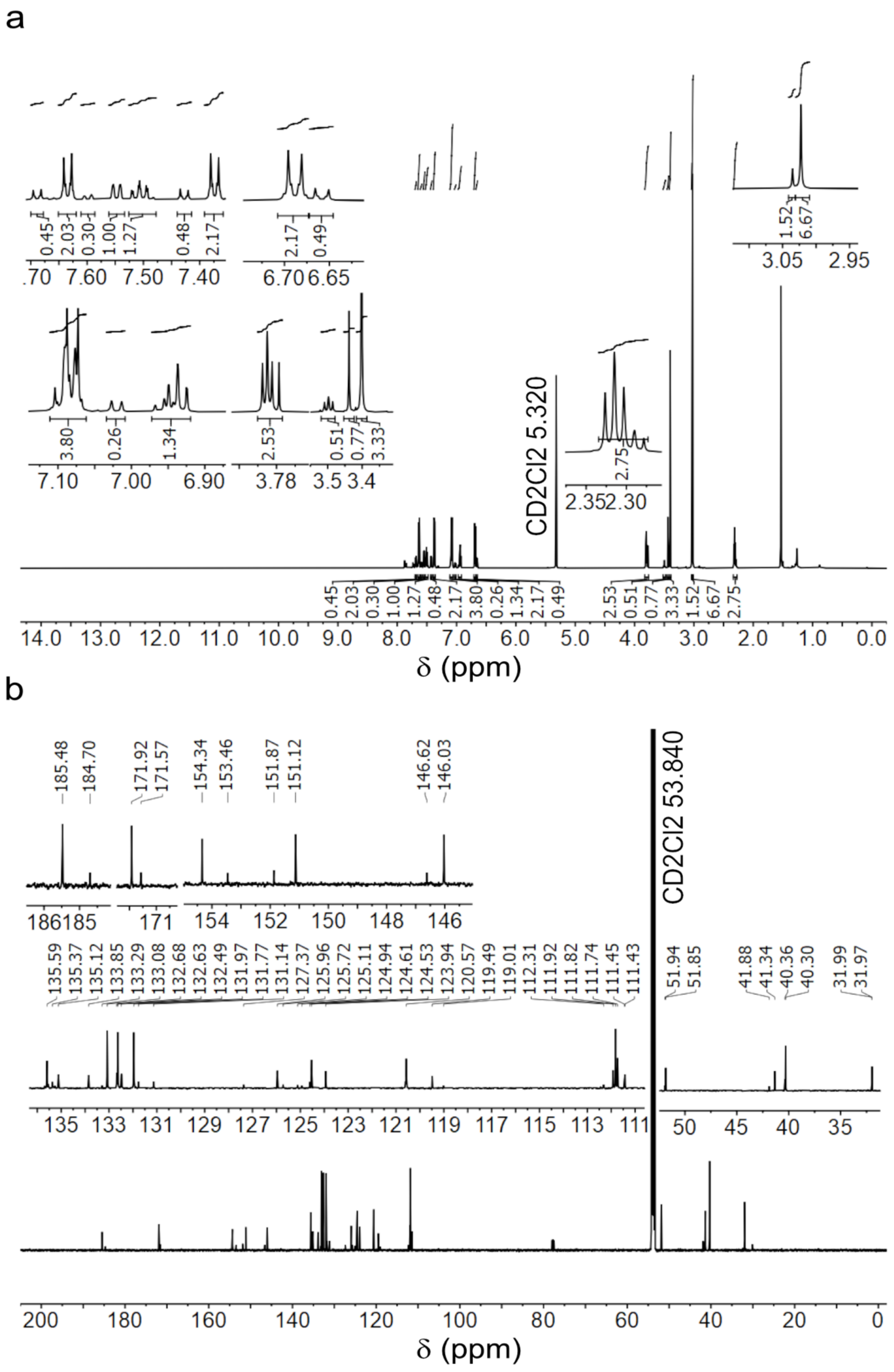

Supplementary Figure 25: NMR spectra of **4c** with isomer ratio of E : Z = 1 : 4.5. **a** ^{1}H NMR spectrum (601 MHz, CD_2Cl_2, 25 °C). **b** ^{13}C NMR spectrum (151 MHz, CD_2Cl_2, 25 °C). Source data are provided as Source Data File.

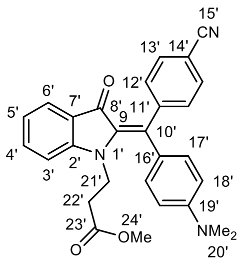

Z-**4c**

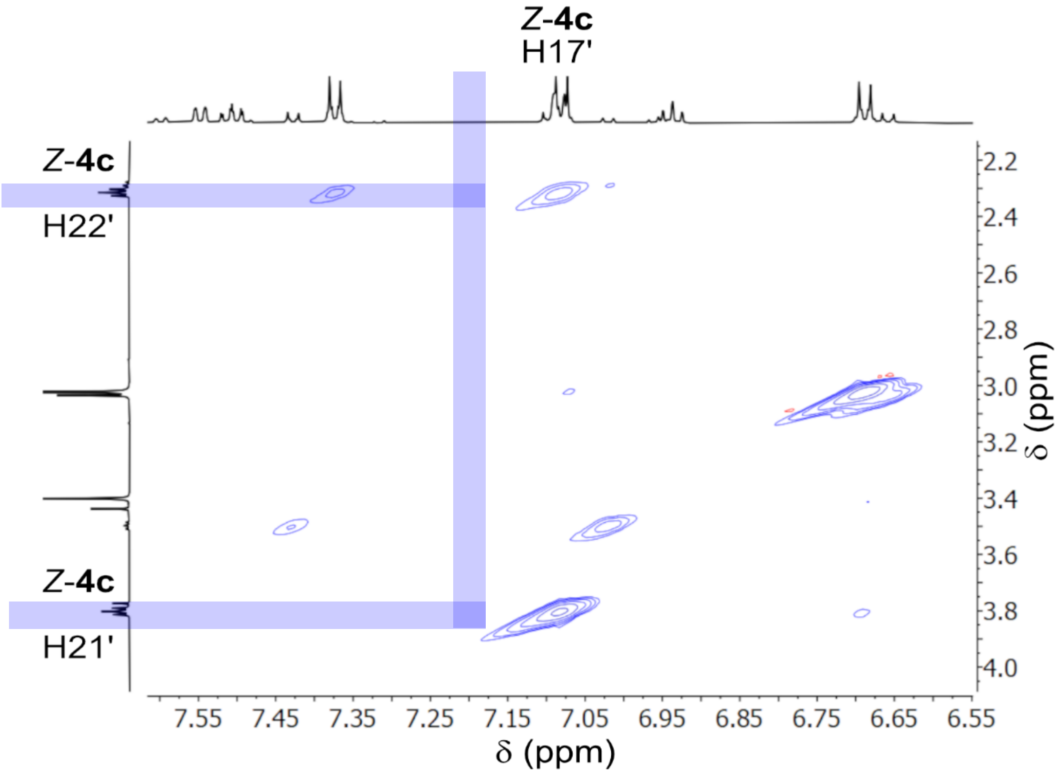

Supplementary Figure 26: Partial 2D ^{1}H-^{1}H NOESY NMR spectrum (601 MHz, CD_2Cl_2, 25 °C) of a *E*/*Z*-isomeric mixture (*E* : *Z* = 1 : 4.5) of **4c**. The cross signals between the protons at 7.11 – 7.07 ppm (H17’ of *Z*-**4c**) with those at 3.80 ppm (H21’ of *Z*-**4c**) and 2.31 (H22’ of *Z*-**4c**) confirm that the thermally more stable isomer is the hypsochromic *Z* isomer. Source data are provided as Source Data File.

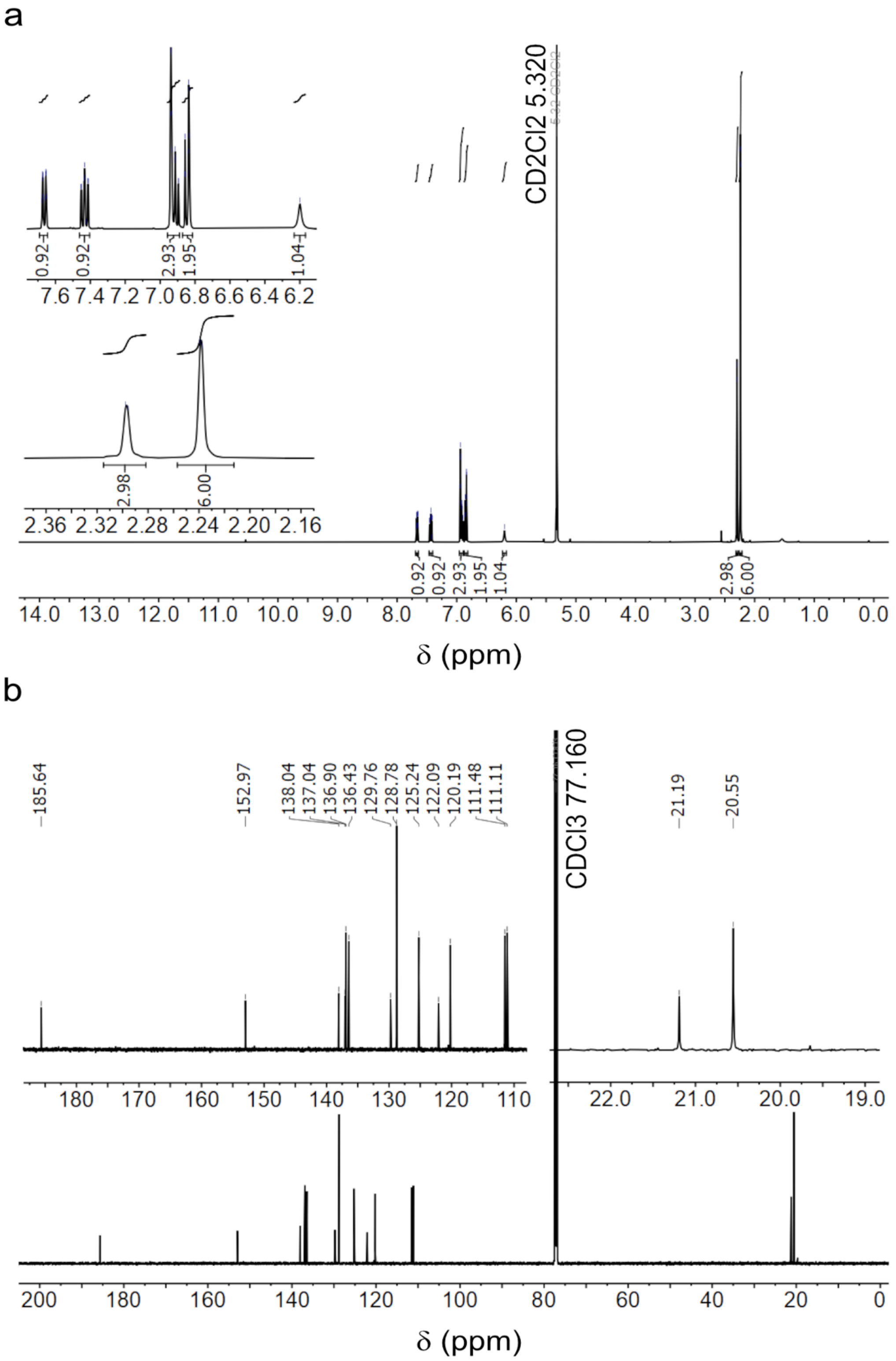

Supplementary Figure 27: NMR spectra of **5**. **a** ^{1}H NMR spectrum (400 MHz, CD_2Cl_2, 23 °C). **b** ^{13}C NMR spectrum (151 MHz, $CDCl_3$, 23 °C). Source data are provided as Source Data File.

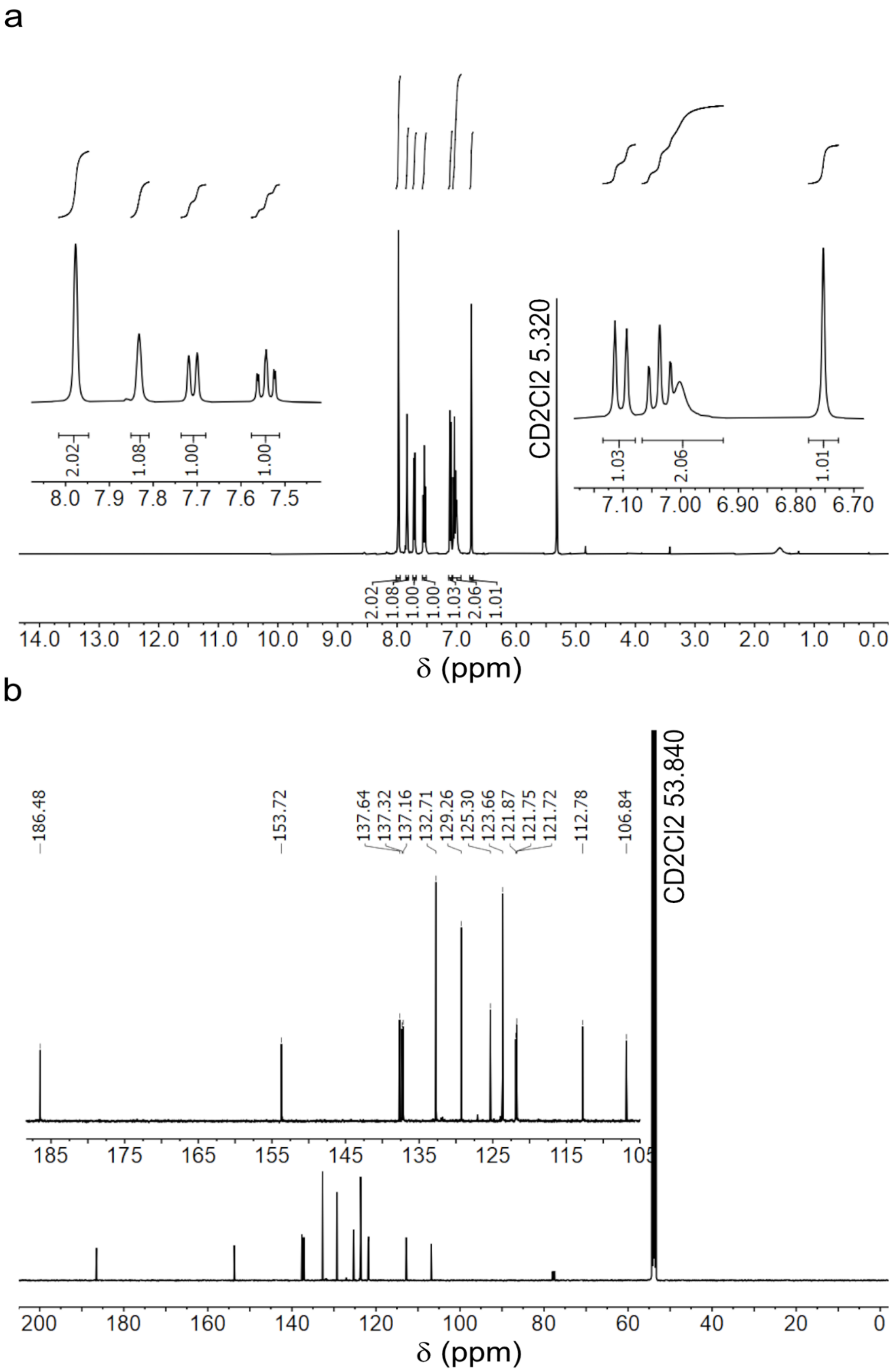

Supplementary Figure 28: NMR spectra of **6**. **a** ^{1}H NMR spectrum (400 MHz, CD_2Cl_2, 23 °C). **b** ^{13}C NMR spectrum (126 MHz, CD_2Cl_2, 23 °C). Source data are provided as Source Data File.

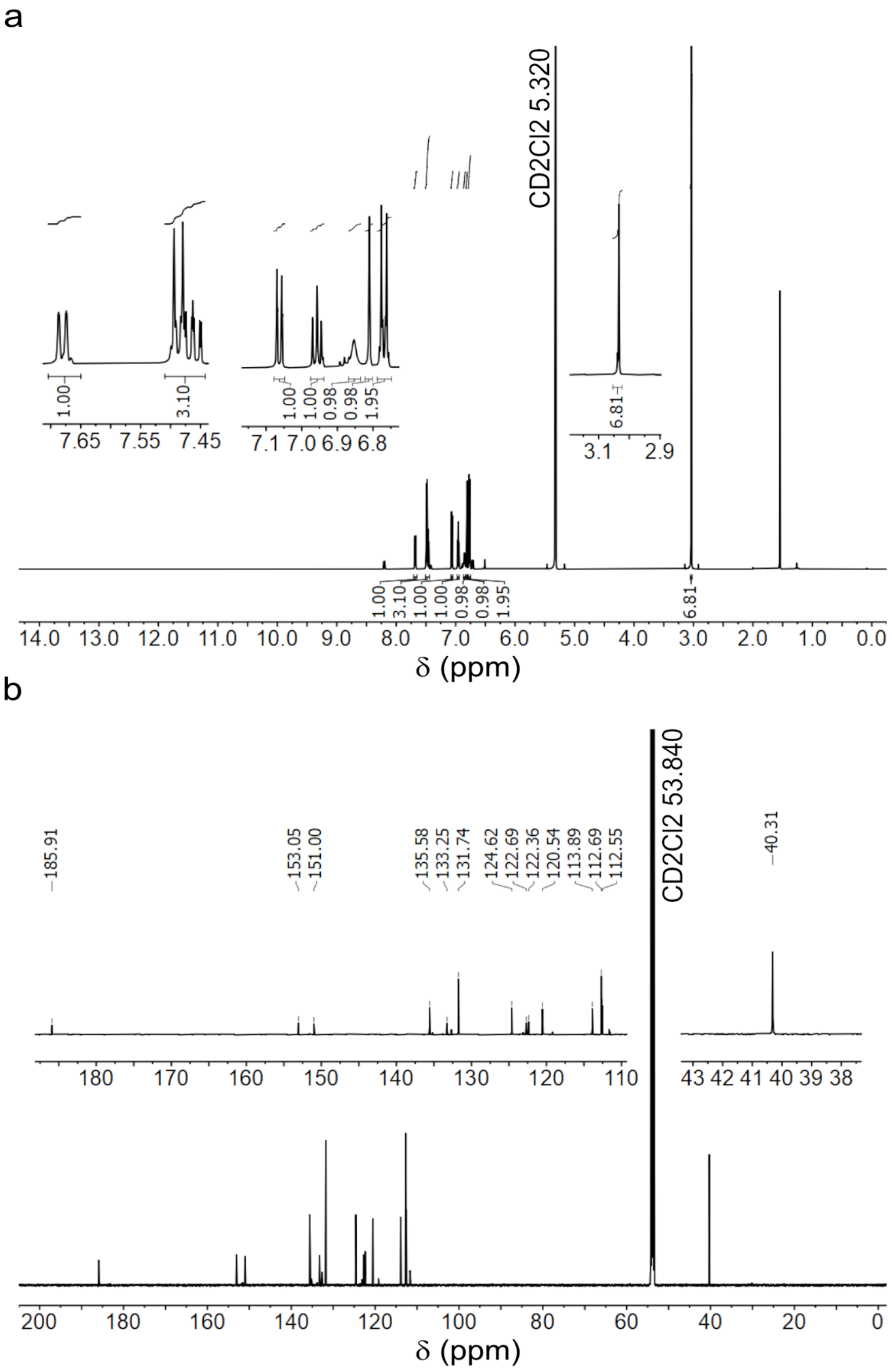

Supplementary Figure 29: NMR spectra of **7**. **a** ^{1}H NMR spectrum (601 MHz, CD_2Cl_2, 25 °C). **b** ^{13}C NMR spectrum (151 MHz, CD_2Cl_2, 25 °C). Source data are provided as Source Data File.

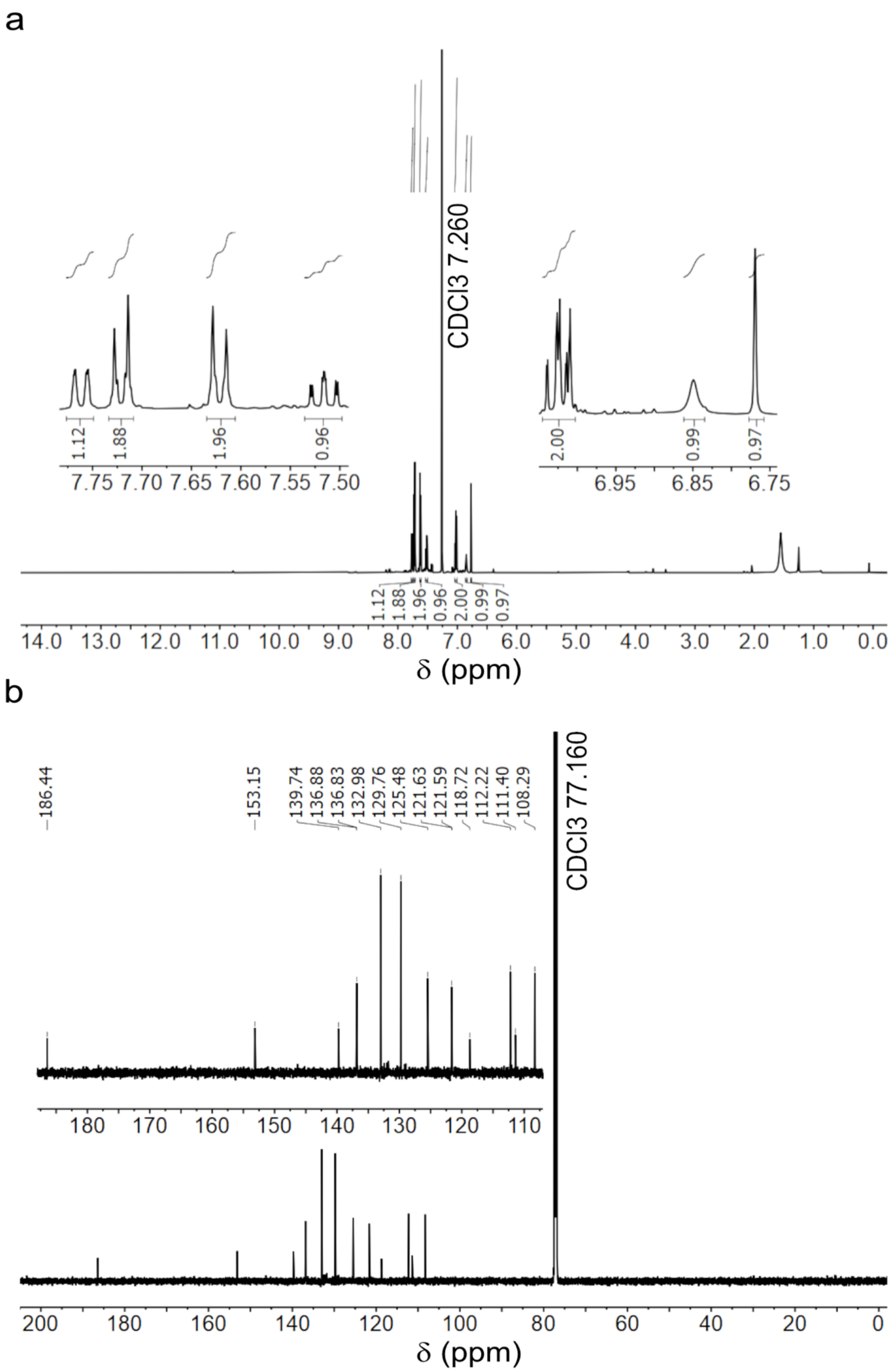

Supplementary Figure 30: NMR spectra of **8**. **a** ^{1}H NMR spectrum (601 MHz, $CDCl_3$, 25 °C). **b** ^{13}C NMR spectrum (151 MHz, $CDCl_3$, 25 °C). Source data are provided as Source Data File.

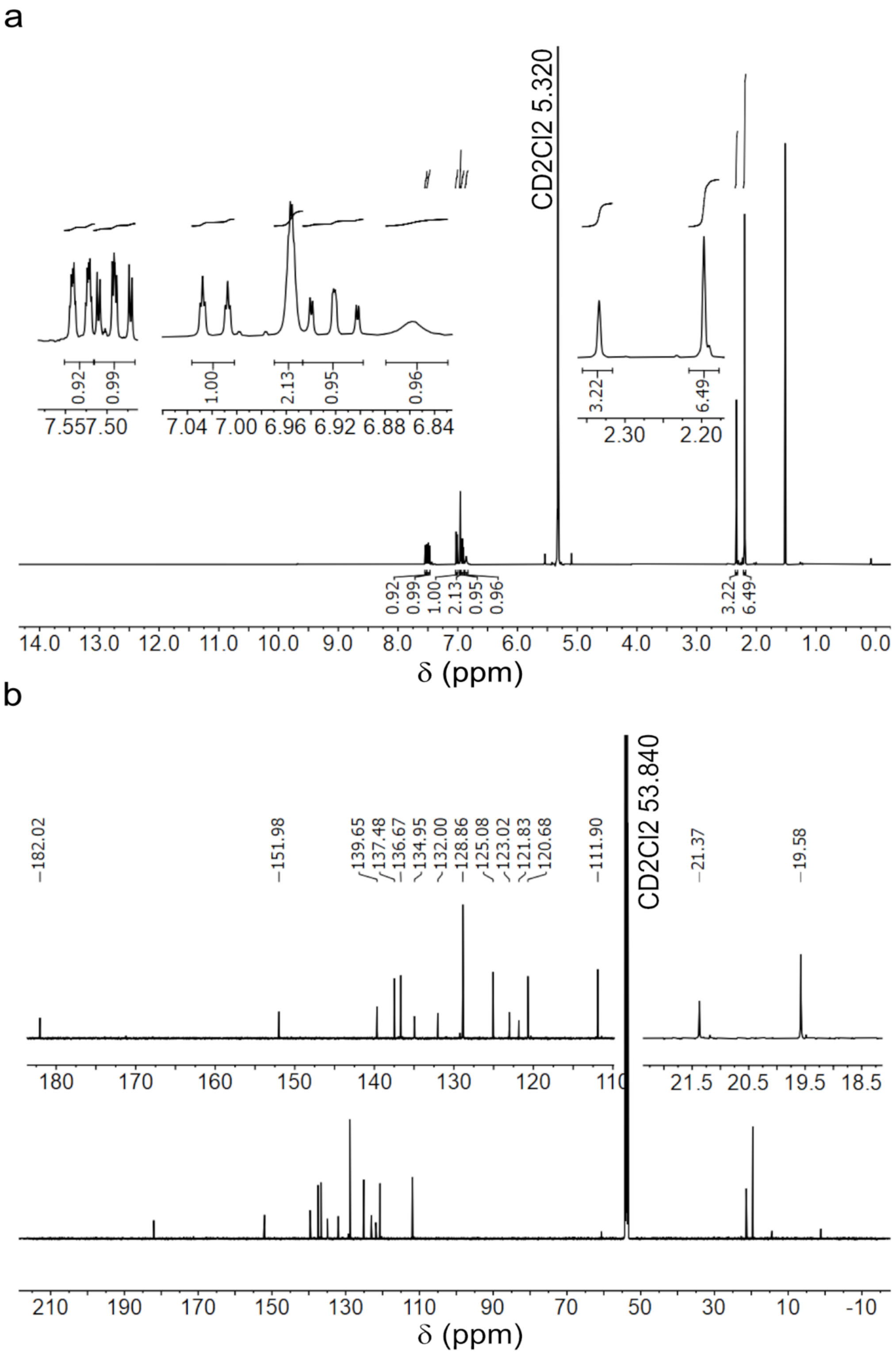

Supplementary Figure 31: NMR spectra of **9**. **a** ^{1}H NMR spectrum (400 MHz, CD_2Cl_2, 23 °C). **b** ^{13}C NMR spectrum (151 MHz, CD_2Cl_2, 23 °C). Source data are provided as Source Data File.

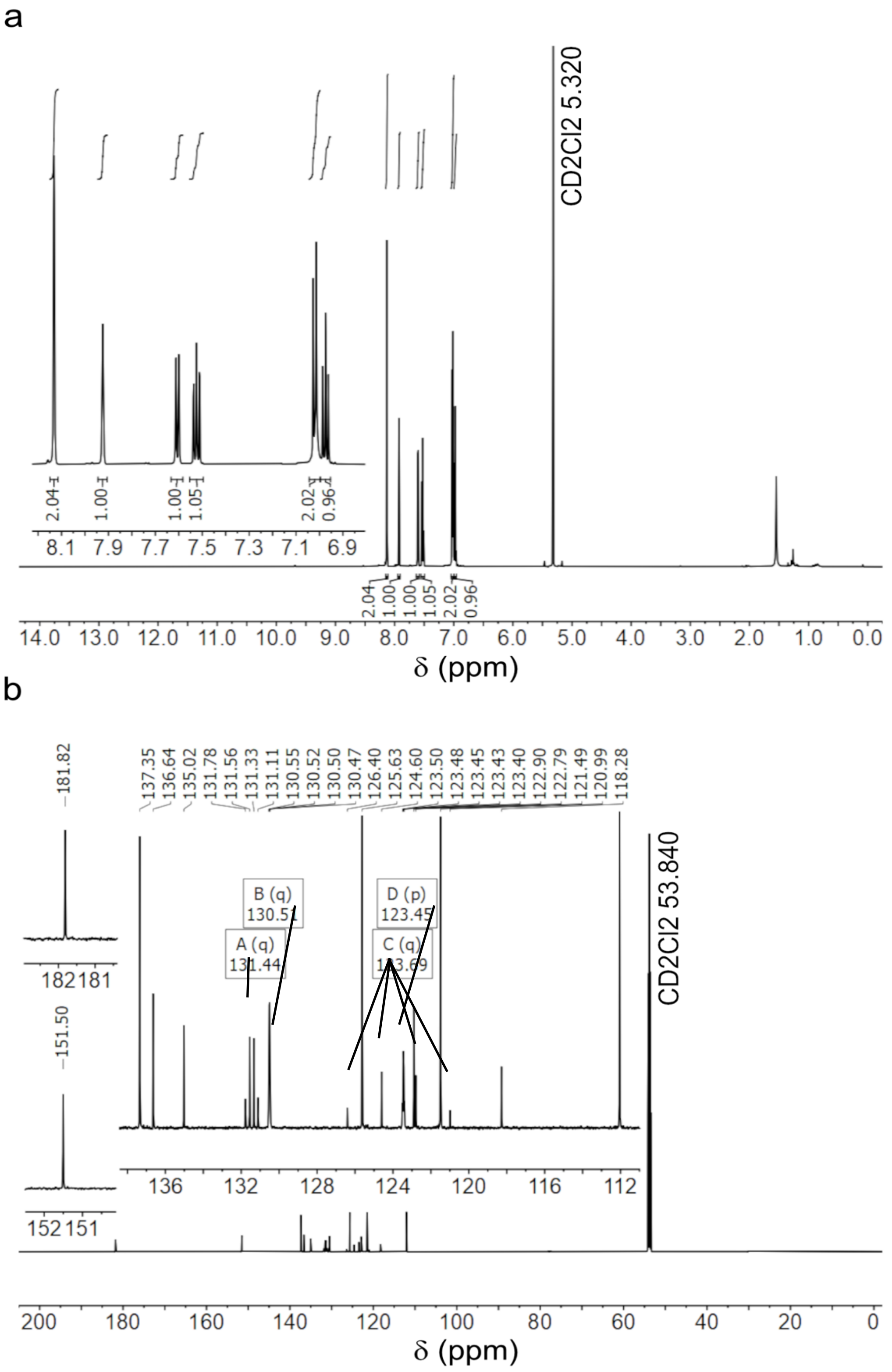

Supplementary Figure 32: NMR spectra of **10**. **a** ^{1}H NMR spectrum (601 MHz, CD_2Cl_2, 25 °C). **b** ^{13}C NMR spectrum (151 MHz, CD_2Cl_2, 25 °C). Source data are provided as Source Data File.

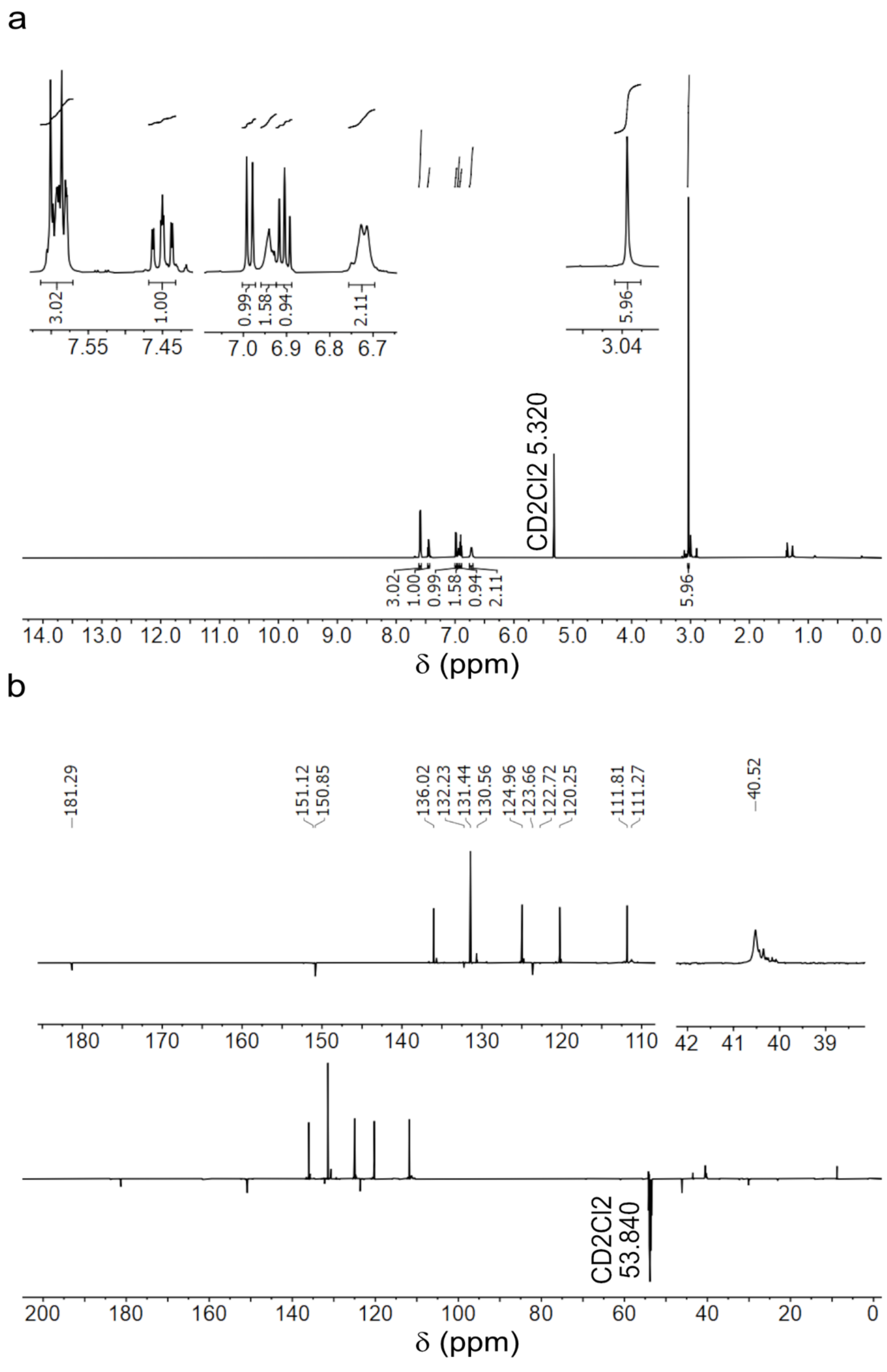

Supplementary Figure 33: NMR spectra of **11**. **a** ^{1}H NMR spectrum (601 MHz, CD_2Cl_2, 25 °C). **b** ^{13}C(DEPTq135) NMR spectrum (151 MHz, CD_2Cl_2, 25 °C). Source data are provided as Source Data File.

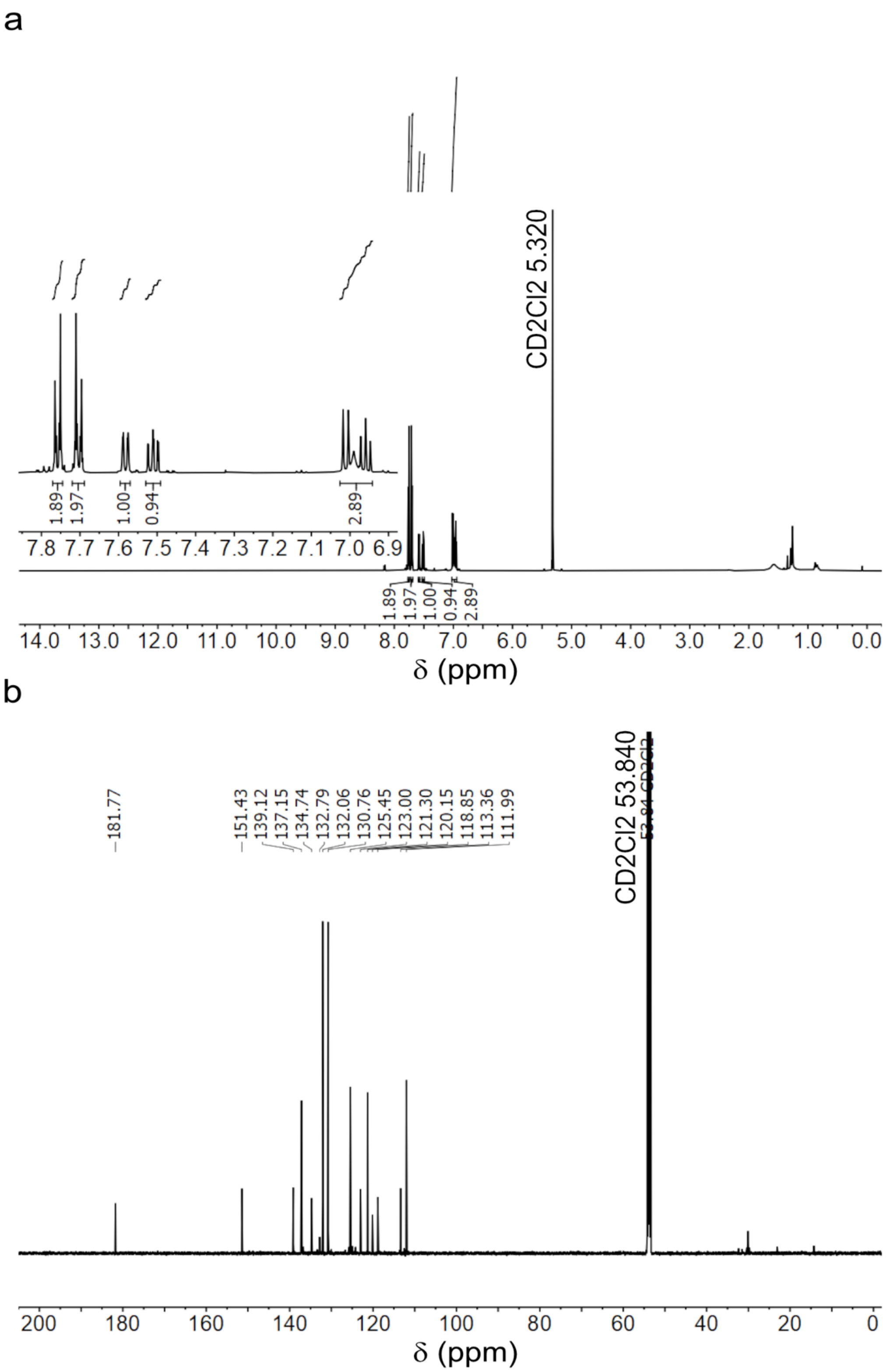

Supplementary Figure 34: NMR spectra of **12**. **a** ^{1}H NMR spectrum (601 MHz, CD_2Cl_2, 25 °C). **b** ^{13}C NMR spectrum (151 MHz, CD_2Cl_2, 25 °C). Source data are provided as Source Data File.

Supplementary Note 4: Determination of molar absorption coefficients of pure E and Z isomers

Samples of the respective diaryl-HI $E + Z$ isomer mixtures in toluene-d_8 solutions with known concentrations of 2×10^{-2} to 8×10^{-2} mol L^{-1} were irradiated with LEDs of 450 nm to 625 nm wavelength. The resulting isomeric compositions were determined by integration of suitable signals in the corresponding ^{1}H NMR spectra. Afterwards defined volumes between 10 to 180 µL were removed from the NMR tubes and diluted in 2.50 mL toluene (spectroscopic grade) inside a UV/Vis cuvette to obtain spectroscopic concentrations of 3×10^{-5} to 8×10^{-5} mol L^{-1}. Subsequently the corresponding UV/Vis spectra (of diaryl-HI mixtures with now known isomeric ratio) were recorded. This procedure was repeated at least three times for each sample. Hereupon, the positions of the isosbestic points of those consecutive UV/Vis measurements were compared to minimize dilution errors. This is necessary as the total concentration of the solution inside the UV/Vis cuvette has to be known and must not change for consecutive UV/Vis measurements of different isomeric compositions. Otherwise, the extrapolations of

(5) and (6) does not work.

The absorption spectra of the pure E and Z isomers are defined as absorbance as a function of wavelength and concentration according to (1) and (2).

$$A(E) = \mathrm{f}(\lambda_{E1,2,3\ldots}, [E_{1,2,3\ldots}]) \tag{1}$$

$$A(Z) = \mathrm{f}(\lambda_{Z1,2,3\ldots}, [Z_{1,2,3\ldots}]) \tag{2}$$

$A(E)$ = Absorbance of E isomer in abs. u. at certain wavelength λ in nm

$\mathrm{f}(\lambda_{E1,2,3\ldots}, [E_{1,2,3\ldots}])$ = function of wavelength λ in nm and concentration of the E isomer in mol L^{-1}

$A(Z)$ = Absorbance of Z isomer in abs. u. at certain wavelength λ in nm

$\mathrm{f}(\lambda_{Z1,2,3\ldots}, [Z_{1,2,3\ldots}])$ = function of wavelength λ in nm and concentration of the Z isomer in mol L^{-1}

The measured absorption spectra of $E + Z$ isomer mixtures, see Equation (3) and (4), then are a linear combination of pure absorption spectra of E and Z isomers multiplied with the

corresponding mole fractions, which were determined independently by ^{1}H NMR spectroscopy (percentage divided by 100).

$$A(E+) = A(E) \cdot x_{\mathrm{E+}}^{\mathrm{E}} + A(Z) \cdot x_{\mathrm{E+}}^{\mathrm{Z}} \tag{3}$$

$$A(Z+) = A(E) \cdot x_{\mathrm{Z+}}^{\mathrm{E}} + A(Z) \cdot x_{\mathrm{Z+}}^{\mathrm{Z}} \tag{4}$$

$A(E+)$ = Absorption spectra of an *E* isomer enriched solution containing both isomers

$A(Z+)$ = Absorption spectra of the *Z* isomer enriched solution containing both isomers

$x_{\mathrm{Z+}}^{\mathrm{Z}}$ = mole fraction of *Z* isomer in the *Z* enriched solution of *Z*+ divided by 100

$x_{\mathrm{Z+}}^{\mathrm{E}}$= mole fraction of *E* isomer in the *Z* enriched solution of *Z*+ divided by 100

$x_{\mathrm{E+}}^{\mathrm{Z}}$ = mole fraction of *Z* isomer in the *E* enriched solution of *E*+ divided by 100

$x_{\mathrm{E+}}^{\mathrm{E}}$= mole fraction of *E* isomer in the *E* enriched solution of *E*+ divided by 100

Combination of

(3) and (4) and expression of *A*(*Z*) by *A*(*E*) and vice versa leads to

(5) and (6). The hereby determined absorption spectra are the extrapolated absorption spectra of the pure *E* and pure *Z* isomer, respectively.

$$A(E) = \frac{A(Z+) \cdot x_{\mathrm{E+}}^{\mathrm{Z}} - A(E+) \cdot x_{\mathrm{Z+}}^{\mathrm{Z}}}{x_{\mathrm{E+}}^{\mathrm{Z}} \cdot x_{\mathrm{Z+}}^{\mathrm{E}} - x_{\mathrm{Z+}}^{\mathrm{Z}} \cdot x_{\mathrm{E+}}^{\mathrm{E}}} \tag{5}$$

$$A(Z) = \frac{A(Z+) \cdot x_{\mathrm{E+}}^{\mathrm{E}} - A(E+) \cdot x_{\mathrm{Z+}}^{\mathrm{E}}}{x_{\mathrm{Z+}}^{\mathrm{Z}} \cdot x_{\mathrm{E+}}^{\mathrm{E}} - x_{\mathrm{E+}}^{\mathrm{Z}} \cdot x_{\mathrm{Z+}}^{\mathrm{E}}} \tag{6}$$

Calculations of the molar absorption coefficients of pure *E* and *Z* isomers from the extrapolated pure *E* and *Z* isomer absorption spectra (as obtained from

(5) and (6), respectively) employ the LAMBERT-BEER law as described in Equation (7) and Equation (8).

$$A(E) = \log\left(\frac{I_0}{I_1}\right) = \varepsilon_E \cdot [E] \cdot d \tag{7}$$

$$A(Z) = \log\left(\frac{I_0}{I_1}\right) = \varepsilon_Z \cdot [Z] \cdot d \tag{8}$$

I_0 = intensity of incident light beam

I_1 = intensity of transmitted light beam

ε_E= molar absorption coefficient of *E* isomer in L mol^{-1} cm^{-1}

ε_Z= molar absorption coefficient of *Z* isomer in L mol^{-1} cm^{-1}

$[E]$= concentration of *E* isomer in mol L^{-1}

$[Z]$= concentration of *Z* isomer in mol L^{-1}

d = thickness of the UV/Vis cuvette (1 cm)

Therefore, the exact concentration of the respective *E* and *Z* isomer in solution is needed for the particular absorption spectrum of each pure isomer. An absorption spectrum of a solution containing both isomers with known total concentration [*E* + *Z*] is recorded. One of the fundamental boundary conditions for the here described experiment is constancy of the isosbestic points. At the wavelength at which the isosbestic point is observed the molar absorption coefficients as well as the absorption of both *E* and *Z* isomers is the same. Therefore, at this particular wavelength both previously determined absorption spectra of pure *E* and *Z* isomers can be scaled to the mixed spectrum of *E* + *Z* isomers with known concentration.

When absorption spectra extraction is done as described here the corresponding concentrations of pure *E* or *Z* isomer $[E]_{extra}$ or $[Z]_{extra}$ are inherently the same as the recorded total concentration of the *E* + *Z* mixture [*E*+*Z*] from that measurement, as described in (9).

$$[E + Z] \overset{\text{def}}{=} [E]_{\text{extra}} = [Z]_{\text{extra}} \tag{9}$$

$[E + Z]$ = total concentration of the *E*/*Z* mixture of the weighted sample in the UV/Vis cuvette in mol L^{-1}

$[E]_{\text{extra}}$= concentration of pure *E* isomer corresponding to the extrapolated *E* isomer absorption spectrum in the UV/Vis cuvette in mol L^{-1}

$[Z]_{\text{extra}}$= concentration of the pure *Z* isomer corresponding to the extrapolated *Z* isomer absorption spectrum in the UV/Vis cuvette in mol L^{-1}

Therefore, the molar absorption coefficients of pure *E* and *Z* isomers are calculated according to (10) and (11).

$$\varepsilon_{\text{E}} = \frac{A(E)}{[E+Z] \cdot d} \tag{10}$$

$$\varepsilon_{\text{Z}} = \frac{A(Z)}{[E+Z] \cdot d} \tag{11}$$

Supplementary Table 2: Maxima of molar absorption coefficients ε_{max} and corresponding wavelengths λ_{max} of the lowest energy absorption band of the pure hypsochromic and bathochromic isomers and related $\Delta\lambda_{max}$ and $\Delta\varepsilon_{max}$ values between the hypsochromic and bathochromic isomer in toluene solution at 23 °C.

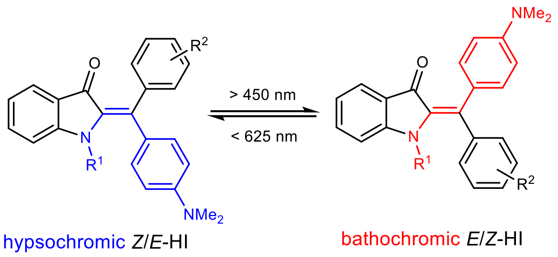

HI	ε_{max} of hypsochromic isomer (L mol^{-1} cm^{-1}) (stereoisomer)	λ_{max} of hypsochromic isomer (nm) (stereoisomer)	ε_{max} of bathochromic isomer (L mol^{-1} cm^{-1}) (stereoisomer)	λ_{max} of bathochromic isomer (nm) (stereoisomer)	$\Delta\lambda_{max}$ (nm) and $\Delta\varepsilon_{max}$ (L mol^{-1} cm^{-1}) between hypsochromic and bathochromic isomer
**1a**	15300 (*E*)	477 (*E*)	12200 (*Z*)	518 (*Z*)	41; 3100
**1b**	7700 (*E*)	499 (*E*)	7700 (*Z*)	539 (*Z*)	40; 0
**1c**	9600 (*E*)	493 (*E*)	7600 (*Z*)	534 (*Z*)	41; 2000
**2a**	-^{A}	-^{A}	-^{A}	-^{A}	-^{A}
**2b**	9600 (*E*)	518 (*E*)	8300 (*Z*)	563 (*Z*)	45; 1300
**2c**	6000 (*E*)	507 (*E*)	5100 (*Z*)	562 (*Z*)	55; 900
**3a**	5900 (*E*)	477 (*E*)	4700 (*Z*)	523 (*Z*)	46; 1200
**3b**	9700 (*E*)	512 (*E*)	9000 (*Z*)	542 (*Z*)	30; 700
**3c**	8800 (*E*)	503 (*E*)	7300 (*Z*)	541 (*Z*)	38; 1500
**4a**	-^{A}	-^{A}	-^{A}	-^{A}	-^{A}
**4b**	7100 (*Z*)	517 (*Z*)	6500 (*E*)	561 (*E*)	44; 600
**4c**	4100 (*Z*)	507 (*Z*)	3600 (*E*)	560 (*E*)	53; 500

A: not detected due to photodegradation by irradiation with light.

4.1 Molar absorption coefficient of diaryl-HI 1a

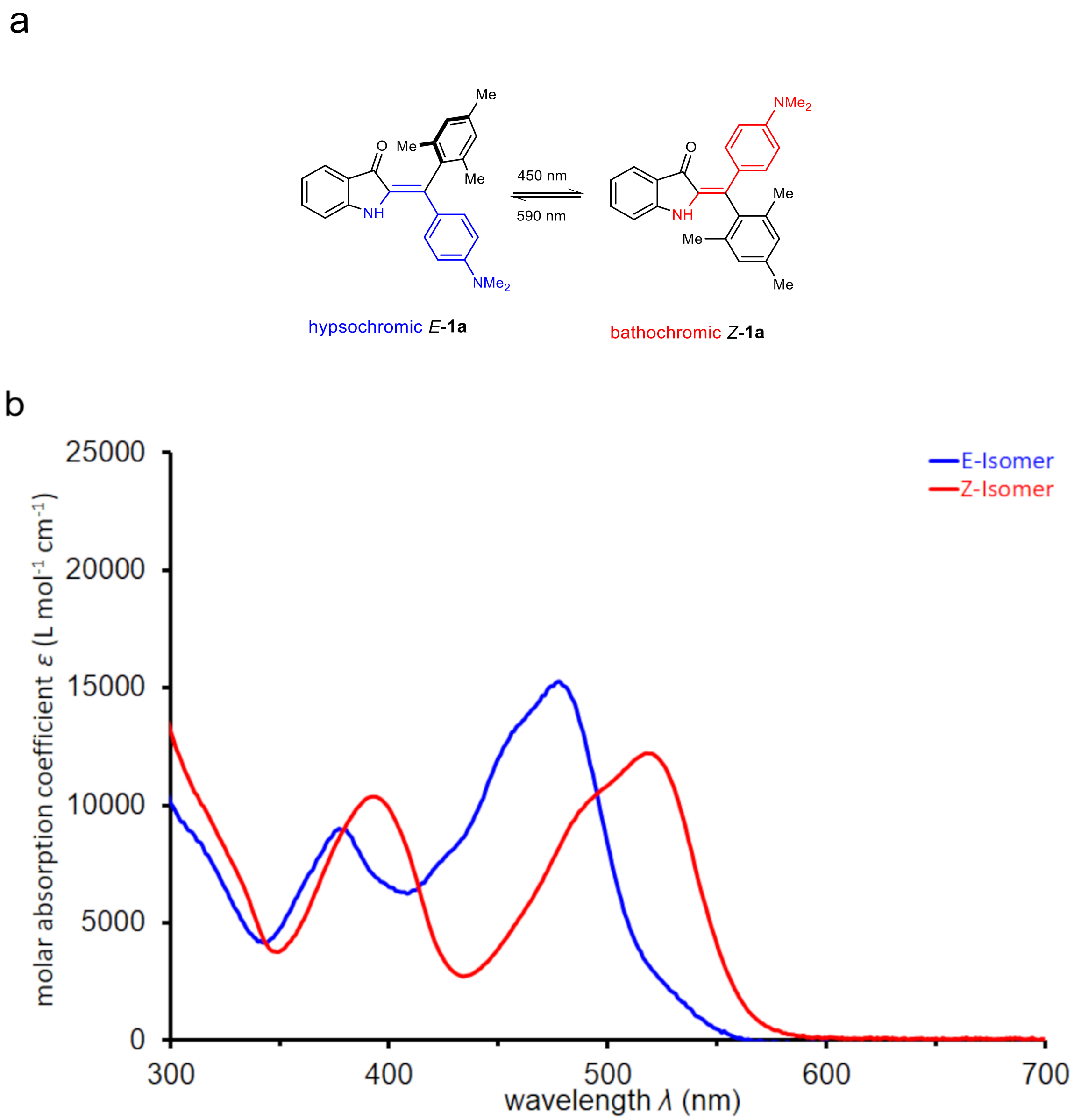

Supplementary Figure 35: a Schematic illustration of the photoisomerization from the hypsochromic (marked blue) to the bathochromic (marked red) diaryl-HI isomers of **1a** and vice versa. **b** Experimentally determined molar absorption coefficients of the pure *E* (marked blue) and *Z* (marked red) isomers of diaryl-HI **1a** in toluene solution at 23 °C. Source data are provided as Source Data File.

4.2 Molar absorption coefficient of diaryl-HI 1b

a

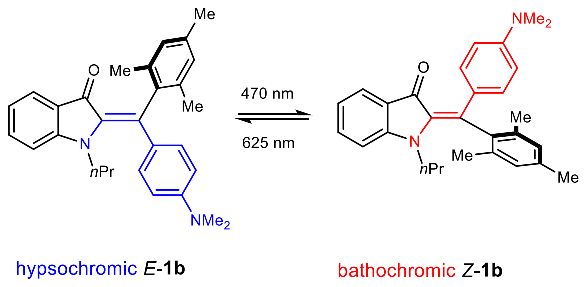

b

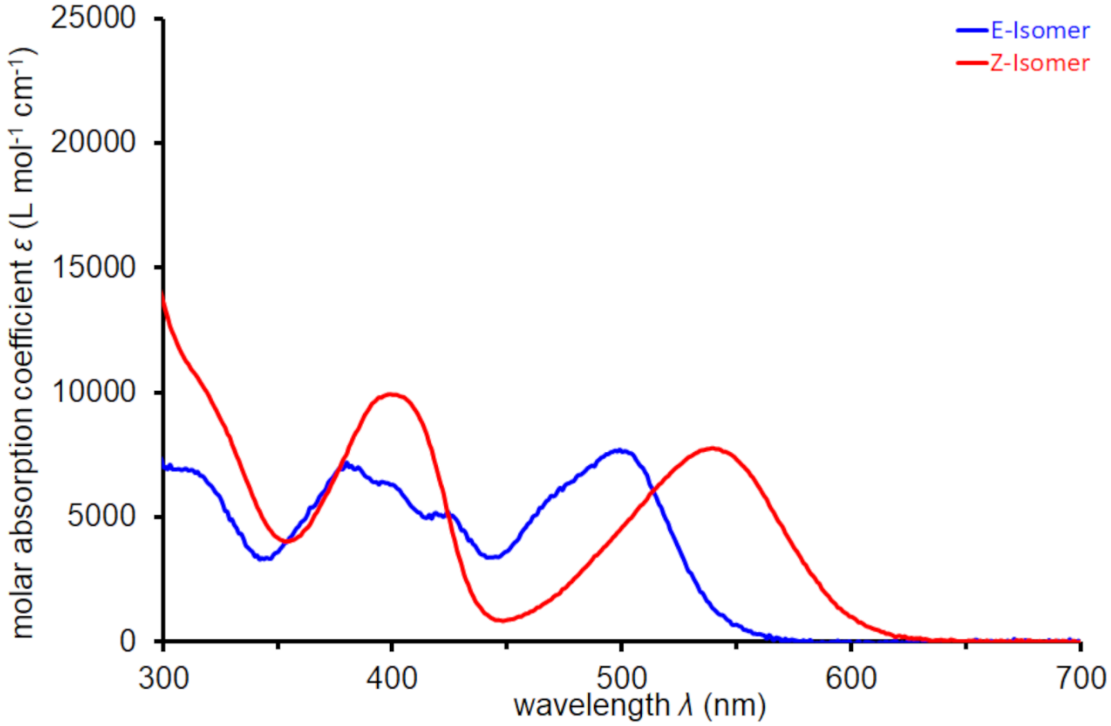

Supplementary Figure 36: a Schematic illustration of the photoisomerization from the hypsochromic (marked blue) to the bathochromic (marked red) diaryl-HI isomers of **1b** and vice versa. **b** Experimentally determined molar absorption coefficients of the pure *E* (marked blue) and *Z* (marked red) isomers of diaryl-HI **1b** in toluene solution at 23 °C. Source data are provided as Source Data File.

4.3 Molar absorption coefficient of diaryl-HI 1c

a

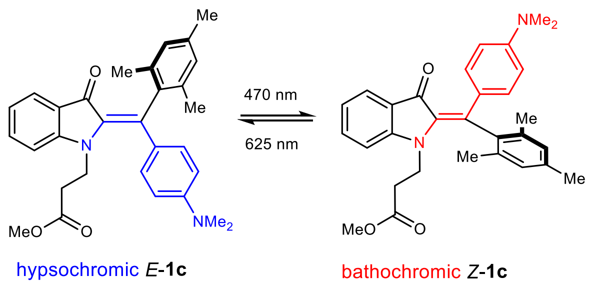

b

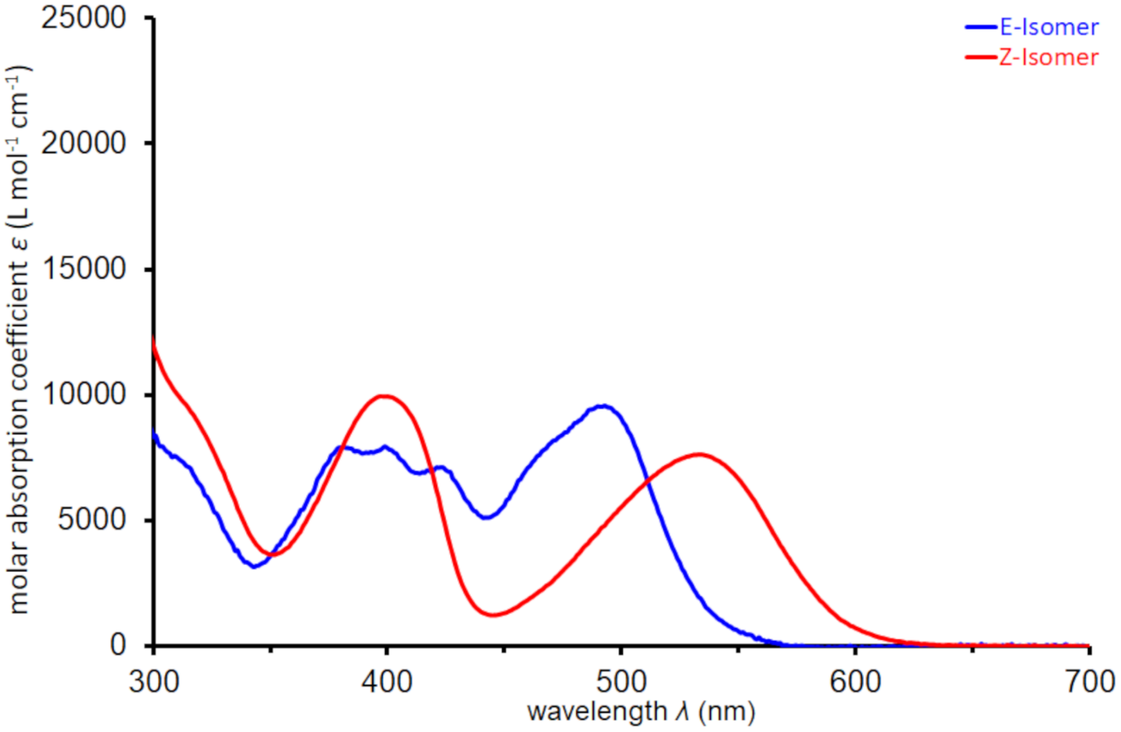

Supplementary Figure 37: **a** Schematic illustration of the photoisomerization from the hypsochromic (marked blue) to the bathochromic (marked red) diaryl-HI isomers of **1c** and vice versa. **b** Experimentally determined molar absorption coefficients of the pure *E* (marked blue) and *Z* (marked red) isomers of diaryl-HI **1c** in toluene solution at 23 °C. Source data are provided as Source Data File.

4.4 Molar absorption coefficient of diaryl-HI 2b

a

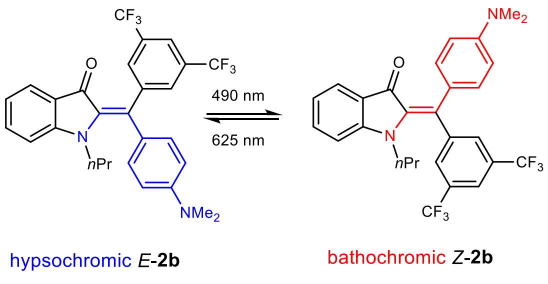

b

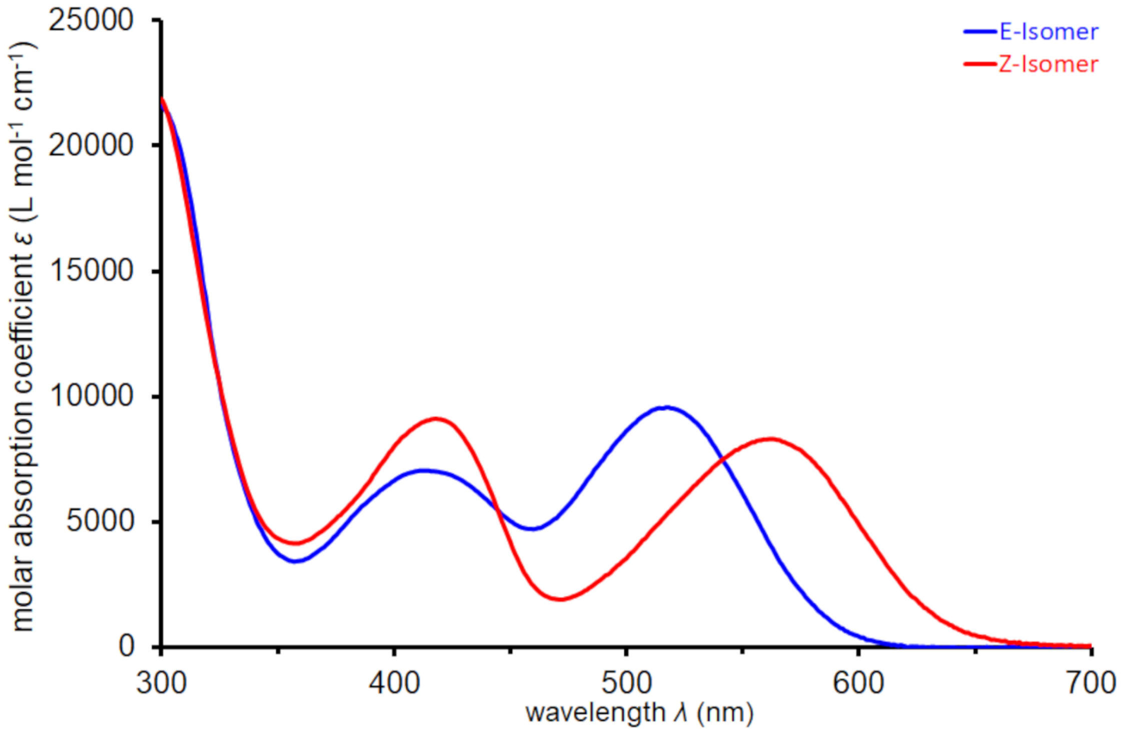

Supplementary Figure 38: a Schematic illustration of the photoisomerization from the hypsochromic (marked blue) to the bathochromic (marked red) diaryl-HI isomers of **2b** and vice versa. **b** Experimentally determined molar absorption coefficients of the pure *E* (marked blue) and *Z* (marked red) isomers of diaryl-HI **2b** in toluene solution at 23 °C. Source data are provided as Source Data File.

4.5 Molar absorption coefficient of diaryl-HI 2c

a

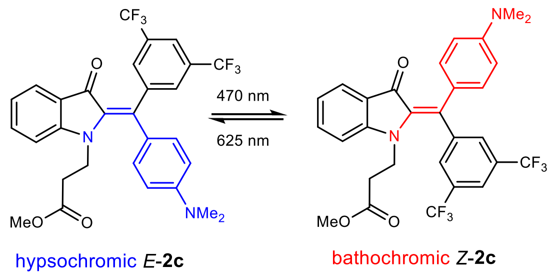

b

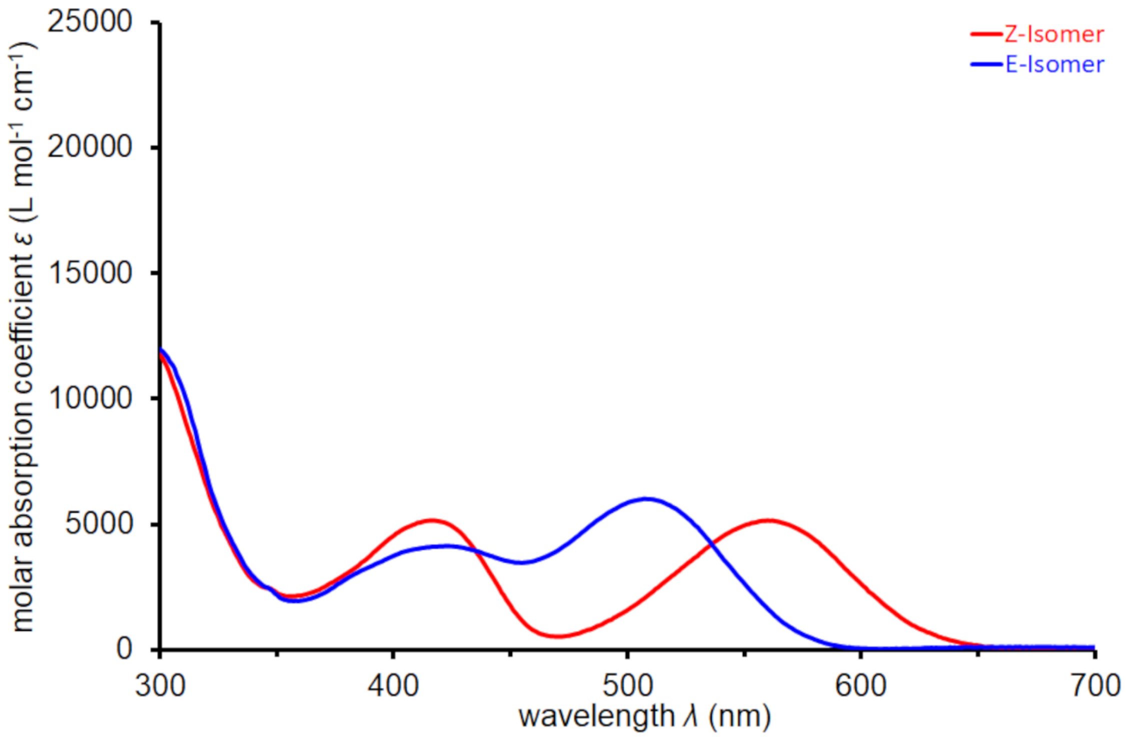

Supplementary Figure 39: a Schematic illustration of the photoisomerization from the hypsochromic (marked blue) to the bathochromic (marked red) diaryl-HI isomers of **2c** and vice versa. **b** Experimentally determined molar absorption coefficients of the pure *E* (marked blue) and *Z* (marked red) isomers of diaryl-HI **2c** in toluene solution at 23 °C. Source data are provided as Source Data File.

4.6 Molar absorption coefficient of diaryl-HI 3a

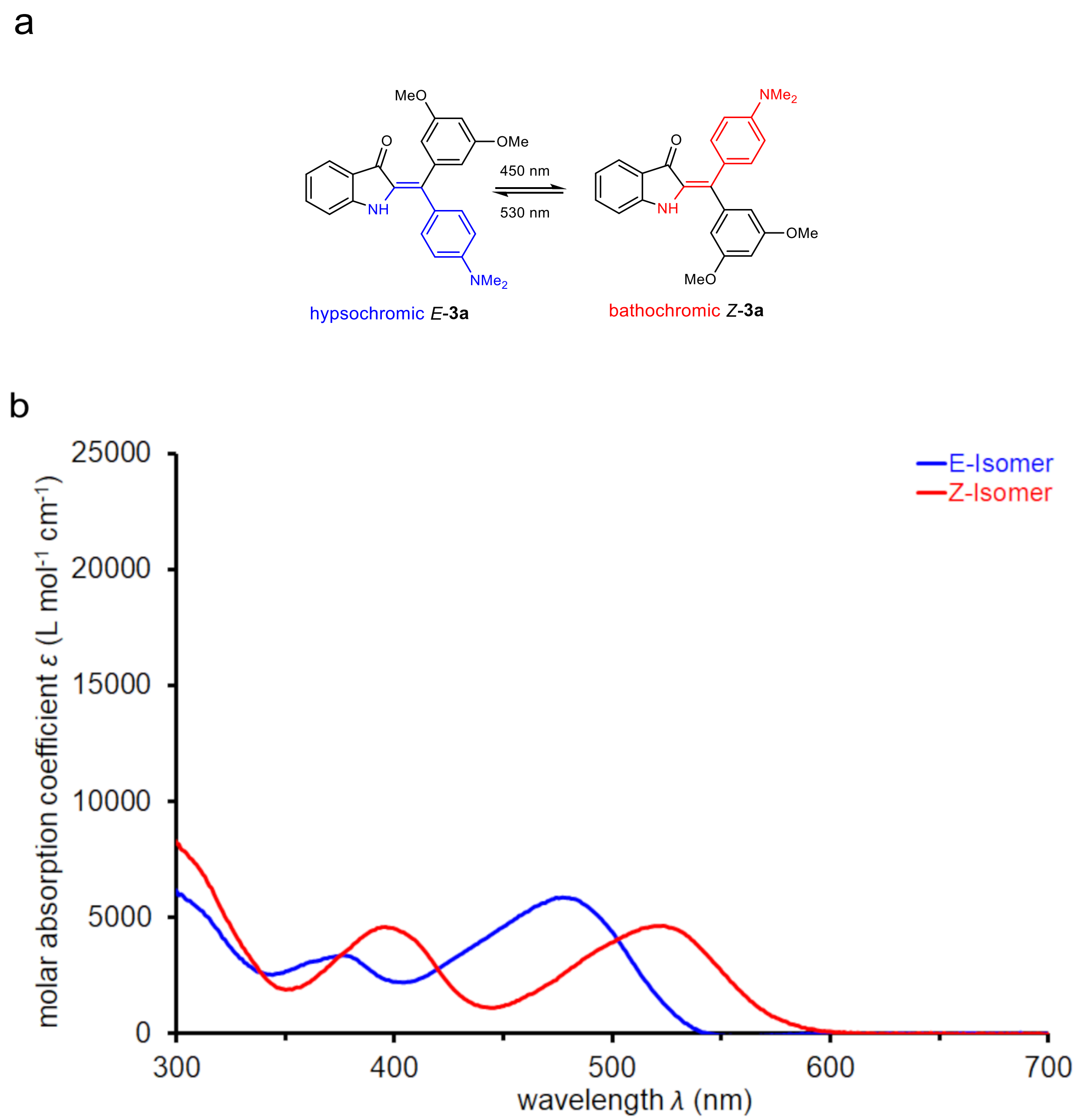

Supplementary Figure 40: a Schematic illustration of the photoisomerization from the hypsochromic (marked blue) to the bathochromic (marked red) diaryl-HI isomers of **3a** and vice versa. **b** Experimentally determined molar absorption coefficients of the pure *E* (marked blue) and *Z* (marked red) isomers of diaryl-HI **3a** in toluene solution at 23 °C. Source data are provided as Source Data File.

4.7 Molar absorption coefficient of diaryl-HI 3b

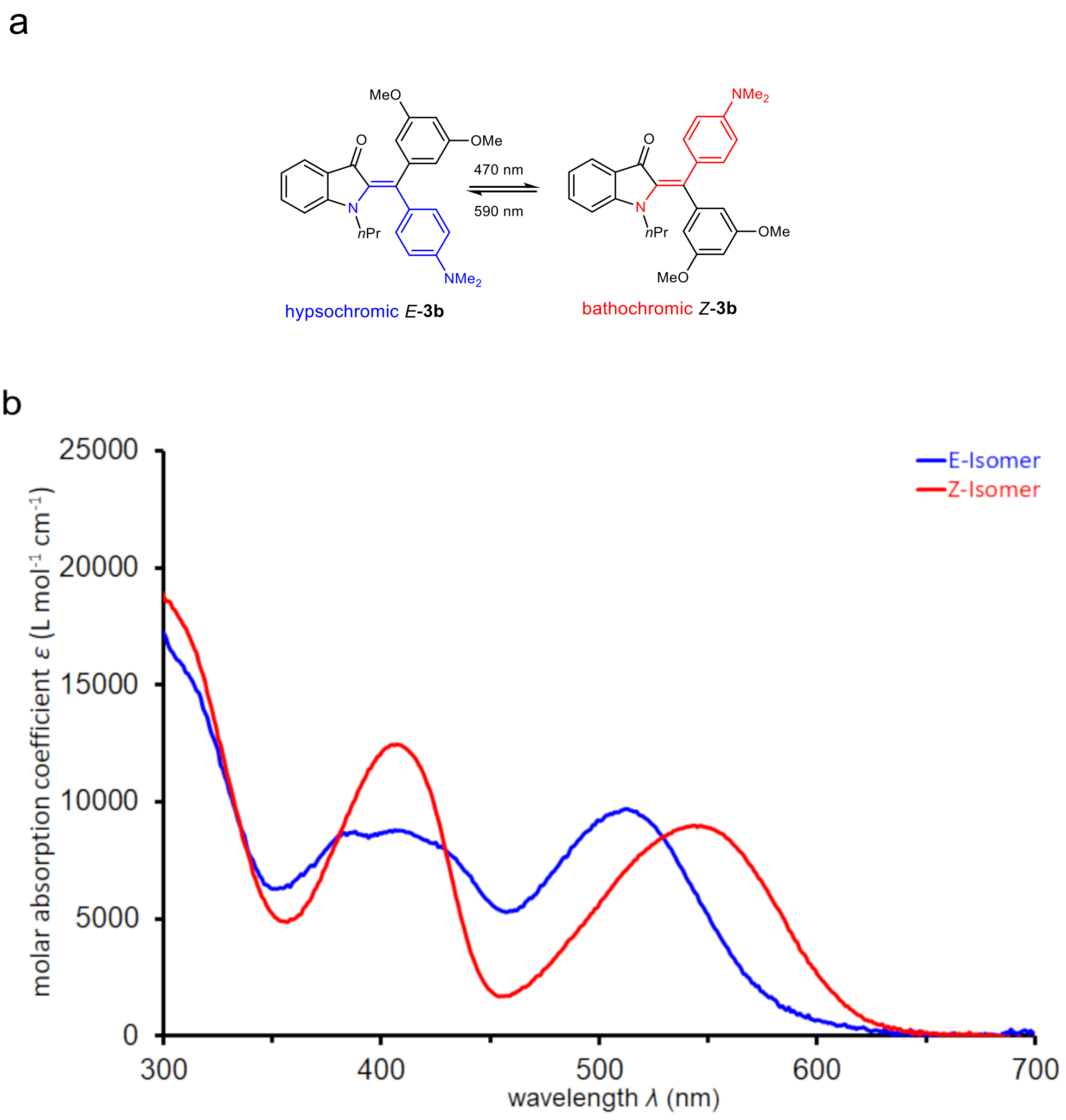

Supplementary Figure 41: a Schematic illustration of the photoisomerization from the hypsochromic (marked blue) to the bathochromic (marked red) diaryl-HI isomers of **3b** and vice versa. **b** Experimentally determined molar absorption coefficients of the pure *E* (marked blue) and *Z* (marked red) isomers of diaryl-HI **3b** in toluene solution at 23 °C. Source data are provided as Source Data File.

4.8 Molar absorption coefficient of diaryl-HI 3c

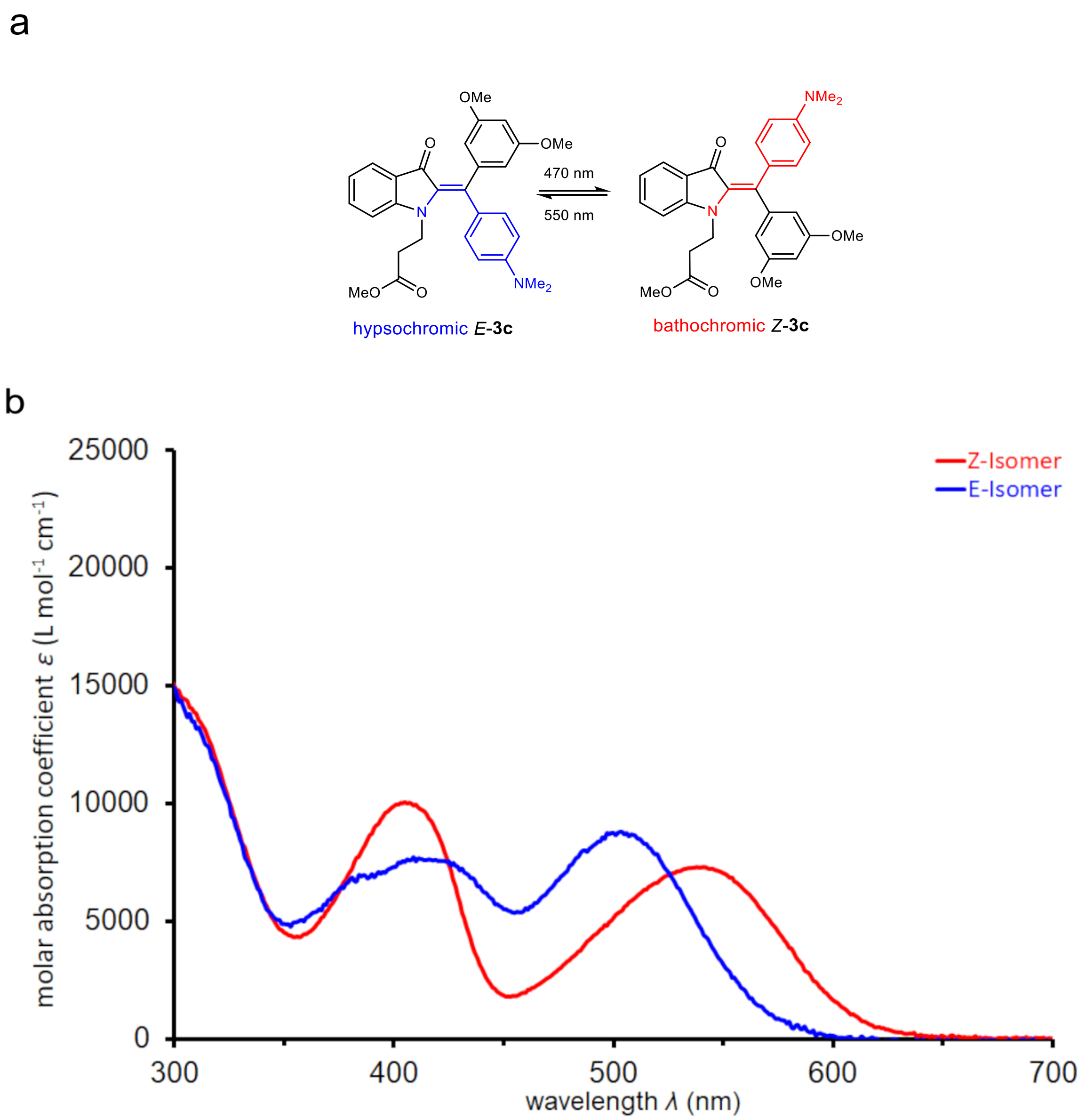

Supplementary Figure 42: a Schematic illustration of the photoisomerization from the hypsochromic (marked blue) to the bathochromic (marked red) diaryl-HI isomers of **3c** and vice versa. **b** Experimentally determined molar absorption coefficients of the pure *E* (marked blue) and *Z* (marked red) isomers of diaryl-HI **3c** in toluene solution at 23 °C. Source data are provided as Source Data File.

4.9 Molar absorption coefficient of diaryl-HI 4b

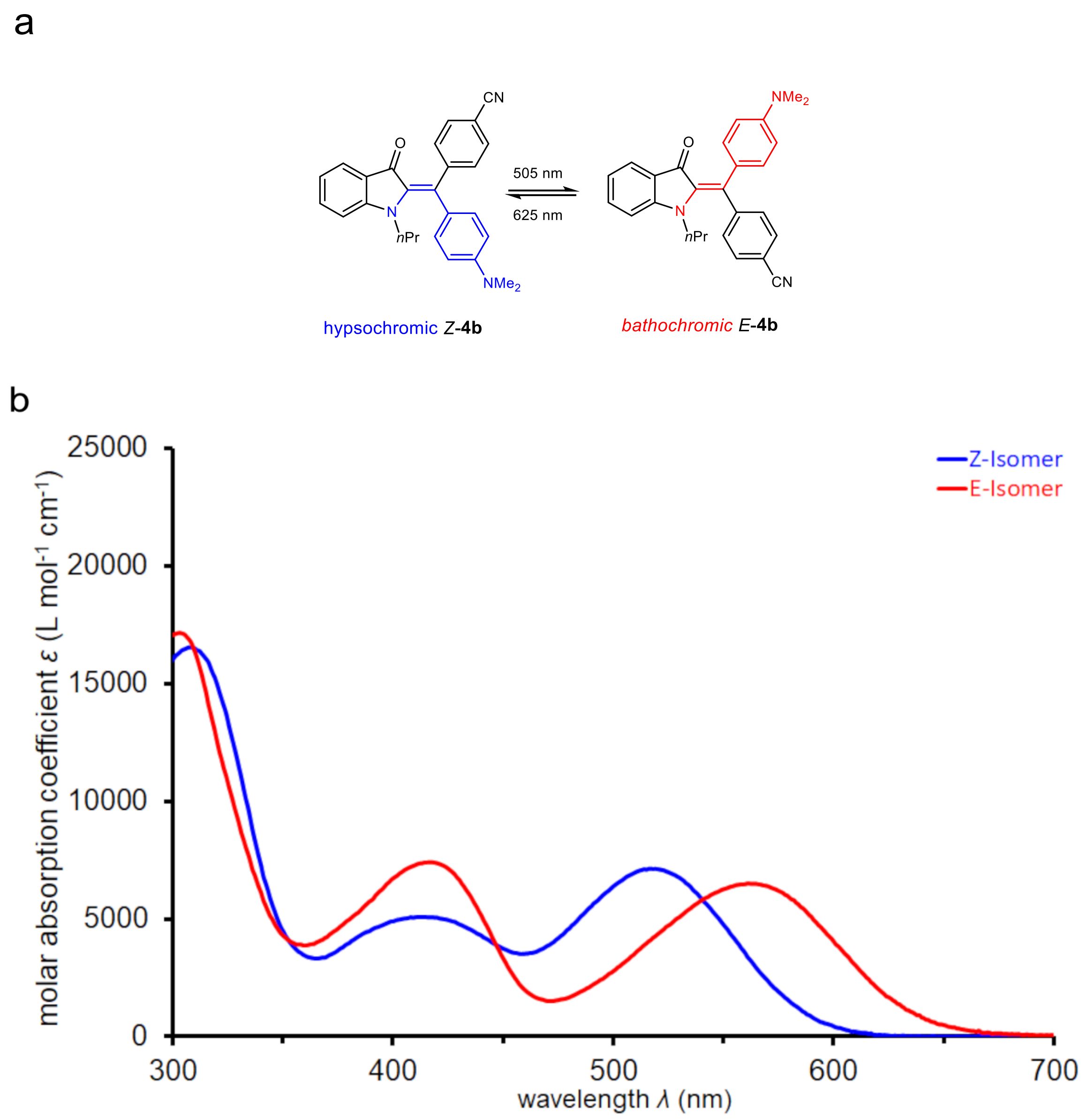

Supplementary Figure 43: a Schematic illustration of the photoisomerization from the hypsochromic (marked blue) to the bathochromic (marked red) diaryl-HI isomers of **4b** and vice versa. **b** Experimentally determined molar absorption coefficients of the pure *Z* (marked blue) and *E* (marked red) isomers of diaryl-HI **4b** in toluene solution at 23 °C. Source data are provided as Source Data File.

4.10 Molar absorption coefficient of diaryl-HI 4c

a

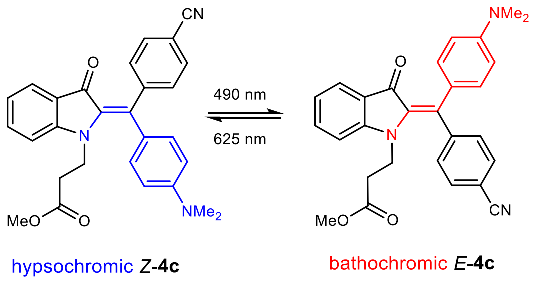

b

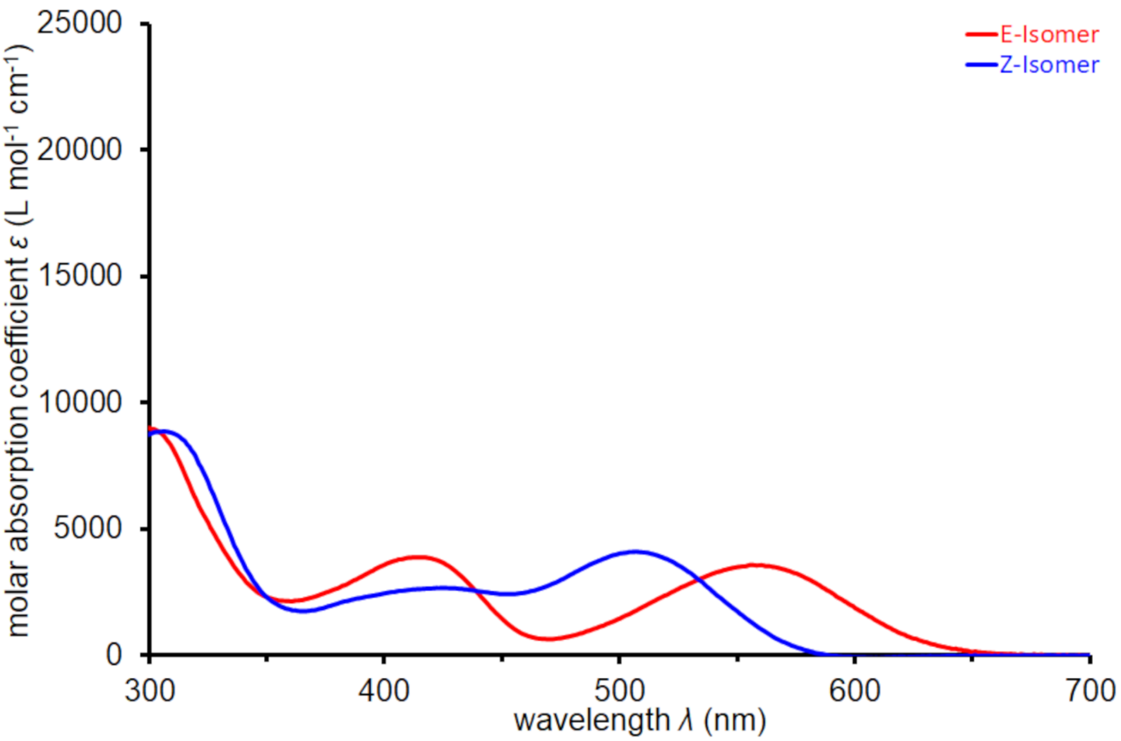

Supplementary Figure 44: a Schematic illustration of the photoisomerization from the hypsochromic (marked blue) to the bathochromic (marked red) diaryl-HI isomers of **4c** and vice versa. **b** Experimentally determined molar absorption coefficients of the pure *Z* (marked blue) and *E* (marked red) isomers of diaryl-HI **4c** in toluene solution at 23 °C. Source data are provided as Source Data File.

Supplementary Note 5: Photoisomerization experiments using UV/Vis and NMR spectroscopy

For preparation of stock solutions in deuterated solvents 1 mL volumetric flasks were charged with 1.25 to 2.88 mg diaryl-HI sample weighted on a Sartorius Cubis® MSE2.7S ultrafine balance. The samples of the respective diaryl-HIs in toluene-d_8 with known concentrations were irradiated with LEDs of 450 nm to 625 nm at 23 °C and the isomeric compositions at pss were afterwards determined by integration of suitable signals in the ^{1}H NMR spectra. For photoisomerization experiments in UV/Vis cuvettes, defined volumes of 10 to 180 µL were removed from the 1 mL stock solutions in deuterated solvents and dissolved in 2.50 mL toluene (spectroscopic grade). Afterwards, the quartz cuvettes were irradiated with LEDs of 450 nm to 625 nm at 23 °C and the isomer compositions were determined subsequently using the known molar absorption coefficients. The somewhat curious incomplete bathochromic to hypsochromic photoconversions are most likely explained by the tailing emissions of red-light LEDs, which leads to concurrent excitation of the hypsochromic photoproduct.

For the experiments, LEDs from Roithner Lasertechnik GmbH and Thorlabs GmbH (340 nm, 53 mW; 365 nm, 1150 mW; 385 nm, 1650 mW; 395 nm, 400 mW, 405 nm, 1500 mW; 420 nm, 174 mW; 430 nm, 490 mW; 450 nm, 1850 mW, 470 nm, 760 mW; 490 nm, 205 mW; 505 nm, 400 mW; 515 nm, 150 mW; 530 nm, 370 mW; 565 nm, 880 mW; 590 nm 230 mW, 595 nm, 820 mW; 625 nm, 700 mW; 660 nm, 1050 mW; 680 nm, 210 mW, 730 nm, 680 mW) were used for irradiation of UV/Vis and NMR samples. Emission profiles for the most relevant LEDs used in this study are found in the Supporting Information of ref. [3]. For irradiation of the NMR samples a LED from Mountain Photonics GmbH (625 nm, UHP-T-625-DI) was used additionally.

Supplementary Table 3: *E*/*Z* isomeric ratios of hypsochromic and bathochromic isomers of diaryl-HIs **1-4** obtained in the photostationary state (pss) after irradiation with light of different wavelengths in toluene-d_8 or tetrahydrofuran-d_8 solutions at 23 °C. All values given were determined using ^{1}H NMR spectroscopy.

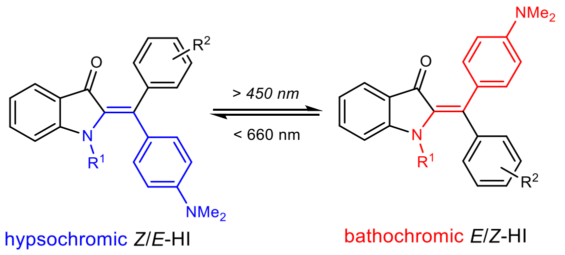

HI	pss hypsochromic enriched species (% *E*/*Z*) (LED nm)	pss bathochromic enriched species (% *E*/*Z*) (LED nm)
**1a**	47% *E*[C] (590 nm)	94% *Z*[C] (450 nm)
**1b**	62% *E*[C] (625 nm)	100% *Z*[C] (470 nm)
**1c**	63% *E*[C] (625 nm)	100% *Z*[C] (470 nm)
**2a**	-[A,C]	-[A,C]
**2b**	79% *E*[C] (625 nm)	92% *Z*[C] (490 nm)
**2c**	92% *E*[C] / 91% *E*[B] (660 nm[C] / 625 nm[B])	92% *Z*[C] / 81% *Z*[B] (470 nm)
**3a**	50% *E*[A,C] (530 nm)	53% *Z*[A,C] (450 nm)
**3b**	62% *E*[C] (590 nm)	92% *Z*[C] (470 nm)
**3c**	46% *E*[C] (550 nm)	72% *Z*[C] (470 nm)
**4a**	-[A,C]	-[A,C]
**4b**	87% *Z*[C] / 88% *Z*[B] (660 nm[C] / 625 nm[B])	89% *E*[C] / 81% *E*[B] (505 nm)
**4c**	93% *Z*[C] / 88% *Z*[B] (660 nm[C] / 625 nm[B])	90% *E*[C] / 90% *E*[B] (490 nm)

A: photodegradation occurred due to irradiation with light. **B:** in tetrahydrofuran-d_8 solution at 23 °C. **C:** in toluene-d_8 solution at 23 °C.

Supplementary Table 4: Corresponding wavelengths λ_{max} and wavelength differences $\Delta\lambda_{max}$ at lowest energy absorption maximum A_{max} recorded for diaryl-HIs **1-4** in solvents of different polarity at 23 °C after reaching the pss using different irradiation wavelengths. All values given were determined using UV/Vis spectroscopy.

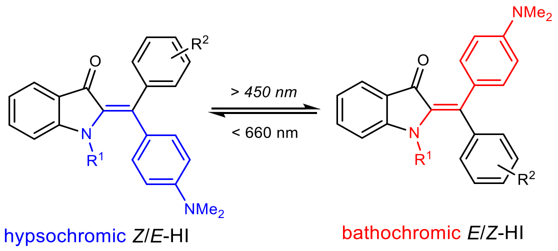

HI	λ_{max} at lowest energy A_{max} at pss of hypsochromic enriched species (nm) (LED nm)	λ_{max} at lowest energy A_{max} at pss of bathochromic enriched species (nm) (LED nm)	$\Delta\lambda_{max}$ between lowest energy ΔA_{max} of hypsochromic and bathochromic enriched species at pss (nm)
**1a**	$478^{C, G}$ (590 nm)	$518^{B, G}$ (450 nm)	40^{G}
**1b**	$504^{C, G}$ / 529^{E} / 525^{J} (617 nm^{G} / 660 nm^{E} / 625 nm^{J})	$537^{B, G}$ / 559^{E} /560^{J} (470 nm)	33^{G} / 30^{E} / 35^{J}
**1c**	$497^{C, G}$ (617 nm)	$532^{B, G}$ (470 nm)	35^{G}
**2a**^{A}	$488^{C, G}$ (23 °C)	$488^{B, G}$ (470)	0^{G}
**2b**	$521^{C, G}$ (625 nm)	$556^{B, G}$ (490 nm)	35^{G}
**2c**	$511^{C, G}$ / $512^{C, D}$ / $530^{E, F}$ / 527^{I} (625 nm)	$550^{B, G}$ / $542^{B, D}$ / $549^{B, E}$ / 540^{I} (470 nm)	39^{G} / 30^{D} / 19^{E} / 13^{I}
**3a**^{A}	$488^{C, G}$ (530 nm)	$506^{B, G}$ (450 nm)	18^{G}
**3b**	$517^{C, G}$ (590 nm)	$543^{B, G}$ (470 nm)	26^{G}
**3c**	$522^{C, G}$ (550 nm)	$535^{B, G}$ (470 nm)	13^{G}
**4a**^{A}	$488^{B, G}$ (23 °C)	$495^{C, G}$ (450 nm)	7^{G}
**4b**	$522^{B, G}$ / $523^{B, D}$ / $544^{H, E}$ (625 nm)	$558^{C, G}$ / $549^{C, D}$ / $552^{C, E}$ (505 nm)	36^{G} / 26^{D} / 8^{E}
**4c**	$510^{B, G}$ / $512^{B, D}$ / $531^{H, E}$ (625 nm)	$548^{C, G}$ / $543^{C, D}$ / $552^{C, E}$ (490 nm)	38^{G} / 31^{D} / 21^{E}

A: Photoisomerization without photodegradation is possible at UV/Vis concentrations. **B:** *Z* isomer enriched. **C:** *E* isomer enriched. **D:** in tetrahydrofuran. **E:** in methanol. **F:** thermal enrichment of *E* isomer at 23 °C. **G:** in toluene. **H:** thermal enrichment of *Z* isomer at 23 °C. **I:** in dimethylsulfoxide (DMSO). **J:** DMSO:H_2O 2:1.

5.1 Photoisomerization of diaryl-HI 1a

a

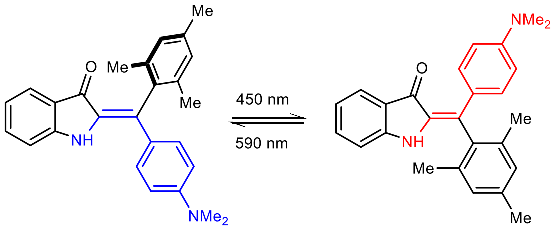

hypsochromic *E*-**1a** bathochromic *Z*-**1a**

b

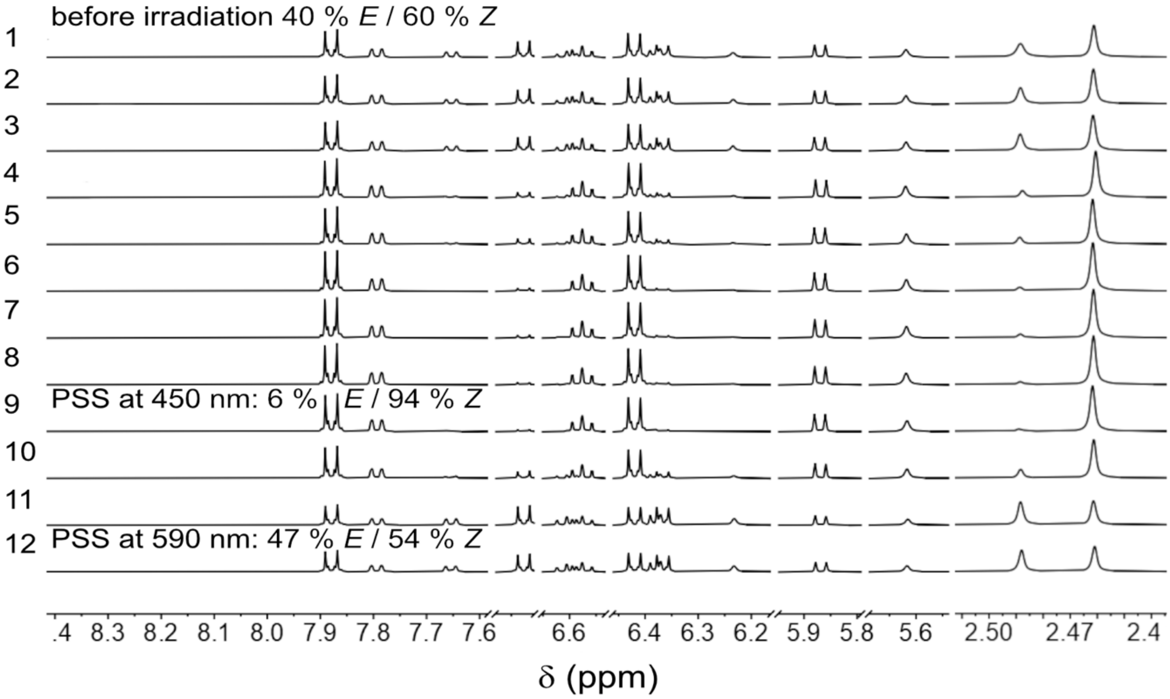

Supplementary Figure 45: a Schematic illustration of the photoisomerization from the hypsochromic to the bathochromic isomer of diaryl-HI **1a** and vice versa. **b** ^{1}H NMR spectra 1 – 12 (400 MHz, toluene-d_8, 23 °C) of diaryl-HI **1a** recorded after different irradiation durations using light of different wavelengths at 23 °C. Magnification varies in the 2.5 to 2.4 ppm section of the partial ^{1}H NMR spectra. From 1 to 12: partial ^{1}H NMR spectrum of diaryl-HI **1a** with isomeric composition of 40% *E* isomer and 60% *Z* isomer before irradiation in spectrum 1, progress of *Z* isomer enrichment by irradiating with a 450 nm LED to the pss resulting in an isomer composition of 6% *E* isomer and 94% *Z* isomer in spectrum 9, progress of *E* isomer enrichment by irradiating with a 590 nm LED to the pss with an isomeric composition of 47% *E* isomer and 54% *Z* isomer in spectrum 12. Source data are provided as Source Data File.

a

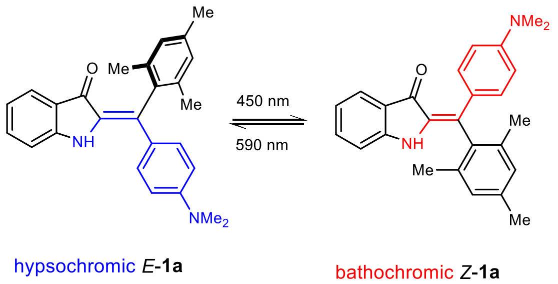

b

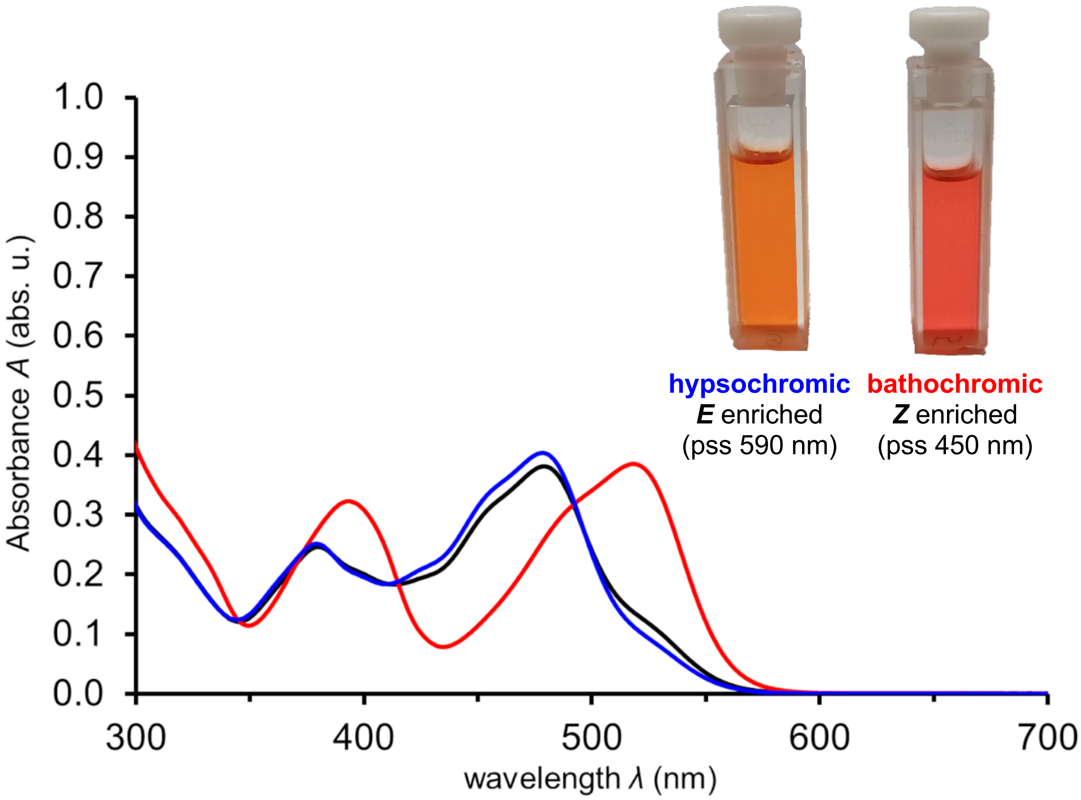

Supplementary Figure 46: a Schematic illustration of the photoisomerization from the hypsochromic to the bathochromic isomer of diaryl-HI **1a** and vice versa. **b** Absorption spectra of diaryl-HI **1a** in toluene solution at 23 °C recorded before and after different irradiation durations using light of different wavelengths. Absorbance of a diaryl-HI **1a** solution containing a mixture of *E* and *Z* isomer before irradiation (black line, A_{max} at 479 nm), absorbance of the bathochromic *Z* isomer enriched solution (red line, A_{max} at 518 nm) obtained after irradiation to the pss with 450 nm light, absorbance of the hypsochromic *E* isomer enriched solution (blue line, A_{max} at 478 nm) obtained after irradiation to the pss with 590 nm light. Source data are provided as Source Data File.

5.2 Photoisomerization of diaryl-HI 1b

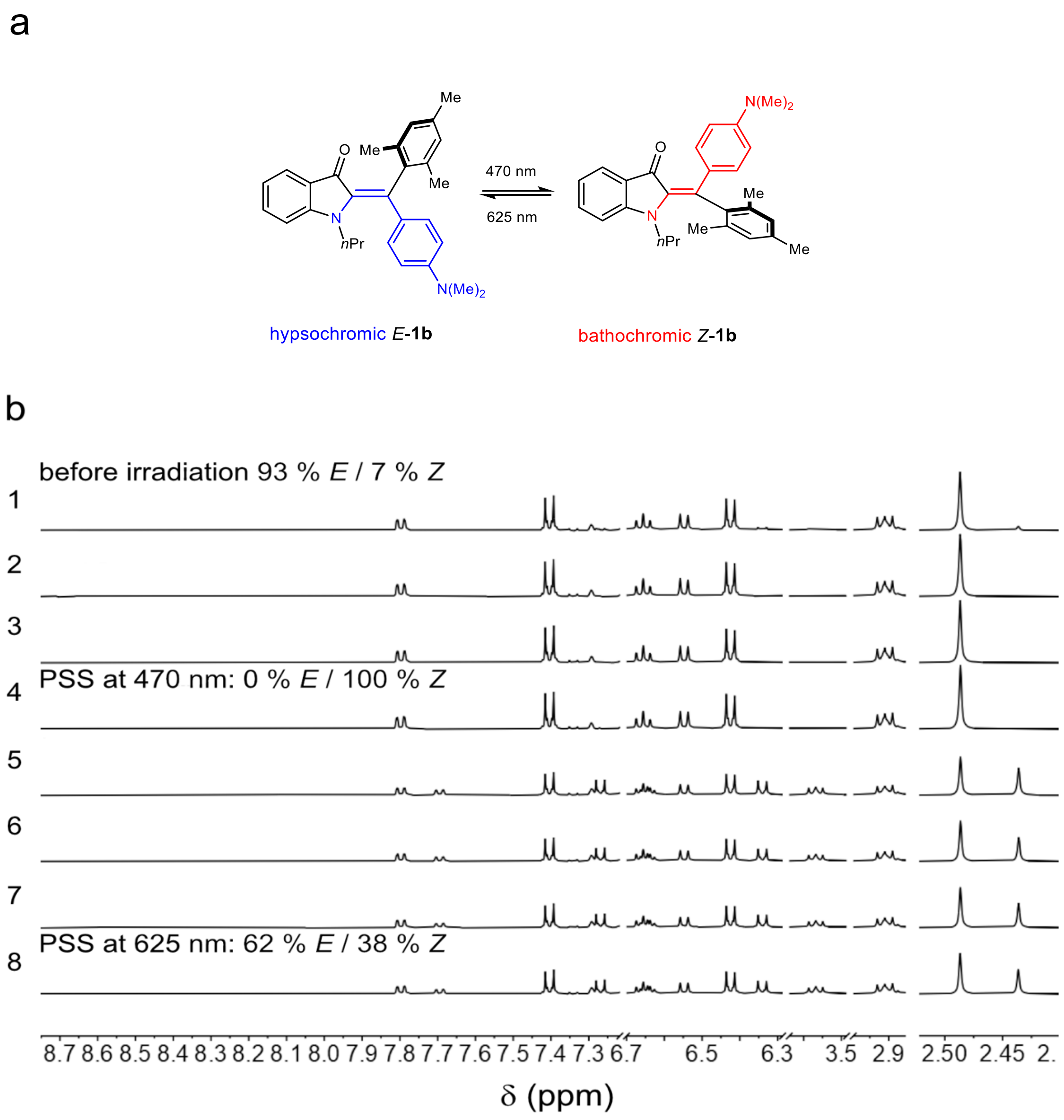

Supplementary Figure 47: a Schematic illustration of the photoisomerization from the hypsochromic to the bathochromic isomer of diaryl-HI **1b** and vice versa. **b** ^{1}H NMR spectra 1 – 8 (400 MHz, toluene-d_8, 23 °C) of diaryl-HI **1b** recorded after different irradiation durations using light of different wavelengths at 23 °C. Magnification varies in the 2.5 to 2.4 ppm section of the partial ^{1}H NMR spectra. From 1 - 8: partial ^{1}H NMR spectrum of diaryl-HI **1b** with isomeric composition of 93% *E* isomer and 7% *Z* isomer before irradiation in spectrum 1, progress of *Z* isomer enrichment by irradiating with a 470 nm LED to the pss resulting in an isomer composition of 0% *E* isomer and 100% *Z* isomer in spectrum 4, progress of *E* isomer enrichment by irradiating with a 625 nm LED to the pss with an isomeric composition of 62% *E* isomer and 38% *Z* isomer in spectrum 8. Source data are provided as Source Data File.

a

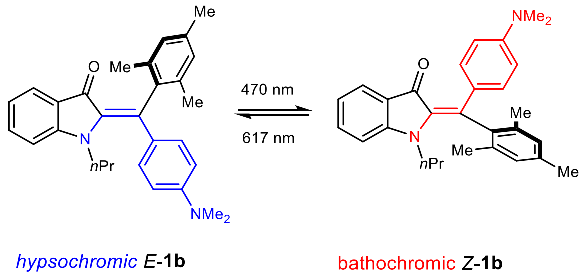

b

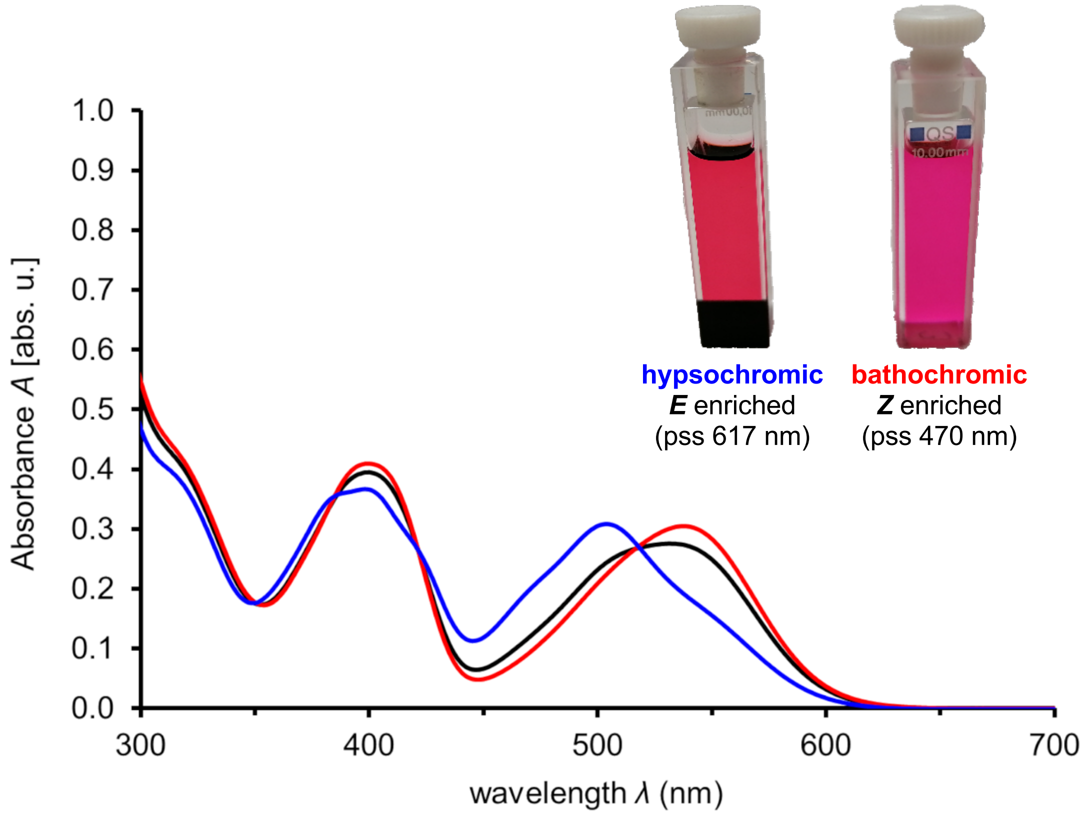

Supplementary Figure 48: a Schematic illustration of the photoisomerization from the hypsochromic to the bathochromic isomer of diaryl-HI **1b** and vice versa. **b** Absorption spectra of diaryl-HI **1b** in toluene solution at 23 °C recorded before and after different irradiation durations using light of different wavelengths. Absorbance of a diaryl-HI **1b** solution containing a mixture of *E* and *Z* isomer before irradiation (black line, A_{max} at 531 nm), absorbance of the bathochromic *Z* isomer enriched solution (red line, A_{max} at 537 nm) obtained after irradiation to the pss with 470 nm light, absorbance of the hypsochromic *E* isomer enriched solution (blue line, A_{max} at 504 nm) obtained after irradiation to the pss with 617 nm light. Source data are provided as Source Data File.

a

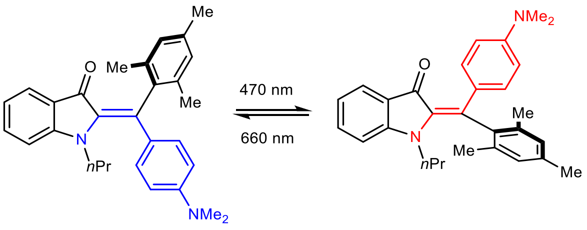

b

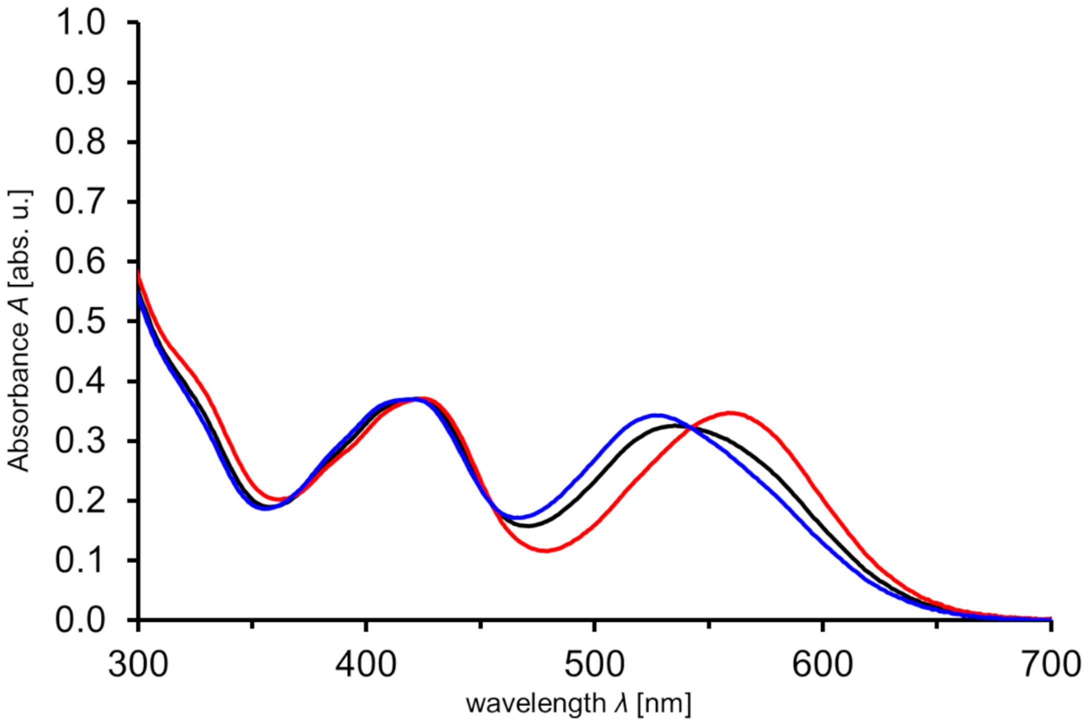

Supplementary Figure 49: a Schematic illustration of the photoisomerization from the hypsochromic to the bathochromic isomer of diaryl-HI **1b** and vice versa. **b** Absorption spectra of diaryl-HI **1b** in methanol solution at 23 °C recorded before and after different irradiation durations using light of different wavelengths. Absorbance of a diaryl-HI **1b** solution containing a mixture of *E* and *Z* isomer before irradiation (black line, A_{max} at 535 nm), absorbance of the bathochromic *Z* isomer enriched solution (red line, A_{max} at 559 nm) obtained after irradiation to the pss with 470 nm light, absorbance of the hypsochromic *E* isomer enriched solution (blue line, A_{max} at 529 nm) obtained after irradiation to the pss with 660 nm light. No effect of reduced thermal stability was noticed. The bathochromic *Z*-**1b** isomer enriched solution at pss was stable for at least 45 min at 23 °C in the dark. Source data are provided as Source Data File.

a

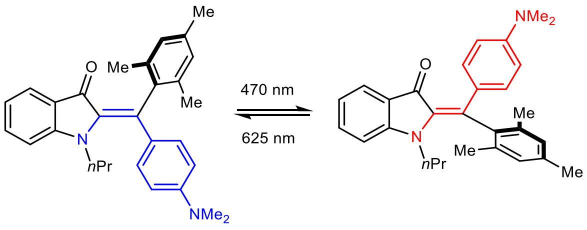

b

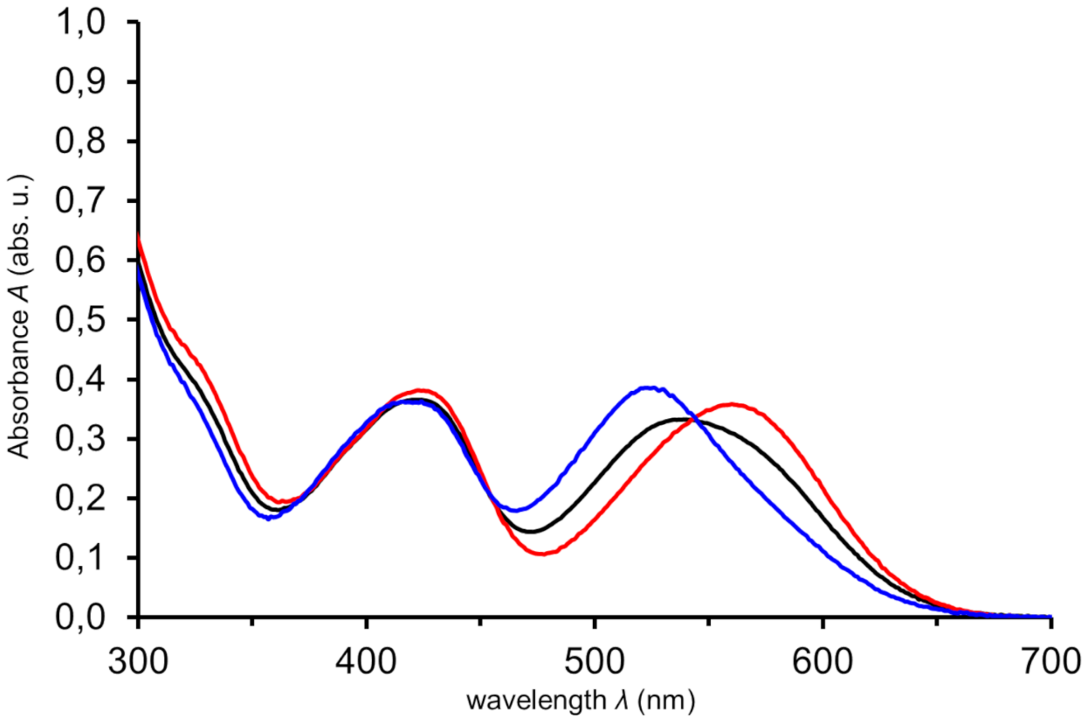

Supplementary Figure 50: a Schematic illustration of the photoisomerization from the hypsochromic to the bathochromic isomer of diaryl-HI **1b** and vice versa. **b** Absorption spectra of diaryl-HI **1b** in DMSO:H_2O 2:1 solution at 23 °C recorded before and after different irradiation durations using light of different wavelengths. Absorbance of a diaryl-HI **1b** solution containing a mixture of E and Z isomer before irradiation (black line, A_{max} at 539 nm), absorbance of the bathochromic Z isomer enriched solution (red line, A_{max} at 560 nm) obtained after irradiation to the pss with 470 nm light, absorbance of the hypsochromic E isomer enriched solution (blue line, A_{max} at 525 nm) obtained after irradiation to the pss with 625 nm light. No reduced thermal stability was noticed. The bathochromic Z-**1b** isomer enriched solution at pss was stable for at least 33 min at 23 °C in the dark. Source data are provided as Source Data File.

5.3 Photoisomerization of diaryl-HI 1c

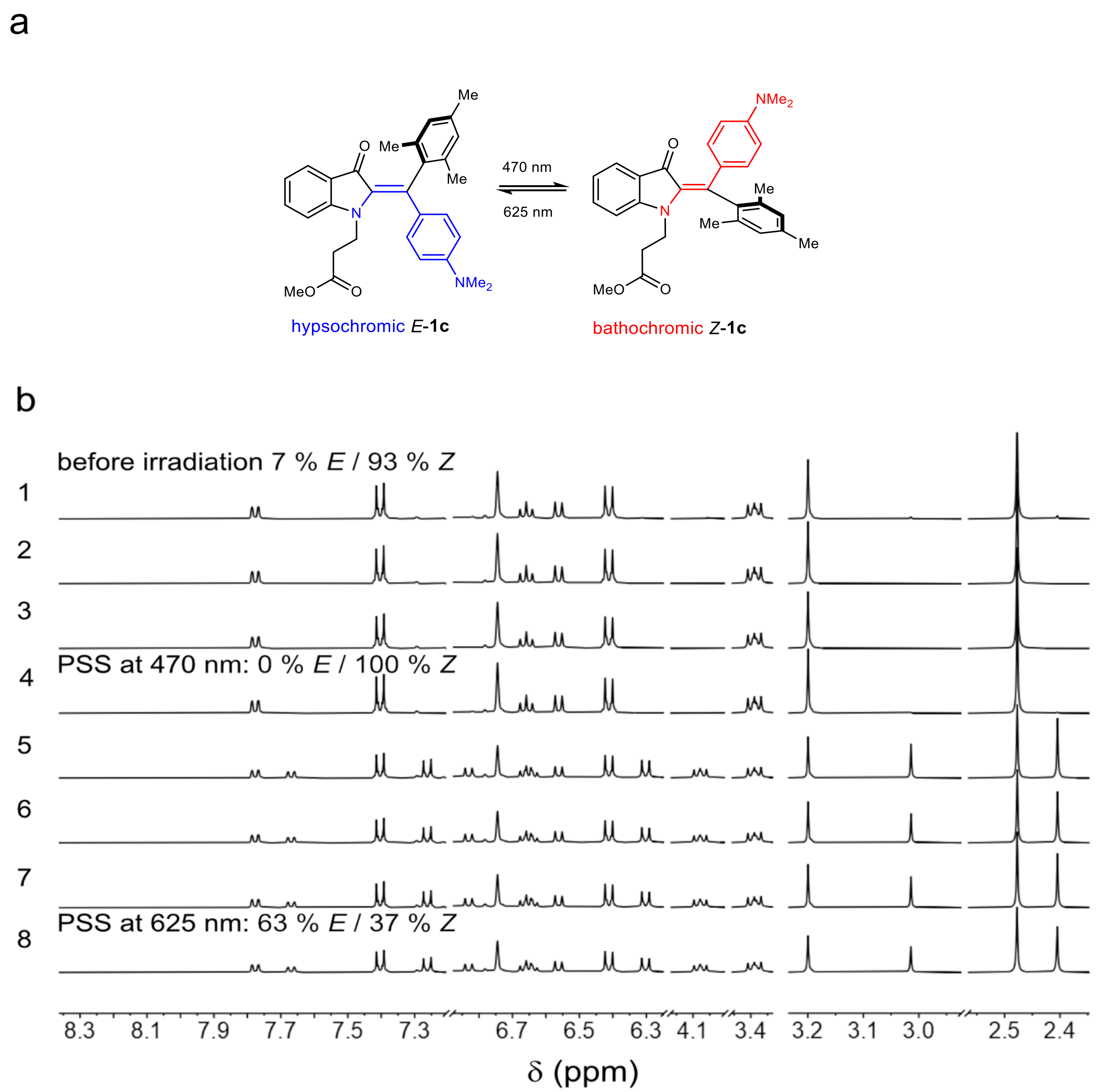

Supplementary Figure 51: **a** Schematic illustration of the photoisomerization from the hypsochromic to the bathochromic isomer of diaryl-HI **1c** and vice versa. **b** ^{1}H NMR spectra (400 MHz, toluene-d_8, 23 °C) 1 – 8 of diaryl-HI **1c** recorded after different irradiation durations using light of different wavelengths at 23 °C. Magnification varies in the 3.2 to 2.4 ppm section of the partial ^{1}H NMR spectra. From 1 - 8: partial ^{1}H NMR spectrum of diaryl-HI **1c** with isomeric composition of 93% *E* isomer and 7% *Z* isomer before irradiation in spectrum 1, progress of *Z* isomer enrichment by irradiating with a 470 nm LED to the pss resulting in an isomer composition of 0% *E* isomer and 100% *Z* isomer in spectrum 4, progress of *E* isomer enrichment by irradiating with a 625 nm LED to the pss with an isomeric composition of 63% *E* isomer and 37% *Z* isomer in spectrum 8. Source data are provided as Source Data File.

a

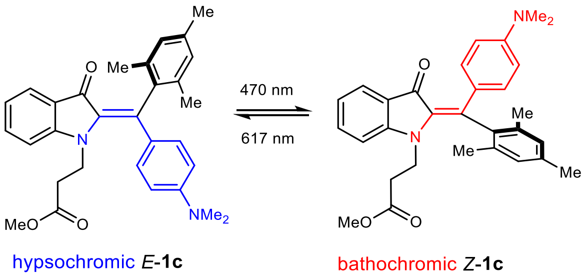

b

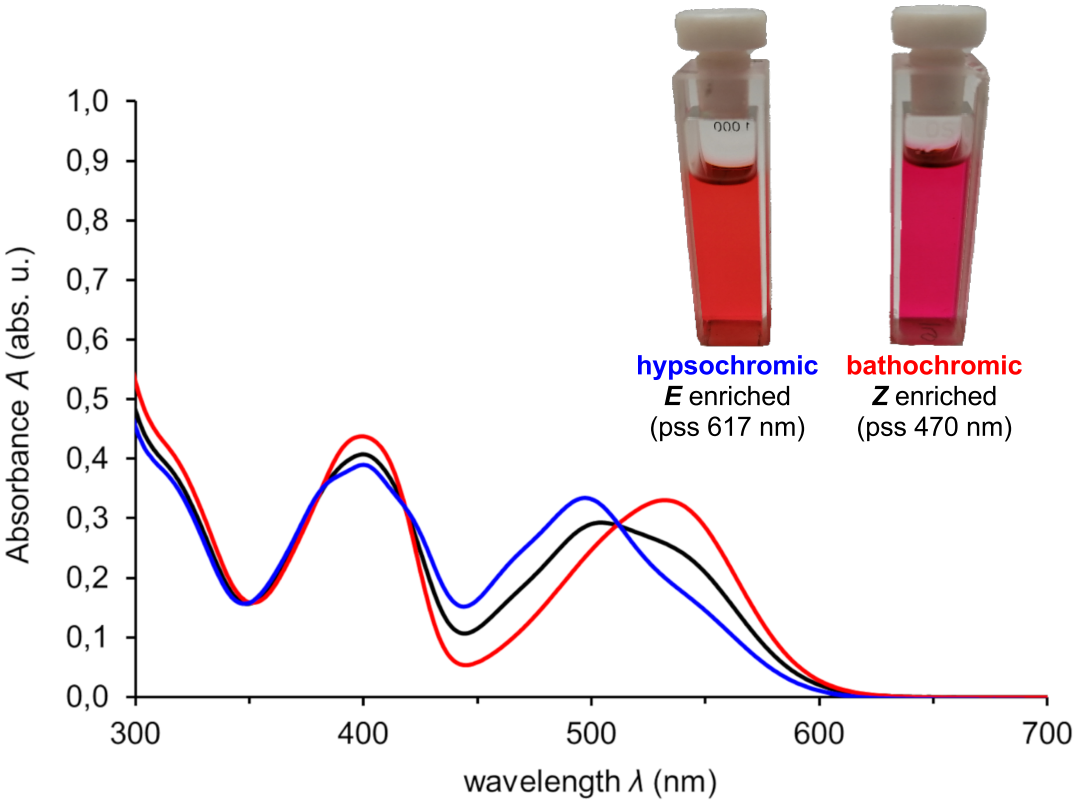

Supplementary Figure 52: a Schematic illustration of the photoisomerization from the hypsochromic to the bathochromic isomer of diaryl-HI **1c** and vice versa. **b** Absorption spectra of diaryl-HI **1c** in toluene solution at 23 °C recorded before and after different irradiation durations using light of different wavelengths. Absorbance of a diaryl-HI **1c** solution containing a mixture of *E* and *Z* isomer before irradiation (black line, A_{max} at 504 nm), absorbance of the bathochromic *Z* isomer enriched solution (red line, A_{max} at 532 nm) obtained after irradiation to the pss with 470 nm light, absorbance of the hypsochromic *E* isomer enriched solution (blue line, A_{max} at 497 nm) obtained after irradiation to the pss with 617 nm light. Source data are provided as Source Data File.

5.4 Photoisomerization of diaryl-HI 2a

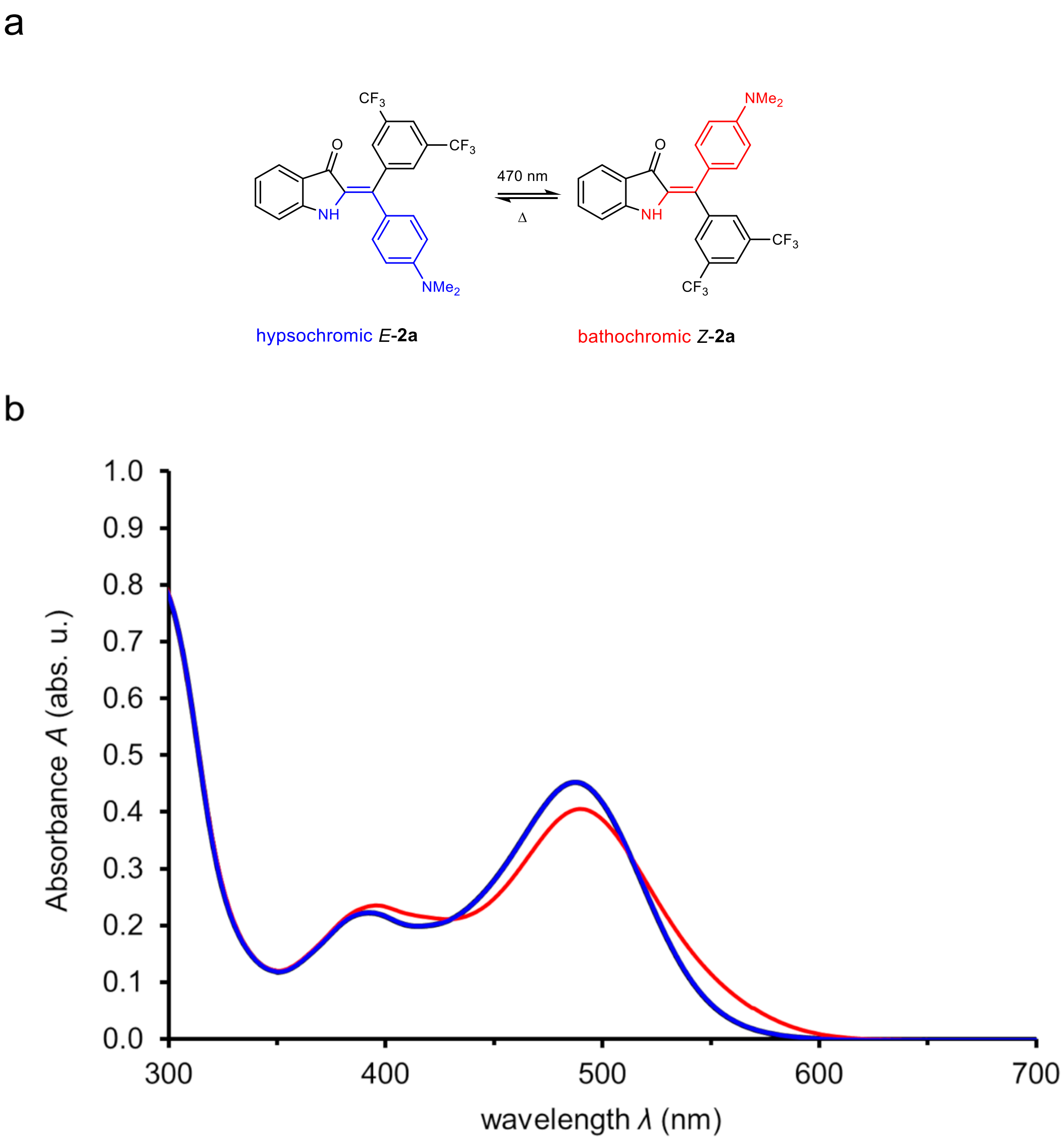

Supplementary Figure 53: a Schematic illustration of the photoisomerization from the hypsochromic to the bathochromic isomer of diaryl-HI **2a** and vice versa. **b** Absorption spectra of diaryl-HI **2a** in toluene solution at 23 °C recorded before and after different irradiation durations using light of different wavelengths. Absorbance of a diaryl-HI **2a** solution containing a mixture of *E* and *Z* isomer before irradiation (black line, A_{max} at 488 nm), absorbance of the bathochromic *Z* isomer enriched solution (red line, A_{max} at 488 nm) obtained after irradiation with 470 nm light, absorbance of the hypsochromic *E* isomer enriched solution (blue line, A_{max} at 488 nm) obtained after thermal *Z* to *E* isomerization at 23 °C of bathochromic *Z* isomer enriched solution. Source data are provided as Source Data File.

5.5 Photoisomerization of diaryl-HI 2b

a

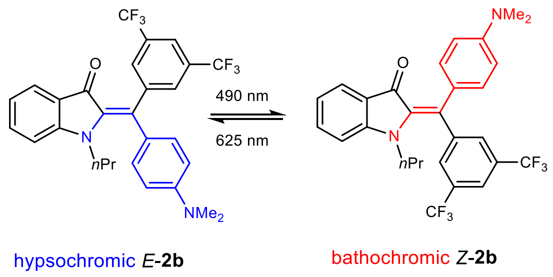

b

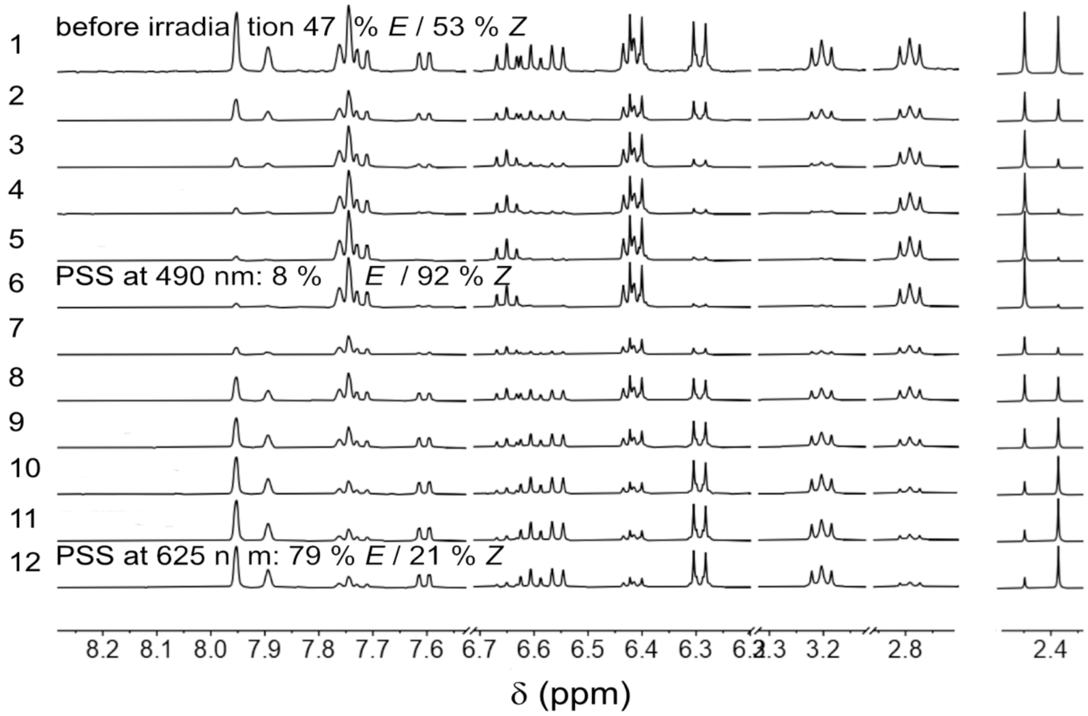

Supplementary Figure 54: a Schematic illustration of the photoisomerization from the hypsochromic to the bathochromic isomer of diaryl-HI **2b** and vice versa. **b** ^{1}H NMR spectra (400 MHz, toluene-*d*8, 23 °C) 1 – 12 of diaryl-HI **2b** recorded after different irradiation durations using light of different wavelengths at 23 °C. Magnification varies in the 2.4 ppm section of the partial ^{1}H NMR spectra. From 1 - 12: partial ^{1}H NMR spectrum of diaryl-HI **2b** with isomeric composition of 47% *E* isomer and 53% *Z* isomer before irradiation in spectrum 1, progress of *Z* isomer enrichment by irradiating with a 490 nm LED to the pss resulting in an isomer composition of 8% *E* isomer and 92% *Z* isomer in spectrum 6, progress of *E* isomer enrichment by irradiating with a 625 nm LED to the pss with an isomeric composition of 79% *E* isomer and 21% *Z* isomer in spectrum 12. Source data are provided as Source Data File.

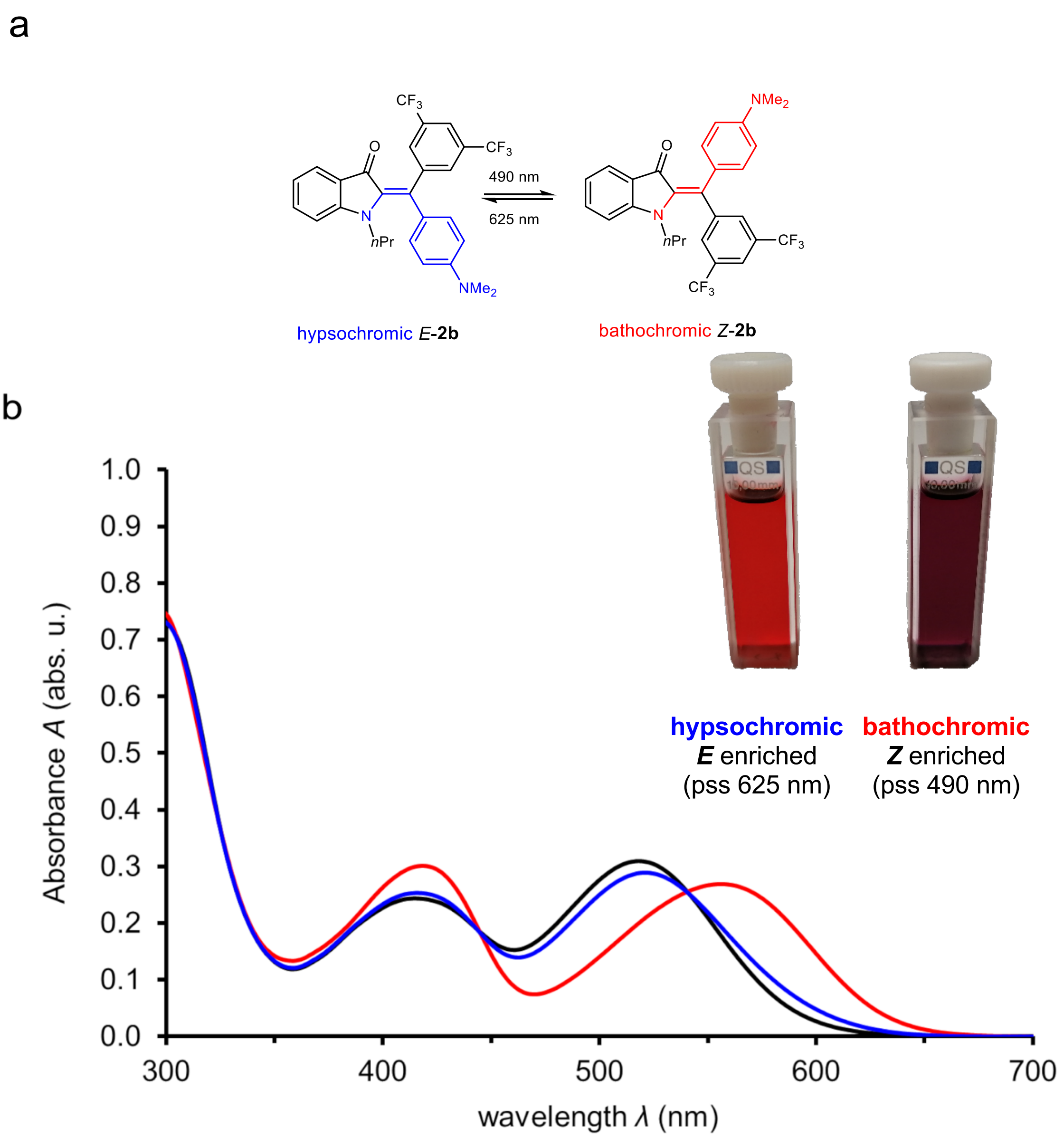

Supplementary Figure 55: a Schematic illustration of the photoisomerization from the hypsochromic to the bathochromic isomer of diaryl-HI **2b** and vice versa. **b** Absorption spectra of diaryl-HI **2b** in toluene solution at 23 °C recorded before and after different irradiation durations using light of different wavelengths. Absorbance of a diaryl-HI **2b** solution containing a mixture of *E* and *Z* isomer before irradiation (black line, A_{max} at 518 nm), absorbance of the bathochromic *Z* isomer enriched solution (red line, A_{max} at 556 nm) obtained after irradiation to the pss with 490 nm light, absorbance of the hypsochromic *E* isomer enriched solution (blue line, A_{max} at 521 nm) obtained after irradiation to the pss with 625 nm light. Source data are provided as Source Data File.

5.6 Photoisomerization of diaryl-HI 2c

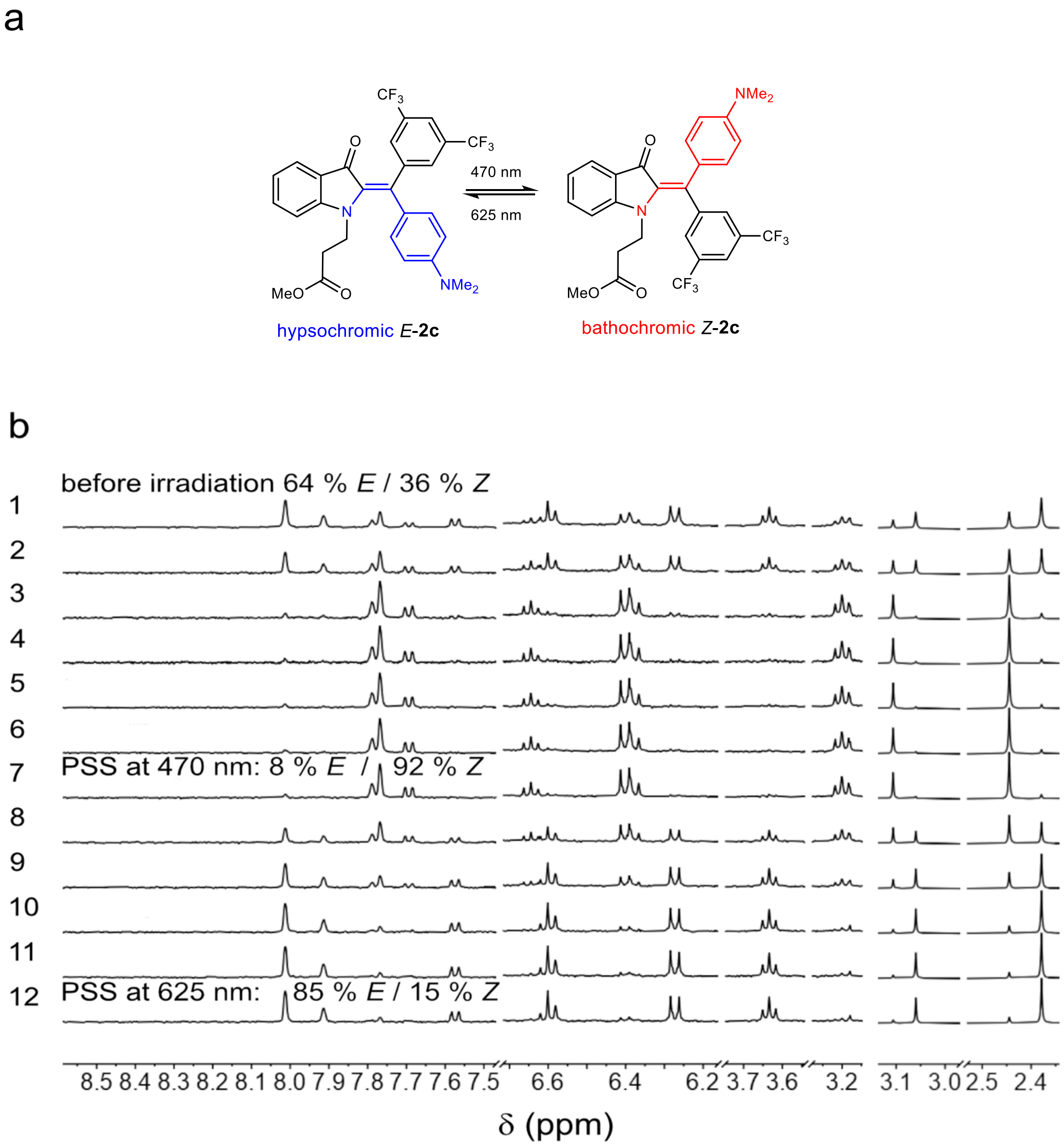

Supplementary Figure 56: a Schematic illustration of the photoisomerization from the hypsochromic to the bathochromic isomer of diaryl-HI **2c** and vice versa. **b** ^{1}H NMR spectra (400 MHz, toluene-*d*$_8$, 23 °C) 1 – 12 of diaryl-HI **2c** recorded after different irradiation durations using light of different wavelengths at 23 °C. Magnification varies in the 3.1 to 2.4 ppm section of the partial ^{1}H NMR spectra. From 1 - 12: partial ^{1}H NMR spectrum of diaryl-HI **2c** with isomeric composition of 64% *E* isomer and 36% *Z* isomer before irradiation in spectrum 1, progress of *Z* isomer enrichment by irradiating with a 470 nm LED to the pss resulting in an isomer composition of 8% *E* isomer and 92% *Z* isomer in spectrum 7, progress of *E* isomer enrichment by irradiating with a 625 nm LED to the pss with an isomeric composition of 85% *E* isomer and 15% *Z* isomer in spectrum 12. Source data are provided as Source Data File.

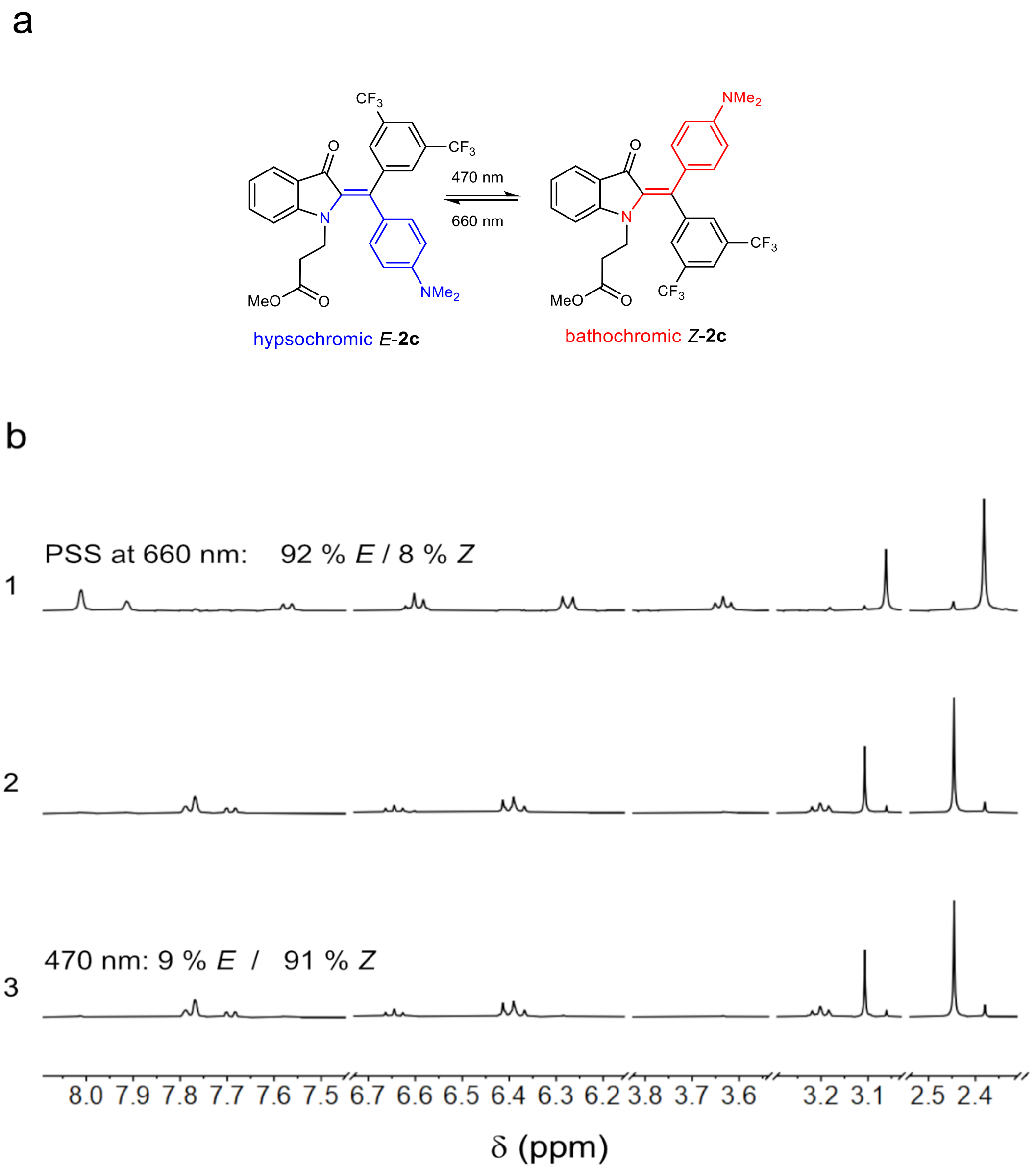

Supplementary Figure 57: a Schematic illustration of the photoisomerization from the hypsochromic to the bathochromic isomer of diaryl-HI **2c**. **b** ^{1}H NMR spectra (400 MHz, toluene-*d*$_8$, 23 °C) 1 – 3 of diaryl-HI **2c** recorded after different irradiation durations using light of different wavelengths at 23 °C. From 1 – 3: partial ^{1}H NMR spectrum of diaryl-HI **2c** with isomeric composition of 92% *E* isomer and 8% Z isomer after irradiation with light of 660 nm (pss) in spectrum 1, progress of *Z* isomer enrichment by irradiating with a 470 nm LED to an isomer composition of 9% *E* isomer and 91% *Z* isomer in spectrum 3. Source data are provided as Source Data File.

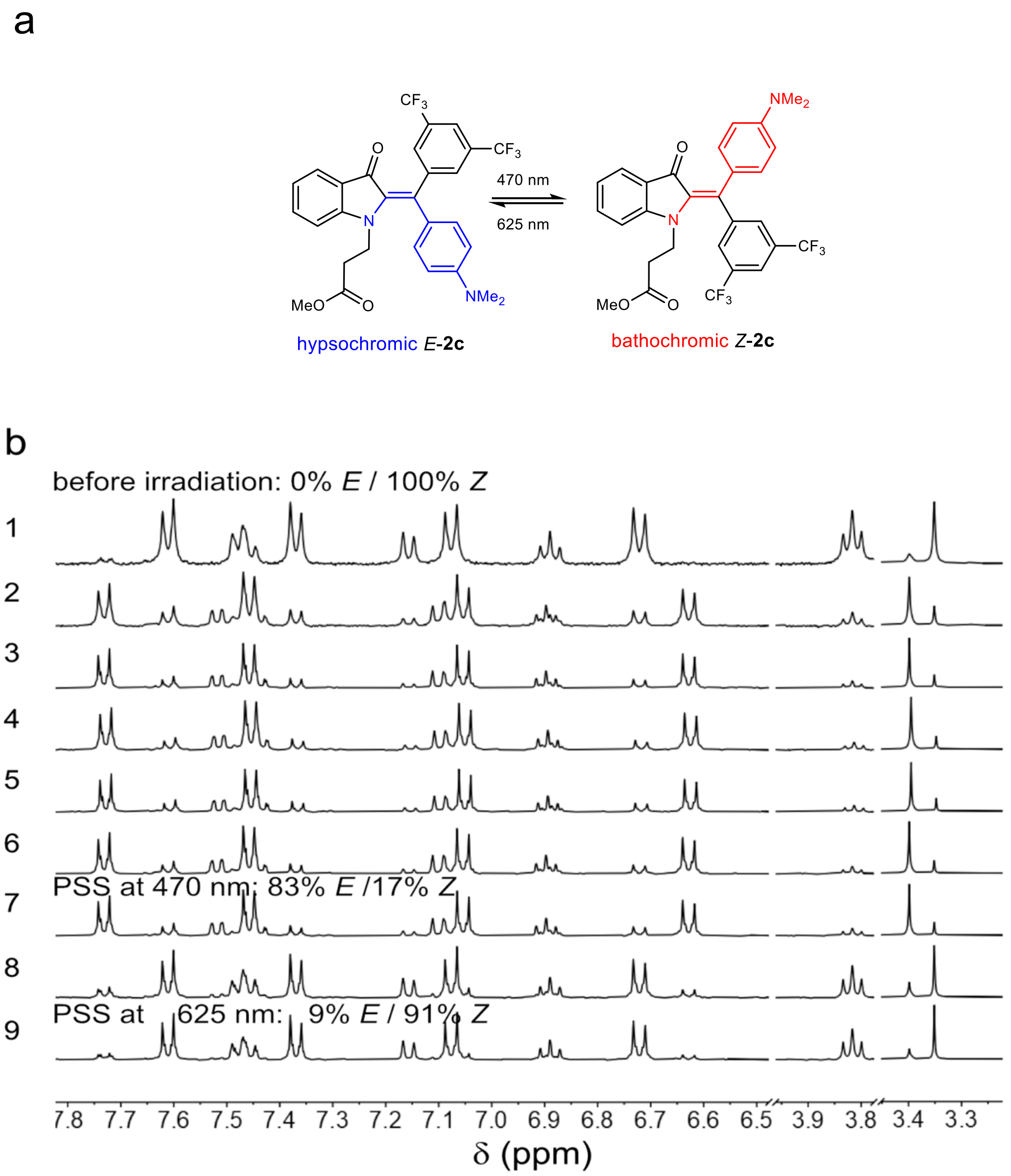

Supplementary Figure 58: a Schematic illustration of the photoisomerization from the hypsochromic to the bathochromic isomer of diaryl-HI **2c** and vice versa. **b** ^{1}H NMR spectra (400 MHz, THF-d_8, 23 °C) 1 – 9 of diaryl-HI **2c** recorded after different irradiation durations using light of different wavelengths at 23 °C. From top to bottom: partial ^{1}H NMR spectrum of diaryl-HI **2c** with isomeric composition of 24% *E* isomer and 76% *Z* isomer after 10 min of irradiation with a 470 nm LED in spectrum 1, progress of *Z* isomer enrichment by irradiating with a 470 nm LED to the pss resulting in an isomer composition of 19% *E* isomer and 81% *Z* isomer in spectrum 7, progress of *E* isomer enrichment by irradiating with a 625 nm LED to the pss with an isomeric composition of 91% *E* isomer and 9% *Z* isomer in spectrum 9. Source data are provided as Source Data File.

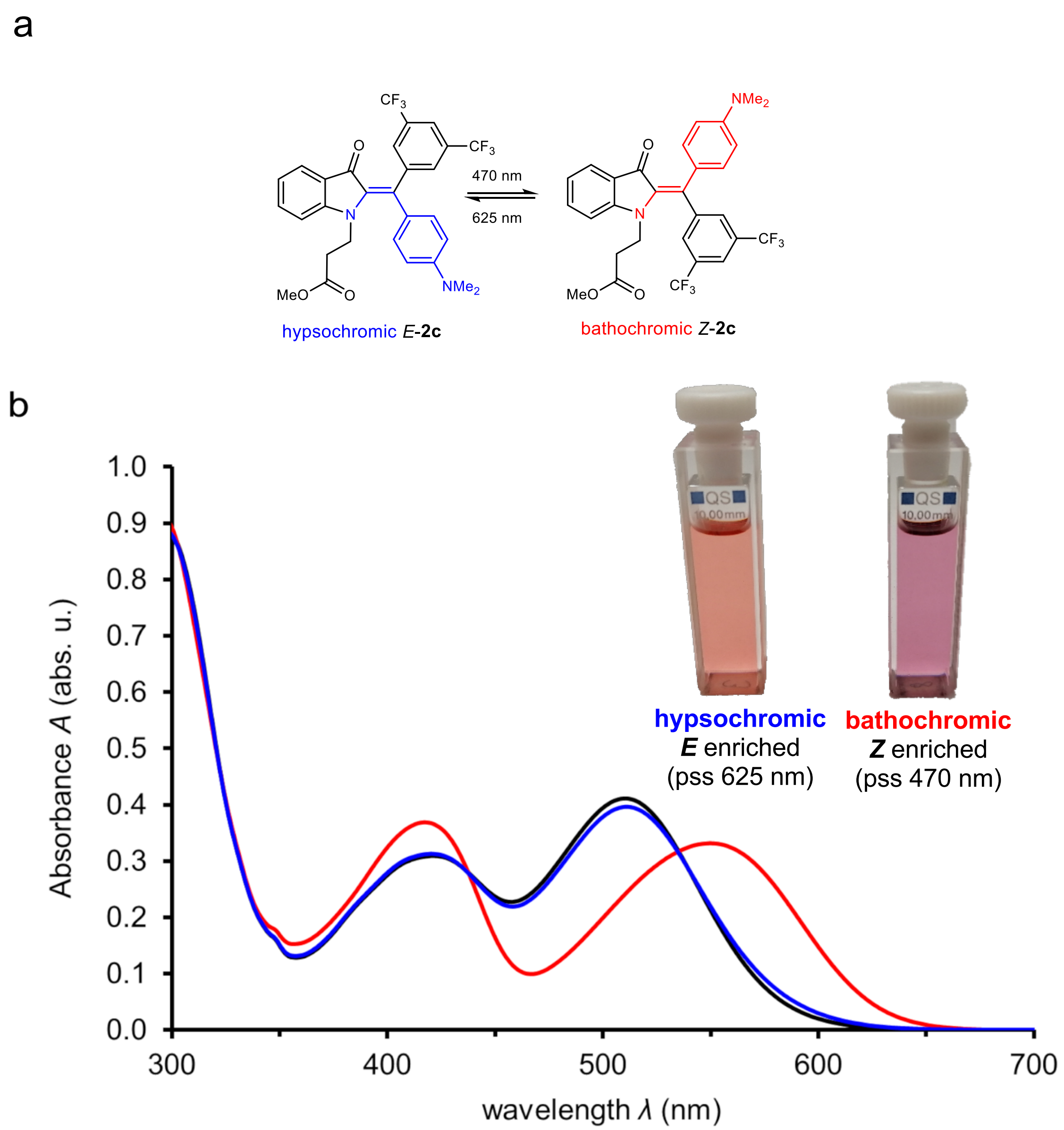

Supplementary Figure 59: **a** Schematic illustration of the photoisomerization from the hypsochromic to the bathochromic isomer of diaryl-HI **2c** and vice versa. **b** Absorption spectra of diaryl-HI **2c** in toluene solution at 23 °C recorded before and after different irradiation durations using light of different wavelengths. Absorbance of a diaryl-HI **2c** solution containing a mixture of *E* and *Z* isomer before irradiation (black line, A_{max} at 510 nm), absorbance of the bathochromic *Z* isomer enriched solution (red line, A_{max} at 550 nm) obtained after irradiation to the pss with 470 nm light, absorbance of the hypsochromic *E* isomer enriched solution (blue line, A_{max} at 511 nm) obtained after irradiation to the pss with 625 nm light. Source data are provided as Source Data File.

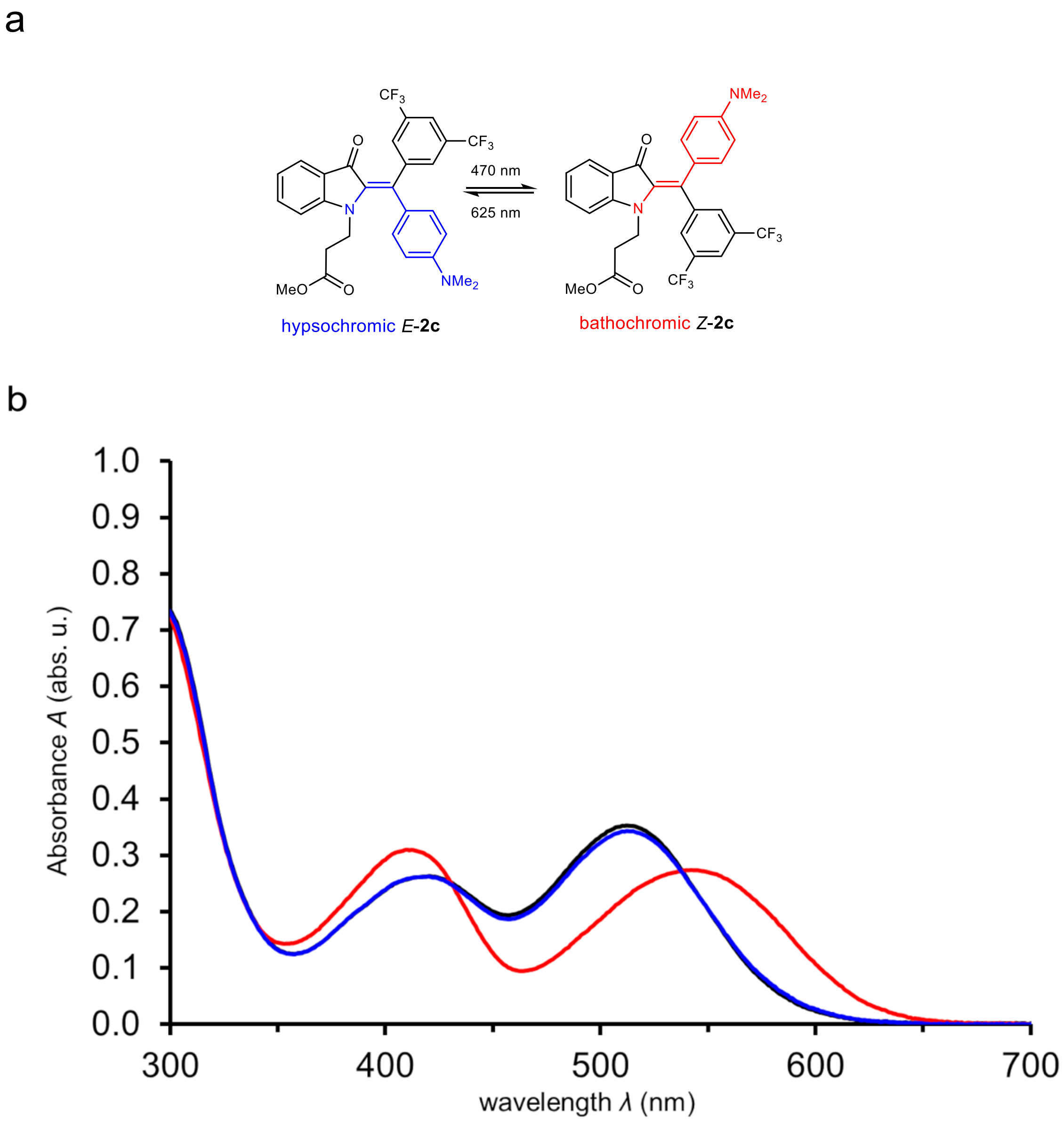

Supplementary Figure 60: **a** Schematic illustration of the photoisomerization from the hypsochromic to the bathochromic isomer of diaryl-HI **2c** and vice versa. **b** Absorption spectra of diaryl-HI **2c** in tetrahydrofuran solution at 23 °C recorded before and after different irradiation durations using light of different wavelengths. Absorbance of a diaryl-HI **2c** solution containing a mixture of *E* and enriched *Z* isomer before irradiation (black line, A_{max} at 512 nm), absorbance of the bathochromic *Z* isomer enriched solution (red line, A_{max} at 542 nm) obtained after irradiation to the pss with 470 nm light, absorbance of the hypsochromic *E* isomer enriched solution (blue line, A_{max} at 512 nm) obtained after irradiation to the pss with 625 nm light. Source data are provided as Source Data File.

a)

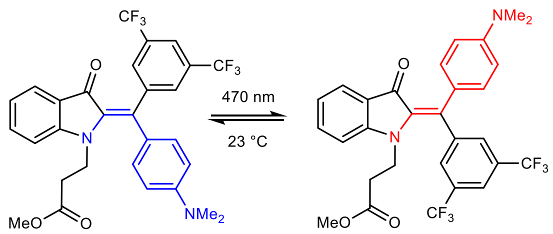

b

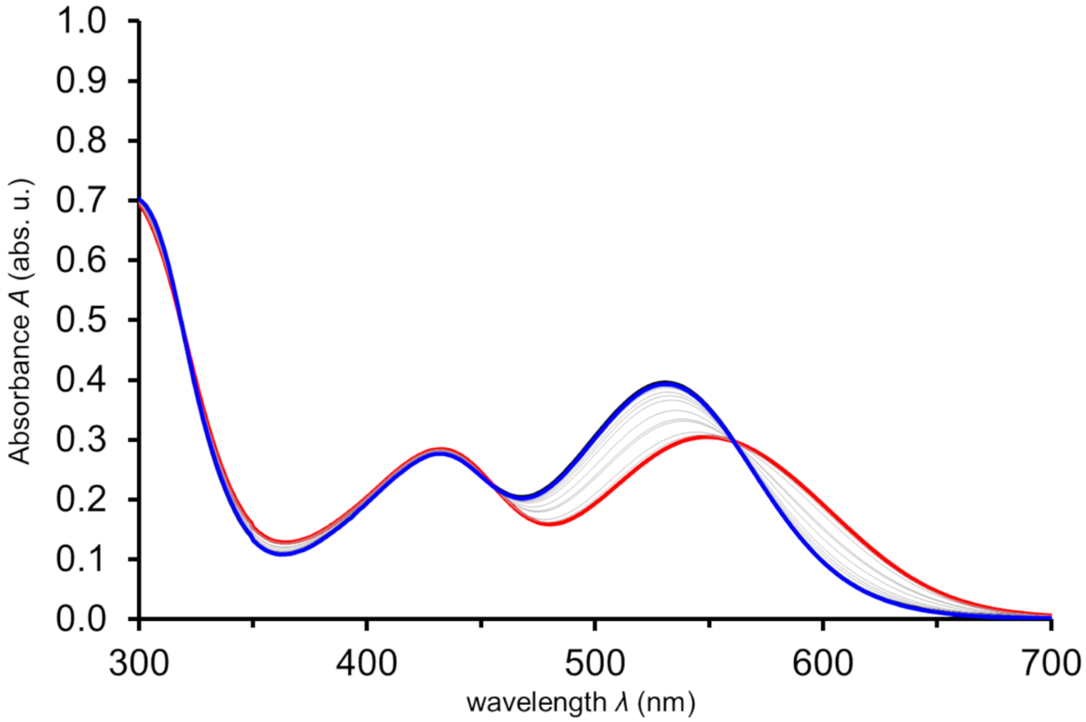

Supplementary Figure 61: a) Schematic illustration of the photoisomerization from the hypsochromic to the bathochromic isomer of diaryl-HI **2c** and vice versa thermal isomerization at 23 °C. **b)** Absorption spectra of diaryl-HI **2c** in methanol solution at 23 °C recorded before and after different irradiation durations using light of 470 nm. Absorbance of a diaryl-HI **2c** solution containing a mixture of *E* and *Z* isomer before irradiation (black line, A_{max} at 531 nm), absorbance of the bathochromic *Z* isomer enriched solution (red line, A_{max} at 549 nm) obtained after irradiation to the pss with 470 nm light, absorbance of the hypsochromic *E* isomer enriched solution (grey lines and blue line with A_{max} at 530 nm) obtained after different times of thermal isomerization at 23 °C beginning with bathochromic *Z* isomer enriched solution. The thermal bistability is decreased in methanol compared to tetrahydrofuran, toluene or *o*-xylene. Source data are provided as Source Data File.

a

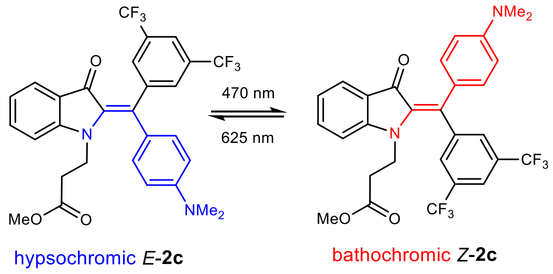

b

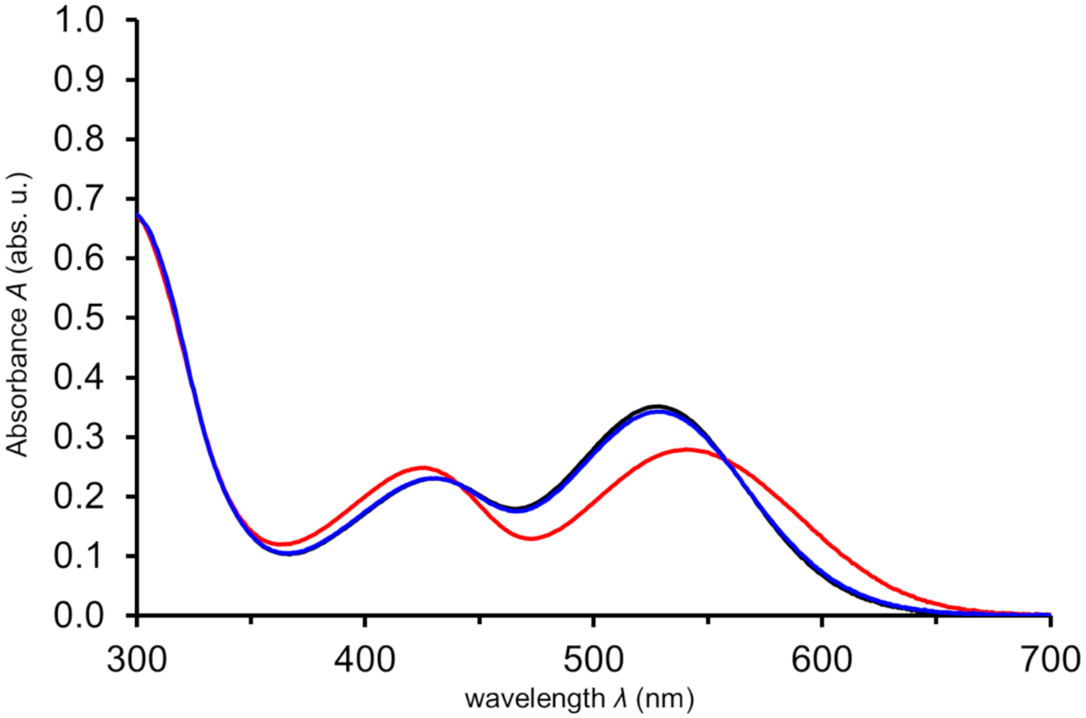

Supplementary Figure 62: a Schematic illustration of the photoisomerization from the hypsochromic to the bathochromic isomer of diaryl-HI **2c** and vice versa. **b** Absorption spectra of diaryl-HI **2c** in DMSO solution at 23 °C recorded before and after different irradiation durations using light of different wavelengths. Absorbance of a diaryl-HI **2c** solution containing a mixture of *E* and enriched *Z* isomer before irradiation (black line, A_{max} at 528 nm), absorbance of the bathochromic *Z* isomer enriched solution (red line, A_{max} at 540 nm) obtained after irradiation to the pss with 470 nm light, absorbance of the hypsochromic *E* isomer enriched solution (blue line, A_{max} at 527 nm) obtained after irradiation to the pss with 625 nm light. Irradiation with 660 nm light does not show an effect at a similar timescale. No effect of decreased thermal stability was noticed. Source data are provided as Source Data File.

5.7 Photoisomerization of diaryl-HI 3a

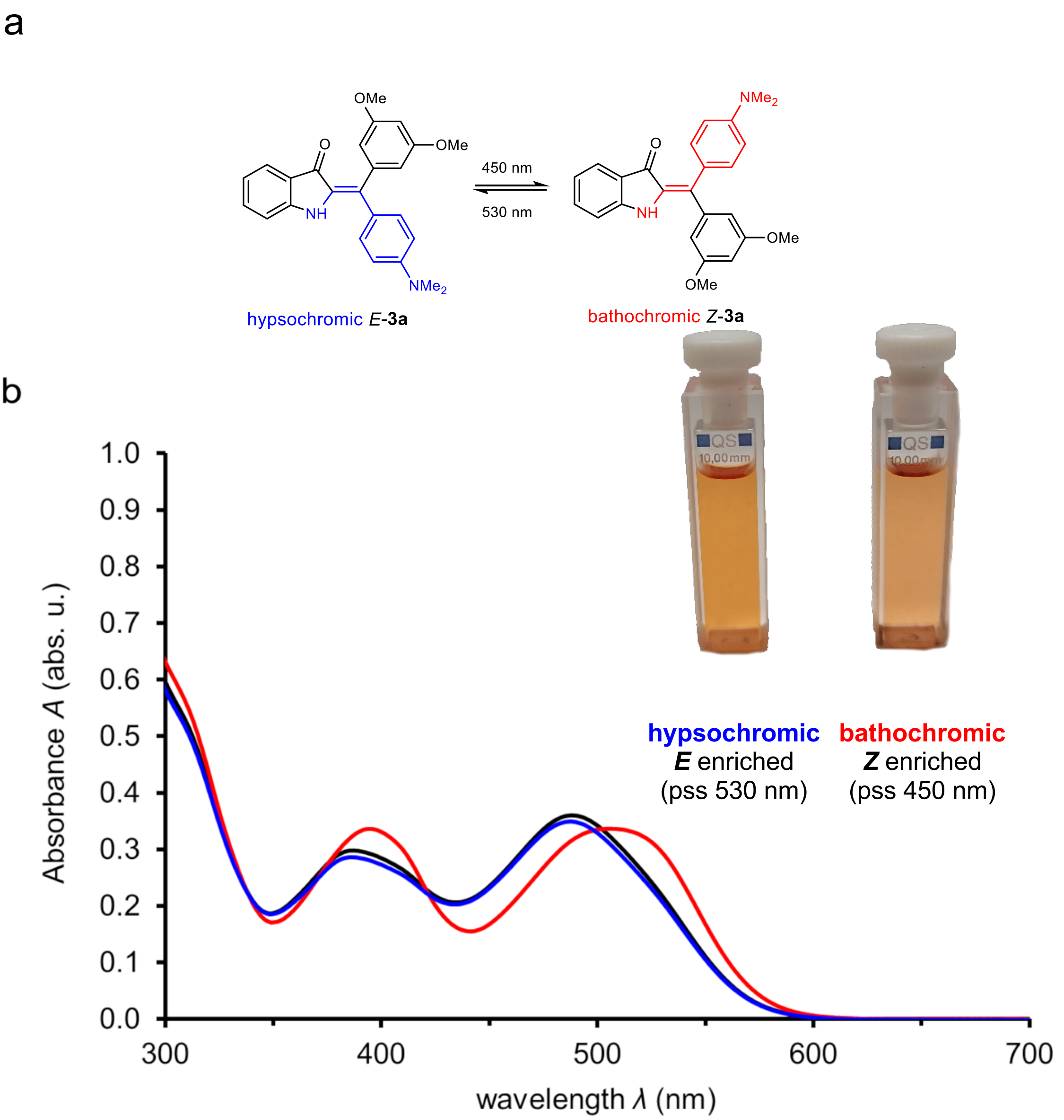

Supplementary Figure 63: a Schematic illustration of the photoisomerization from the hypsochromic to the bathochromic isomer of diaryl-HI **3a** and vice versa. **b** Absorption spectra of diaryl-HI **3a** in toluene solution at 23 °C recorded before and after different irradiation durations using light of different wavelengths. Absorbance of a diaryl-HI **3a** solution containing a mixture of *E* and *Z* isomer before irradiation (black line, A_{max} at 488 nm), absorbance of the bathochromic *Z* isomer enriched solution (red line, A_{max} at 506 nm) obtained after irradiation to the pss with 450 nm light, absorbance of the hypsochromic *E* isomer enriched solution (blue line, A_{max} at 488 nm) obtained after irradiation to the pss with 530 nm light. The hypsochromic *E* isomer enriched solution (blue line) shows already photodegradation (shift of isosbestic point). Source data are provided as Source Data File.

5.8 Photoisomerization of diaryl-HI 3b

a

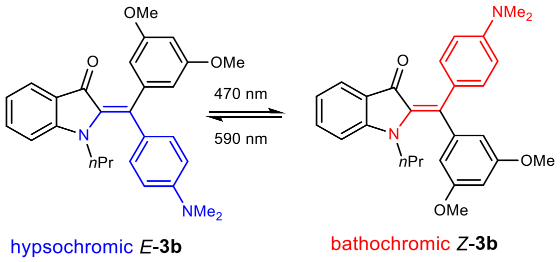

b

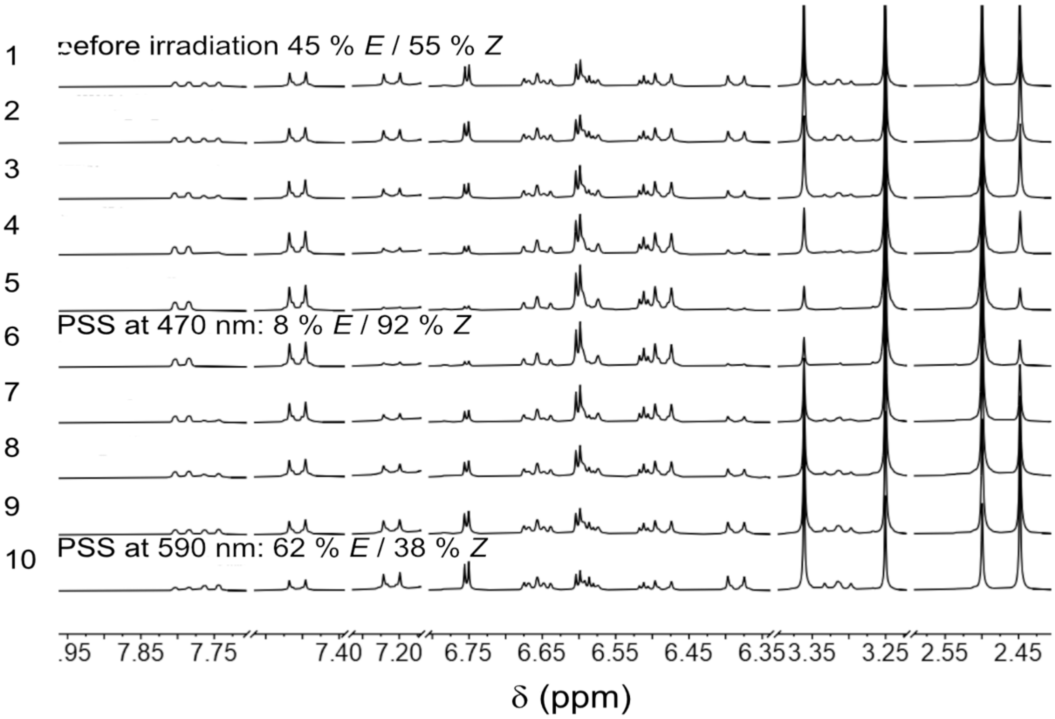

Supplementary Figure 64: a Schematic illustration of the photoisomerization from the hypsochromic to the bathochromic isomer of diaryl-HI **3b** and vice versa. **b** ^{1}H NMR spectra (400 MHz, toluene-d_8, 23 °C) 1 – 10 of diaryl-HI **3b** recorded after different irradiation durations using light of different wavelengths at 23 °C. From 1 - 10: partial ^{1}H NMR spectrum of diaryl-HI **3b** with isomeric composition of 45% *E* isomer and 55% *Z* isomer before irradiation in spectrum 1, progress of *Z* isomer enrichment by irradiating with a 470 nm LED to the pss resulting in an isomer composition of 8% *E* isomer and 92% *Z* isomer in spectrum 6, progress of *E* isomer enrichment by irradiating with a 590 nm LED to the pss with an isomeric composition of 62% *E* isomer and 38% *Z* isomer in spectrum 10. Source data are provided as Source Data File.

a

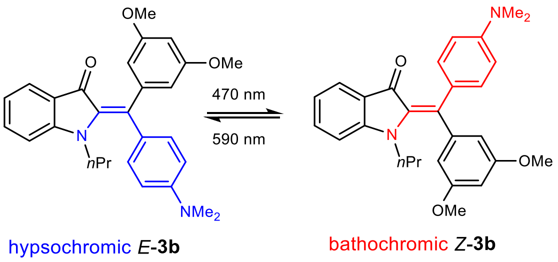

b

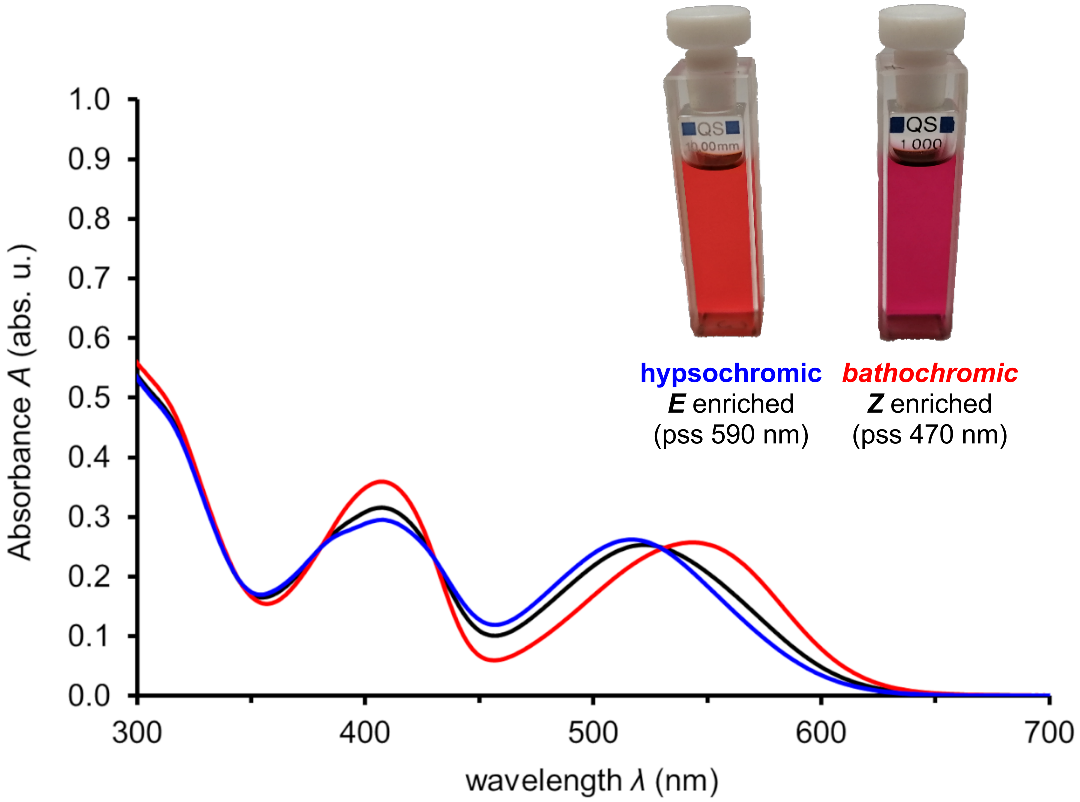

Supplementary Figure 65: a Schematic illustration of the photoisomerization from the hypsochromic to the bathochromic isomer of diaryl-HI **3b** and vice versa. **b** Absorption spectra of diaryl-HI **3b** in toluene solution at 23 °C recorded before and after different irradiation durations using light of different wavelengths. Absorbance of a diaryl-HI **3b** solution containing a mixture of *E* and *Z* isomer before irradiation (black line, A_{max} at 522 nm), absorbance of the bathochromic *Z* isomer enriched solution (red line, A_{max} at 543 nm) obtained after irradiation to the pss with 470 nm light, absorbance of the hypsochromic *E* isomer enriched solution (blue line, A_{max} at 517 nm) obtained after irradiation to the pss with 590 nm light. Source data are provided as Source Data File.

5.9 Photoisomerization of diaryl-HI 3c at NMR concentrations

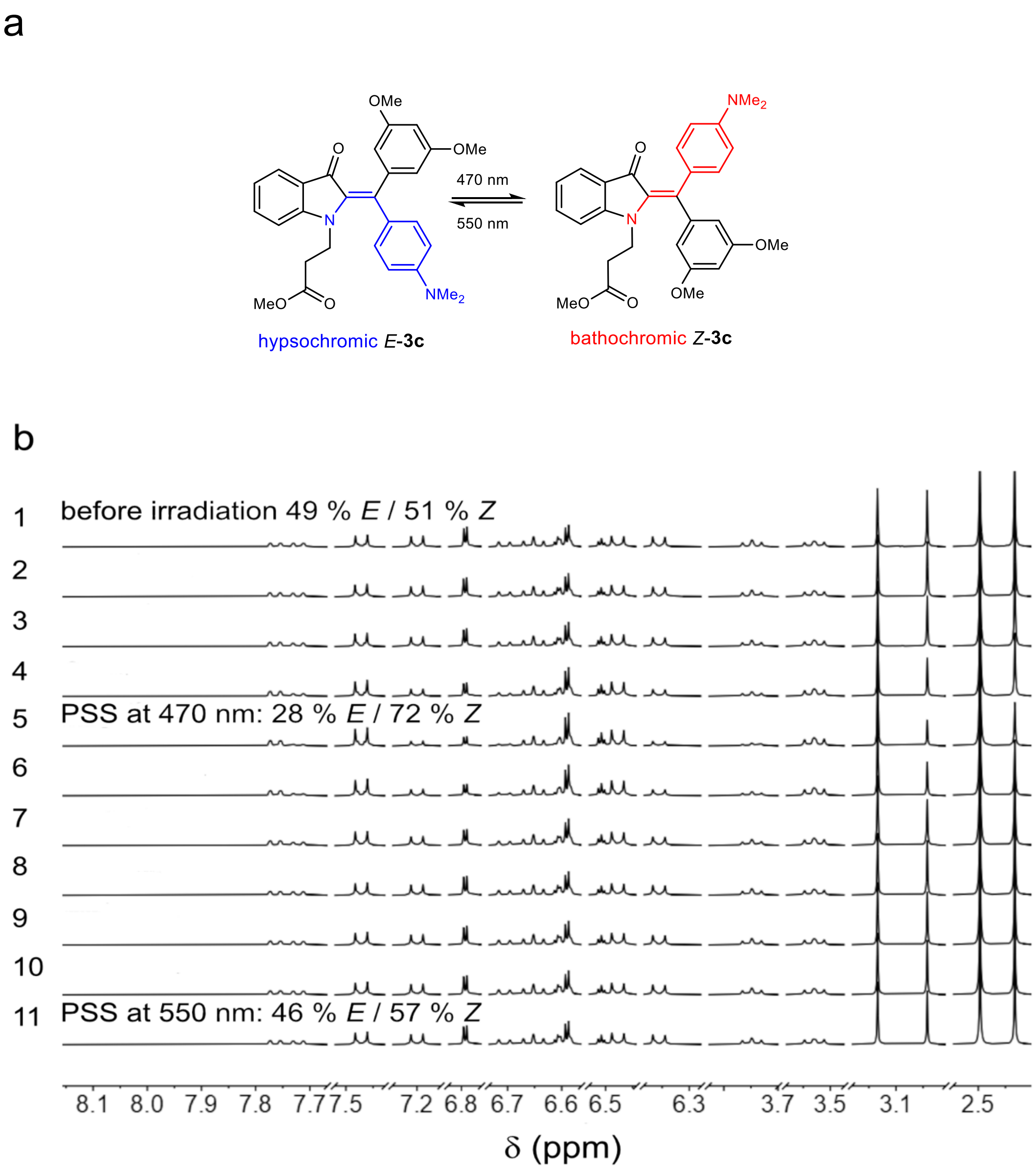

Suplementary Figure 66: a Schematic illustration of the photoisomerization from the hypsochromic to the bathochromic isomer of diaryl-HI **3c** and vice versa. **b** ^{1}H NMR spectra (400 MHz, toluene-d_8, 23 °C) 1 – 11 of diaryl-HI **3c** recorded after different irradiation durations using light of different wavelengths at 23 °C. From 1 - 11: partial ^{1}H NMR spectrum of diaryl-HI **3c** with isomeric composition of 49% *E* isomer and 51% *Z* isomer before irradiation in spectrum 1, progress of *Z* isomer enrichment by irradiating with a 470 nm LED to the pss resulting in an isomer composition of 28% *E* isomer and 72% *Z* isomer in spectrum 5, progress of *E* isomer enrichment by irradiating with a 550 nm LED to the pss with an isomeric composition of 46% *E* isomer and 57% *Z* isomer in spectrum 11. Source data are provided as Source Data File.

a

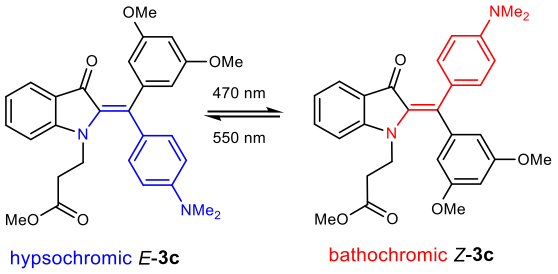

b

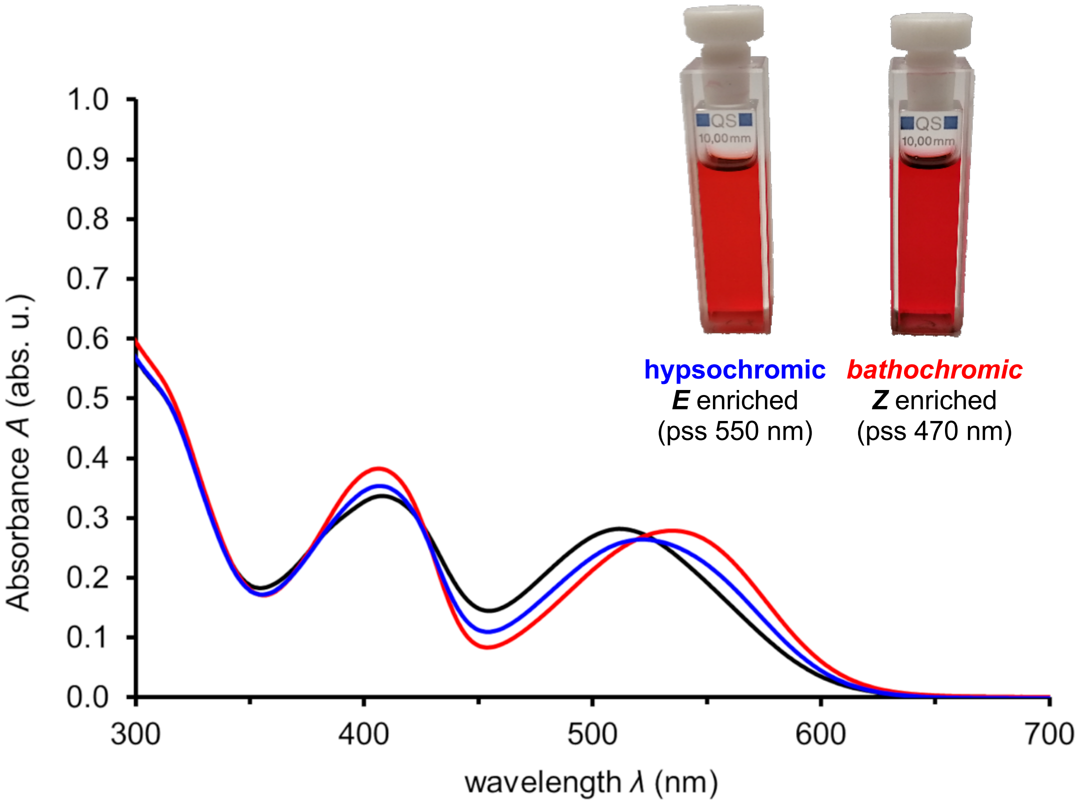

Supplementary Figure 67: a Schematic illustration of the photoisomerization from the hypsochromic to the bathochromic isomer of diaryl-HI **3c** and vice versa. **b** Absorption spectra of diaryl-HI **3c** in toluene solution at 23 °C recorded before and after different irradiation durations using light of different wavelengths. Absorbance of a diaryl-HI **3c** solution containing a mixture of *E* and *Z* isomer before irradiation (black line, A_{max} at 512 nm), absorbance of the bathochromic *Z* isomer enriched solution (red line, A_{max} at 535 nm) obtained after irradiation to the pss with 470 nm light, absorbance of the hypsochromic *E* isomer enriched solution (blue line, A_{max} at 522 nm) obtained after irradiation to the pss with 550 nm light. Source data are provided as Source Data File.

5.10 Photoisomerization of diaryl-HI 4a

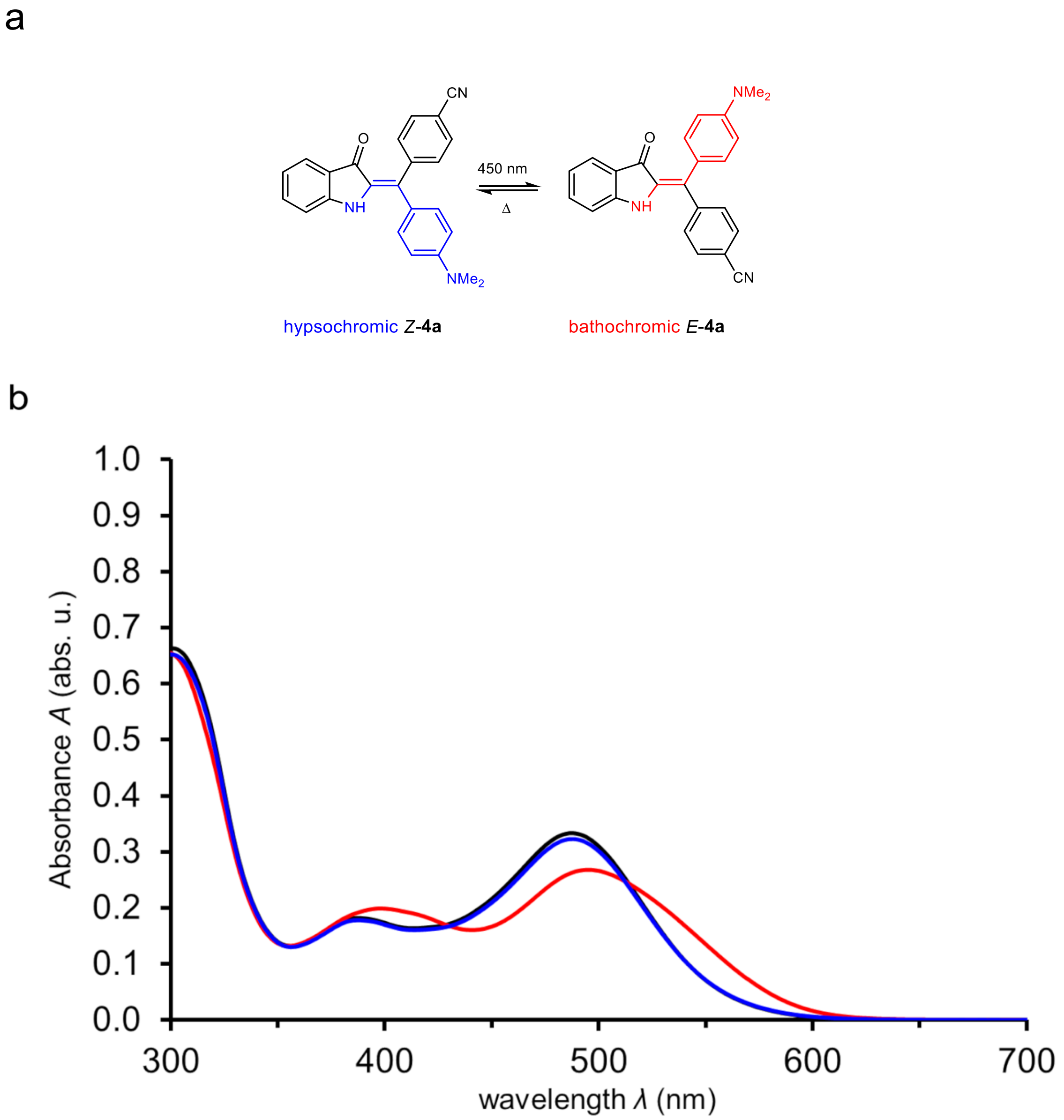

Supplementary Figure 68: **a** Schematic illustration of the photoisomerization from the hypsochromic to the bathochromic isomer of diaryl-HI **4a** and vice versa. **b** Absorption spectra of diaryl-HI **4a** in toluene solution at 23 °C recorded before and after different irradiation durations using light of different wavelengths. Absorbance of a diaryl-HI **4a** solution containing a mixture of *E* and *Z* isomer before irradiation (black line, A_{max} at 488 nm), absorbance of the bathochromic *E* isomer enriched solution (red line, A_{max} at 495 nm) obtained after irradiation to the pss with 450 nm light, absorbance of the hypsochromic *Z* isomer enriched solution (blue line, A_{max} at 488 nm) obtained after thermal *E* to *Z* isomerization at 23 °C of bathochromic *E* isomer enriched solution. Source data are provided as Source Data File.

5.11 Photoisomerization of diaryl-HI 4b

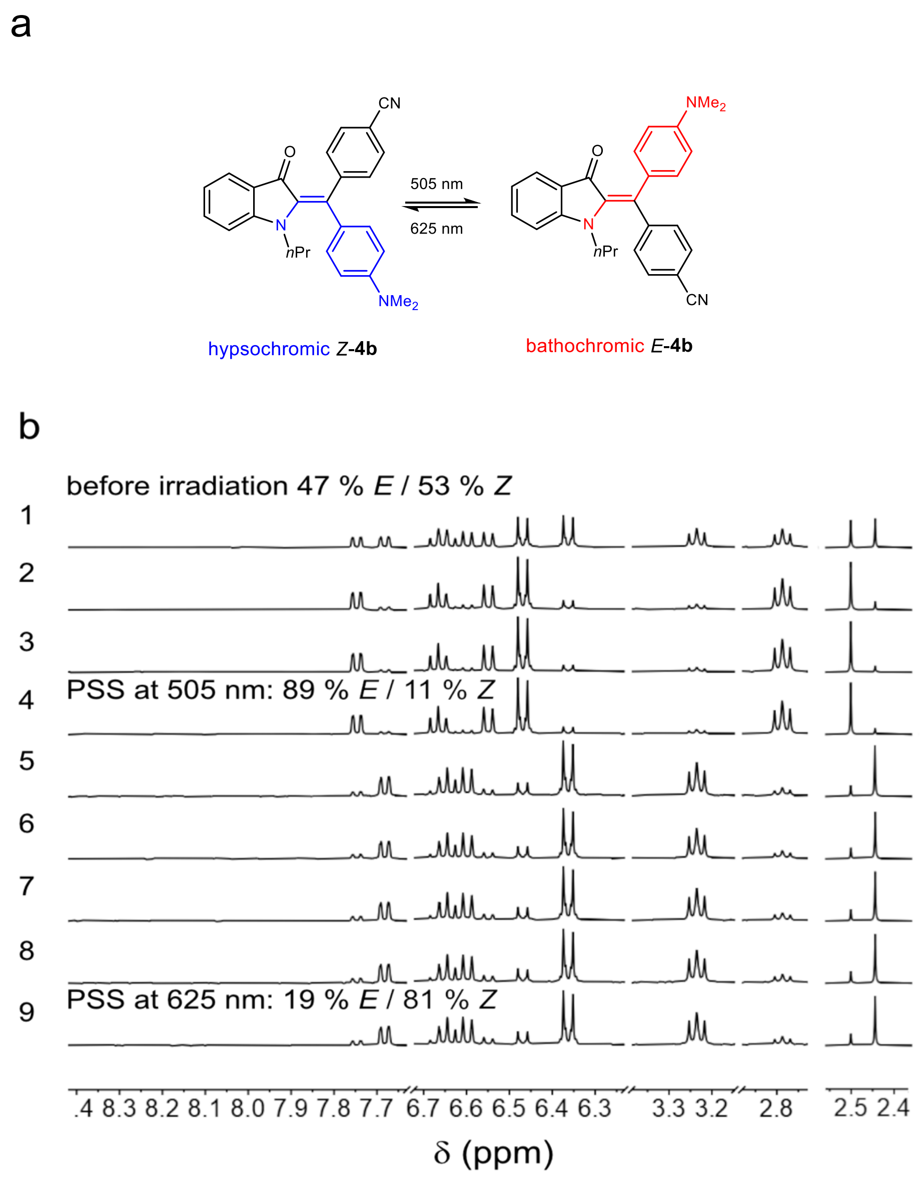

Supplementary Figure 69: a Schematic illustration of the photoisomerization from the hypsochromic to the bathochromic isomer of diaryl-HI **4b** and vice versa. **b** ^{1}H NMR spectra (400 MHz, toluene-d_8, 23 °C) 1 – 9 of diaryl-HI **4b** recorded after different irradiation durations using light of different wavelengths at 23 °C. Magnification varies in the 2.5 ppm section of the partial ^{1}H NMR spectra. From 1 - 9: partial ^{1}H NMR spectrum of diaryl-HI **4b** with isomeric composition of 47% *E* isomer and 53% *Z* isomer before irradiation in spectrum 1, progress of *E* isomer enrichment by irradiating with a 505 nm LED to the pss resulting in an isomer composition of 89% *E* isomer and 11% *Z* isomer in spectrum 4, progress of *Z* isomer enrichment by irradiating with a 625 nm LED to the pss with an isomeric composition of 19% *E* isomer and 81% *Z* isomer in spectrum 9. Source data are provided as Source Data File.

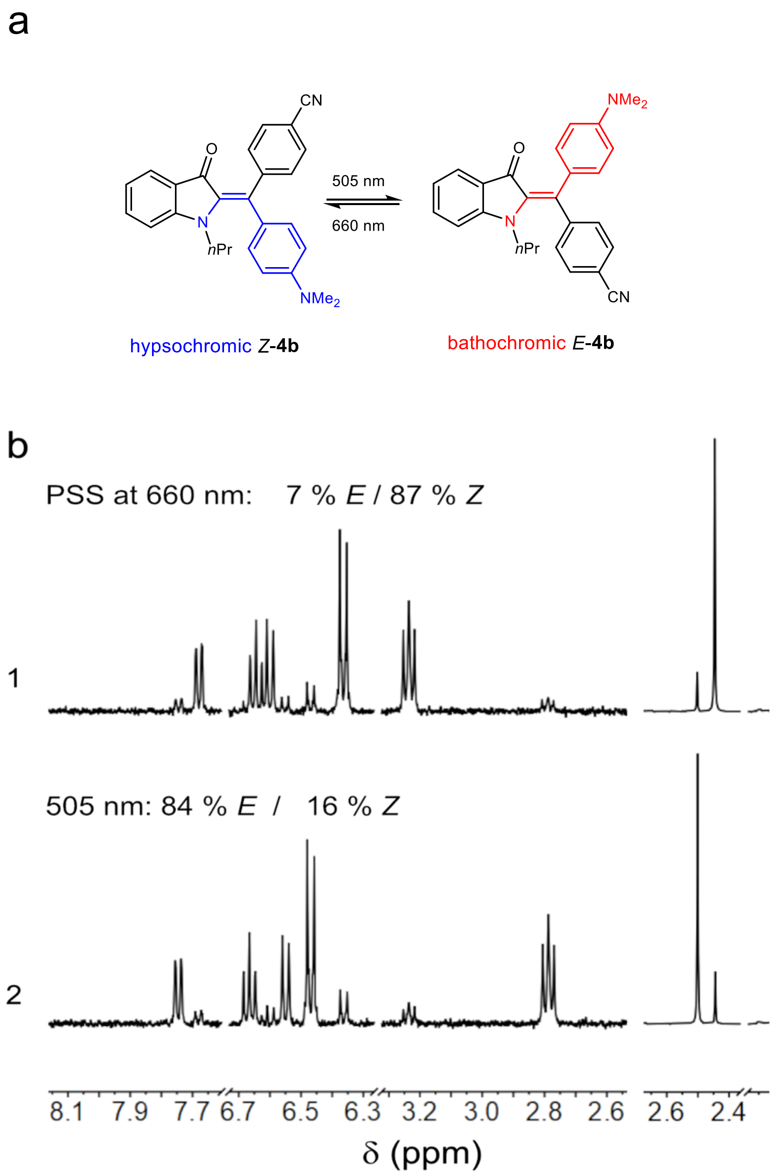

Supplementary Figure 70: a Schematic illustration of the photoisomerization from the hypsochromic to the bathochromic isomer of diaryl-HI **4b**. **b** ^{1}H NMR spectra (400 MHz, toluene-d_8, 23 °C) 1 – 2 of diaryl-HI **4b** recorded after different irradiation durations using light of different wavelengths at 23 °C. Magnification varies in the 2.5 ppm section of the partial ^{1}H NMR spectra. From 1 - 2: partial ^{1}H NMR spectrum of diaryl-HI **4b** with isomeric composition of 7% *E* isomer and 87% *Z* isomer after irradiation with light of 660 nm (pss) in spectrum 1, progress of *E* isomer enrichment by irradiating with a 505 nm LED resulting in an isomer composition of 84% *E* isomer and 16% *Z* isomer in spectrum 2. Source data are provided as Source Data File.

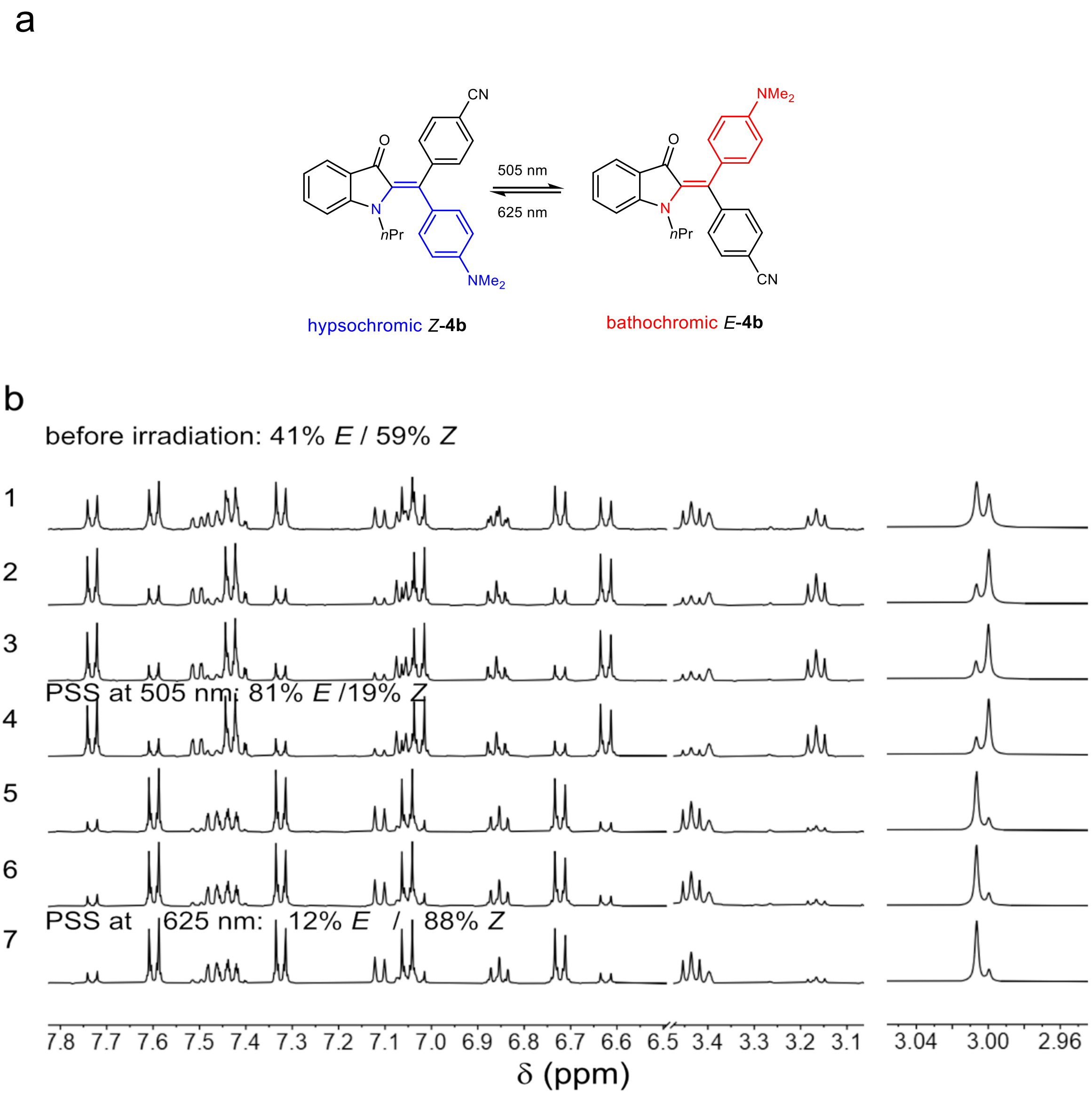

Supplementary Figure 71: a Schematic illustration of the photoisomerization from the hypsochromic to the bathochromic isomer of diaryl-HI **4b** and vice versa. **b** ^{1}H NMR spectra (400 MHz, THF-d_8, 23 °C) 1 – 7 of diaryl-HI **4b** recorded after different irradiation durations using light of different wavelengths at 23 °C. Magnification varies in the 3.04 to 2.96 ppm section of the partial ^{1}H NMR spectra. From 1 – 7: partial ^{1}H NMR spectrum of diaryl-HI**4b** with isomeric composition of 41% *E* isomer and 59% *Z* isomer before irradiation in spectrum 1, progress of *E* isomer enrichment by irradiating with a 505 nm LED to the pss resulting in an isomer composition of 81% *E* isomer and 19% *Z* isomer in spectrum 4, progress of *Z* isomer enrichment by irradiating with a 625 nm LED to the pss with an isomeric composition of 12% *E* isomer and 88% *Z* isomer in spectrum 7. Source data are provided as Source Data File.

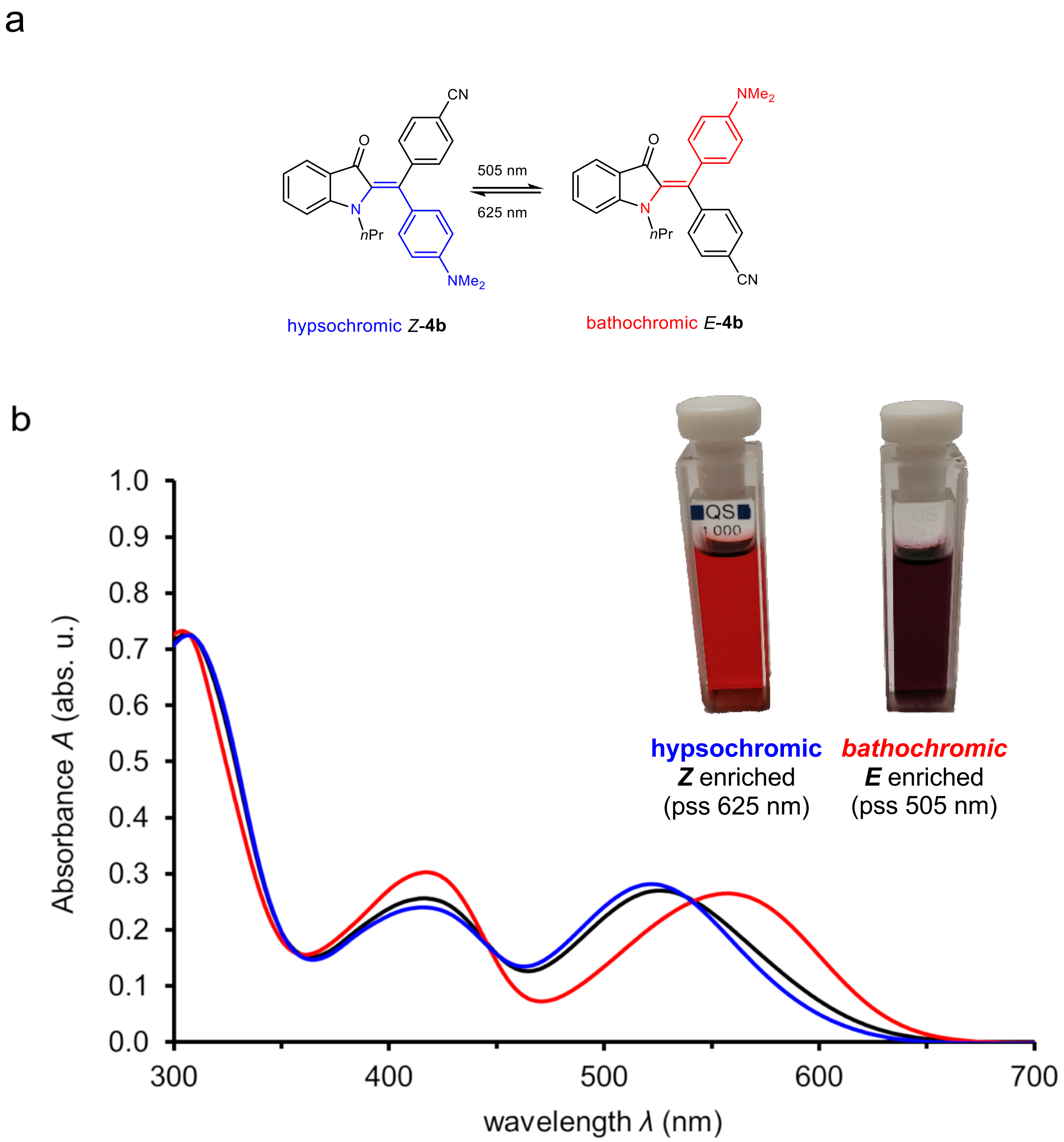

Supplementary Figure 72: a Schematic illustration of the photoisomerization from the hypsochromic to the bathochromic isomer of diaryl-HI **4b** and vice versa. **b** Absorption spectra of diaryl-HI **4b** in toluene solution at 23 °C recorded before and after different irradiation durations using light of different wavelengths. Absorbance of a diaryl-HI **4b** solution containing a mixture of *E* and *Z* isomer before irradiation (black line, A_{max} at 525 nm), absorbance of the bathochromic *E* isomer enriched solution (red line, A_{max} at 558 nm) obtained after irradiation to the pss with 505 nm light, absorbance of the hypsochromic *Z* isomer enriched solution (blue line, A_{max} at 522 nm) obtained after irradiation to the pss with 625 nm light. Source data are provided as Source Data File.

a

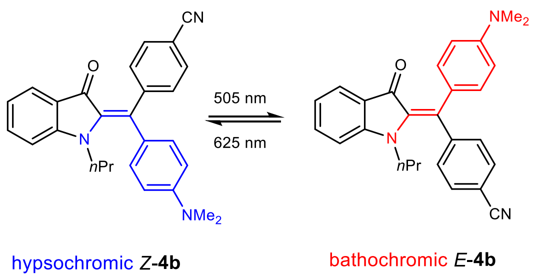

b

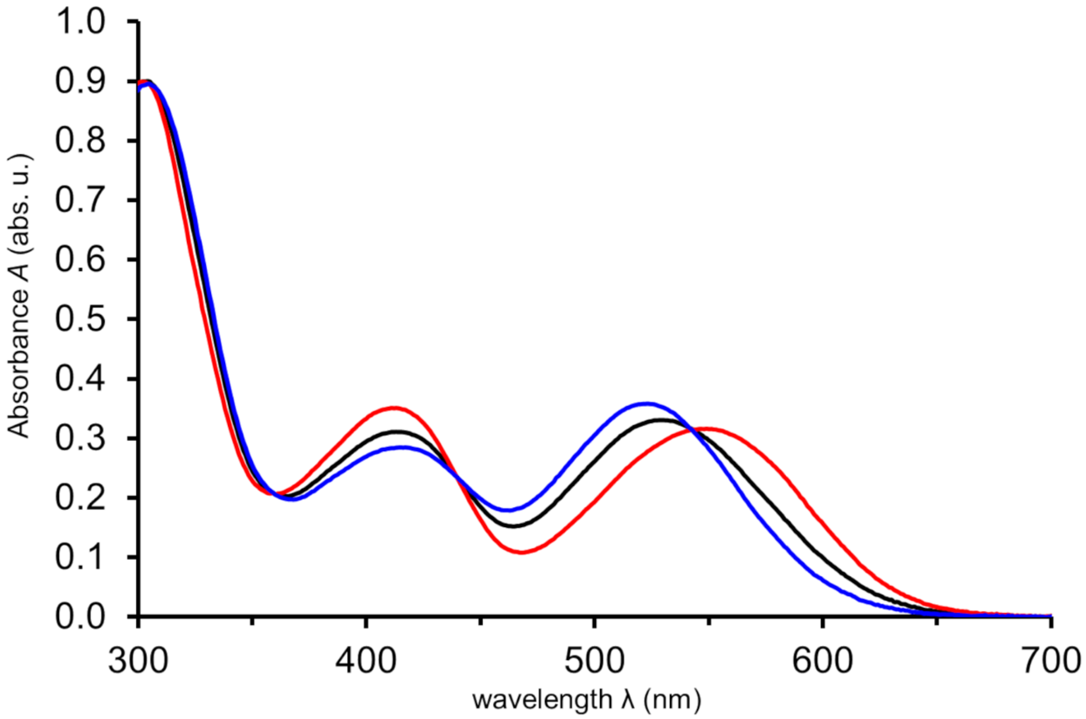

Supplementary Figure 73: a Schematic illustration of the photoisomerization from the hypsochromic to the bathochromic isomer of diaryl-HI **4b** and vice versa. **b** Absorption spectra of diaryl-HI **4b** in tetrahydrofuran solution at 23 °C recorded before and after different irradiation durations using light of different wavelengths. Absorbance of a diaryl-HI **4b** solution containing a mixture of *E* and *Z* isomer before irradiation (black line, A_{max} at 530 nm), absorbance of the bathochromic *E* isomer enriched solution (red line, A_{max} at 549 nm) obtained after irradiation to the pss with 505 nm light, absorbance of the hypsochromic *Z* isomer enriched solution (blue line, A_{max} at 523 nm) obtained after irradiation to the pss with 625 nm light. The solution of the enriched bathochromic *E* isomer did not undergo thermal induced changes at 23 °C for at least 1 h. Source data are provided as Source Data File.

a

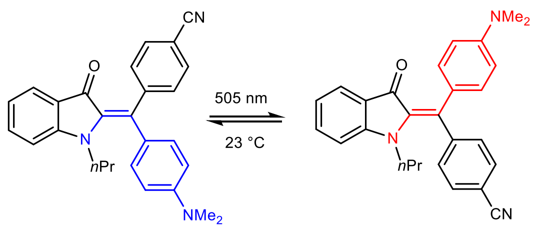

b

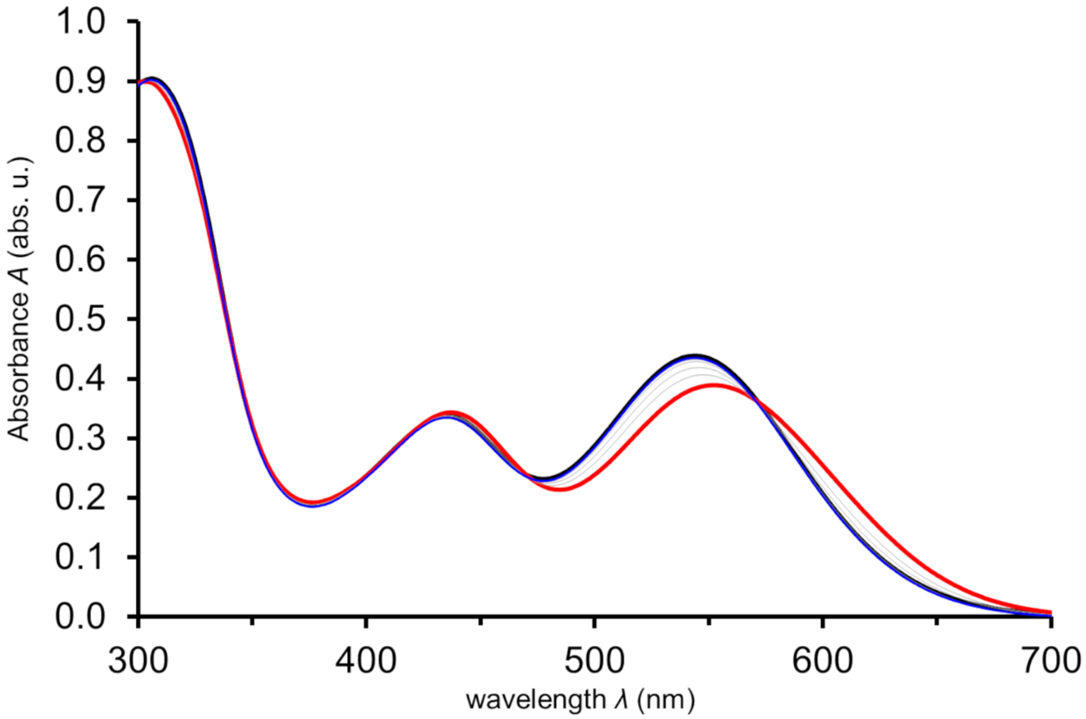

Supplementary Figure 74: a Schematic illustration of the photoisomerization from the hypsochromic to the bathochromic isomer of diaryl-HI **4b** and vice versa thermal isomerization at 23 °C. **b** Absorption spectra of diaryl-HI **4b** in methanol solution at 23 °C recorded before and after different irradiation durations using light of 505 nm. Absorbance of a diaryl-HI **4b** solution containing a mixture of *E* and *Z* isomer before irradiation (black line, A_{max} at 543 nm), absorbance of the bathochromic *E* isomer enriched solution (red line, A_{max} at 552 nm) obtained after irradiation to the pss with 505 nm light, absorbance of the hypsochromic *Z* isomer enriched solution (grey lines and blue line with, A_{max} at 544 nm) obtained after different times of thermal isomerization at 23 °C beginning with bathochromic *E* isomer enriched solution. The thermal bistability is decreased in methanol solution compared to tetrahydrofuran, toluene, or *o*-xylene. Source data are provided as Source Data File.

5.12 Photoisomerization of diaryl-HI 4c

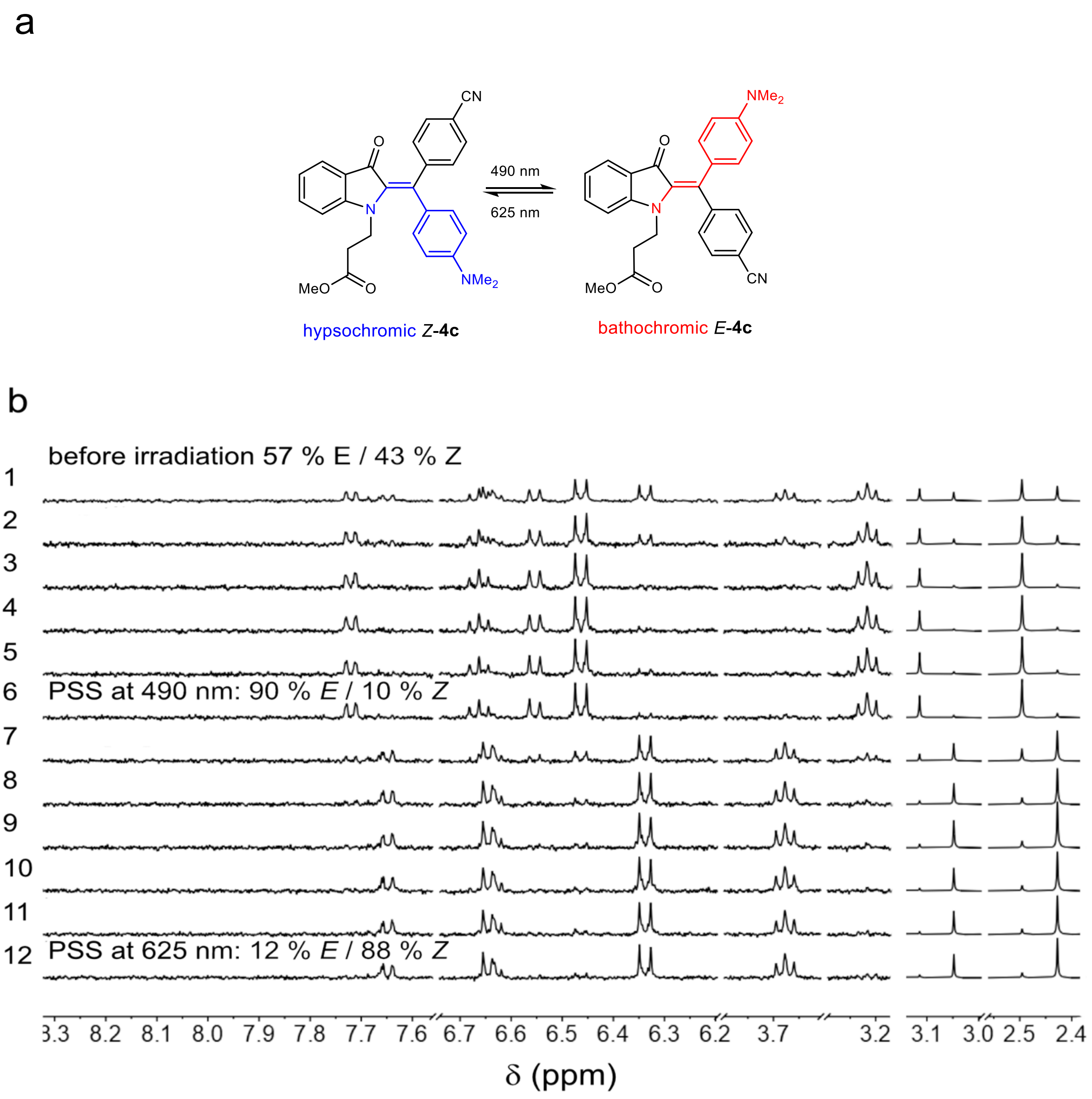

Supplementary Figure 75: a Schematic illustration of the photoisomerization from the hypsochromic to the bathochromic isomer of diaryl-HI **4c** and vice versa. **b** ^{1}H NMR spectra (400 MHz, toluene-d_8, 23 °C) 1 – 12 of diaryl-HI **4c** recorded after different irradiation durations using light of different wavelengths at 23 °C. Magnification varies in the 3.1 to 2.4 ppm section of the partial ^{1}H NMR spectra. From 1 - 12: partial ^{1}H NMR spectrum of diaryl-HI **4c** with isomeric composition of 57% *E* isomer and 43% *Z* isomer before irradiation in spectrum 1, progress of *E* isomer enrichment by irradiating with a 490 nm LED to the pss resulting in an isomer composition of 90% *E* isomer and 10% *Z* isomer in spectrum 6, progress of *Z* isomer enrichment by irradiating with a 625 nm LED to the pss with an isomeric composition of 12% *E* isomer and 88% *Z* isomer in spectrum 12. Source data are provided as Source Data File.

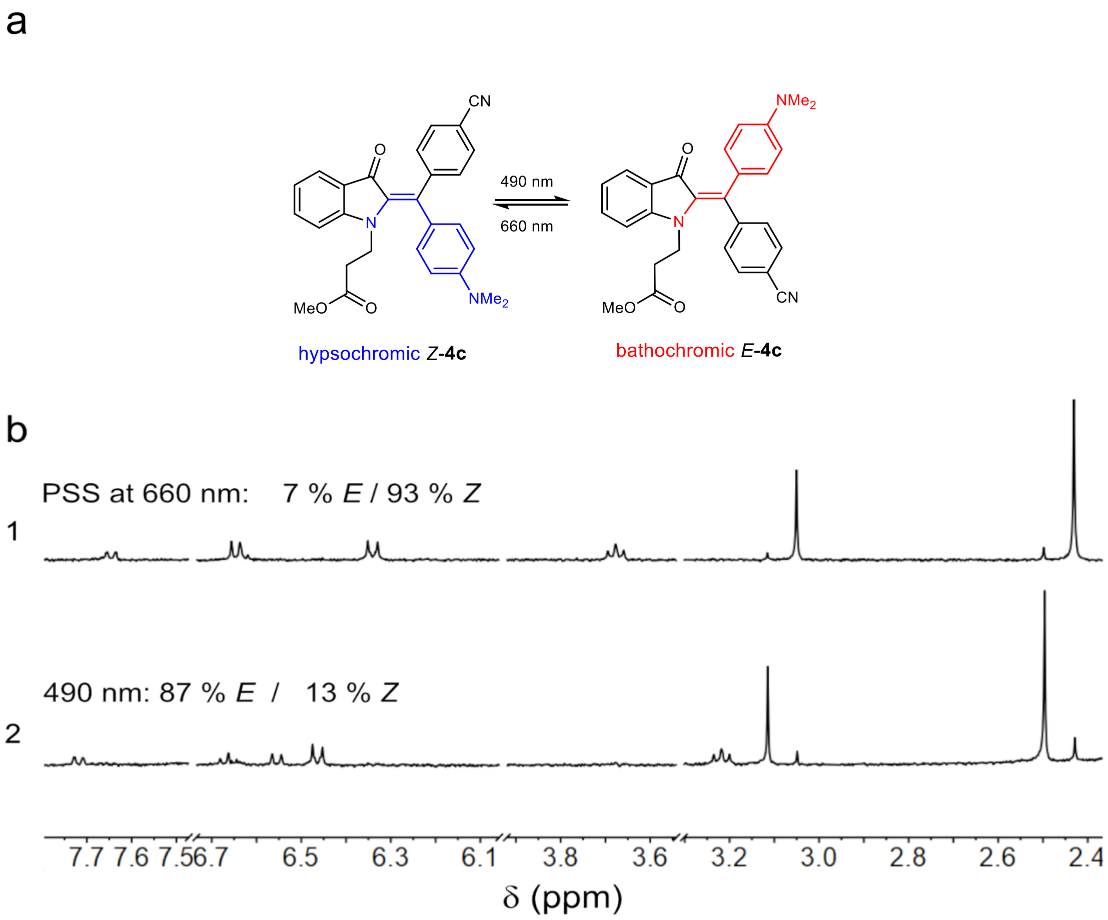

Supplementary Figure 76: a Schematic illustration of the photoisomerization from the hypsochromic to the bathochromic isomer of diaryl-HI **4c**. **b** ^{1}H NMR spectra (400 MHz, toluene-d_8, 23 °C) 1 – 2 of diaryl-HI **4c** recorded after different irradiation durations using light of different wavelengths at 23 °C. From 1 - 2: partial ^{1}H NMR spectrum of diaryl-HI **4c** with isomeric composition of 7% *E* isomer and 93% *Z* isomer after irradiation with light of 660 nm (pss) in spectrum 1, progress of *E* isomer enrichment by irradiating with a 490 nm LED resulting in an isomer composition of 90% *E* isomer and 10% *Z* isomer in spectrum 2. Source data are provided as Source Data File.

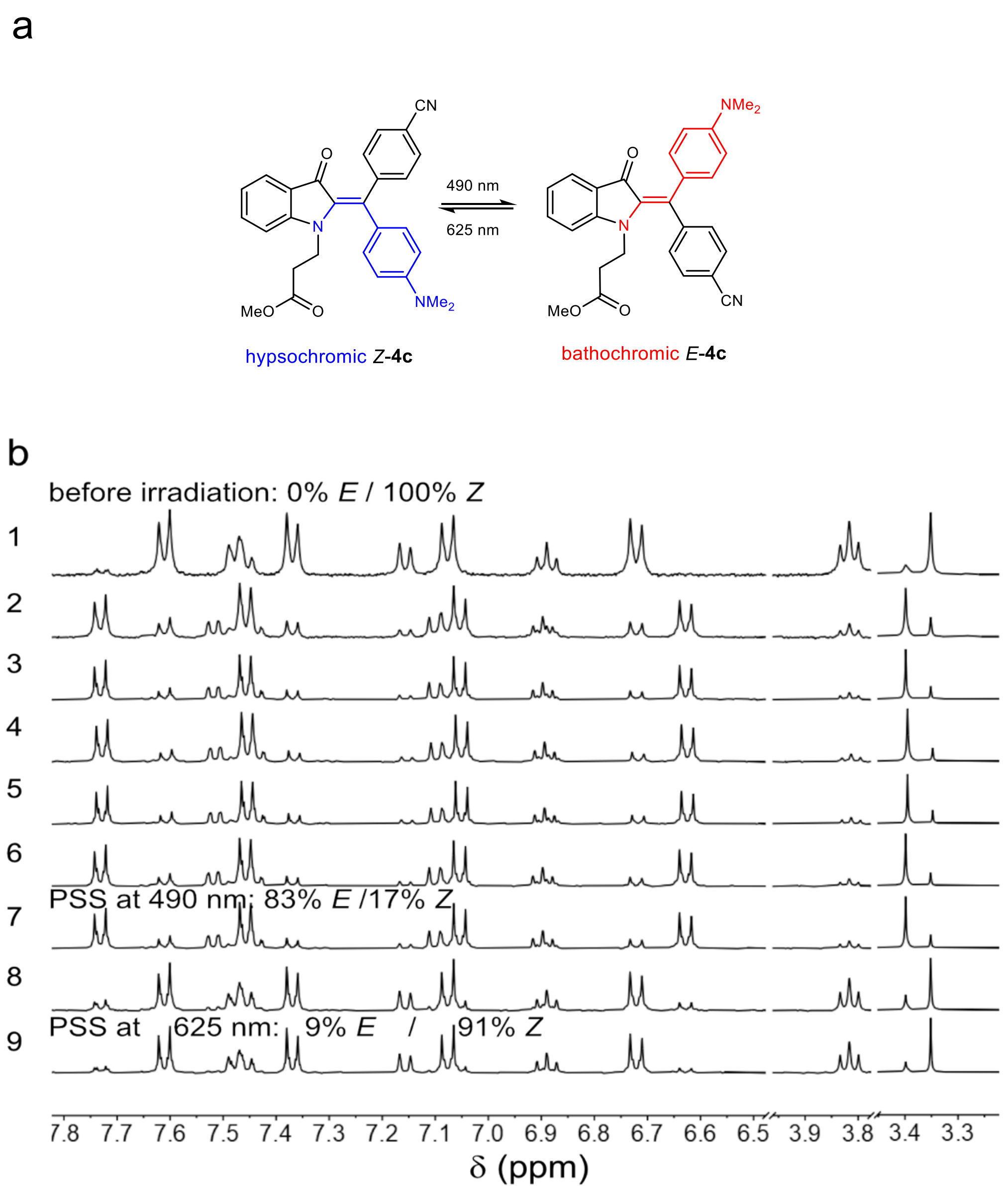

Supplementary Figure 77: a Schematic illustration of the photoisomerization from the hypsochromic to the bathochromic isomer of diaryl-HI **4c** and vice versa. **b** ^{1}H NMR spectra (400 MHz, THF-d_8, 23 °C) 1 – 9 of diaryl-HI **4c** recorded after different irradiation durations using light of different wavelengths at 23 °C. Magnification varies in the 3.4 to 3.2 ppm section of the partial ^{1}H NMR spectra. From 1 - 9: partial ^{1}H NMR spectrum of diaryl-HI **4c** with isomeric composition of 0% *E* isomer and 100% *Z* isomer before irradiation in spectrum 1, progress of *E* isomer enrichment by irradiating with a 490 nm LED to the pss resulting in an isomer composition of 83% *E* isomer and 17% *Z* isomer in spectrum 7, progress of *Z* isomer enrichment by irradiating with a 625 nm LED to the pss with an isomeric composition of 9% *E* isomer and 91% *Z* isomer in spectrum 9. Source data are provided as Source Data File.

a

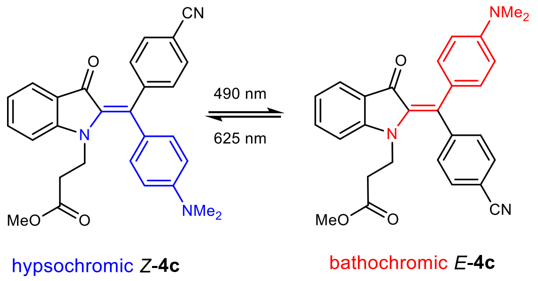

b

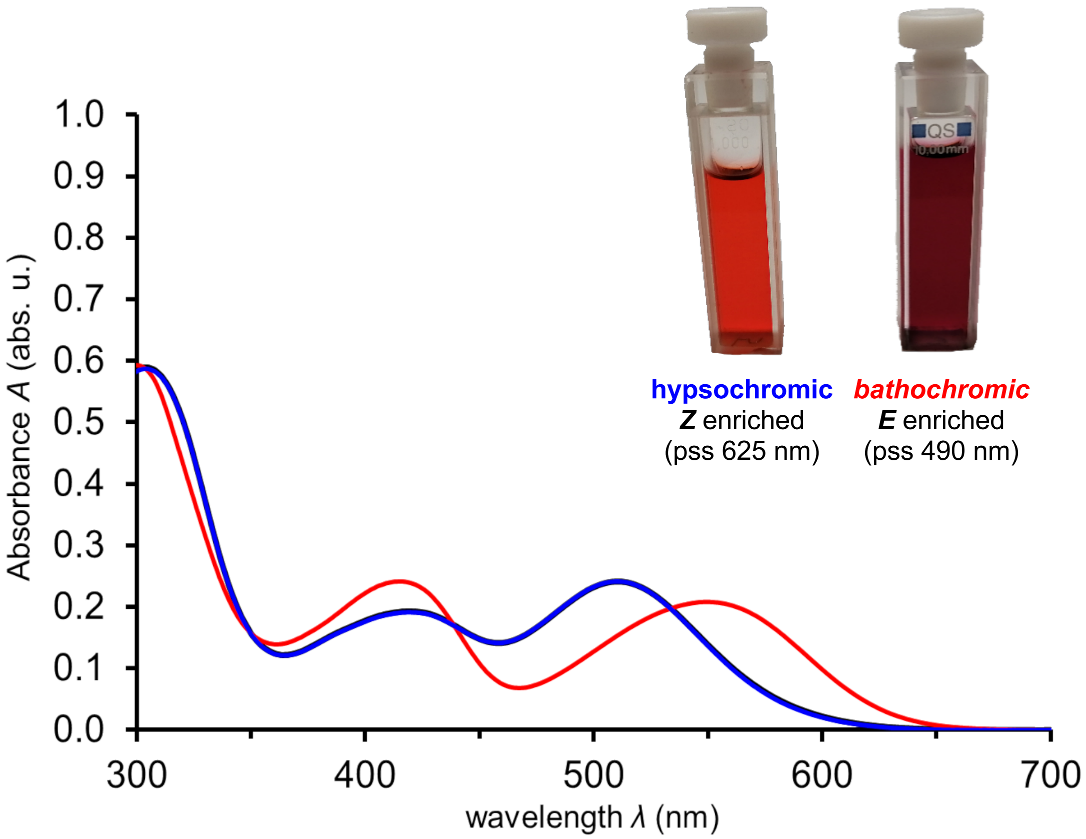

Supplementary Figure 78: a Schematic illustration of the photoisomerization from the hypsochromic to the bathochromic isomer of diaryl-HI **4c** and vice versa. **b** Absorption spectra of diaryl-HI **4c** in toluene solution at 23 °C recorded before and after different irradiation durations using light of different wavelengths. Absorbance of a diaryl-HI **4c** solution containing a mixture of *E* and *Z* isomer before irradiation (black line, A_{max} at 510 nm), absorbance of the bathochromic *E* isomer enriched solution (red line, A_{max} at 548 nm) obtained after irradiation to the pss with 490 nm light, absorbance of the hypsochromic *Z* isomer enriched solution (blue line, A_{max} at 510 nm) obtained after irradiation to the pss with 625 nm light. Source data are provided as Source Data File.

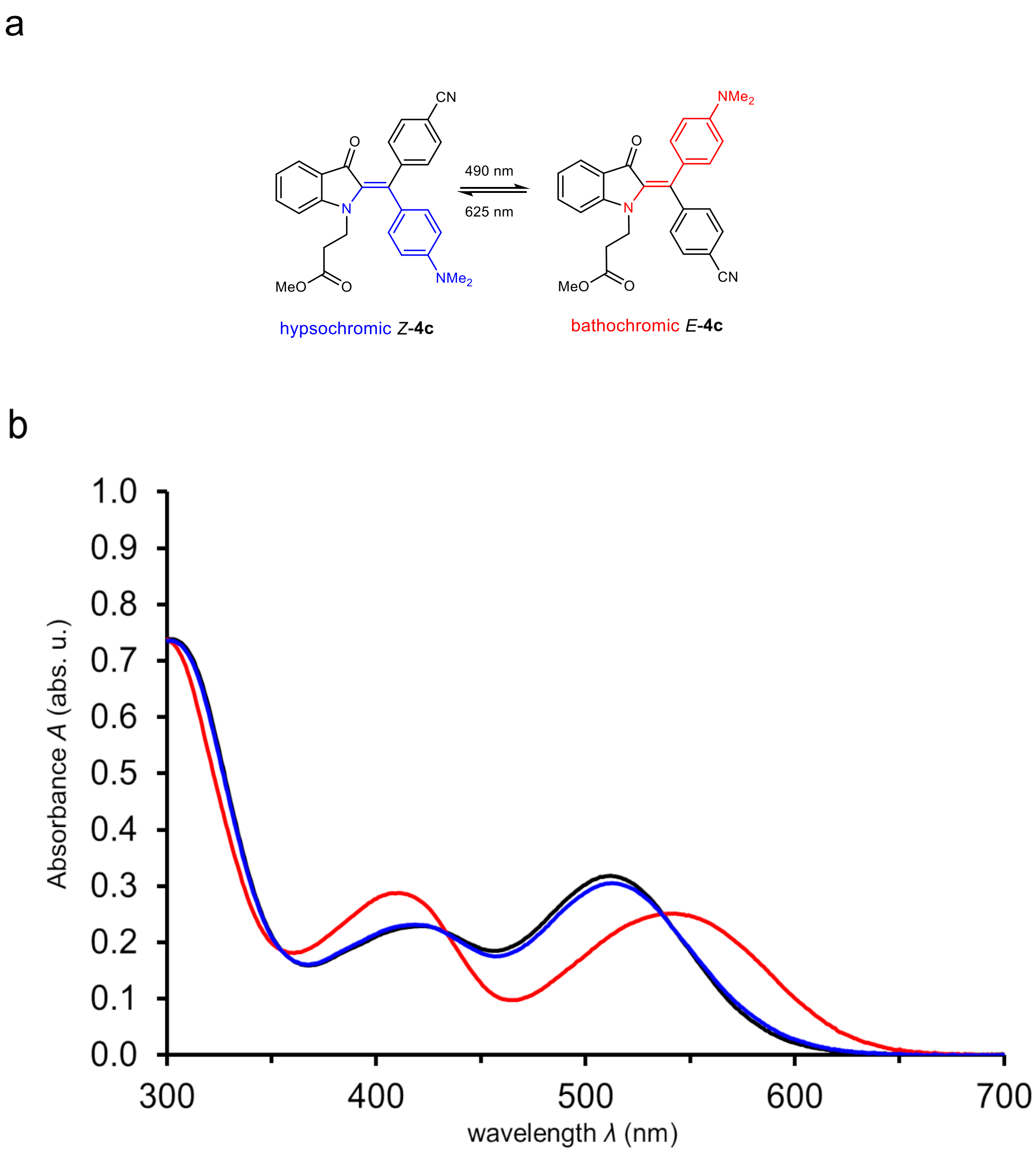

Supplementary Figure 79: a Schematic illustration of the photoisomerization from the hypsochromic to the bathochromic isomer of diaryl-HI **4c** and vice versa. **b** Absorption spectra of diaryl-HI **4c** in tetrahydrofuran solution at 23 °C recorded before and after different irradiation durations using light of different wavelengths. Absorbance of a diaryl-HI **4c** solution containing a mixture of *E* and *Z* isomer before irradiation (black line, A_{max} at 512 nm), absorbance of the bathochromic *E* isomer enriched solution (red line, A_{max} at 543 nm) obtained after irradiation to the pss with 490 nm light, absorbance of the hypsochromic *Z* isomer enriched solution (blue line, A_{max} at 512 nm) obtained after irradiation to the pss with 625 nm light. Source data are provided as Source Data File.

a

b

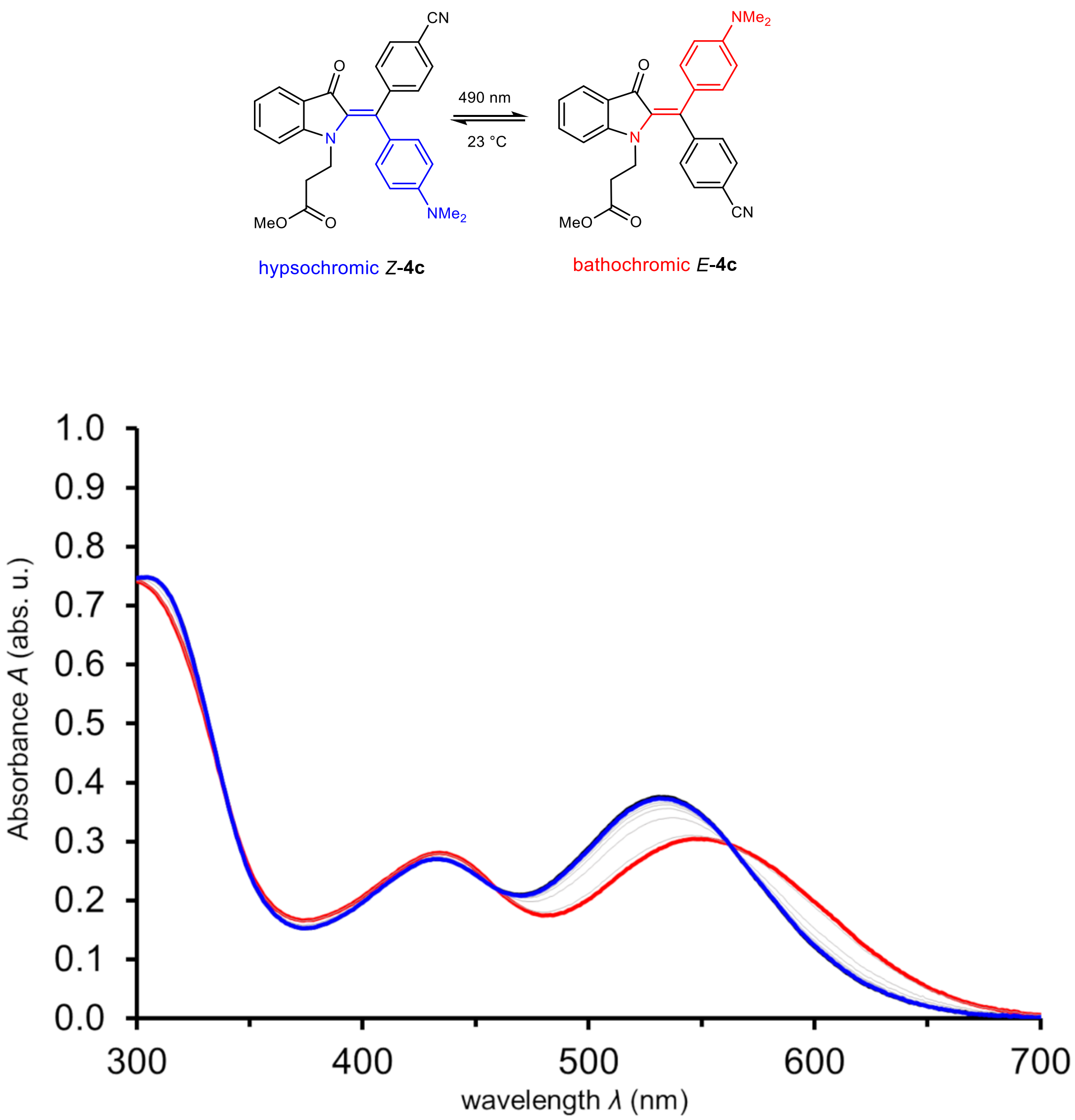

Supplementary Figure 80: a Schematic illustration of the photoisomerization from the hypsochromic to the bathochromic isomer of diaryl-HI **4c** and vice versa thermal isomerization at 23 °C. **b** Absorption spectra of diaryl-HI **4c** in methanol solution at 23 °C recorded before and after different irradiation durations using light of 490 nm. Absorbance of a diaryl-HI **4c** solution containing a mixture of *E* and *Z* isomer before irradiation (black line, Abs_{max} at 533 nm), absorbance of the bathochromic *E* isomer enriched solution (red line, A_{max} at 552 nm) obtained after irradiation to the pss with 490 nm light, absorbance of the hypsochromic *Z* isomer enriched solution (grey lines and blue line with, A_{max} at 531 nm) obtained after different times of thermal isomerization at 23 °C beginning with bathochromic *E* isomer enriched solution. The thermal bistability is decreased in methanol compared to tetrahydrofuran, toluene, or *o*-xylene. Source data are provided as Source Data File.

Supplementary Note 6: Quantum yield determination

The initial slope method requires that quantum yields can only be determined for diaryl-HIs whose hypsochromic species can be enriched to at least 80% in solution. This is because a polynomial fit is used to extrapolate to a composition of 100% hypsochromic and 0% bathochromic diaryl-HI species against time in solution. The initial slope m_0 belonging to the first order derivative of the polynomial fit at 100% hypsochromic diaryl-HI species is a key parameter in the quantum yield determination formula. The stronger the hypsochromic species is enriched, the more accurate the polynomial fit becomes. Enrichment was done prior to measurement by irradiating the respective diaryl-HI solutions with a 625 nm LED (Supplementary Table 4). Sample solutions in UV/Vis cuvettes were then irradiated with different HP-LEDs connected to a glass fiber and photons were counted using a previously published instrumental setup of the E. Riedle group.[1]

The changing concentrations of both species were determined according to (17 and (18) from the known molar absorption coefficients Equation (12) to (14), the known total concentration of the sample ((15)) and the condition expressed in (16), which shows that the measured absorption spectrum of an *E* + *Z* mixture is the sum of the individual absorption spectra of the sole *E* and *Z* isomers.

$$\varepsilon_{\lambda} = \frac{A_{\lambda,[\mathrm{E+Z}]}}{[E+Z]} \tag{12}$$

ε_{λ} = molar absorption coefficient of the *E* + *Z* isomer mixture at certain wavelength λ in L mol^{-1}

$A_{\lambda,[\mathrm{E+Z}]}$ = absorbance of the sample at certain wavelength λ and distinct total concentration of the *E* + *Z* isomer mixture in abs. u.

$[E+Z]$ = total concentration of the *E* + *Z* isomer mixture in the UV/Vis cuvette in mol L^{-1}

$$\varepsilon_{\mathrm{E},\lambda} = \frac{A_{\mathrm{E},\lambda,[\mathrm{E}]}}{[E]} \tag{13}$$

$\varepsilon_{\mathrm{E},\lambda}$ = molar absorption coefficient of the *E* isomer of the diaryl-HI at certain wavelength λ in L mol^{-1}

$A_{\mathrm{E},\lambda,[\mathrm{E}]}$ = absorbance of the *E* isomer at certain wavelength λ and concentration of [*E*] in abs. u.

$[E]$ = concentration of the *E* isomer in the UV/Vis cuvette in mol L^{-1}

$$\varepsilon_{\mathrm{Z},\lambda} = \frac{A_{\mathrm{Z},\lambda,[\mathrm{Z}]}}{[Z]} \tag{14}$$

$\varepsilon_{\mathrm{Z},\lambda}$= molar absorption coefficient of the *Z* isomer of the diaryl-HI at certain wavelength λ in L mol^{-1}

$A_{\mathrm{Z},\lambda,[\mathrm{Z}]}$ = absorbance of the *Z* isomer at certain wavelength λ and concentration of [*Z*] in abs. u.

$[Z]$ = concentration of the *Z* isomer in the UV/Vis cuvette in mol L^{-1}

$$[E+Z] = [E] + [Z] \tag{15}$$

$$A_{\lambda,[\mathrm{E+Z}]} = A_{\mathrm{E},\lambda,[\mathrm{E}]} + A_{\mathrm{Z},\lambda,[\mathrm{Z}]} \tag{16}$$

$$[E] = [E+Z]\frac{\varepsilon_{\lambda} - \varepsilon_{\mathrm{Z},\lambda}}{\varepsilon_{\mathrm{E},\lambda} - \varepsilon_{\mathrm{Z},\lambda}} \tag{17}$$

$$[Z] = [E+Z]\left(1 - \frac{\varepsilon_{\lambda} - \varepsilon_{\mathrm{Z},\lambda}}{\varepsilon_{\mathrm{E},\lambda} - \varepsilon_{\mathrm{Z},\lambda}}\right) \tag{18}$$

The photochemical quantum yields $\phi_{Z/E}$ and $\phi_{E/Z}$ of the *Z* → *E* and *E* → *Z* photoisomerization are described as the ratio of isomerized molecules to absorbed photons according to (19), which is based on the physical quantities of (20) to (23). In the following, subscripted zeros refer to an extrapolated initial state of 100% hypsochromic diaryl-HI species, which is equivalent to the starting point t_0 of the experiment.

$$\Phi = \frac{n}{n_{\mathrm{h\nu}}} = \frac{V_{\mathrm{sample}} \cdot \mathrm{N_A} \cdot \mathrm{h} \cdot \mathrm{c}}{P_0 \cdot f_{\mathrm{corrected}} \cdot \lambda_{\mathrm{ex}}} m_0 \cdot c_{\mathrm{sample}} \tag{19}$$

Φ = photochemical quantum yield of the arising species

n = number of isomerized molecules

$n_{\mathrm{h\nu}}$ = number of absorbed photons

V_{sample} = sample volume within the UV/Vis cuvette in L

N_A = Avogadro constant (6.02214×10^{23} mol^{-1})

h = Planck constant (6.626×10^{-34} J s)

c = speed of light (2.99792×10^{8} m s^{-1})

m_0 = initial slope at t_0 of the arising isomer against time in % s^{-1}

$P_0 \cdot f_{\mathrm{corrected}}$ = corrected weighted initial power read-out at the photometer at t_0 in W

λ_{ex} = excitation wavelength in m

The linearly extrapolated, weighted, and corrected initial power read-out at the photometer at 100% hypsochromic diaryl-HI species $P_0 \times f_{\mathrm{corrected}}$ of (23) was determined as following. At first, the power read-out measured in the presence of the sample P_{sample} at any time t of the experiment was subtracted from the power read-out measured in the presence of the solely solvent filled UV/Vis cuvette P_{solv} according to (20).

$$P = (P_{\mathrm{solv}} - P_{\mathrm{sample}}) \text{ at any time } t \tag{20}$$

P = power read-out at the photometer at any time t in W

P_{solv} = power read-out in the presence of the solvent filled UV/Vis cuvette at any time t in W

P_{sample} = power read-out in the presence of the sample filled UV/Vis cuvette during photoisomerization at any time t in W

Then, a small correction factor f accounting for back reflection at the terminal glass/air interface of the cuvette was obtained for every measured power read-out value P ((20)) of the experiment according to (21).

$$f = \frac{1 + R\frac{P_{\text{sample}}}{P_{\text{solv}}}}{1 - R} \tag{21}$$

f = small correction factor from back reflection at the terminal glass/air interface

R = reflection coefficient of the cuvette at the exit surface glass/air (0.0357)

The power read-out values P of (20) were then multiplied with the small correction factors f of (21) to give the weighted power read-out $P \times f$ at the photometer for every measuring point of the experiment. The $P \times f$ values were plotted against the relative concentration decrease in % of the corresponding hypsochromic diaryl-HI species during photoisomerization. A linear regression was fitted and extrapolated to the initial weighted power read-out value at 100% hypsochromic diaryl-HI species $P_0 \times f_0$ at t_0.

The $P \times f$ values were also corrected according to (22) by the time dependent correction factor k for the intensity read-out drift of the photometer.

$$P \cdot f_{\text{corrected}} = P \cdot f \cdot k \text{ at any time } t \tag{22}$$

$P \cdot f_{\text{corrected}}$= weighted corrected power read-out at 100 % hypsochromic diaryl-HI species in W

k = time dependent correction factor for the intensity read-out drift of the photometer

To this end, the power read-out of the detector at the start ($P_{\text{air,start}}$) and at the end ($P_{\text{air,end}}$) of the experiment was recorded together with the overall experiment time. These numbers define the slope of a line that expresses the intensity read-out drift of the photometer over time. Additionally, the absolute time of every obtained P value was recorded beforehand. With the absolute time of the two measurement points $P_{\text{air,start}}$ and $P_{\text{air,end}}$ and the absolute time of each individual photoisomerization measurement point in hand, the intensity read-out drift of the photometer can be corrected for each $P \times f$ value individually as described above in (22). Again, the $P \times f_{\text{corrected}}$ values were plotted against the relative concentration decrease in % of the corresponding hypsochromic diaryl-HI species during photoisomerization. A linear

regression was fitted and extrapolated to the initial weighted power read-out value at 100% hypsochromic diaryl-HI species $P_0 \times f_{\text{corrected}}$ at t_0. The extrapolated $P_0 \times f_{\text{corrected}}$ value of the corresponding linear regression at 100% hypsochromic diaryl-HI as described in (23) was used for (19).

$$P_0 \cdot f_{\text{corrected}} = P_0 \cdot f_0 \cdot k_0 \text{ at } t_0 \tag{23}$$

The initial slope m_0 of (19) was obtained from plotting the % of arising bathochromic diaryl-HI against irradiation time. The first derivative of the corresponding polynomial fit at 0% bathochromic isomer then gives the slope m_0.

The quantum yields for the hypsochromic to bathochromic photoisomerization could be measured for diaryl-HIs **2b**, **2c**, **4b** and **4c**. The quantum yields for the bathochromic to hypsochromic photoisomerization could not be measured due to quantum yields <1% in general for this direction, which severely limited accurate photon counting.

Supplementary Table 5: Experimentally determined quantum yields for the photoisomerization of diaryl-HI isomers in toluene solution at 23 °C and corresponding experimentally measured parameters.

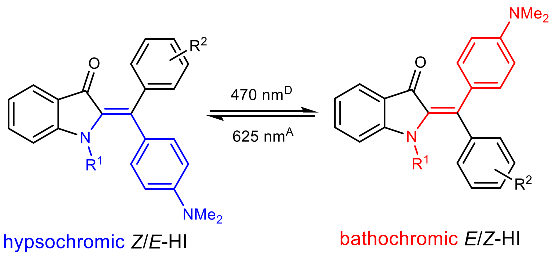

HI	$\phi_{E/Z}$ (%) (at nm)	$\phi_{Z/E}$ (%) (at nm)	c_{sample} (mol L^{-1})	V_{sample} (mL)	$P_0 \times f_{corrected}$ at 100% hypsochromic *E*/*Z*-HI	m_0 (%*E* s^{-1}/ %*Z* s^{-1})
**1a**	-[C]	<1 (625)[A]	-	-	-	-
**1b**	-[C]	<1 (625)[A]	-	-	-	-
**1c**	-[C]	<1 (625)[A]	-	-	-	-
**2a**	-[C]	<1 (625)[A]	-	-	-	-
**2b**	5 (470)[D]	<1 (625)[A]	4.87×10^{-5}	2.55	0.526	-[B]/0.0777
**2c**	4 (470)[D]	<1 (625)[A]	8.55×10^{-5}	2.62	0.636	-[B]/0.0442
**3a**	-[C]	<1 (625)[A]	-	-	-	-
**3b**	-[C]	<1 (625)[A]	-	-	-	-
**3c**	-[C]	<1 (625)[A]	-	-	-	-
**4a**	<1 (625)[A]	-[C]	-	-	-	-
**4b**	<1 (625)[A]	5 (470)[D]	7.81×10^{-5}	2.55	0.580	0.0499/-[B]
**4c**	<1 (625)[A]	6 (470)[D]	1.87×10^{-4}	2.68	0.791	0.0367/-[B]

A: The quantum yields for the bathochromic to hypsochromic direction of photoisomerization could not be measured as the actual values are below 1%. **B:** Initial slope m_0 for the photoisomerization from the bathochromic to the hypsochromic isomer could not be measured due to quantum yields below 1%. **C:** Initial slope method could not be used due to limited enrichment of hypsochromic isomer by irradiation with light. **D:** Quantum yield for the hypsochromic to bathochromic direction of photoisomerization.

6.1 Quantum yield for the *E* to *Z* photoisomerization of diaryl-HI 2b in toluene

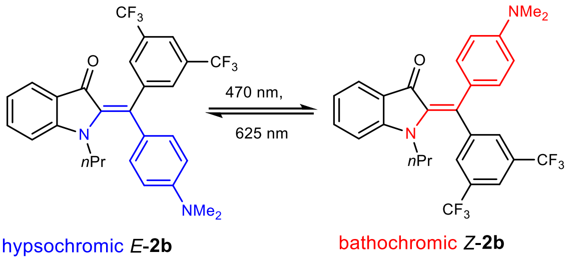

Supplementary Figure 81: Schematic illustration for the photoisomerization from the hypsochromic to the bathochromic isomer of diaryl-HI **2b** in toluene solution at 23 °C. Source data are provided as Source Data File.

The quantum yield measurement for the photoisomerization of the hypsochromic *E* isomer to the bathochromic *Z* isomer of diaryl-HI **2b** in toluene solution using 470 nm light started with a mixture of 82% *E*-**2b** and 18% *Z*-**2b**. Because the reaction did not start at 0% *Z* isomer (Supplementary Figure 82) the photoconversion kinetics were fitted with a polynomial of sixth order to establish the initial slope according to (24):

$$y = -4.4840 \cdot 10^{-20}x^6 + 7.3336 \cdot 10^{-16}x^5 - 4.6955 \cdot 10^{-12}x^4 + 1.5518 \cdot 10^{-8}x^3 - 3.2212 \cdot 10^{-5}x^2 + 5.4664 \cdot 10^{-2}x + 18.958 \quad (24)$$

The polynomial of (24) at y = *Z* isomer [%] = 0 gives x = -289.7853. The first derivation ((25)) of (24) at x = -289.7853 then gives the initial slope $m_0 = 7.7725 \times 10^{-2}$ %*Z* s^{-1} corresponding to 0% *Z* isomer as depicted in Supplementary Figure 82.

$$y' = -2.6904 \cdot 10^{-19}x^5 + 3.6668 \cdot 10^{-15}x^4 - 1.8782 \cdot 10^{-11}x^3 + 4.6553 \cdot 10^{-8}x^2 - 6.4423 \cdot 10^{-5}x + 0.05466 \quad (25)$$

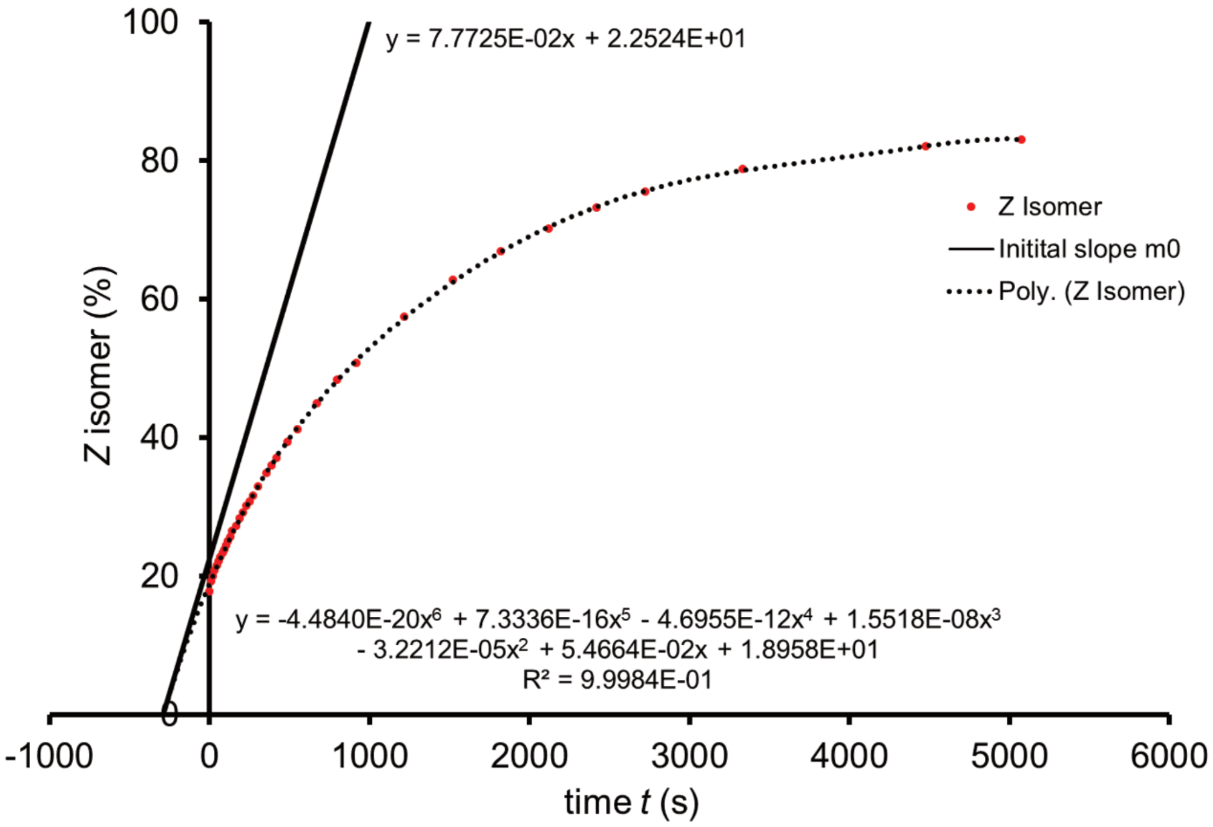

Supplementary Figure 82: *E* to *Z* photoisomerization of diaryl-HI **2b** (red dots) in toluene at 23 °C using a 470 nm HP-LED for irradiation. Starting point of the measurement was an isomeric mixture containing 18% *Z*-**2b**. The photokinetics were fitted with a sixth order polynomial (black dotted line) to establish the initial slope at 0% *Z*-**2b**. The polynomial formula is shown at the bottom. The first derivative of the polynomial formula at *Z* isomer (%) = 0 gives the initial slope $m_0 = 0.0777$ %*Z* s^{-1} (formula at the top of the figure). Source data are provided as Source Data File.

The corrected weighted initial power factor $P_0 \times f_{corrected}$ at the powermeter at 0% *Z* (=100% *E*) isomer content was calculated according to the linear regression of (26) shown in Supplementary Figure 83. The value $P_0 \times f_{corrected} = 0.526$ mW was used for the quantum yield calculation of (19), resulting in a quantum yield of $\phi_{E/Z} = 5\%$.

$$y = 4.7890 \cdot 10^{-4} x + 4.7850 \cdot 10^{-1} \quad (26)$$

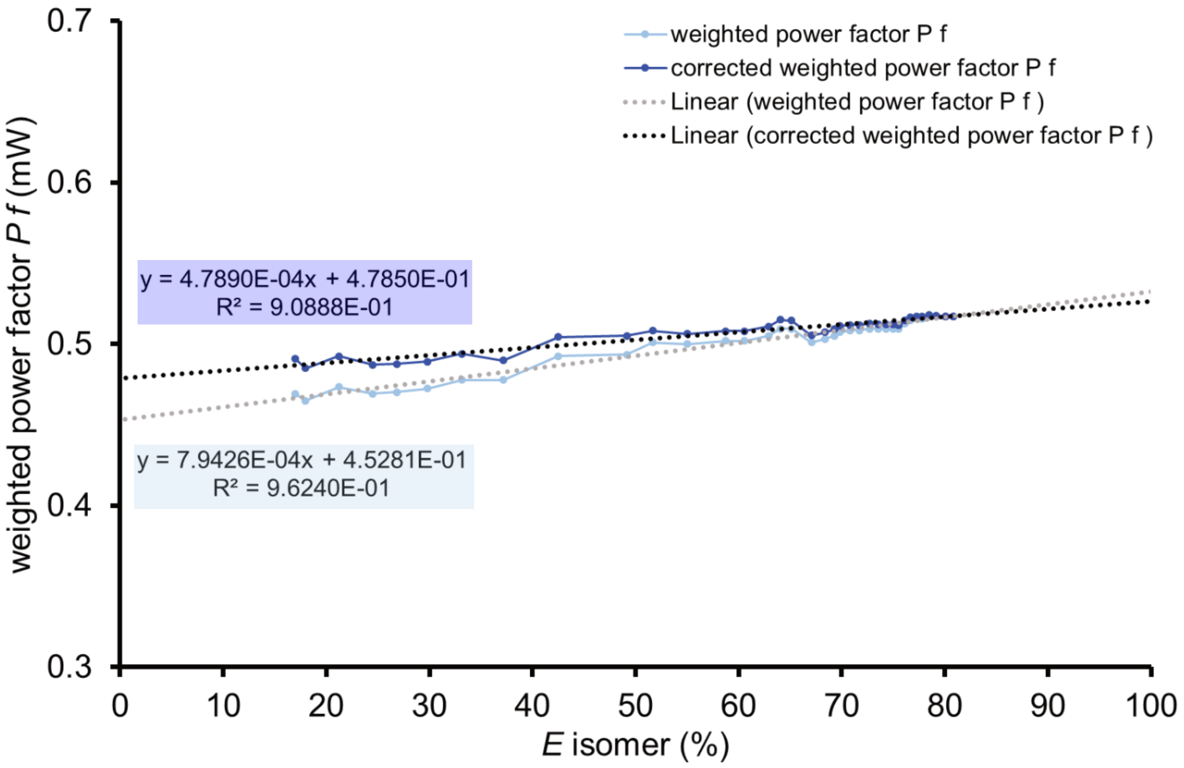

Supplementary Figure 83: Weighted power factor $P \times f$ (light blue progression) plotted as a function of *E* isomer percentage during quantum yield measurement of diaryl-HI **2b** *E* to *Z* photoisomerization and corresponding linear regression (grey dotted line with corresponding formula displayed below). Additionally the corrected weighted power factor $P \times f_{\text{corrected}}$ (dark blue progression, Equation (26)) is plotted together with the corresponding linear regression (black dotted line, formula at the top of the figure). The extrapolated value at 100% *E* isomer of the linear regression of the corrected weighted power factor $P_0 \times f_{\text{corrected}}$ was taken to calculate the quantum yield $\phi_{E/Z}$ (%) in Supplementary Table **5** with the formula of (19). Source data are provided as Source Data File.

6.2 Quantum yield for the E to Z photoisomerization of diaryl-HI 2c in toluene

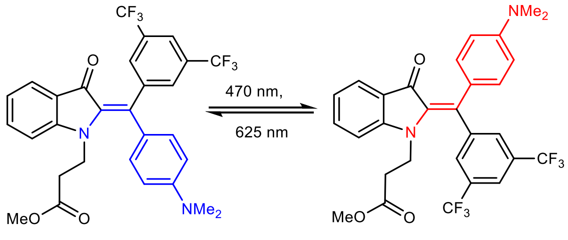

Supplementary Figure 84: Schematic illustration for the photoisomerization from the hypsochromic to the bathochromic isomer of diaryl-HI **2c** in toluene solution at 23 °C. Source data are provided as Source Data File.

The quantum yield measurement for the photoisomerization of the hypsochromic *E* isomer to the bathochromic *Z* isomer of diaryl-HI **2c** in toluene solution using 470 nm light started with a mixture of 87% *E*-**2c** and 13% *Z*-**2c**. Because the reaction did not start at 0% *Z* isomer (also see Supplementary Figure 85) the photoconversion kinetics were fitted with a polynomial of sixth order to establish the initial slope according to (27):

$$y = -1.2340 \cdot 10^{-22}x^6 + 6.6748 \cdot 10^{-18}x^5 - 1.4305 \cdot 10^{-13}x^4 + 1.6017 \cdot 10^{-9}x^3 - 1.0235 \cdot 10^{-5}x^2 + 3.7076 \cdot 10^{-2}x + 13.061 \quad (27)$$

The polynomial of (27) at y = *Z* isomer [%] = 0 gives x = -322.1496. The first derivation ((28)) of (27) at x = -322.1496 then gives the initial slope m_0 = 4.4189 · 10^{-2} %*Z* s^{-1} corresponding to 0% *Z* isomer as depicted in Supplementary Figure 85.

$$y' = -7.4037 \cdot 10^{-22}x^5 + 3.3374 \cdot 10^{-17}x^4 - 5.7221 \cdot 10^{-13}x^3 + 4.8050 \cdot 10^{-9}x^2 - 2.0470 \cdot 10^{-5}x + 0.0371 \quad (28)$$

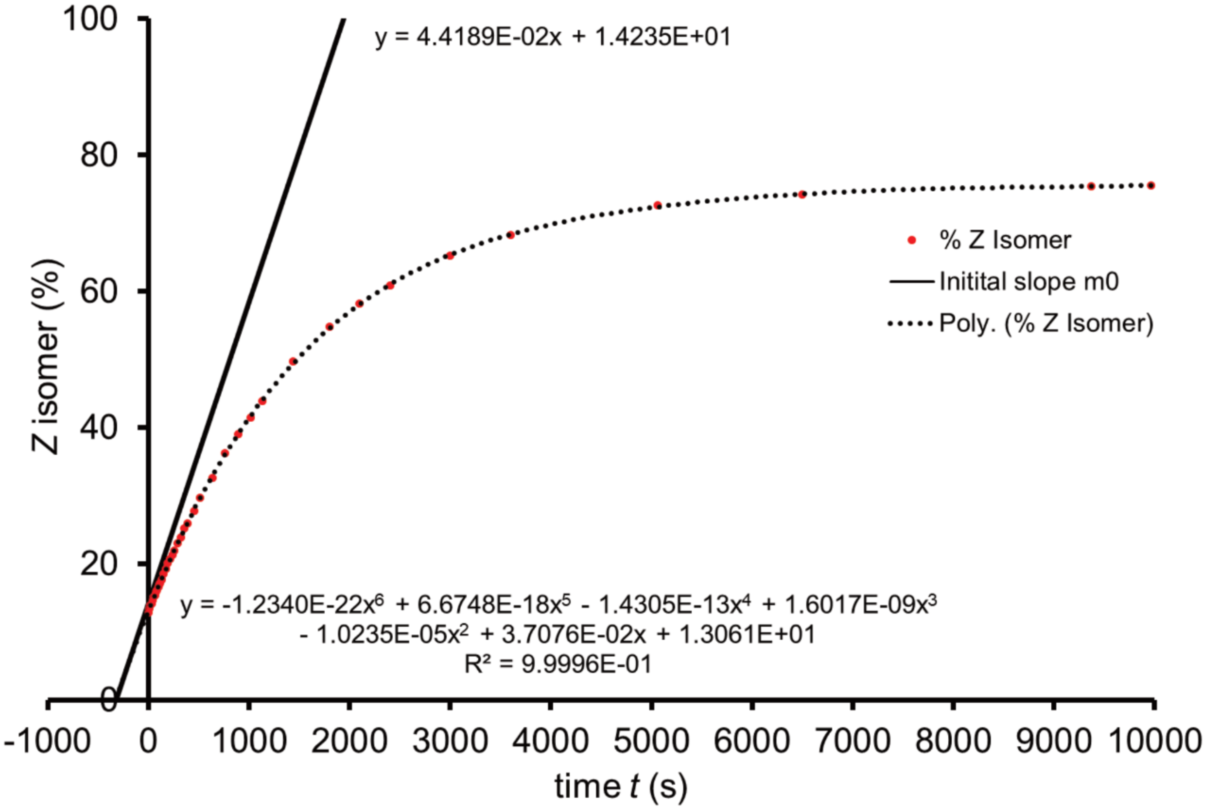

Supplementary Figure 85: *E* to *Z* photoisomerization of diaryl-HI **2c** (red dots) in toluene at 23 °C using a 470 nm HP-LED for irradiation. Starting point of the measurement was an isomeric mixture containing 13% *Z*-**2c**. The photokinetics was fitted with a sixth order polynomial (black dotted line) to establish the initial slope at 0% *Z*-**2c**. The polynomial formula is shown at the bottom. The first derivative of the polynomial formula at *Z* isomer (%) = 0 gives the initial slope $m_0 = 0.0442\%Z\ s^{-1}$ (formula at the top of the figure). Source data are provided as Source Data File.

The corrected weighted initial power factor $P_0 \times f_{\text{corrected}}$ at the powermeter at 0% *Z* (=100% *E*) isomer content was calculated according to the linear regression of (29) shown in Supplementary Figure 86). The value $P_0 \times f_{\text{corrected}} = 0.636$ mW was used for the quantum yield calculation of (19), resulting in a quantum yield of $\phi_{E/Z} = 4\%$.

$$y = 2.3035 \cdot 10^{-3}x + 0.4061 \tag{29}$$

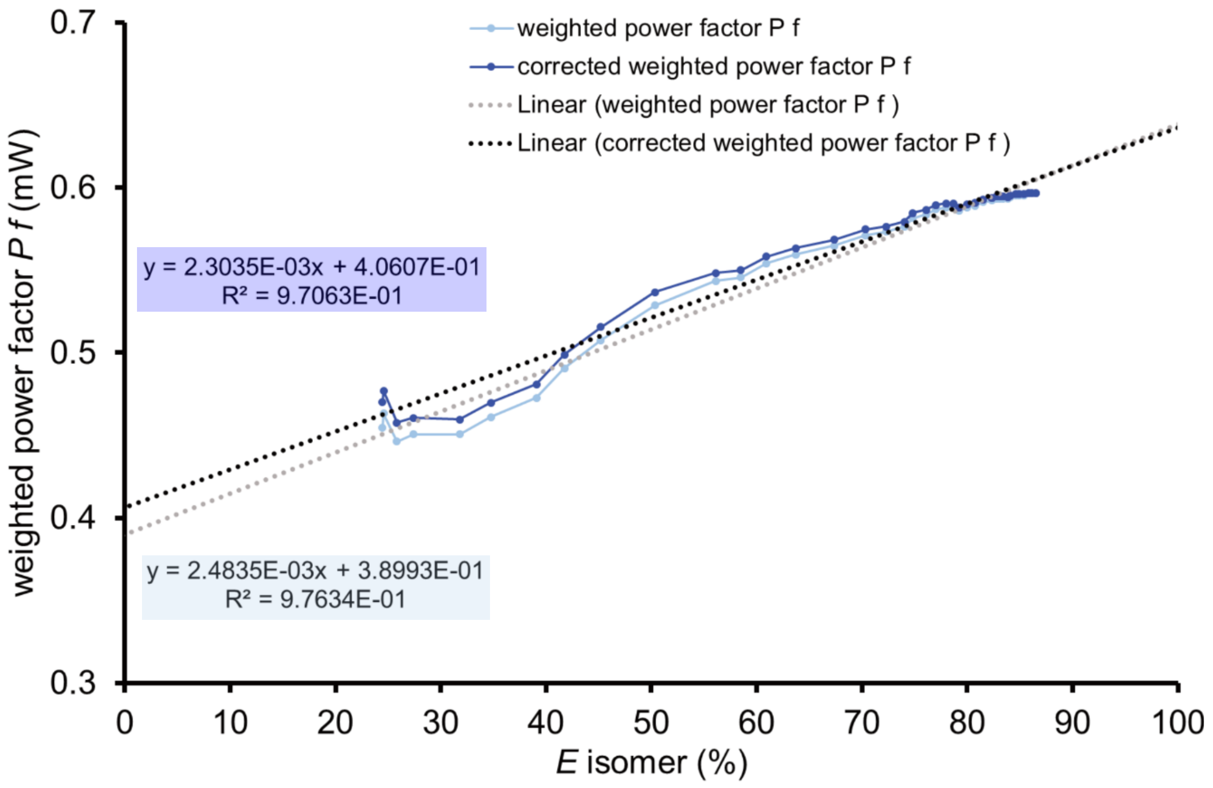

Supplementary Figure 86: Weighted power factor $P \times f$ (light blue progression) plotted as a function of *E* isomer percentage during quantum yield measurement of diaryl-HI **2c** *E* to *Z* photoisomerization and corresponding linear regression (grey dotted line with corresponding formula displayed below). Additionally the corrected weighted power factor $P \times f_{\text{corrected}}$ (dark blue progression, Equation (29)) is plotted together with the corresponding linear regression (black dotted line, formula at the top of the figure). The extrapolated value at 100% *E* isomer of the linear regression of the corrected weighted power factor $P_0 \times f_{\text{corrected}}$ was taken to calculate the quantum yield $\phi_{E/Z}$ (%) in Supplementary Table 5 with the formula of Equation (19). Source data are provided as Source Data File.

6.3 Quantum yield for the *Z* to *E* photoisomerization of diaryl-HI 4b in toluene

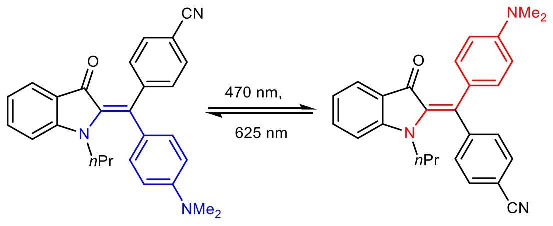

Supplementary Figure 87: Schematic illustration for the photoisomerization from the hypsochromic to the *bathochromic* isomer of diaryl-HI **4b** in toluene solution at 23 °C. Source data are provided as Source Data File.

The quantum yield measurement for the photoisomerization of the hypsochromic *Z* isomer to the *bathochromic E* isomer of diaryl-HI **4b** in toluene solution using 470 nm light started with a mixture of 21% *E*-**4b** and 79% *Z*-**4b**. Because the reaction did not start at 0% *E* isomer (Supplementary Figure 88)) the photoconversion kinetics were fitted with a polynomial of sixth order to establish the initial slope according to (30):

$$y = -3.9280 \cdot 10^{-21}x^6 + 7.3861 \cdot 10^{-17}x^5 - 5.8155 \cdot 10^{-13}x^4 + 2.8698 \cdot 10^{-9}x^3 - 1.1492 \cdot 10^{-5}x^2 + 3.5816 \cdot 10^{-2}x + 21.1396 \tag{30}$$

The polynomial of (30) at y = *Z* isomer (%) = 0 gives x = -504.3775. The first derivation ((31)) of (30) at x = -504.3775 gives the initial slope m_0 = 4.9922 × 10^{-2} %*E* s^{-1} corresponding to 0% *E* isomer as depicted in Supplementary Figure 88.

$$y' = -2.3568 \cdot 10^{-20}x^5 + 3.6931 \cdot 10^{-16}x^4 - 2.3262 \cdot 10^{-12}x^3 + 8.6095 \cdot 10^{-9}x^2 - 2.2984 \cdot 10^{-5}x + 3.5816 \cdot 10^{-2} \tag{31}$$

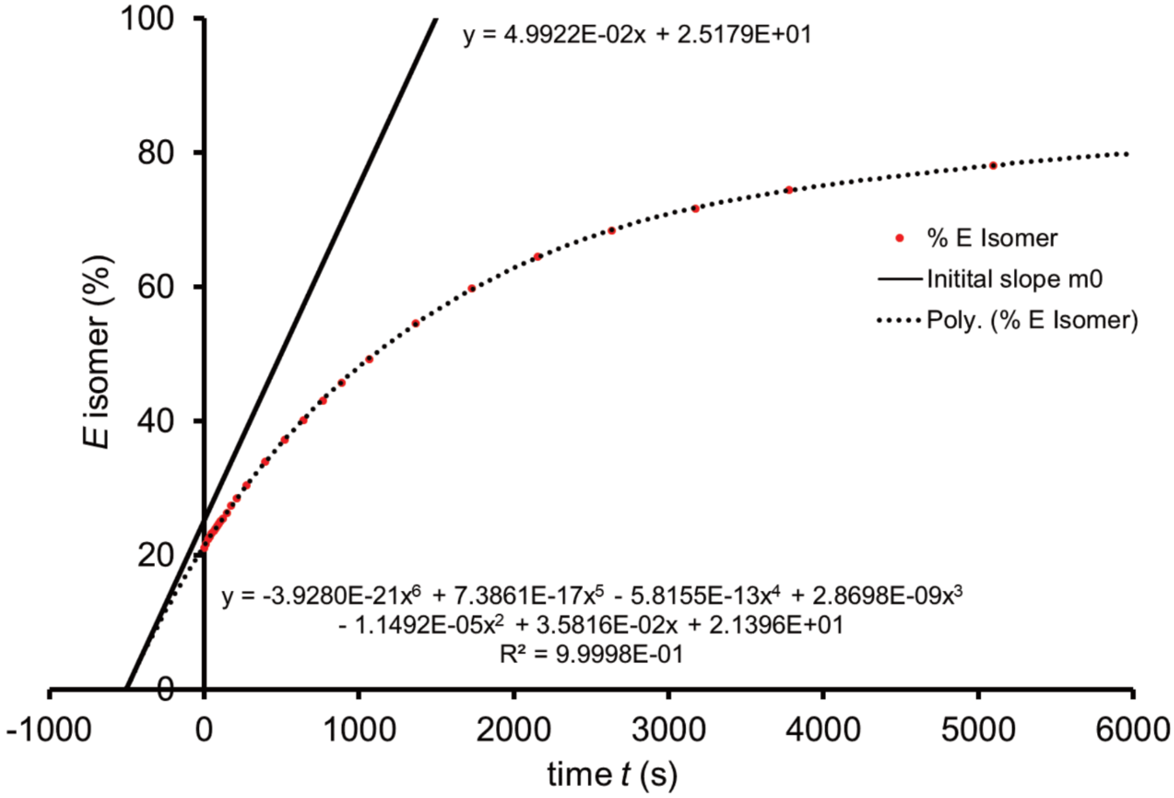

Supplementary Figure 88: *Z* to *E* photoisomerization of diaryl-HI **4b** (red dots) in toluene at 23 °C using a 470 nm HP-LED for irradiation. Starting point of the measurement was an isomeric mixture containing 21% *E*-**4b**. The photokinetics was fitted with a sixth order polynomial (black dotted line) to establish the initial slope at 0% *E*-**4b**. The polynomial formula is shown at the bottom. The first derivative of the polynomial formula at *E* isomer (%) = 0 gives the initial slope m_0 = 0.0499 %*E* s^{-1} (formula at the top of the figure). Source data are provided as Source Data File.

The corrected weighted initial power factor $P_0 \times f_{\text{corrected}}$ at the powermeter at 0% *E* (=100% *Z*) isomer content was calculated according to the linear regression of (32) shown in Supplementary Figure 89. The value $P_0 \times f_{\text{corrected}}$ = 0.580 mW was used for the quantum yield calculation of (19), resulting in a quantum yield of $\phi_{Z/E}$ = 5%.

$$y = -8.4331 \cdot 10^{-5}x + 5.8870 \cdot 10^{-1} \tag{32}$$

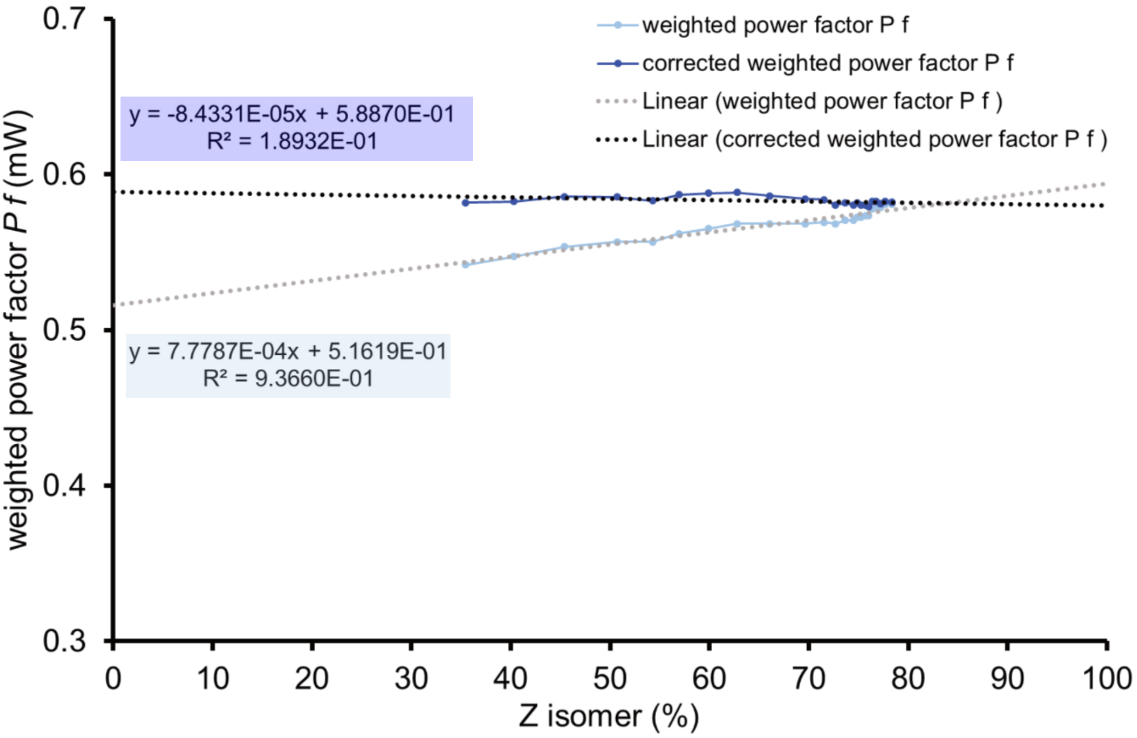

Supplementary Figure 89: Weighted power factor $P \times f$ (light blue progression) plotted as a function of E isomer percentage during quantum yield measurement of diaryl-HI **4b** E to Z photoisomerization and corresponding linear regression (grey dotted line with corresponding formula displayed below). Additionally the corrected weighted power factor $P \cdot f_{\mathrm{corrected}}$ (dark blue progression, (39)) is plotted together with the corresponding linear regression (black dotted line, formula at the top of the figure). The extrapolated value at 100% Z isomer of the linear regression of the corrected weighted power factor $P_0 \times f_{\mathrm{corrected}}$ was taken to calculate the quantum yield $\phi_{Z/E}$ (%) in Supplementary Table 5 with the formula of Equation (19). Source data are provided as Source Data File.

6.4 Quantum yield for the *Z* to *E* photoisomerization of diaryl-HI 4c in toluene

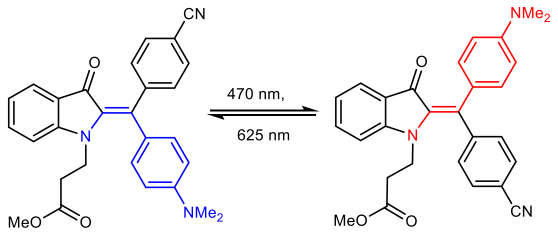

Supplementary Figure 90: Schematic illustration for the photoisomerization from the hypsochromic to the bathochromic isomer of diaryl-HI **4c** in toluene solution at 23 °C. Source data are provided as Source Data File.

The quantum yield measurement for the photoisomerization of the hypsochromic *Z* isomer to the bathochromic *E* isomer of diaryl-HI **4c** in toluene solution using 470 nm light started with a mixture of 20% *E*-**4c** and 80% *Z*-**4c**. Because the reaction did not start at 0% *E* isomer (also see (45)) the photoconversion kinetics were fitted with a polynomial of fifth order to establish the initial slope according to (33):

$$y = 1.9715 \cdot 10^{-18}x^5 - 6.2898 \cdot 10^{-14}x^4 8.4721 \cdot 10^{-3}x^3 - 6.3455 \cdot 10^{-6}x^2 + 2.7703 \cdot 10^{-2}x + 20.127 \quad (33)$$

The polynomial of at y = *Z* isomer (%) = 0 gives x = -628.1850. The first derivation ((34)) of (33) at x = -628.1850 gives the initial slope m_0 = 3.6742 × 10^{-2} %*E* s^{-1} corresponding to 0% *E* isomer as depicted in Supplementary Figure 91.

$$y' = 9.8575 \cdot 10^{-18}x^4 - 2.5159 \cdot 10^{-13}x^3 + 2.5416 \cdot 10^{-9}x^2 - 1.2691 \cdot 10^{-5}x + 2.7703 \cdot 10^{-2} \quad (34)$$

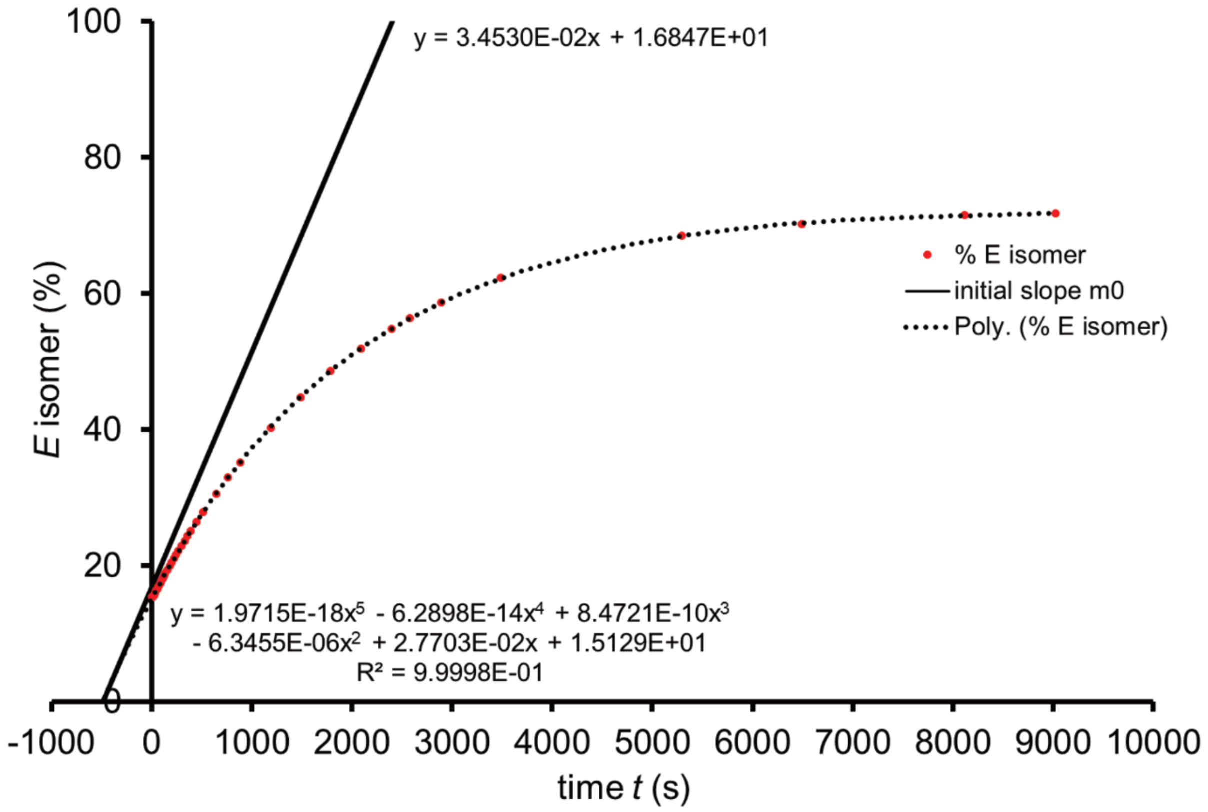

Supplementary Figure 91: *Z* to *E* photoisomerization of diaryl-HI **4c** (red dots) in toluene at 23 °C using a 470 nm HP-LED for irradiation. Starting point of the measurement was an isomeric mixture containing 20% *E*-**4c**. The photokinetics was fitted with a sixth order polynomial (black dotted line) to establish the initial slope at 0% *E*-**4c**. The polynomial formula is shown at the bottom. The first derivative of the polynomial formula at *E* isomer (%) = 0 gives the initial slope m_0 = 0.0367 %*E* s^{-1} (formula at the top of the figure). Source data are provided as Source Data File.

The corrected weighted initial power factor $P_0 \times f_{\text{corrected}}$ at the powermeter at 0% *E* (=100% *Z*) isomer content was calculated according to the linear regression of (35) in Supplementary Figure 92. The value $P_0 \times f_{\text{corrected}}$ = 0.7905 mW was used for the quantum yield calculation of (19), resulting in a quantum yield of $\phi_{Z/E}$ = 6%.

$$y = 1.6764 \cdot 10^{-3}x + 6.2290 \cdot 10^{-1} \quad (35)$$

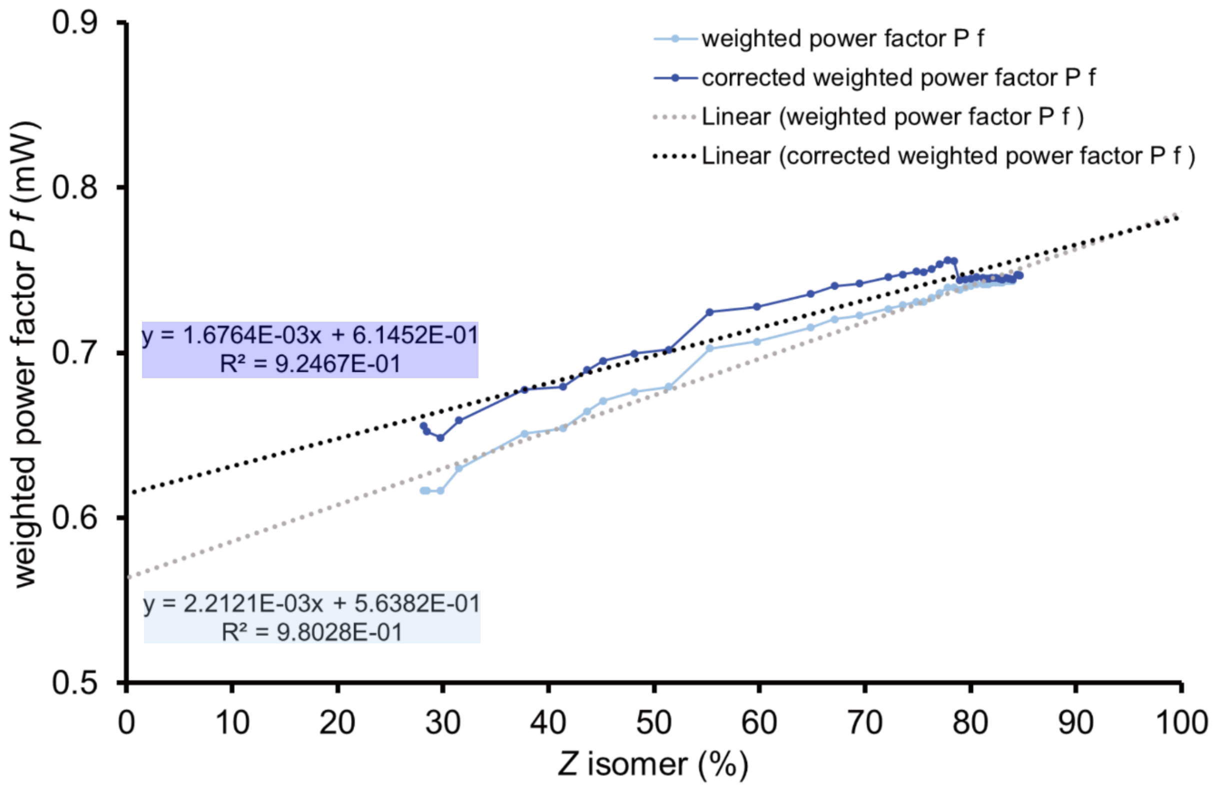

Supplementary Figure 92: Weighted power factor $P \times f$ (light blue progression) plotted as a function of *E* isomer percentage during quantum yield measurement of diaryl-HI **4c** *E* to *Z* photoisomerization and corresponding linear regression (grey dotted line with corresponding formula displayed below). Additionally the corrected weighted power factor $P \times f_{corrected}$ (dark blue progression, (39)) is plotted together with the corresponding linear regression (black dotted line, formula at the top of the figure). The extrapolated value at 100% *Z* isomer of the linear regression of the corrected weighted power factor $P_0 \times f_{corrected}$ was taken to calculate the quantum yield $\phi_{Z/E}$ (%) in Supplementary Table 5 with the formula of Equation (19). Source data are provided as Source Data File.

Supplementary Note 7: Thermal stabilities of isomeric states

NMR tubes were charged with the respective diaryl-HI solutions at concentrations in the mid mM range. The diaryl-HI solutions within the NMR tubes were then irradiated with suitable LEDs of emission maxima ranging from 450 nm to 590 nm to enrich a particular isomer. The resulting solutions were then kept at temperatures between 0 °C to 135 °C in the dark and changes in the isomer composition were determined by ^{1}H NMR spectroscopy through integration of indicative signals. Consecutive ^{1}H NMR measurements were measured in suitable time intervals to obtain enough measurement points. At the beginning of each thermal isomerization kinetic measurement, the heating intervals between measurement points were chosen to be short and then were prolonged over measurement progress to keep the changes of isomer compositions well trackable over time. The measurements were completed after the isomer composition reached its equilibrium, which was evidenced by constant isomer composition over three heating intervals.

HIs possessing a Gibbs energy of activation for thermal isomerization larger than 24 kcal mol^{-1} were irradiated with external LED light sources at 22 °C. The heating processes were carried out in oil bathes at temperatures between 60 °C to 135 °C. After the heating interval, the samples were quickly cooled to 0 °C in an ice bath before the ^{1}H NMR spectra were recorded. This was done to shock-freeze the isomer composition obtained at high temperature for accurate measurements.

HIs possessing a Gibbs energy of activation for thermal isomerization smaller than 24 kcal mol^{-1} were irradiated in situ at 0 °C or 25 °C with high power LEDs connected to a glass fiber within the NMR spectrometer. After irradiation, the thermal isomerizations at 0 °C or 25 °C in the dark were also recorded by ^{1}H NMR measurements.

For simplification, the formular below describe the thermal isomerization from *E* to *Z* isomers. The vice versa thermal *Z* to *E* isomerization is described by the corresponding similar equations. The two unimolecular elemental reactions of the thermal isomerization process are described by (36) and (37):

$$E \overset{k_{\mathrm{E}}}{\rightarrow} Z \tag{36}$$

$$Z \overset{k_{\mathrm{Z}}}{\rightarrow} E \tag{37}$$

E = decaying species during thermal isomerization

Z = arising species during thermal isomerization

k_{E} = rate constant of thermal isomerization from species $E \rightarrow Z$ at experimental temperature in s^{-1}

k_{Z} = rate constant of thermal isomerization from species $Z \rightarrow E$ at experimental temperature in s^{-1}

The combination of the forward and backward thermal isomerization reactions described in (36) and (37) leads to the thermal equilibrium of the isomers E and Z as described by (38):

$$E \underset{k_{\mathrm{Z}}}{\overset{k_{\mathrm{E}}}{\rightleftarrows}} Z \tag{38}$$

The corresponding rate law to (38) can be described as following:

$$\frac{\mathrm{d}[E]}{\mathrm{d}t} = k_{\mathrm{Z}}[Z] - k_{\mathrm{E}}[E] \tag{39}$$

$[E]$ = concentration of decaying species during thermal isomerization in mol L^{-1}

$[Z]$ = concentration of arising species during thermal isomerization in mol L^{-1}

The concentration of the rising isomer Z at any time t of the experiment can be expressed through (40) with the initial concentrations $[E_0]$ und $[Z_0]$ of the isomers at the start time t_0 and $[E]$, the concentration of isomer E at any time t of the experiment.

$$[Z] = [E] + [Z_0] - [E] \tag{40}$$

$[E_0]$ = initial concentration of decaying species during thermal isomerization in mol L^{-1}

$[Z_0]$ = initial concentration of arising species during thermal isomerization in mol L^{-1}

The combination of (39) and (40) leads to (41):

$$\frac{\mathrm{d}[E]}{\mathrm{dt}} = k_Z[E_0 + Z_0] - [E](k_E + k_Z) \tag{41}$$

As the thermal isomerization results in an equilibrium with no net-changes of isomer composition ((42)), (39) and (40) can be written by taking into account the law of mass action as given in (43) and (44).

$$\frac{\mathrm{d}[E]}{\mathrm{d}t} = \frac{\mathrm{d}[Z]}{\mathrm{d}t} = 0 \tag{42}$$

$$\frac{k_E}{k_Z} = \frac{[Z_{eq}]}{[E_{eq}]} = K \tag{43}$$

$$[Z_{eq}] = [E_0] + [Z_0] - [E^{eq}] \tag{44}$$

$[E_{eq}]$ = concentration of decaying species at thermal equilibrium in mol L^{-1}

$[Z_{eq}]$ = concentration of arising species at thermal equilibrium in mol L^{-1}

K = thermodynamic equilibration constant

After insertion of (44) into (43) and transforming it to express $k_Z[E_0+Z_0]$, (45) was obtained.

$$k_Z[E_0 + Z_0] = [E_{eq}](k_E + k_Z) \tag{45}$$

Insertion of (45) into (41) leads to the previously mentioned rate law of first order, as shown in (46), which is transformed into (47) by taking into account, that $\mathrm{d}[E_{eq}]/\mathrm{d}t$ has

to be zero, which means that d[E] can be described as d[$E - E_{eq}$].

$$\frac{\mathrm{d}[E]}{\mathrm{dt}} = (k_\mathrm{E} + k_\mathrm{Z})\,[E_\mathrm{eq} - E] \tag{46}$$

$$\frac{\mathrm{d}[E]}{[E - E_\mathrm{eq}]} = \frac{\mathrm{d}[E - E_\mathrm{eq}]}{[E - E_\mathrm{eq}]} = -(k_\mathrm{E} + k_\mathrm{Z})\mathrm{d}t \tag{47}$$

Integration of (47) within the range of $t_0 \rightarrow t$ and applying the constraint that $t_0 = 0$ leads to (49), which takes into account, that the global rate constant k towards a thermodynamic equilibrium K is the sum of the elemental rate constants k_E and k_Z, gives (48).

$$k = k_\mathrm{E} + k_\mathrm{Z} \tag{48}$$

$$\ln\left(\frac{[E_0 - E_\mathrm{eq}]}{[E_\mathrm{t} - E_\mathrm{eq}]}\right) = (k_\mathrm{E} + k_\mathrm{Z})t = kt \tag{49}$$

k = global rate constant of thermal isomerization from $E \rightarrow Z$ and $Z \rightarrow E$ towards a thermodynamic equilibrium at experimental temperature T in s^{-1}

The derivation towards (49) also shows, that the thermal E/Z or Z/E isomerization can be a unimolecular first order reaction, that proceeds towards an equilibrium with both isomers present, if the plot of the decaying isomer E proportion against time t according to the logarithmic left part of (49) shows a linear correlation. The resulting slope of this plot determines the experimentally obtained global rate constant k at the experimental temperature T. The insertion of the law of mass action of (43) into (48) is demonstrated in (50), which can be transformed into (51) and shows how to calculate the rate constant k_E out of the previously mentioned plot of constant k ((49)).

$$k = k_{\mathrm{E}} + k_{\mathrm{E}}\frac{[E_{\mathrm{eq}}]}{[Z_{\mathrm{eq}}]} \tag{50}$$

$$k_{\mathrm{E}} = \frac{k}{1 + \frac{[E_{\mathrm{eq}}]}{[Z_{\mathrm{eq}}]}} \tag{51}$$

The equilibration half-life time $t_{1/2}^{\mathrm{eq}}$ of a thermal isomerization towards an equilibrium can be derived from (49) with the constraint, that

$$E = [E_0] - \frac{[E_0 - E_{\mathrm{eq}}]}{2} = \frac{[E_0 + E_{\mathrm{eq}}]}{2} \tag{52}$$

and is described by (53) via insertion of (52) into (49) and transforming it to express t (= $t_{1/2}^{\mathrm{eq}}$).

$$t_{1/2}^{\mathrm{eq}} = \frac{\ln 2}{k} \tag{53}$$

> $t_{1/2}^{\mathrm{eq}}$ = equilibration half-life time of thermal isomerization from $E \rightarrow Z$ at experiment temperature in s

The calculation for the thermal equilibration half-life times at 25 °C ($t_{1/2}^{\mathrm{eq}\,25\,°\mathrm{C}}$, (55)) has been done by linear extrapolation of the global rate constant k to 25 °C using the Eyring equation in the form of (56). This has been done for better comparison of the different photoswitches. Therefore, the thermal equilibration Gibbs energy of activation $\Delta G^{\ddagger}_{\mathrm{E}\leftrightarrow\mathrm{Z\ equil.}}$ ((54)) for the thermal isomerization from species $E \rightarrow Z$ uses the global rate constant k instead of the specific rate constant k_{E}. This takes into account, that a thermal back isomerization ($Z \rightarrow E$) always occurs towards reaching an equilibrium between two species. The thermal equilibration Gibbs energy of activation $\Delta G^{\ddagger}_{\mathrm{E}\leftrightarrow\mathrm{Z\ equil.}}$ ((54)) is just used for extrapolation of the thermal equilibration half-life time at 25 °C ((55)).

$$\Delta G^{\ddagger}_{\mathrm{E}\leftrightarrow\mathrm{Z\ equil.}} = -RT\ln\left(\frac{\mathrm{h}k}{\mathrm{k_B}T}\right) \tag{54}$$

$$t_{1/2}^{\mathrm{eq}\ 25\ ^{\circ}\mathrm{C}} = \frac{\ln 2}{k_{25\ ^{\circ}\mathrm{C}}} \tag{55}$$

$$k_{25\ ^{\circ}\mathrm{C}} = \frac{\mathrm{k_B} T_{25\ ^{\circ}\mathrm{C}}}{\mathrm{h}} \mathrm{e}^{\frac{-\Delta G^{\ddagger}_{\mathrm{E}\leftrightarrow\mathrm{Z}}}{\mathrm{R}T_{25\ ^{\circ}\mathrm{C}}}} \tag{56}$$

$t_{1/2}^{\mathrm{eq}\ 25\ ^{\circ}\mathrm{C}}$ = extrapolated equilibration half-life time of thermal isomerization from species *E* → *Z* at 25 °C in s

$k_{25\ ^{\circ}C}$ = extrapolated global rate constant for the thermal isomerization *E* → *Z* and *Z* → *E* at 25 °C in s^{-1}

$\mathrm{k_B}$ = Boltzmann constant (1.381×10^{-23} J K^{-1})

$T_{25\ ^{\circ}C}$ = absolute temperature at 25 °C in K

h = Planck constant (6.626×10^{-34} J s)

$\Delta G^{\ddagger}_{\mathrm{E}\leftrightarrow\mathrm{Z\ equil.}}$ = thermal equilibration Gibbs energy of activation derived from global rate constant *k* at experiment temperature for the combined thermal isomerization from *E* → *Z* and thermal back isomerization from *Z* → *E* in J mol^{-1}

Calculation of the Gibbs energy of activation for the sole thermal isomerization from *E* → *Z* $\Delta G^{\ddagger}{}_{\mathrm{E}\rightarrow\mathrm{Z}}$ ((58), note that this is not the composite $\Delta G^{\ddagger}_{\mathrm{E}\leftrightarrow\mathrm{Z\ equil.}}$) employs the Eyring equation ((57)) at the experimental temperature *T* and uses the previously determined rate constant k_{E} and the following physical constants.

$$k_{\mathrm{E}} = \frac{\mathrm{k_B} T}{\mathrm{h}} \mathrm{e}^{\frac{-\Delta G^{\ddagger}_{\mathrm{E}\rightarrow\mathrm{Z}}}{\mathrm{R}T}} \tag{57}$$

$$\Delta G^{\ddagger}_{\mathrm{E}\rightarrow\mathrm{Z}} = -\mathrm{R}T\ln\left(\frac{\mathrm{h}k_{\mathrm{E}}}{\mathrm{k_B}T}\right) \tag{58}$$

$\Delta G^{\ddagger}_{\mathrm{E}\rightarrow\mathrm{Z}}$ = Gibbs energy of activation for thermal isomerization of species $E \rightarrow Z$ in J mol^{-1}

T = absolute temperature during measurement in K

The Gibbs free energy difference $\Delta G_{\mathrm{E}\rightarrow\mathrm{Z}}$ between the two interconverting isomeric species E and Z at thermal equilibrium can be calculated according to (59).

$$\Delta G_{\mathrm{E}\rightarrow\mathrm{Z}} = -\mathrm{R}T\ln\left(\frac{[Z_{\mathrm{eq}}]}{[E_{\mathrm{eq}}]}\right) = -\mathrm{R}T\ln(K) \tag{59}$$

$\Delta G_{\mathrm{E}\rightarrow\mathrm{Z}}$ = Gibbs free energy difference between the interconverting species E and Z in J mol^{-1}

The Gibbs energy of activation for thermal $Z \rightarrow E$ conversion ((60)) was calculated from the Gibbs energy of activation for thermal $E \rightarrow Z$ conversion ((58)) and the Gibbs free energy difference of the interconverting species E and Z ((59)).

$$G^{\ddagger}_{\mathrm{Z}\rightarrow\mathrm{E}} = G^{\ddagger}_{\mathrm{E}\rightarrow\mathrm{Z}} - \Delta G_{\mathrm{E}\rightarrow\mathrm{Z}} \tag{60}$$

$\Delta G^{\ddagger}_{\mathrm{Z}\rightarrow\mathrm{E}}$ = Gibbs energy of activation for thermal isomerization of species $Z \rightarrow E$ in J mol^{-1}

Supplementary Table 6: Experimentally determined Gibbs energies of activation ($\Delta G^{\ddagger}_{E\rightarrow Z}$ and $\Delta G^{\ddagger}_{Z\rightarrow E}$) for thermal *E* to *Z* and *Z* to *E* isomerizations at temperatures between 0 °C to 135 °C; corresponding half-life times at measurement temperatures ($t^{eq}_{1/2}$); linearly extrapolated half-life times at 25 °C ($t^{eq\,25\,°C}_{1/2}$); Gibbs free energy differences (ΔG) between *E* and *Z* isomers; and isomeric ratios (*E* : *Z*) at thermal equilibrium at measurement temperatures.

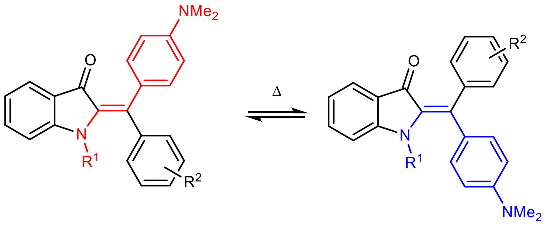

bathochromic *E*/*Z*-HI   hypsochromic *Z*/*E*-HI

HI	$\Delta G^{\ddagger}_{E\rightarrow Z}$ (kcal mol^{-1})	$\Delta G^{\ddagger}_{Z\rightarrow E}$ (kcal mol^{-1})	ΔG (kcal mol^{-1})	$t^{eq}_{1/2}$	$t^{eq\,25\,°C}_{1/2}$	*E* : *Z* at thermal equilibrium [%]
**1a**[B]	25.0[E]	25.3[F]	0.3	25 min (60 °C)	1.5 d	39 : 61 (60 °C)
**1b**[A]	31.2[E]	31.4[F]	0.3	39 min (135 °C)	118 a	42 : 58 (135 °C)
**1c**[A]	31.4[E]	31.9[F]	0.5	56 min (135 °C)	199 a	34 : 66 (135 °C)
**2a**[B]	-[C]	-[C]	-[C]	-[C]	-[C]	-[C]
**2b**[B]	22.9[E]	22.5[F]	0.4	36 min (25 °C)	36 min	67 : 33 (25 °C)
**2c**[B]	29.2[E]	27.9[F]	1.3	4 h (80 °C)	291 d	87 : 13 (80 °C)
**3a**[A]	-[D]	-[D]	0.1	-[D]	-[D]	47 : 53 (0 °C)
**3b**[B]	27.4[E]	27.5[F]	0.1	1.2 h (80 °C)	74 d	46 : 55 (80 °C)
**3c**[B]	24.5[E]	24.6[F]	0.1	10 min (60 °C)	14 h	48 : 52 (60 °C)
**4a**[B]	-[C]	-[C]	-[C]	-[C]	-[C]	-[C]
**4b**[B]	26.5[F]	27.4[E]	0.8	32 min (80 °C)	27 d	23 : 77 (80 °C)
**4c**[B]	26.8[F]	27.5[E]	0.7	45 min (80 °C)	40 d	26 : 74 (80 °C)

A: *o*-xylene-d_{10} as solvent. **B:** toluene-d_8 as solvent. **C:** Enrichment of bathochromic isomer by irradiation with light and thermal decay of enriched isomer measured at 0 °C. Values not determined, as photodegradation occurred. **D:** Values not determined, as no change in isomeric composition during thermal isomerization experiment was detected. **E:** $\Delta G^{\ddagger}$ values were determined through (60). **F:** Bathochromic isomer was enriched by irradiation with light, thermal decay of enriched isomer was measured, and corresponding $\Delta G^{\ddagger}$ values were determined through (58).

7.1 Thermal isomerization of diaryl HI 1a

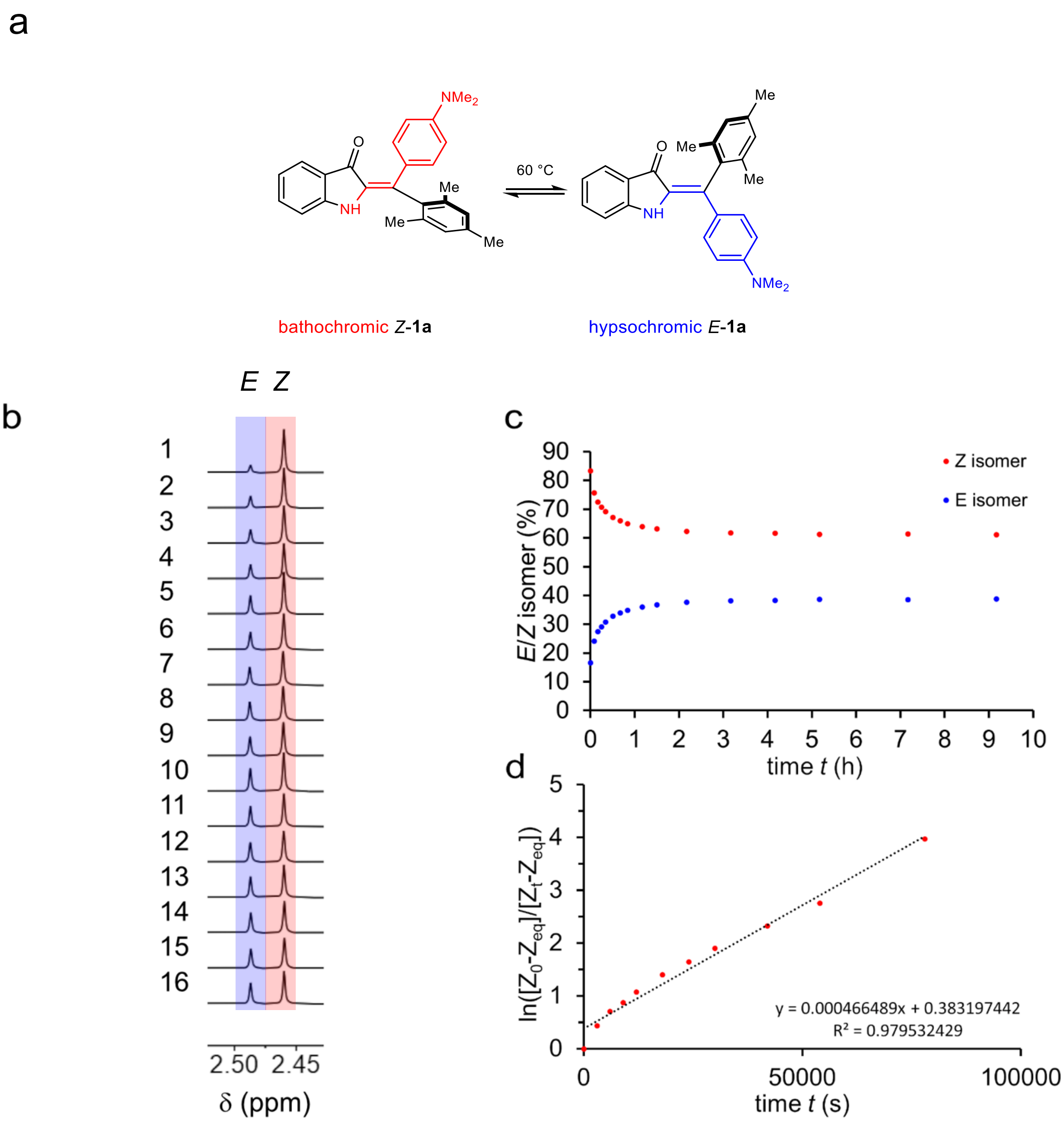

Supplementary Figure 93: Thermal isomerization experiment starting from a bathochromic *Z* isomer enriched solution of diaryl-HI **1a** at 60 °C in toluene-*d*8. To achieve *Z* isomer enrichment, a solution of diaryl-HI **1a** was first irradiated with 450 nm light resulting in an isomeric mixture of *E* : *Z* = 17% : 83%. **a** Schematic illustration of thermal *Z* to *E* isomerization of diaryl-HI **1b** at 60 °C. **b** Partial ^{1}H NMR spectra (400 MHz, *o*-xylene-*d*10, 23 °C) 1 – 16 recorded during the thermal isomerization experiment from bathochromic *Z*-**1a** to hypsochromic *E*-**1a**. Spectra were recorded after different time intervals corresponding to the plotted kinetics of c and d. The top spectrum 1 was recorded after photochemical enrichment of bathochromic *Z* isomer to 93% at the beginning of the heating, the last spectrum 16 was recorded after 9 h 20 min of heating. **c** Thermally induced decrease of bathochromic *Z*-**1a** and corresponding increase of hypsochromic *E*-**1a**. **d** Linearized kinetic plot of decreasing bathochromic *Z*-**1a** according to (49) resulting in a thermodynamic equilibrium with isomer composition *E* : *Z* = 39% : 61%. Source data are provided as Source Data File.

7.2 Thermal isomerization of diaryl-HI 1b

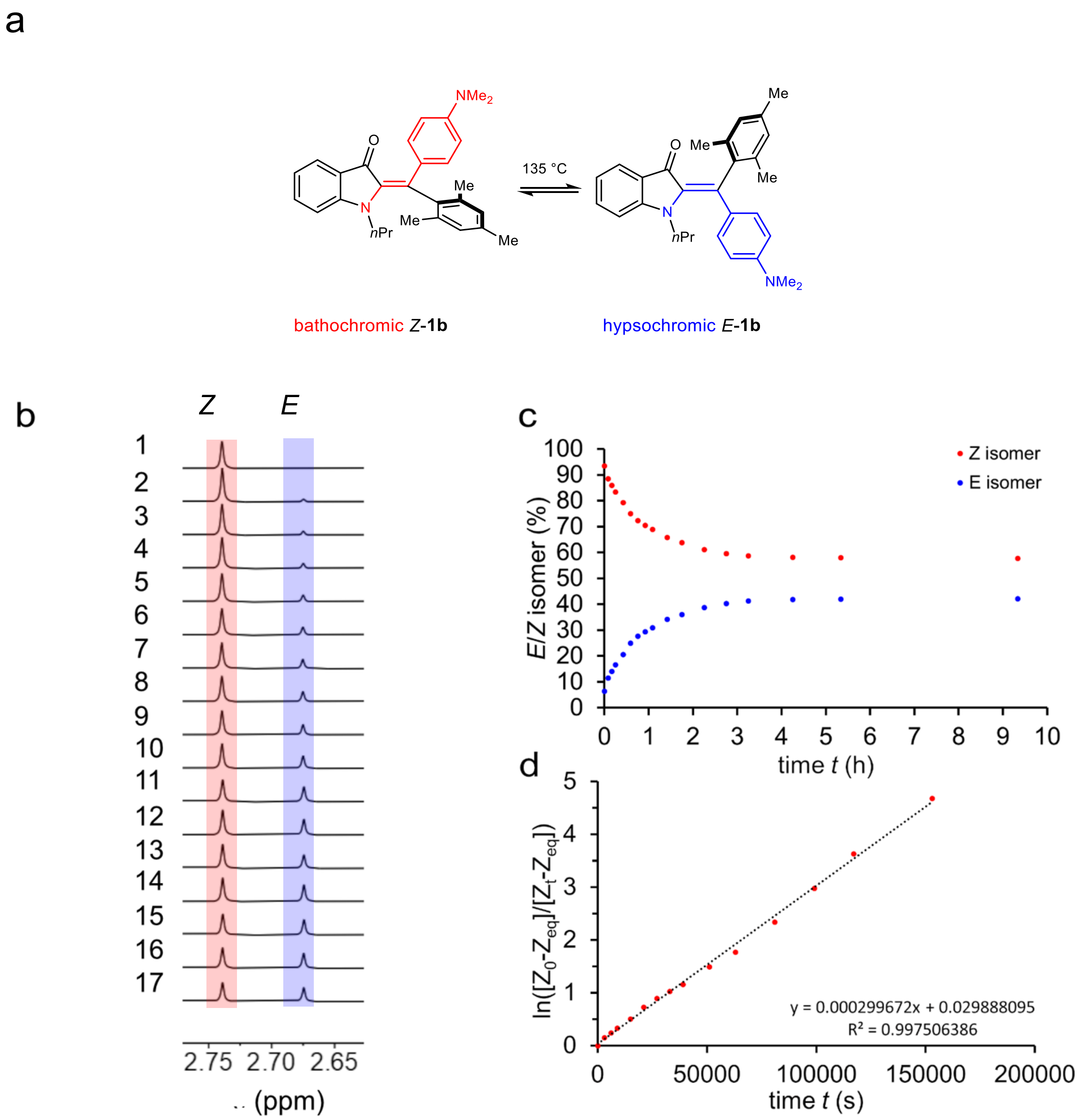

Supplementary Figure 94: Thermal isomerization experiment starting from a bathochromic *Z* isomer enriched solution of diaryl-HI **1b** at 135 °C in *o*-xylene-d_{10}. To achieve *Z* isomer enrichment, a solution of diaryl-HI **1b** was first irradiated with 470 nm light resulting in an isomeric mixture of *E* : *Z* = 7% : 93%. **a** Schematic illustration of thermal *Z* to *E* isomerization of diaryl-HI **1b** at 135 °C. **b** Partial ^{1}H NMR spectra (400 MHz, *o*-xylene-d_{10}, 23 °C) 1 – 17 recorded during the thermal isomerization experiment from bathochromic *Z*-**1b** to hypsochromic *E*-**1b**. Spectra were recorded after different time intervals corresponding to the plotted kinetics of c and d. The top spectrum 1 was recorded after photochemical enrichment of bathochromic *Z* isomer to 93% at the beginning of the heating, the last spectrum 17 was recorded after 9 h 20 min of heating. **c** Thermally induced decrease of bathochromic *Z*-**1b** and corresponding increase of hypsochromic *E*-**1b**. **d** Linearized kinetic plot of decreasing bathochromic *Z*-**1b** according to (49) resulting in a thermodynamic equilibrium with isomer composition *E* : *Z* = 42% : 58%. Source data are provided as Source Data File.

7.3 Thermal isomerization of diaryl-HI 1c

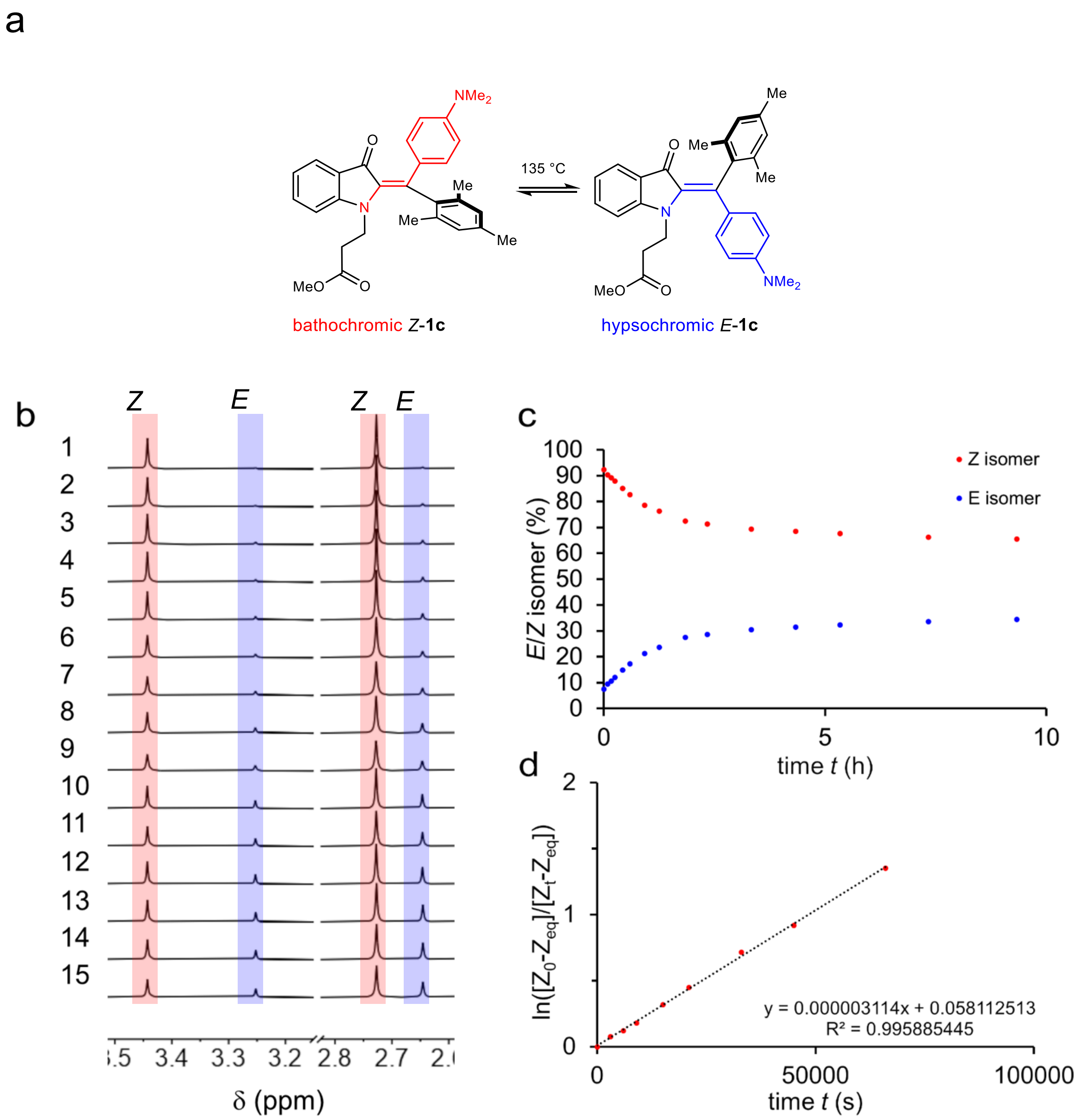

Supplementary Figure 95: Thermal isomerization experiment starting from a bathochromic *Z* isomer enriched solution of diaryl-HI **1c** at 135 °C in *o*-xylene-d_{10}. To achieve *Z* isomer enrichment, a solution of diaryl-HI **1c** was first irradiated with 470 nm light resulting in an isomeric mixture of *E* : *Z* = 8% : 92%. **a** Schematic illustration of thermal *Z* to *E* isomerization of diaryl-HI **1c** at 135 °C. **b** Partial ^{1}H NMR spectra (400 MHz, *o*-xylene-d_{10}, 23 °C) 1 – 15 recorded during the thermal isomerization experiment from bathochromic *Z*-**1c** to hypsochromic *E*-**1c**. Spectra were recorded after different time intervals corresponding to the plotted kinetics of c and d. The top spectrum 1 was recorded after photochemical enrichment of bathochromic *Z* isomer to 92% at the beginning of the heating, the last spectrum 15 was recorded after 9 h 20 min of heating. **c** Thermally induced decrease of bathochromic *Z*-**1c** and corresponding increase of hypsochromic *E*-**1c**. **d** Linearized kinetic plot of decreasing bathochromic *Z*-**1c** according to (49) resulting in a thermodynamic equilibrium with isomer composition *E* : *Z* = 34% : 66%. Source data are provided as Source Data File.

7.4 Thermal isomerization of diaryl-HI 2a

a

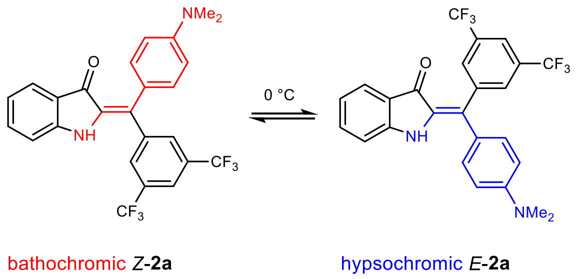

b

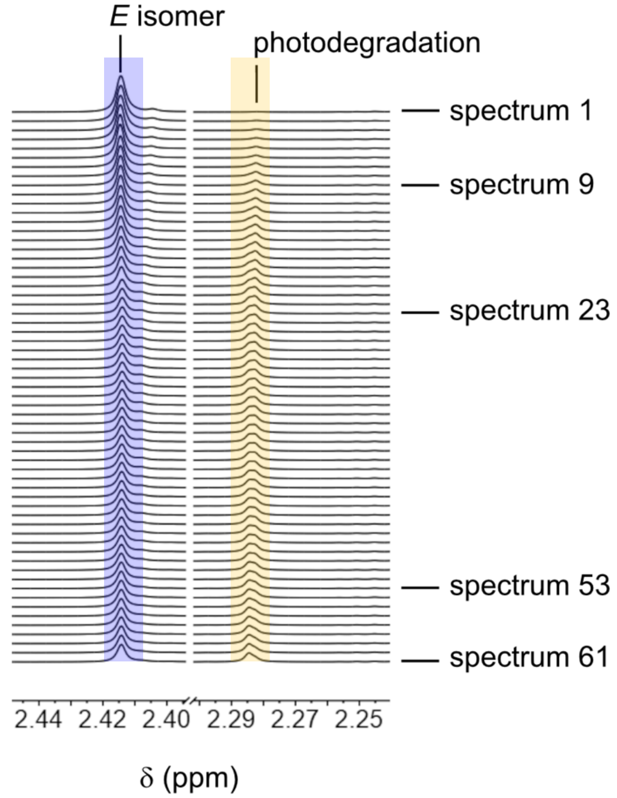

Supplementary Figure 96: Thermal isomerization experiment with the intention to start from a bathochromic *Z* isomer enriched solution of diaryl-HI **2a** at 0 °C in toluene-*d*8. To achieve *Z* isomer enrichment prior to thermal isomerization, the hypsochromic enriched *E* isomer solution of diaryl-HI **2a** was irradiated with 470 nm light. Instead of photochemical enrichment of the bathochromic *Z* isomer of **2a**, photodegradation occurred. **a** Schematic illustration of thermal *Z* to *E* isomerization of diaryl-HI **2a** at 0 °C. **b** ^{1}H NMR spectra (400 MHz, toluene-d_8, 0 °C) of thermal isomerization experiment. The start spectrum 1 has an isomeric composition of *E* : *Z* of 95% : 5%. Between spectrum 1 and spectrum 9, the NMR tube was irradiated over 50 min with light of 470 nm. After about 11 min photodegradation occurred and no *Z* isomer enrichment was detected. Between spectrum 9 and spectrum 23 every 5 min a spectrum was measured during ongoing photodegradation by irradiation. Between spectrum 23 and spectrum 53 every min a spectrum was measured without irradiation. No change in composition was detected. Between spectrum 53 and spectrum 61 every 5 min a spectrum was measured without irradiation. The isomeric composition was stable, even at 23 °C. Source data are provided as Source Data File.

7.5 Thermal isomerization of diaryl-HI 2b

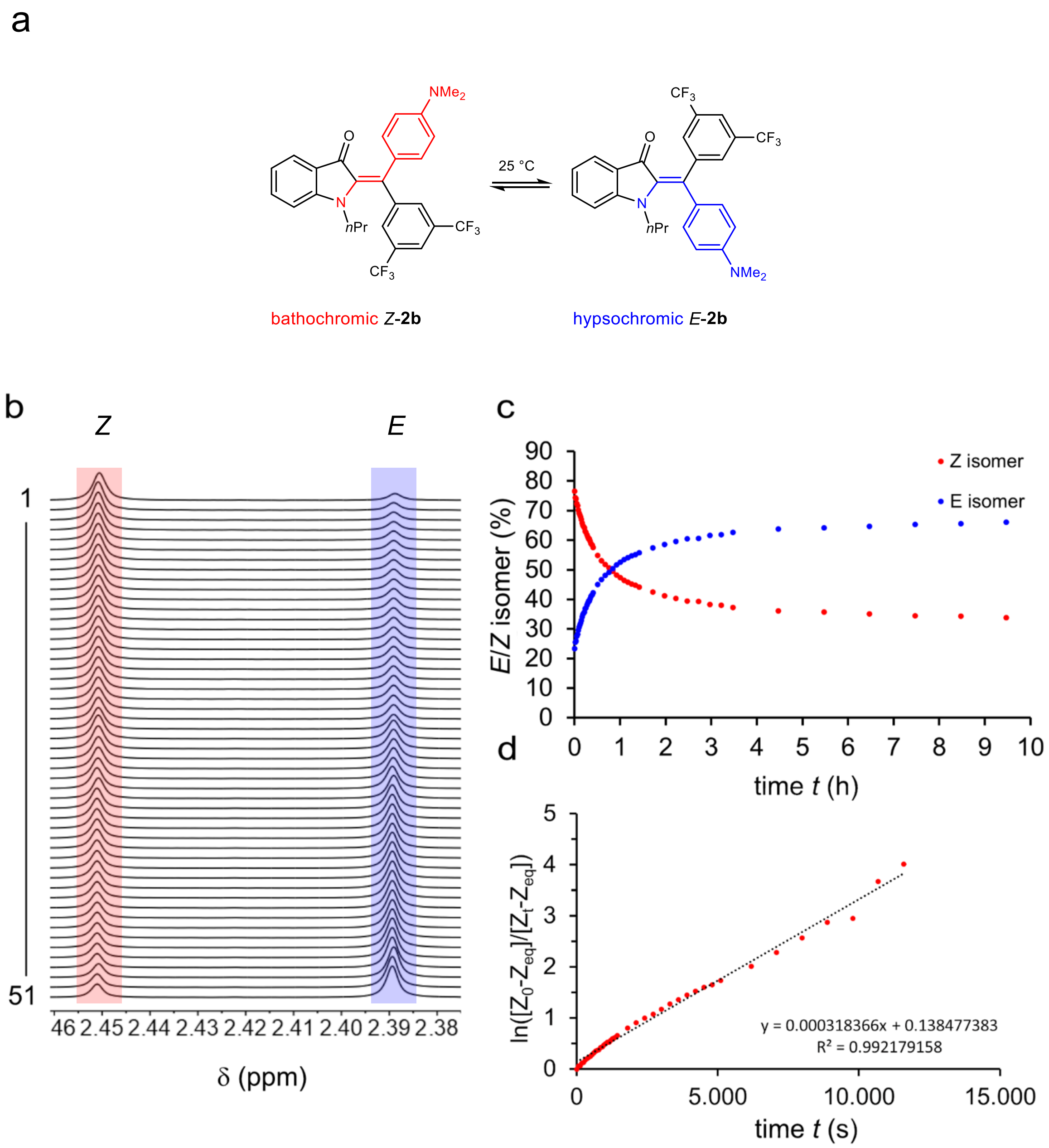

Supplementary Figure 97: Thermal isomerization experiment starting from a bathochromic *Z* isomer enriched solution of diaryl-HI **2b** at 25 °C in toluene-d_8. To achieve *Z* isomer enrichment, a solution of diaryl-HI **2b** was first irradiated with 490 nm light resulting in an isomeric mixture of *E* : *Z* = 23% : 77%. **a** Schematic illustration of thermal *Z* to *E* isomerization of diaryl-HI **2b** at 25 °C. **b** Partial ^{1}H NMR spectra (400 MHz, toluene-d_8, 25 °C) 1 – 51 recorded during the thermal isomerization experiment from bathochromic *Z*-**2b** to hypsochromic *E*-**2b**. Spectra were recorded after different time intervals corresponding to the plotted kinetics of c and d. The top spectrum 1 was recorded after photochemical enrichment of bathochromic *Z* isomer to 77% at the beginning of the thermal isomerization, the last spectrum 51 was recorded after 10 h at 25 °C. **c** Thermally induced decrease of bathochromic *Z*-**2b** and corresponding increase of hypsochromic *E*-**2b**. **d** Linearized kinetic plot of decreasing bathochromic *Z*-**2b** according to (49) resulting in a thermodynamic equilibrium with isomer composition *E* : *Z* = 67% : 33%. Source data are provided as Source Data File.

7.6 Thermal isomerization of diaryl-HI 2c

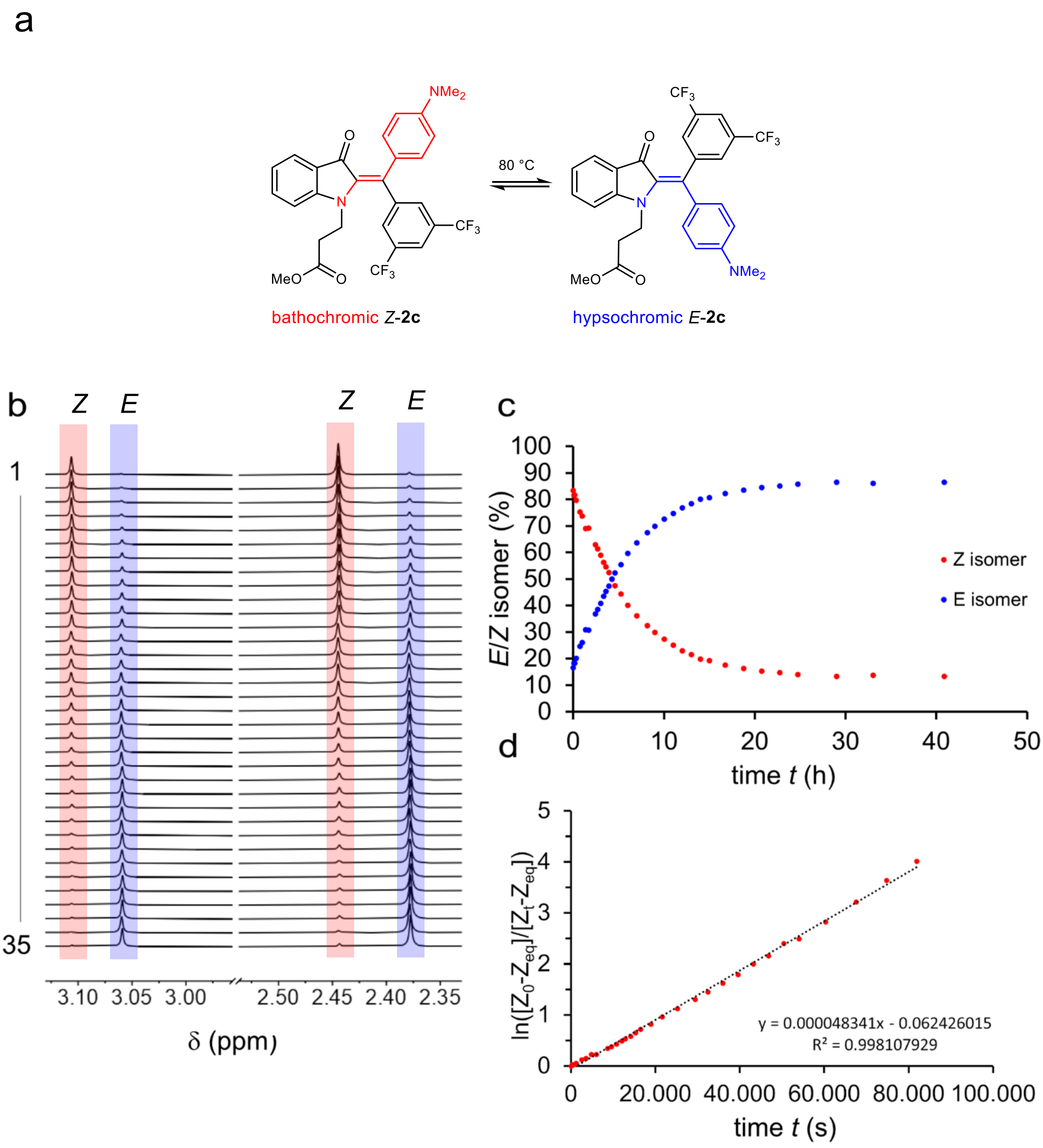

Supplementary Figure 98: Thermal isomerization experiment starting from a bathochromic *Z* isomer enriched solution of diaryl-HI **2c** at 80 °C in toluene-*d*8. To achieve *Z* isomer enrichment, a solution of diaryl-HI **2c** was first irradiated with 470 nm light resulting in an isomeric mixture of *E* : *Z* = 17% : 83%. **a** Schematic illustration of thermal *Z* to *E* isomerization of diaryl-HI **2c** at 80 °C. **b** Partial ^{1}H NMR spectra (400 MHz, toluene-*d*8, 23 °C) 1 – 35 recorded during the thermal isomerization experiment from bathochromic *Z*-**2c** to hypsochromic *E*-**2c**. Spectra were recorded after different time intervals corresponding to the plotted kinetics of c and d. The top spectrum 1 was recorded after photochemical enrichment of bathochromic *Z* isomer to 83% at the beginning of the heating, the last spectrum 35 was recorded after 40 h 50 min of heating. **c** Thermally induced decrease of bathochromic *Z*-**2c** and corresponding increase of hypsochromic *E*-**2c**. **d** Linearized kinetic plot of decreasing bathochromic *Z*-**2c** according to (49) resulting in a thermodynamic equilibrium with isomer composition *E* : *Z* = 86% : 14%. Source data are provided as Source Data File.

7.7 Thermal isomerization of diaryl-HI 3a

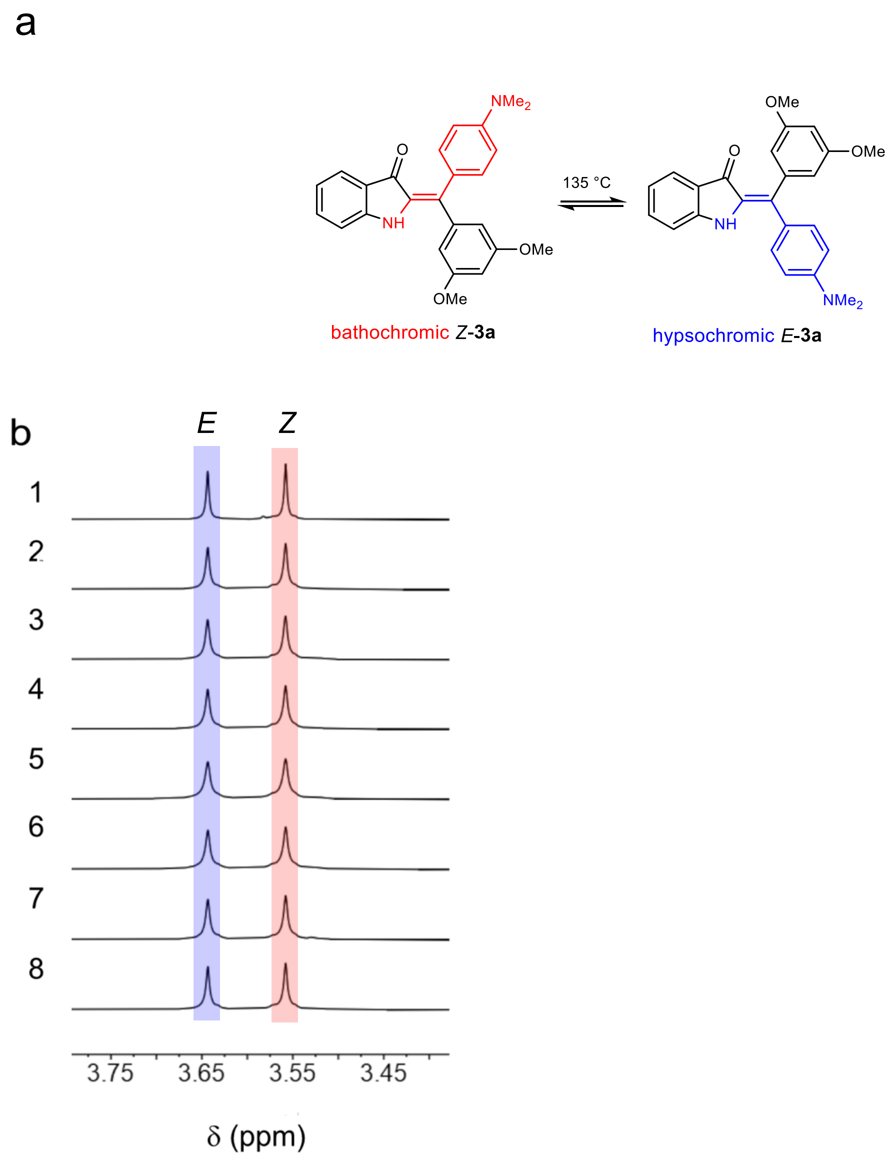

Supplementary Figure 99: Thermal isomerization experiment starting from a bathochromic *Z* isomer enriched solution of diaryl-HI **3a** at 135 °C in *o*-xylene-d_{10}.To achieve *Z* isomer enrichment, a solution of diaryl-HI **3a** was first irradiated with 450 nm light resulting in an isomeric mixture of *E* : *Z* = 43% : 57%. **a** Schematic illustration of thermal *Z* to *E* isomerization of diaryl-HI **3a** at 135 °C. **b** Partial ^{1}H NMR spectra (400 MHz, *o*-xylene-d_{10}, 23 °C) 1 – 8 recorded during the thermal isomerization experiment. The top spectrum 1 was recorded after photochemical enrichment of bathochromic *Z* isomer to 57% at the beginning of the heating, the last spectrum 8 was recorded after 2 h 5 min heating at 135 °C. No change in isomeric composition was observed. The same result was obtained after heating a sample of a *Z*-**3** enriched solution in toluene-d_8 for 90 h 40 min at 100 °C. Source data are provided as Source Data File.

7.8 Thermal isomerization of diaryl-HI 3b

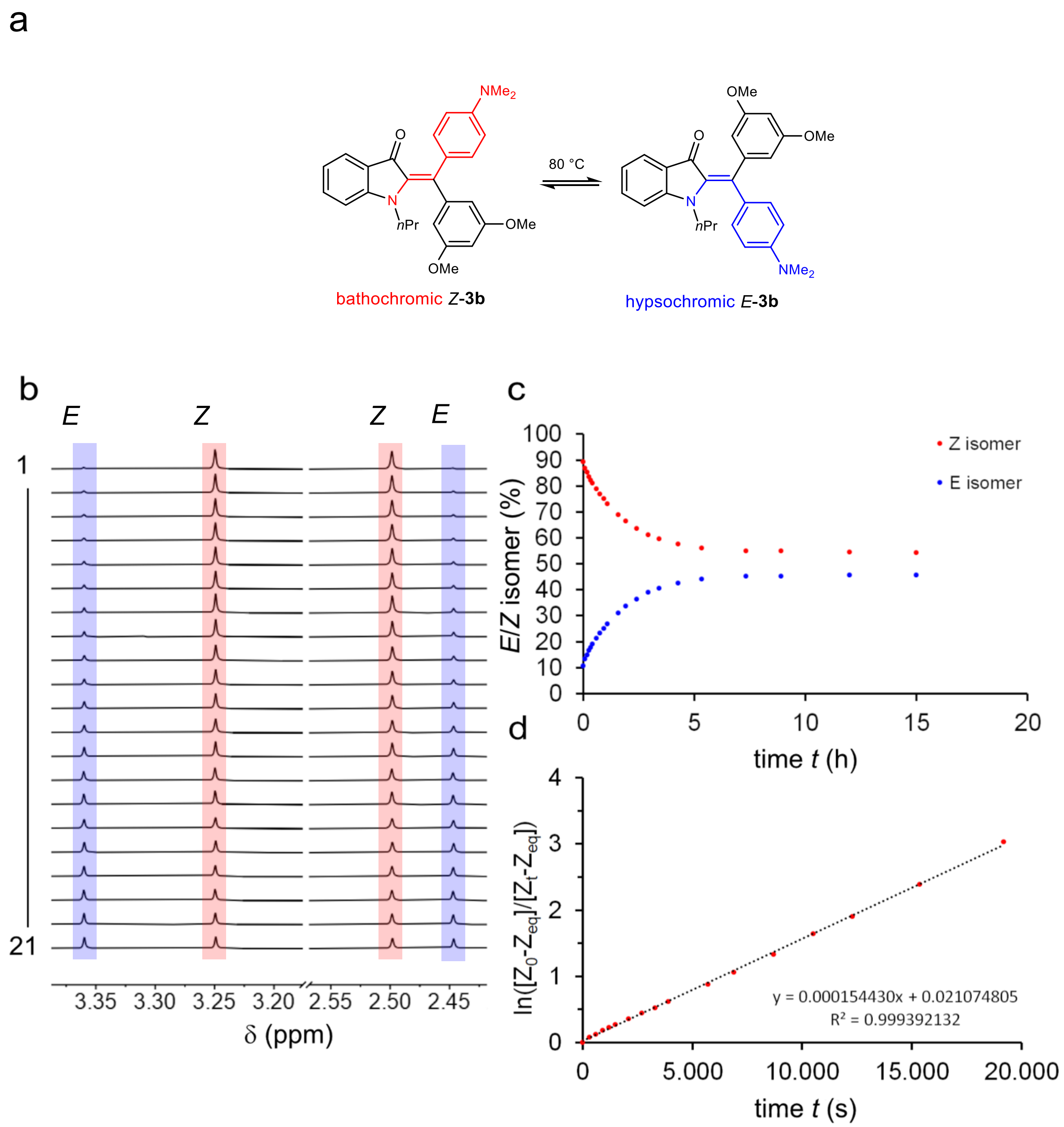

Supplementary Figure 100: Thermal isomerization experiment starting from a bathochromic Z isomer enriched solution of diaryl-HI **3b** at 80 °C in toluene-*d*8. To achieve *Z* isomer enrichment, a solution of diaryl-HI **3b** was first irradiated with 470 nm light resulting in an isomeric mixture of *E* : *Z* = 13% : 87%. **a** Schematic illustration of thermal *Z* to *E* isomerization of diaryl-HI **3b** at 80 °C. **b** Partial ^{1}H NMR spectra (400 MHz, toluene-*d*8, 23 °C) 1 - 21 recorded during the thermal isomerization experiment from bathochromic *Z*-**3b** to hypsochromic *E*-**3b**. Spectra were recorded after different time intervals corresponding to the plotted kinetics of c and d. The top spectrum 1 was recorded after photochemical enrichment of bathochromic *Z* isomer to 87% at the beginning of the heating, the last spectrum 21 was recorded after 15 h of heating. **c** Thermally induced decrease of bathochromic *Z*-**3b** and corresponding increase of hypsochromic *E*-**3b**. **d** Linearized kinetic plot of decreasing bathochromic *Z*-**3b** according to (49) resulting in a thermodynamic equilibrium with isomer composition *E* : *Z* = 46% : 55%. Source data are provided as Source Data File.

7.9 Thermal isomerization of diaryl-HI 3c

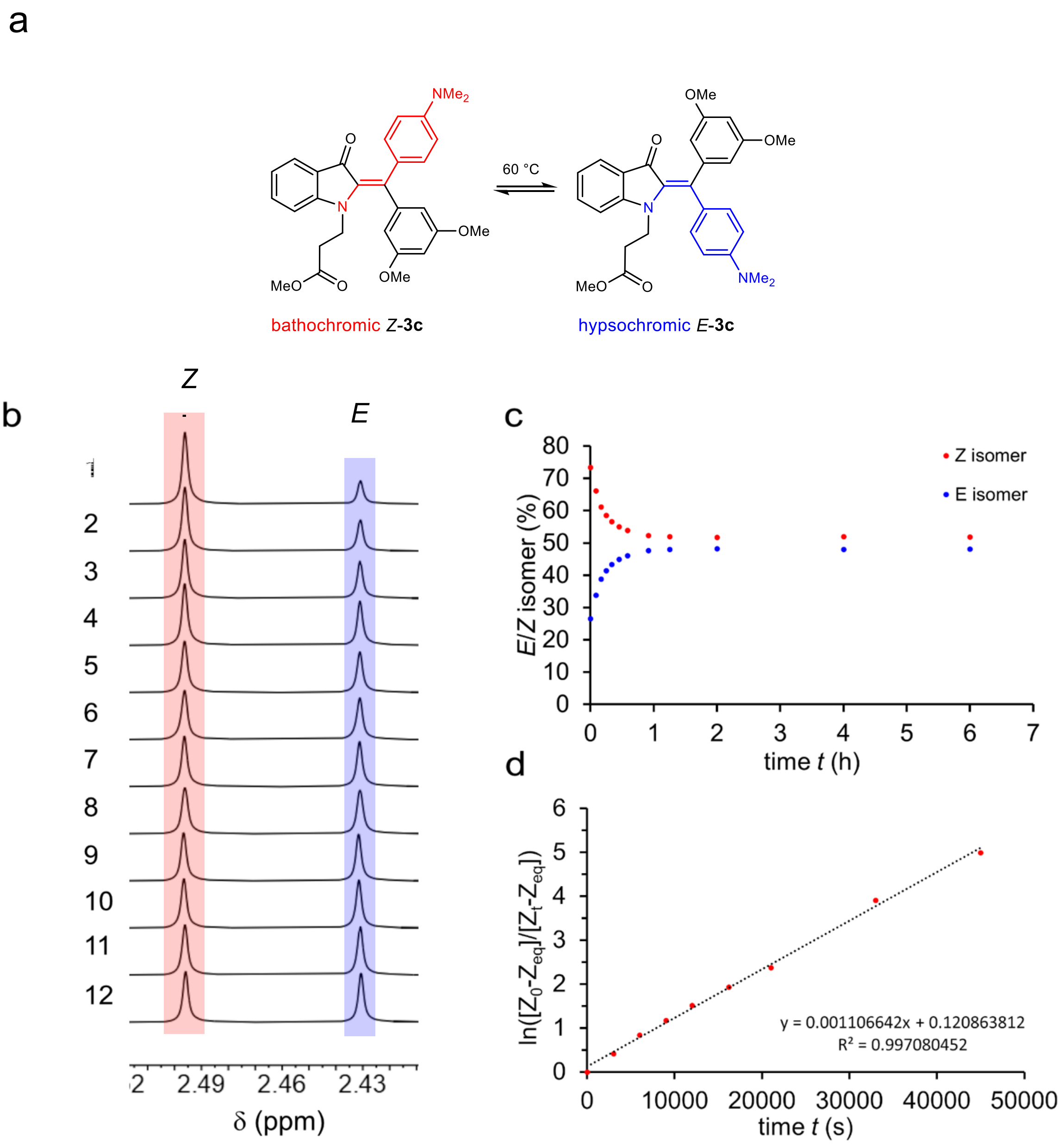

Supplementary Figure 101: Thermal isomerization experiment starting from a bathochromic *Z* isomer enriched solution of diaryl-HI **3c** at 60 °C in toluene-d_8. To achieve *Z* isomer enrichment, a solution of diaryl-HI **3c** was first irradiated with 470 nm light resulting in an isomeric mixture of *E* : *Z* = 26% : 74%. **a** Schematic illustration of thermal *Z* to *E* isomerization of diaryl-HI **3c** at 60 °C. **b** Partial ^{1}H NMR spectra (400 MHz, toluene-d_8, 23 °C) 1 – 12 recorded during the thermal isomerization experiment from bathochromic *Z*-**3c** to hypsochromic *E*-**3c**. Spectra were recorded after different time intervals corresponding to the plotted kinetics of c and d. The top spectrum 1 was recorded after photochemical enrichment of bathochromic *Z* isomer to 74% at the beginning of the heating, the last spectrum 12 was recorded after 6 h of heating. **c** Thermally induced decrease of bathochromic *Z*-**3c** and corresponding increase of hypsochromic *E*-**3c**. **d** Linearized kinetic plot of decreasing bathochromic *Z*-**3c** according to (49) resulting in a thermodynamic equilibrium with isomer composition *E* : *Z* = 48% : 52%. Source data are provided as Source Data File.

7.10 Thermal isomerization of diaryl-HI 4a

a

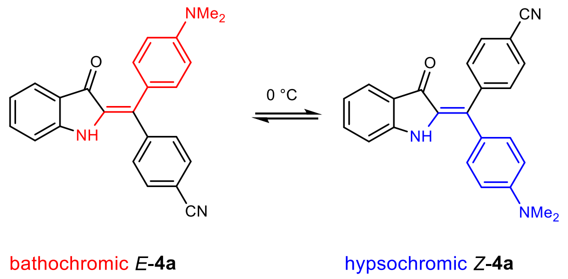

b

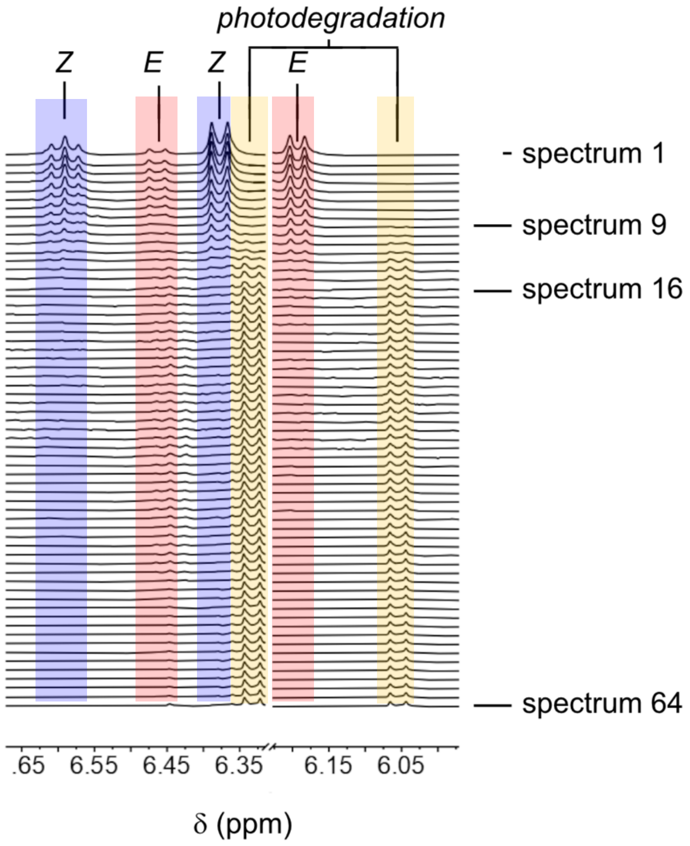

Supplementary Figure 102: Thermal isomerization experiment with the intention to start from a bathochromic *E* isomer enriched solution of diaryl-HI **4a** at 0 °C in toluene-*d*8. To achieve *E* isomer enrichment prior to thermal isomerization, the enriched hypsochromic *Z* isomer solution of diaryl-HI **4a** was irradiated with 450 nm. Instead of photochemical enrichment of the bathochromic *E* isomer of **4a**, photodegradation occurred. **a** Schematic illustration of thermal *E* to *Z* isomerization of diaryl-HI **4a** at 0 °C. **b** ^{1}H NMR spectra (400 MHz, toluene-d_8, 0 °C) of thermal isomerization experiment. No *E* isomer enrichment was detected during irradiation with 450 nm at 0 °C, but photodegradation occured. The start spectrum 1 has an isomeric composition of *E* : *Z* of 14% : 86%. Between spectrum 1 and spectrum 15, the NMR tube was irradiated over 141 min with light of 450 nm at 0 °C with spectra in 1 to 5 min intervals. Spectrum 9 shows the beginning of photodegradation. Between spectrum 16 and spectrum 64 spectra were measured over 17 h in 1 min, 5 min and 1 h intervals. The composition of the solution was stable at least 10 h at 23 °C. Source data are provided as Source Data File.

7.11 Thermal isomerization of diaryl-HI 4b

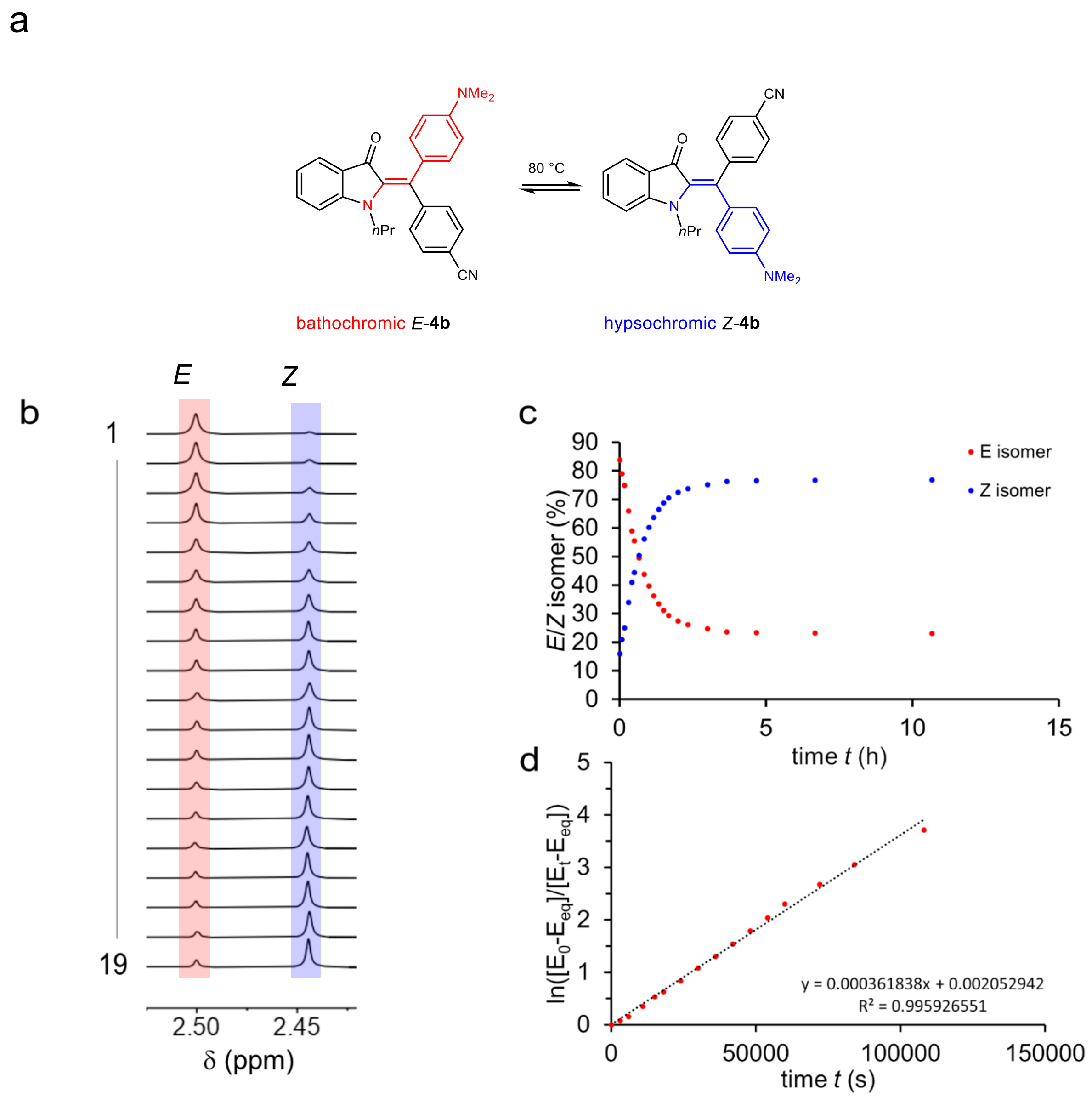

Supplementary Figure 103: Thermal isomerization experiment starting from a bathochromic *E* isomer enriched solution of diaryl-HI **4b** at 80 °C in toluene-d_8. To achieve *E* isomer enrichment, a solution of diaryl-HI **4b** was first irradiated with 505 nm light resulting in an isomeric mixture of *E* : *Z* = 84% : 16%. **a** Schematic illustration of thermal *E* to *Z* isomerization of diaryl-HI **4b** at 80 °C. **b)** Partial ^{1}H NMR spectra (400 MHz, toluene-d_8, 23 °C) 1 – 19 recorded during the thermal isomerization experiment from bathochromic *E*-**4b** to hypsochromic *Z*-**4b**. Spectra were recorded after different time intervals corresponding to the plotted kinetics of c and d. The top spectrum 1 was recorded after photochemical enrichment of bathochromic *E* isomer to 84% at the beginning of the heating, the last spectrum 19 was recorded after 10 h 40 min of heating. **c** Thermally induced decrease of bathochromic *E*-**4b** and corresponding increase of hypsochromic *Z*-**4b**. **d** Linearized kinetic plot of decreasing bathochromic *E*-**4b** according to (49) resulting in a thermodynamic equilibrium with isomer composition *E* : *Z* = 23% : 77%. Source data are provided as Source Data File.

7.12 Thermal isomerization of diaryl-HI 4c

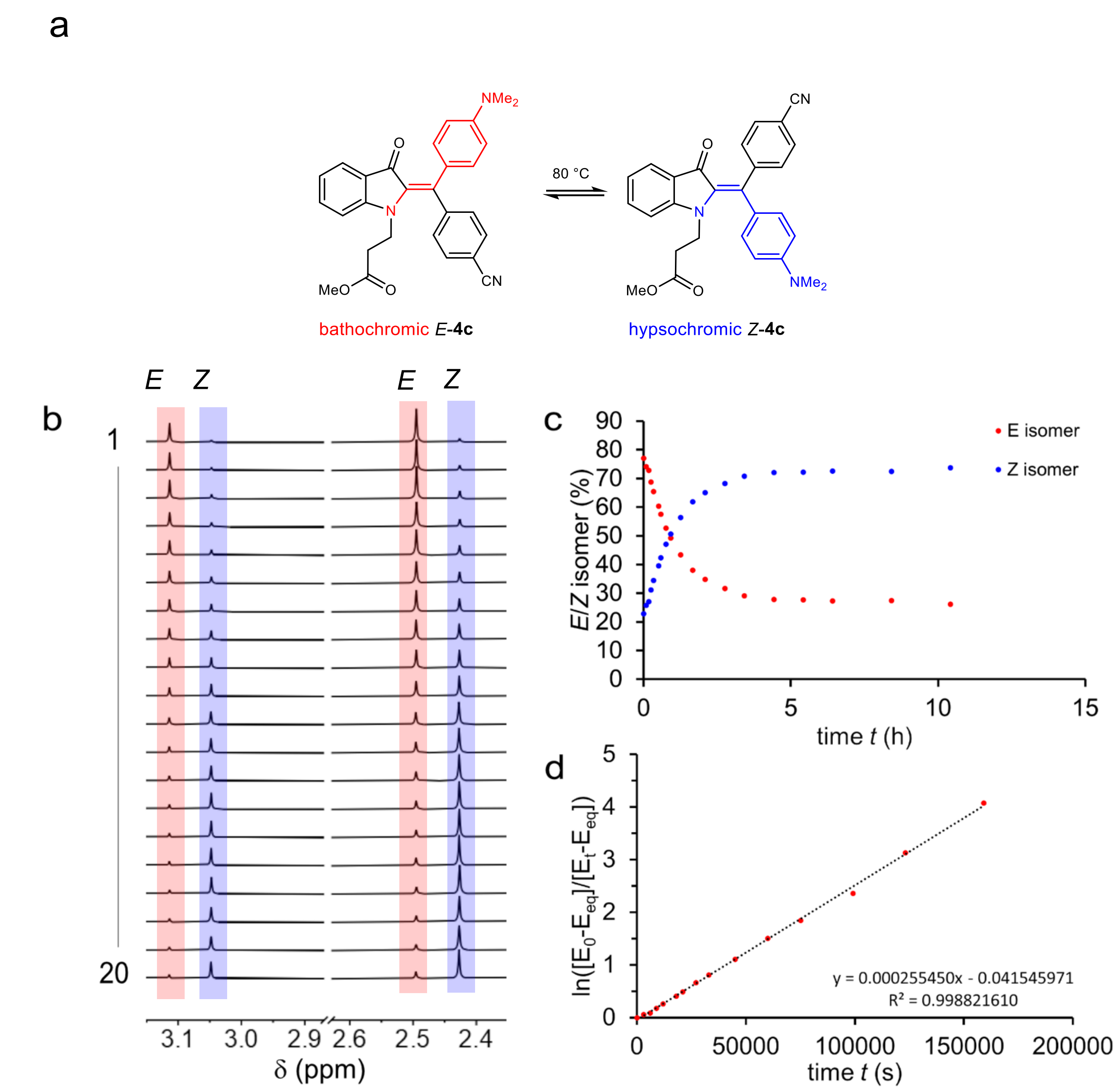

Supplementary Figure 104: Thermal isomerization experiment starting from a bathochromic *E* isomer enriched solution of diaryl-HI **4c** at 80 °C in toluene-*d*8. To achieve *E* isomer enrichment, a solution of diaryl-HI **4c** was first irradiated with 470 nm light resulting in an isomeric mixture of *E* : *Z* = 77% : 23%. **a** Schematic illustration of thermal *E* to *Z* isomerization of diaryl-HI **4c** at 80 °C. **b** Partial ^{1}H NMR spectra (400 MHz, toluene-*d*8, 23 °C) 1 – 20 recorded during the thermal isomerization experiment from bathochromic *E*-**4c** to hypsochromic *Z*-**4c**. Spectra were recorded after different time intervals corresponding to the plotted kinetics of c and d. The top spectrum 1 was recorded after photochemical enrichment of bathochromic *E* isomer to 77% at the beginning of the heating, the last spectrum 20 was recorded after 10 h 25 min of heating. **c** Thermally induced decrease of bathochromic *E*-**4c** and corresponding increase of hypsochromic *Z*-**4c**. **d** Linearized kinetic plot of decreasing bathochromic *E*-**4c** according to (49) resulting in a thermodynamic equilibrium with isomer composition *E* : *Z* = 26% : 74%. Source data are provided as Source Data File.

8. Supplementary Note 8: Acid induced isomerization of diaryl-HIs

Diaryl-HI solutions of compouds **1-4** were prepared with spectroscopic concentrations of 1×10^{-4} to 6×10^{-5} mol L^{-1} in toluene (spectroscopic grade, UV/Vis cuvettes). For preparation of stock solutions 2 mL volumetric flasks were charged with 0.89 to 2.98 mg diaryl-HI sample weighted on a Sartorius Cubis® MSE2.7S ultrafine balance. For isomerization experiments in UV/Vis cuvettes, defined volumes of 50 to 300 µL were removed from the 2 mL stock solutions in toluene and dissolved in 2.50 mL toluene (spectroscopic grade). The samples of the respective diaryl-HIs in toluene with known concentrations were irradiated with LEDs of 450 nm to 625 nm at 5°C or 23 °C to the isomeric compositions at pss. Afterwards 0.1 equiv. to more than 1,000 equiv. of TFA were added and the change of absorbance was monitored subsequently.

The ^{1}H NMR titration experiment of compound **4b** is described later in the chapter, as well as the structural elucidation via NMR spectroscopy in TFA-d_1 and toluene-d_8.

For the experiments, LEDs from Roithner Lasertechnik GmbH and Thorlabs GmbH (450 nm, 1850 mW, 470 nm, 760 mW; 490 nm, 205 mW; 505 nm, 400 mW; 530 nm, 370 mW; 590 nm 230 mW; 625 nm, 700 mW) were used for irradiation of UV/Vis samples.

The results of acid/base induced switching between four distinct and spectroscopically different states is summarized in Supplementary Figure 105:

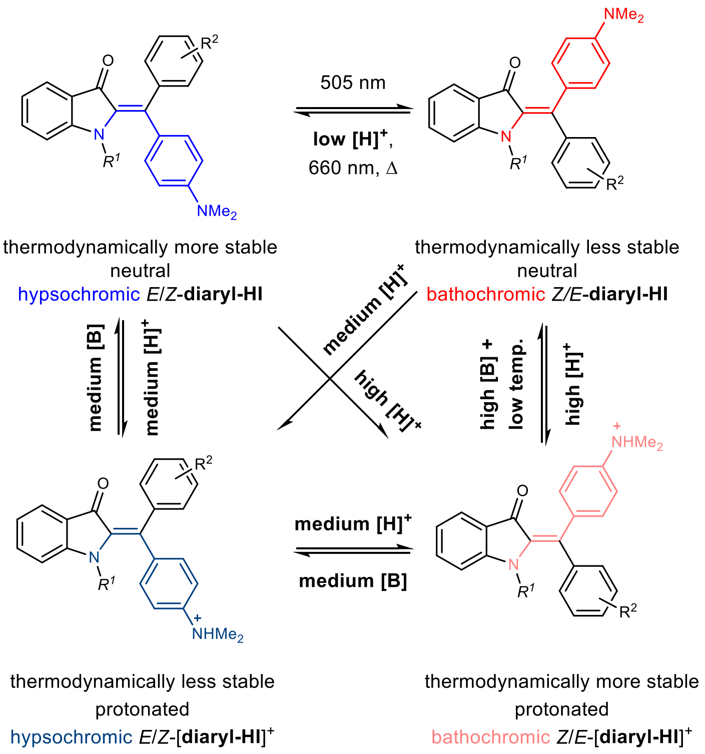

Supplementary Figure 105: Schematic illustration of the acid induced isomerization processes of diaryl-HIs that were elucidated by different experiments as described in the following. Source data are provided as Source Data File.

The effects of acid/base addition on the absorbance as well as on thermal isomerization from thermodynamically less stable bathochromic to thermodynamically more stable hypsochromic unprotonated diaryl-HI species and from thermodynamically less stable hypsochromic to thermodynamic more stable bathochromic protonated diaryl-HI species were monitored in a stepwise manner, and allowing for the following conclusions:

1. The here presented diaryl-HI species are all stable even in the presence of a large excess of added acid and can be restored upon neutralization with NEt_3.

2. Small amounts of acid up to 4.0 equiv. do not alter the absorption spectra significantly but have noticeable effects on the thermal bistability of diaryl-HIs. In general, the most stable isomer under neutral conditions is also preferentially populated in similar percentages under acidic conditions, evidencing that the thermodynamic stability of isomers is not changed after acid addition.

Diaryl-HIs **1** without cross-conjugation or a distinguished inductive donor-acceptor system remain thermally bistable under small amounts of added acid and reversible photoswitching remains unchanged. Diaryl-HIs without pronounced cross-conjugation (**2** and **3**) show moderately accelerated acid induced thermal isomerization, while strongly cross-conjugated diaryl-HIs **4** show the strongest response and the fastest acid induced isomerization.

It is also observed that acid induced isomerization is faster for systems with a free proton at the indoxyl nitrogen. It is slower for systems with a *-I* substituent at the indoxyl nitrogen and the slowest if there is a *+I* substituent at the indoxyl nitrogen.

The ability to photoisomerize can be retained when adding small amounts of acid. As depicted in the following, the isomerization speed is a function of acid amount (acid catalyzed). Therefore, the here presented diaryl-HIs can be used as a multi-triggered system allowing isomerization into one direction with light, and into the other direction with light and/or acid. The isomerization speed is not only a function of light intensity and wavelength but also of proton concentration.

What is more, the *E/Z* isomer composition can be adjusted by addition of small amounts of acid, followed by neutralization with a base. Due to the restored high thermal isomerization barriers, the *E*/*Z* isomer ratio can be kept constant over a long time after the neutralization. This observation allows an acid regulated *E/Z* ratio for the isomerization from the bathochromic to the hypsochromic species, which does not diminish the down streamed addressability of the species with light.

3. After stepwise addition of a larger excess of acid (up to 4,500 equiv.) thermal isomerizations are accelerated for all diaryl-HIs. A third and finally a fourth state is observed at increasing high acid concentrations with new characteristic absorbances. The third species lacks the absorbance band at around 420 nm, and possesses the lowest energy band at around 520 nm. The fourth species shows a small redshift of the lowest energy band as compared to the third state and a second band appearing again at around 360 nm. Because of this, the protonated states are distinct to the human eye as magenta in color. The third state is ascribed to the protonated form of the thermodynamically most stable isomer of the corresponding neutral form. The fourth state is ascribed to the protonated form of thermodynamically less stable isomer of the corresponding neutral form. These assignments were made based on the following UV/Vis and NMR spectroscopic experimental facts. 1. Stepwise acid addition to diaryl-HIs **2** - **4** leads to an observable, first enrichment of the thermodynamically most stable state without pronounced

absorbance changes and upon continued addition to formation of the third and fourth protonated state in UV/Vis experiments. 2. Neutralization of the protonated third state reestablishes the known thermodynamically most stable neutral isomer. Neutralization of the protonated fourth state regenerates the thermodynamically less stable neutral isomer (up to 91% in case of diaryl-HI **4b**). The neutral *E* and *Z* states have been assigned via NMR experiments and their isomerization change during protonation has been followed via an NMR titration experiment. The fourth state has been analyzed via NMR spectroscopy and can be assigned to the thermodynamically less stable isomer of the corresponding neutral form.

Therefore, the diaryl-HIs can be switched between four states (neutral *E* and *Z*, protonated *E* and *Z*) just by adjustment of acid and base concentration, a behavior akin to chemical fueling.

Supplementary Table 7: Wavelengths λ_{max} of the lowest energy absorbance maximum A_{max} recorded for diaryl-HIs **1-4** in toluene at 23 °C after application of different triggers. Column one lists pss λ_{max} values of enriched hypsochromic isomers obtained after irradiation with visible light, column two lists λ_{max} values of the thermal equilibrated *E*/*Z* isomer mixture, and column three lists λ_{max} values of the acid accelerated isomer equilibrated mixtures. The latter values show very good agreement with the values obtained for thermally equilibrated neutral diaryl-HI isomeric mixtures. All values given were determined using UV/Vis spectroscopy.

HI	λ_{max} of lowest energy band A_{max} at pss of hypsochromic enriched species (nm) (isomer enriched, LED emission nm)	λ_{max} of lowest energy band A_{max} at thermal equilibrium between both *E* and *Z* isomers (nm)	λ_{max} of lowest energy band A_{max} after addition of ≤ 4 equiv. $[H^+]$ (nm)
**1a**	478 (*E*, 590 nm)	484	484
**1b**	504 (*E*, 617 nm)	531	stable (537$^{\mathbf{B}}$)
**1c**	497 (*E*, 617 nm)	504	stable
**2a**$^{\mathbf{A}}$	-	-	-
**2b**	521 (*E*, 625 nm)	522	520
**2c**	511 (*E*, 625 nm)	510	510 (530$^{\mathbf{D}}$)
**3a**	488 (*E*, 530 nm)	488	489
**3b**	517 (*E*, 590 nm)	522	523 (536$^{\mathbf{B}}$)
**3c**	522 (*E*, 550 nm)	512	512
**4a**$^{\mathbf{A}}$	-	-	-
**4b**	522 (*Z*, 625 nm)	525	522 (561$^{\mathbf{D}}$, 524$^{\mathbf{C}}$)
**4c**	510 (*Z*, 625 nm)	510	511 (549$^{\mathbf{D}}$)

A: Photodegradation. **B:** λ_{max} at lowest energy A_{max} of diaryl-HI after addition of high excess of TFA (5,000 equiv.) and neutralization in one step with triethylamine. The absorption spectrum establishes 85% of the thermodynamically less stable *Z*-**1b**. **C:** λ_{max} at lowest energy A_{max} of diaryl-HI after high excess of TFA (up to 4,500 equiv.) and stepwise neutralization with triethylamine (up to 4,500 equiv.). **D:** λ_{max} at lowest energy A_{max} of diaryl-HI after addition of high excess of TFA (5,000 equiv.) and neutralization in one step with triethylamine at –79 °C. The absorption spectra establish 58% *Z*-**2c**, 91% *E*-**4b**, and 80% *E*-**4c**. Each isomer enriched compositons are thermodynamically less favoured.

8.1 Acid induced isomerization of diaryl-HI 1a

a

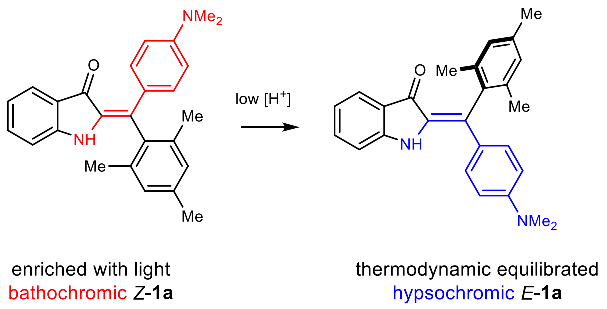

b

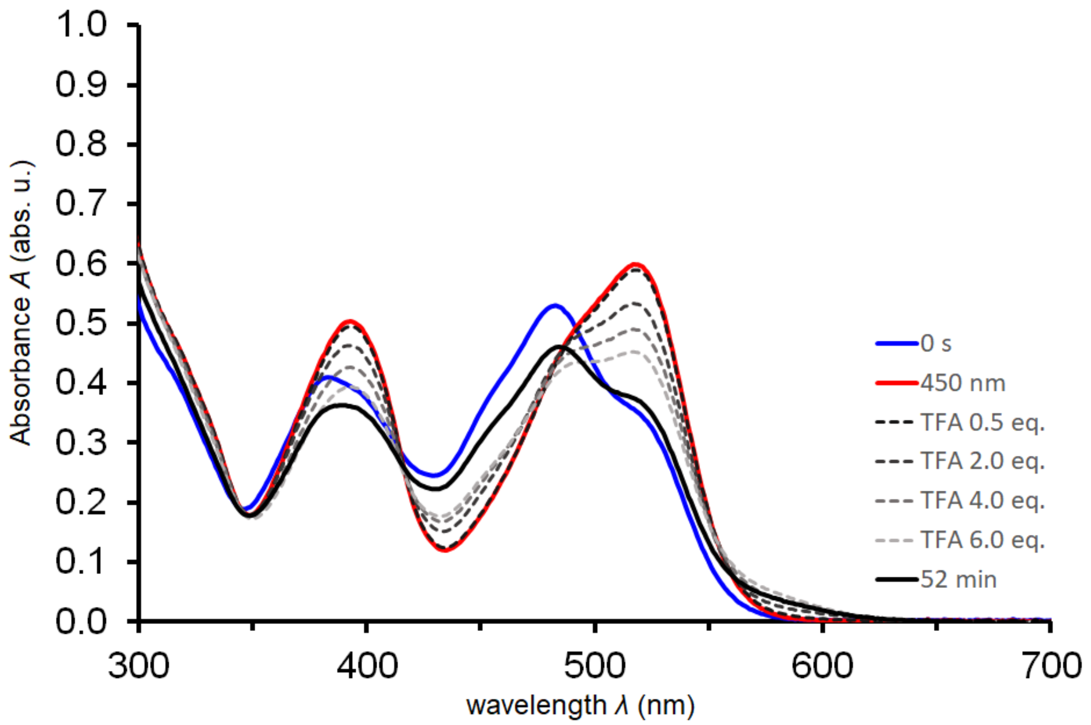

Supplementary Figure 106: a Schematic illustration of the acid induced isomerization from the bathochromic to the hypsochromic isomer of diaryl-HI **1a** (thermal equilibrium E = 39%, Z = 61%; hypsochromic pss: E = 47%; bathochromic pss: Z = 94%). **b** Absorption spectra of diaryl-HI **1a** in toluene solution at 23 °C recorded before (hypsochromic *E*-**1a** enriched, blue curve) and after irradiation with 450 nm light (bathochromic *Z*-**1a** enriched, red curve). Subsequent addition of up to 6.0 equiv. TFA leads to formation of hypsochromic *E*-**1a** within 52 min. The absence of isosbestic points is probably due to dilution effects and a change of solvent polarity during the experiment. Acid induced thermal isomerization is accelerated compared to thermal isomerization of neutral **1a**. Source data are provided as Source Data File.

8.2 Acid induced isomerization of diaryl-HI 1b

a

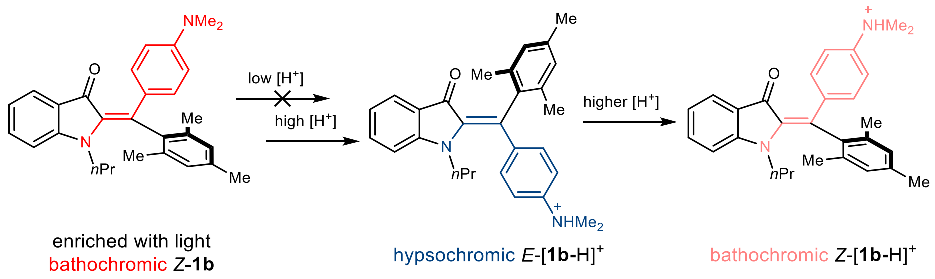

b

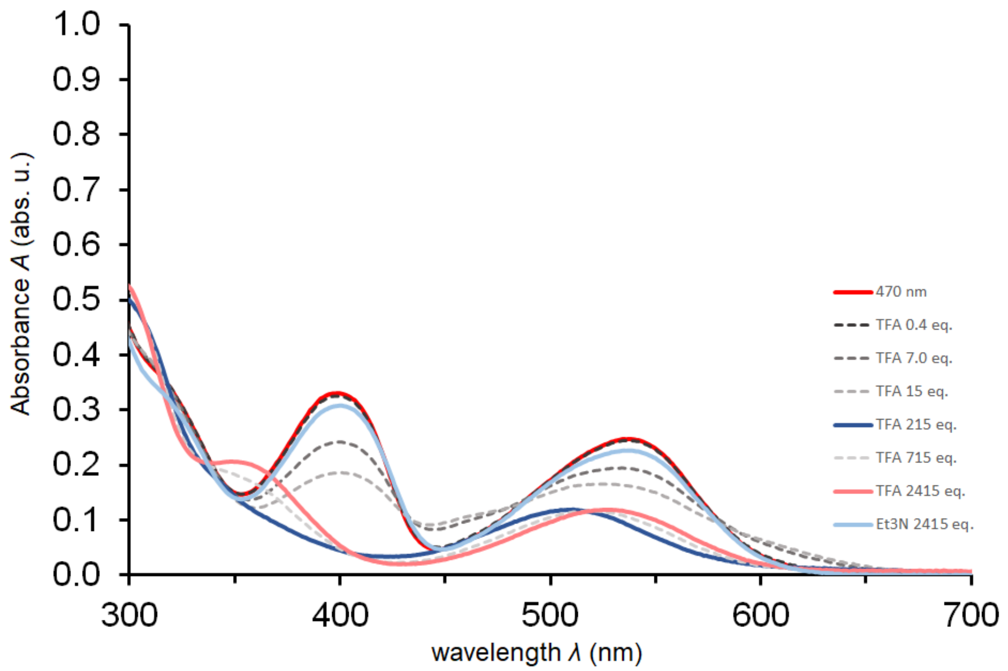

Supplementary Figure 107: a Schematic illustration of the acid/based induced isomerizations of diaryl-HI **1b**. Starting from the hypsochromic *E* isomer the protonated hypsochromic *E*-[**1b**-H]$^+$ isomer and protonated bathochromic *Z*-[**1b**-H]$^+$ isomer can be obtained (thermal equilibrium *E* = 42%, *Z* = 58%; hypsochromic pss: *E* = 62%; bathochromic pss: *Z* = 100%). **b** Absorption spectra of diaryl-HI **1b** in toluene solution at 23 °C recorded after irradiation with 470 nm light (bathochromic *Z*-**1b** enriched, red curve). Subsequent addition of up to 15 equiv. TFA does not lead to formation of appreciable amounts of the hypsochromic isomer. After addition of 315 to 2,415 equiv. TFA two new protonated species are formed, first the third species with hypsochromic shifted absorbance (cyano trace) and then the fourth species with slightly bathochromic shift (salmon red trace), which were assigned to the protonated *E*-[**1b**-H]$^+$ and *Z*-[**1b**-H]$^+$ isomers, respectively. After neutralization with NEt_3, the absorption spectrum of the neutral bathochromic *Z*-isomer of diaryl-HI **1b** is obtained (light blue curve). No acid-induced thermal equilibration leading to significant population of the *E* isomers is observed, which hints at a possible further stabilization of the thermodynamically stable *Z* isomer in acidic medium. Spectroscopically distinct protonated species are formed at high acid concentrations. Source data are provided as Source Data File.

a

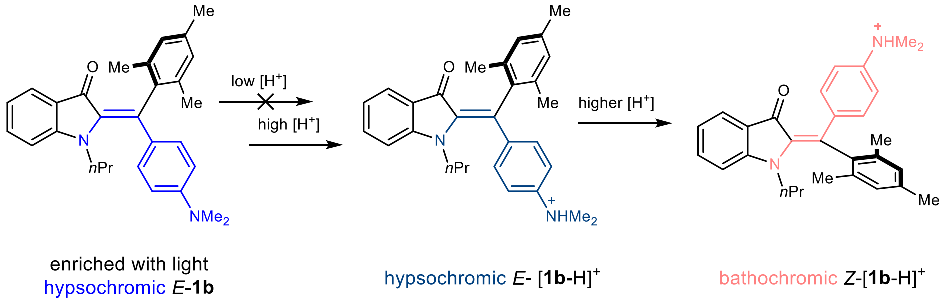

b

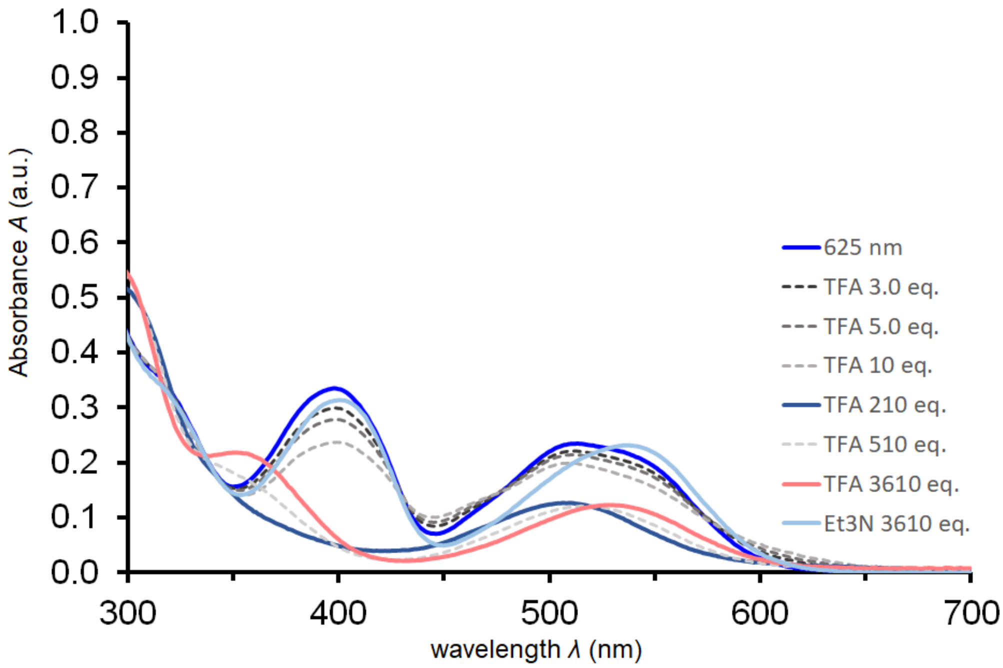

Supplementary Figure 108: a Schematic illustration of the acid/based induced isomerizations of diaryl-HI **1b**. Starting from the hypsochromic *E* isomer the protonated hypsochromic *E*-[**1b**-H]$^+$ isomer and protonated bathochromic *Z*-[**1b**-H]$^+$ isomer can be obtained (thermal equilibrium $E = 42\%$, $Z = 58\%$; hypsochromic pss: $E = 62\%$; bathochromic pss: $Z = 100\%$). **b** Absorption spectra of diaryl-HI **1b** in toluene solution at 23 °C recorded after irradiation with 625 nm light (hypsochromic *E*-**1b** enriched, blue curve). Subsequent addition of up to 10 equiv. TFA does not lead to formation of appreciable amounts of the bathochromic isomer. After addition of 110 to 2,610 equiv. TFA two new protonated species are formed, first the third species with hypsochromic shifted absorbance (cyano trace) and then the fourth species with slightly bathochromic shift (salmon red trace), which were assigned to the protonated *E*-[**1b**-H]$^+$ and *Z*-[**1b**-H]$^+$ isomers, respectively. After neutralization with NEt_3, the absorption spectrum of the neutral bathochromic *Z*-isomer of diaryl-HI **1b** is obtained (light blue curve). Thus, adding a small amount of acid does not lead to isomerization whereas adding a large amount of acid induces facile thermal *E* to *Z* isomerization of **1b**. Spectroscopically distinct protonated species are formed at high acid concentrations. Source data are provided as Source Data File.

a

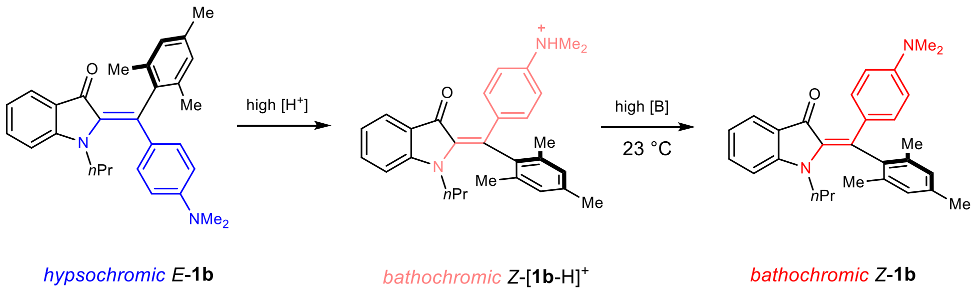

b

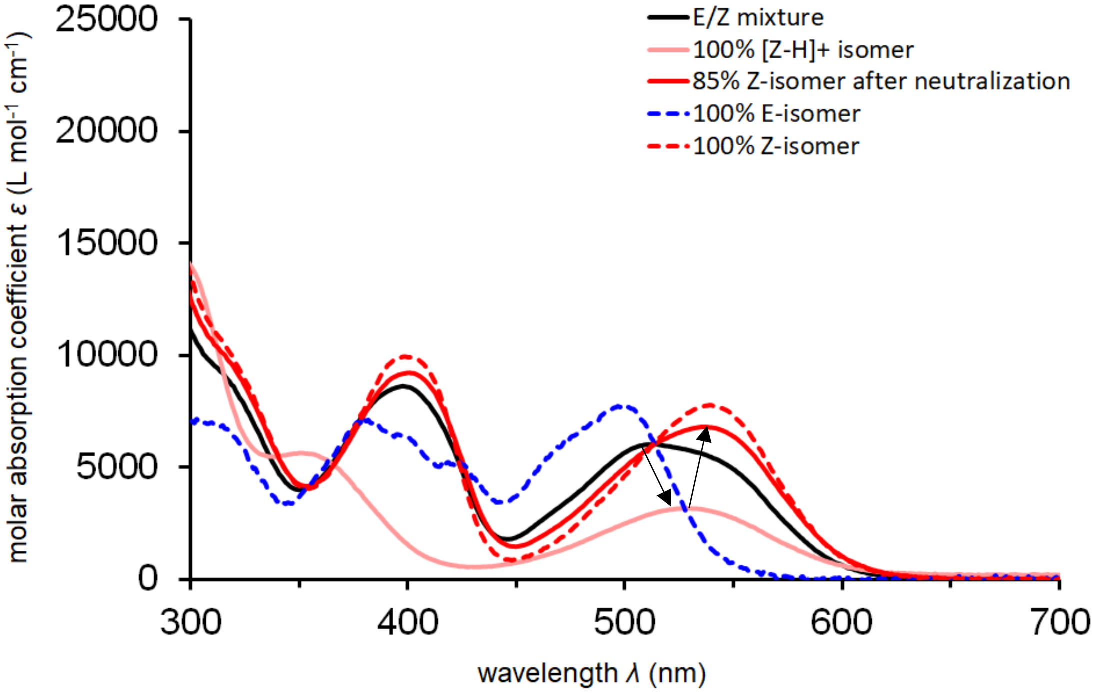

Supplementary Figure 109: Acid/base induced enrichment of thermodynamically less stable *Z*-**1b** isomer. Enrichment of *Z*-**1b** is possible after addition of TFA to a *E*/*Z*-**1b** mixture and subsequent neutralization with triethylamine. **a** Schematic illustration of acid and base induced isomerization of **1b**. **b** To a *E*/*Z*-**1b** mixture in toluene (black curve), concentrated TFA (about 5,000 equiv., salmon curve) was added at 23 °C. The solution containing newly formed *Z*-[**1b**-H]$^+$ was then neutralized with triethylamine (about 5,000 equiv., red curve) at 23 °C to recover 85% of the *Z*-**1b** isomer. Due to the high thermal bistability of the compound cooling is not necessary during the neutralization step. Spectra were scaled to the known isosbestic points of the previously determined molar absorption coefficients of the pure *E* and *Z* isomers (100% *E*-isomer, 100% *Z*-isomer, dashed blue and red curves). Previous acid/base experiments show, that the isosbestic points do not change their position on the x-axis after acid/base treatment but change their position on the y-axis more or less pronounced correlated to dilution effects. The individual isomer content after neutralization with base was determined according to Supplementary Note 4 via Equation (3) or Equation (4) with the constraints that $x_{E+}^{E} + x_{E+}^{Z} = 1$ or $x_{Z+}^{E} + x_{Z+}^{Z} = 1$. Source data are provided as Source Data File.

a

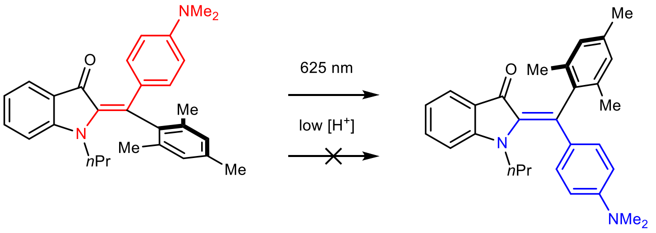

enriched with light
bathochromic *Z*-**1b**

hypsochromic *E*-**1b**

b

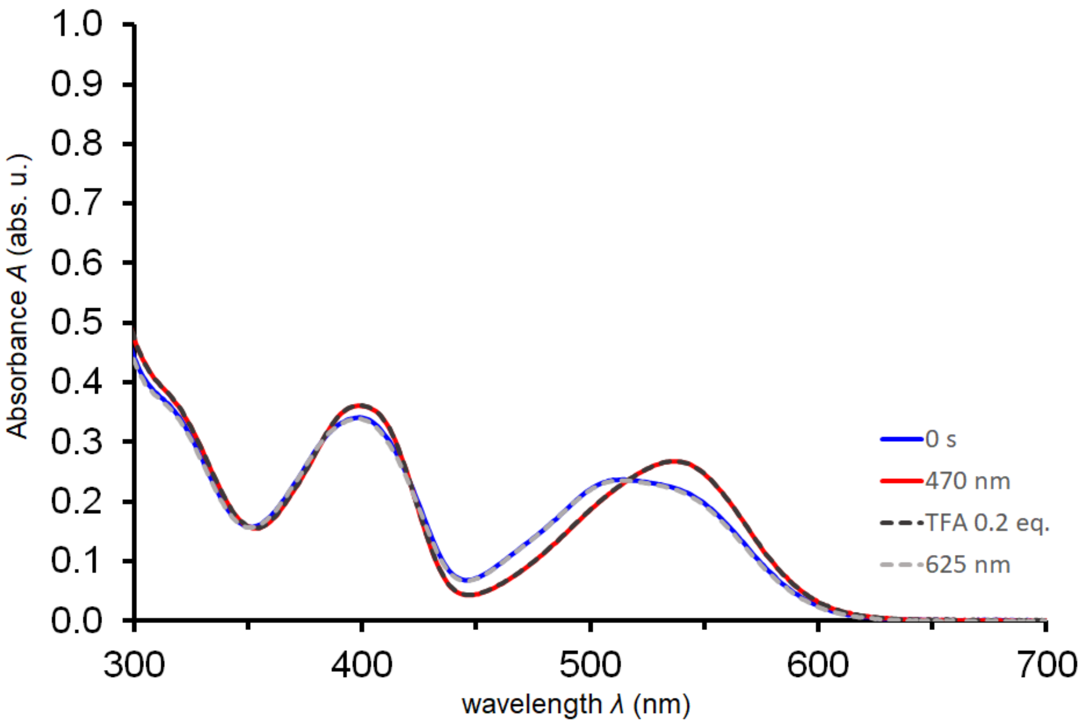

Supplementary Figure 110: a Schematic illustration of the light induced isomerization from the bathochromic to the hypsochromic isomer of diaryl-HI **1b** in the presence of small amounts of acid (thermal equilibrium E = 42%, Z = 58%; hypsochromic pss: E = 62%; bathochromic pss: Z = 100%). **b** Absorption spectra of diaryl-HI **1b** in toluene solution at 23 °C recorded after irradiation with 625 nm light (hypsochromic *E*-**1b** enriched, blue curve) and after irradiation with 470 nm light (bathochromic *Z*-**1b** enriched, red curve). Subsequent addition of 0.2 equiv. TFA to the bathochromic enriched *Z*-**1b** species does not interfere with the response to photoisomerization with light of 625 nm from the bathochromic *Z*-**1b** to the hypsochromic *E*-**1b**. Source data are provided as Source Data File.

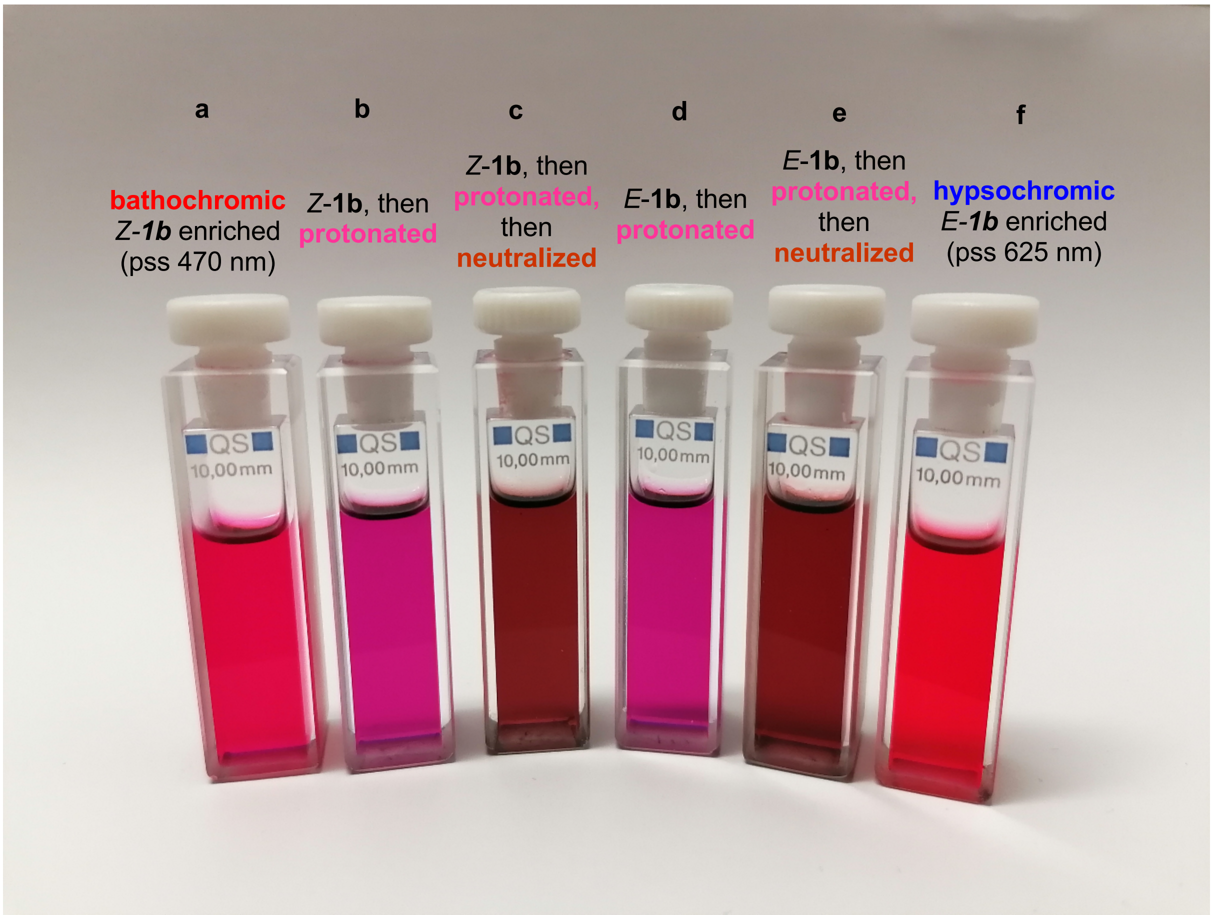

Supplementary Figure 111: Photograph of six diaryl-HI **1b** solutions of same concentrations in toluene at 23 °C. **a** Diaryl-HI **1b** solution was irradiated with 470 nm light until reaching the pss to enrich the bathochromic *Z*-**1b** isomer. **b** *Z*-**1b** solution mixed with excess of TFA. **c** *Z*-**1b** solution mixed with excess of TFA followed by neutralization with NEt_3. **d** *E*-**1b** enriched solution mixed with excess of TFA. **e** *E*-**1b** enriched solution mixed with excess of TFA followed by neutralization with NEt_3. **f** Diaryl-HI **1b** solution irradiated with 625 nm light until reaching the pss to enrich the hypsochromic *E*-**1b** isomer. Source data are provided as Source Data File.

8.3 Acid induced isomerization of diaryl-HI 1c

a

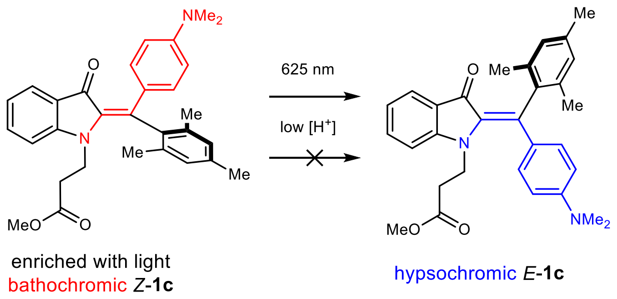

b

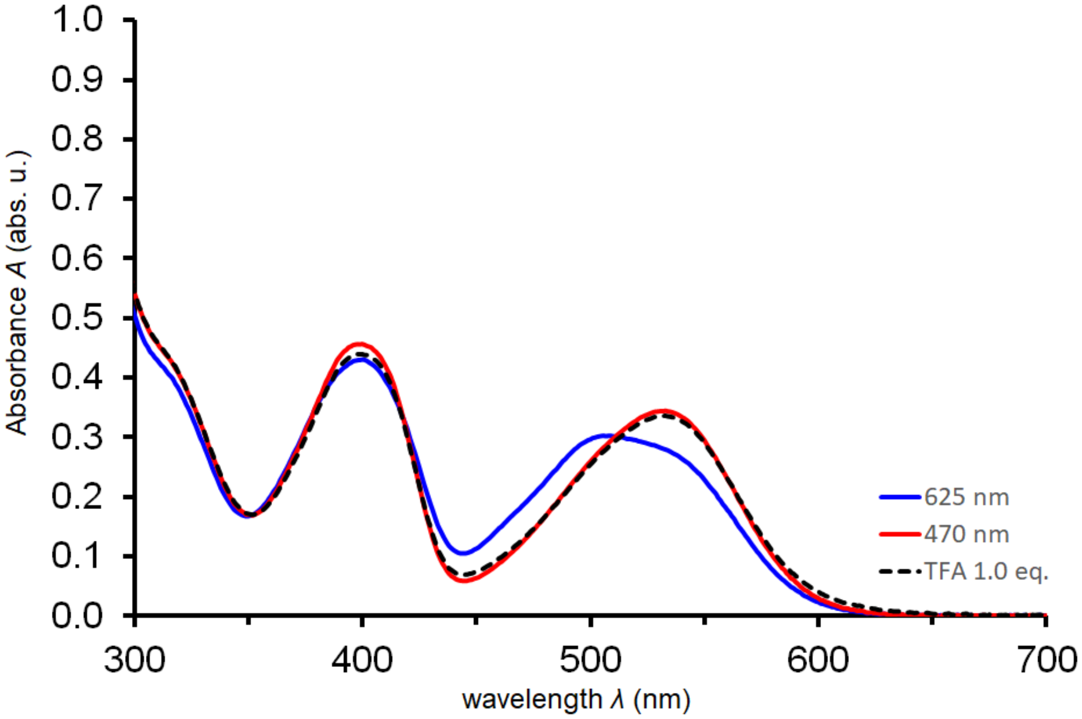

Supplementary Figure 112: a Schematic illustration of retained thermal bistability of diaryl-HI **1c** in the presence of small amounts of acid (thermal equilibrium $E = 34\%$, $Z = 66\%$; hypsochromic pss: $E = 63\%$; bathochromic pss: $Z = 100\%$). **b** Absorption spectra of diaryl-HI **1c** in toluene solution at 23 °C recorded after irradiation with 625 nm light (hypsochromic *E*-**1c** enriched, blue curve) and after irradiation with 470 nm light (bathochromic *Z*-**1c** enriched, red curve). Subsequent addition of 0.1 to 1.0 equiv. TFA (black dotted curve) to the bathochromic enriched *Z*-**1b** species does not induce isomerization to the hypsochromic isomer at least for 10 min. Source data are provided as Source Data File.

8.4 Acid induced isomerization of diaryl-HI 2b

a

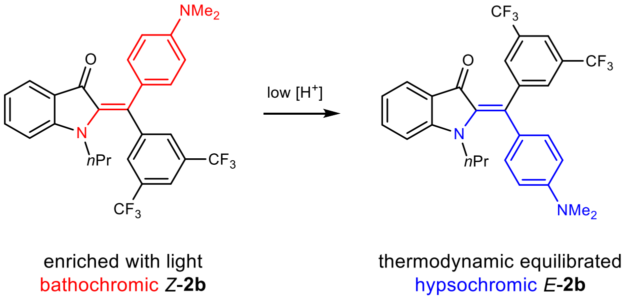

b

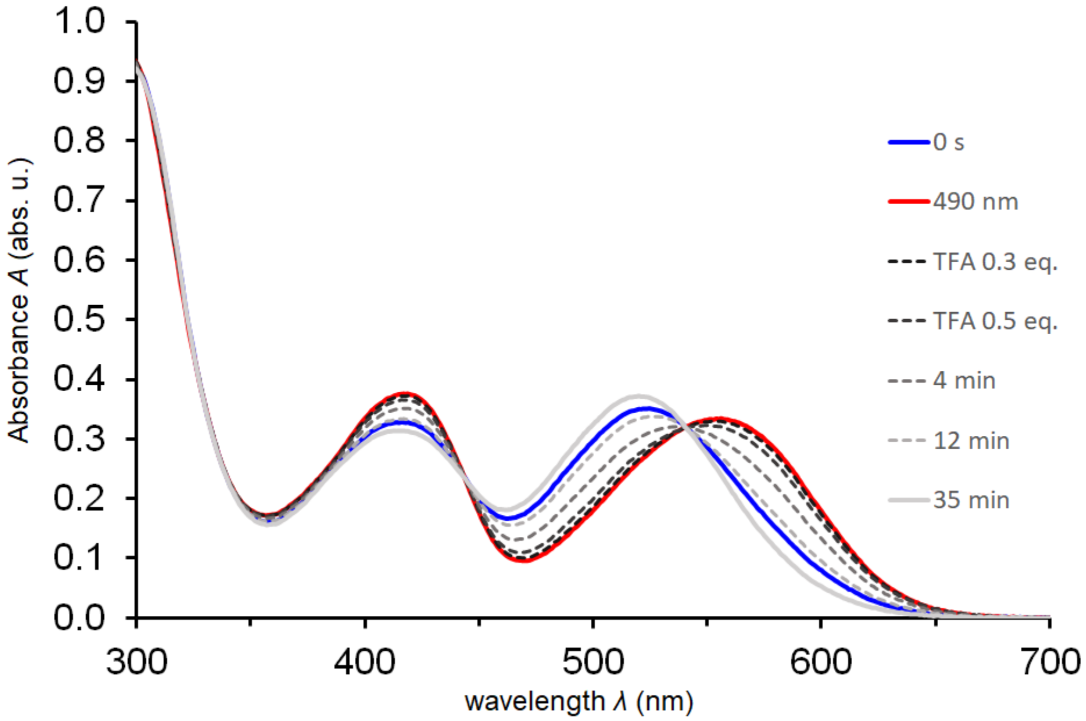

Supplementary Figure 113: a Schematic illustration of the acid induced isomerization from the bathochromic to the hypsochromic isomer of diaryl-HI **2b** (thermal equilibrium *E* = 67%, *Z* = 33%; hypsochromic pss: *E* = 79%; bathochromic pss: *Z* = 92%). **b** Absorption spectra of diaryl-HI **2b** in toluene solution at 23 °C recorded after irradiation with 625 nm light (thermal equilibrated *E*-**2b**, blue curve) and after irradiation with 490 nm light (bathochromic *Z*-**2b** enriched, red curve). Subsequent addition of 0.1 to 0.6 equiv. TFA (black to light grey dotted curves) leads to formation of the hypsochromic isomer *E*-**2b** within 35 min (light grey curve). Acid induced thermal isomerization is accelerated compared to thermal isomerization of neutral **2b**. Source data are provided as Source Data File.

a

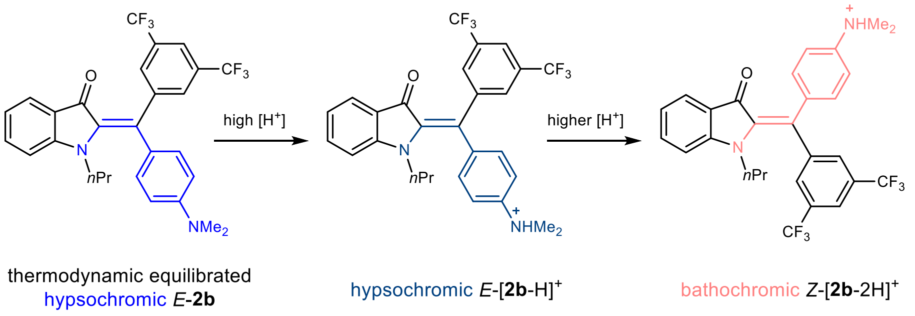

b

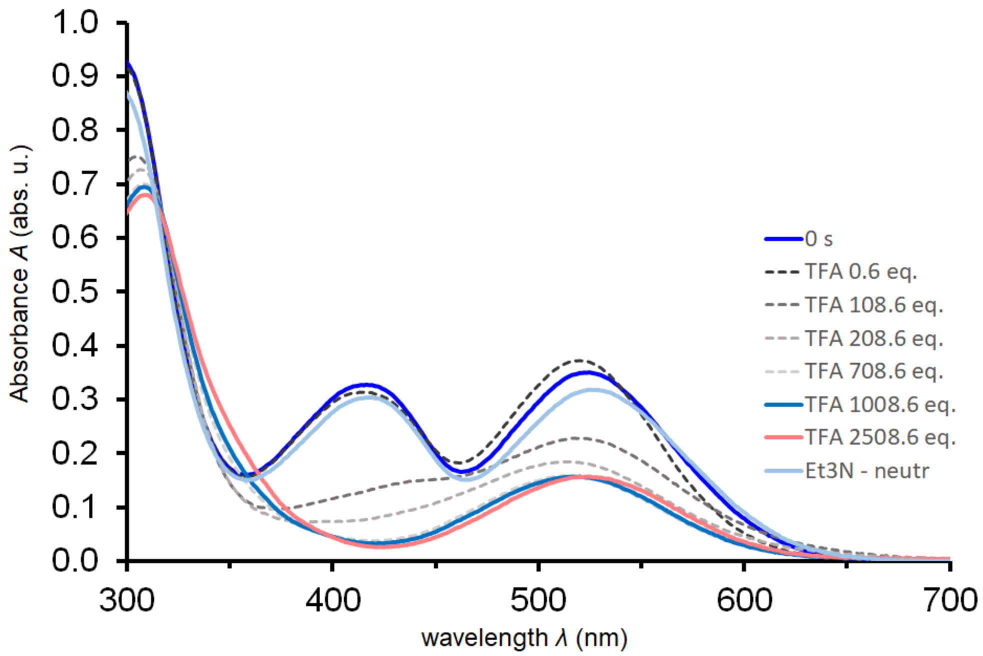

Supplementary Figure 114: a Schematic illustration of the acid/based induced isomerizations of diaryl-HI **2b**. Starting from the bathochromic *Z*-isomer the protonated hypsochromic *E*-[**2b**-H]$^+$ isomer and protonated bathochromic *Z*-[**2b**-H]$^+$ isomer can be obtained (thermal equilibrium *E* = 67%, *Z* = 33%; hypsochromic pss: *E* = 79%; bathochromic pss: *Z* = 92%). **b** Absorption spectra of diaryl-HI **2b** in toluene solution at 23 °C recorded after irradiation with 490 nm light (bathochromic *Z*-**2b** enriched, red curve). Subsequent addition of up to 8.6 equiv. TFA leads to formation of the hypsochromic isomer. After addition of 109 to 1,009 equiv. TFA two new protonated species are formed, first the third species with hypsochromic shifted absorbance (cyano trace) and then the fourth species with slightly bathochromic shift (salmon red trace), which were assigned to the protonated *E*-[**2b**-H]$^+$ and *Z*-[**2b**-H]$^+$ isomers, respectively. After neutralization with NEt_3 at 23 °C, the absorption spectrum dominated by the neutral hypsochromic *E*-isomer of diaryl-HI **2b** is obtained (light blue curve). Thus, adding acid induces facile thermal *Z* to *E* isomerization of **2b** and spectroscopically distinct protonated species are formed at high acid concentrations (*E*-[**2b**-H]$^+$ and *Z*-[**2b**-H]$^+$). The relative isomer ratios in thermal equilibrium are opposite for the neutral and protonated states and therefore the thermodynamic less stable neutral Z-**2b** isomer can only be enriched after neutralization if the sample is cooled. Source Data are provided as Source Data File.

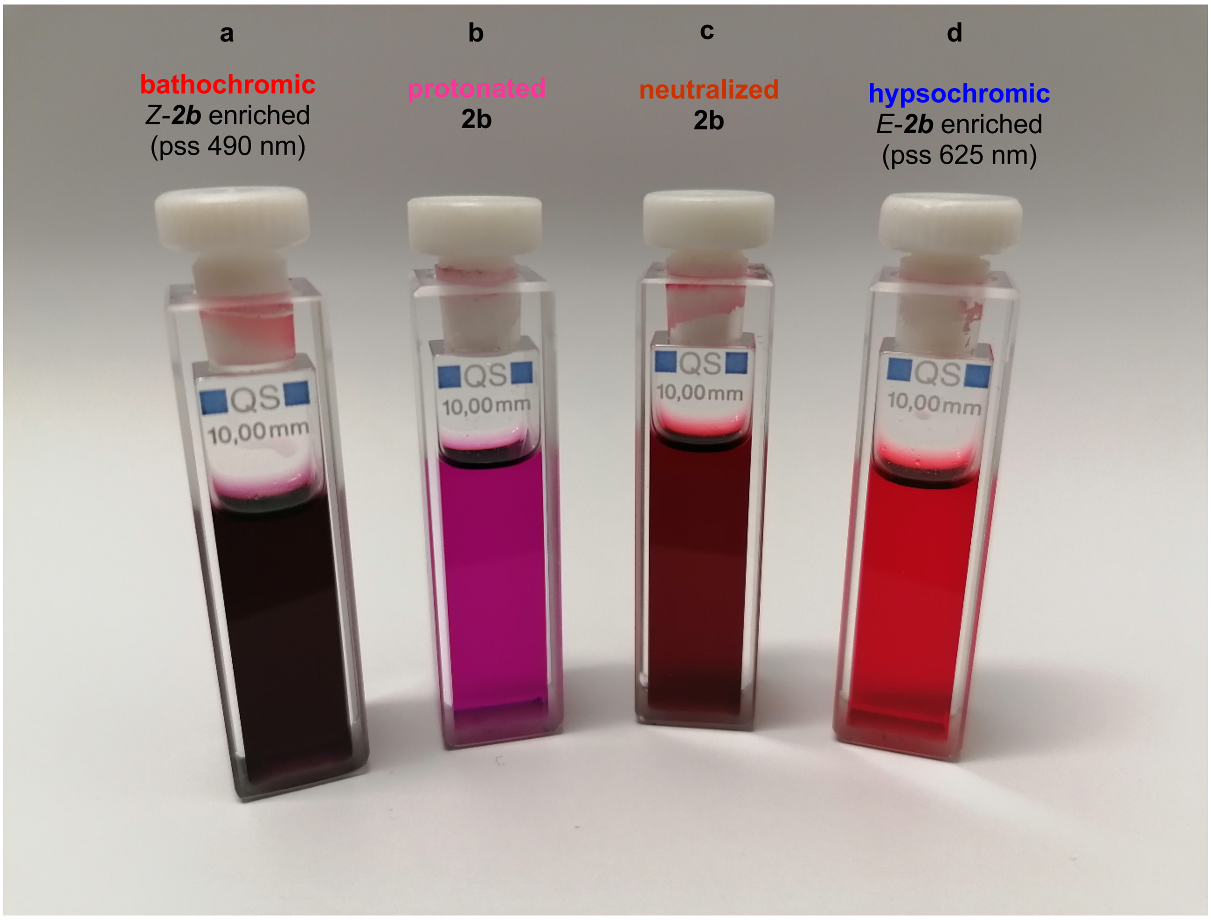

Supplementary Figure 115: Photograph of four diaryl-HI **2b** solutions of same concentrations in toluene at 23 °C. **a** Diaryl-HI **2b** solution was irradiated with 490 nm light until reaching the pss to enrich the bathochromic *Z*-**2b** isomer. **b** *Z*-**2b** solution mixed with excess of TFA. **c** *Z*-**1b** solution mixed with excess of TFA followed by neutralization with NEt_3. **d** Diaryl-HI **2b** solution irradiated with 625 nm light until reaching the pss to enrich the hypsochromic *E*-**2b** isomer. Source data are provided as Source Data File.

8.5 Acid induced isomerization of diaryl-HI 2c

a

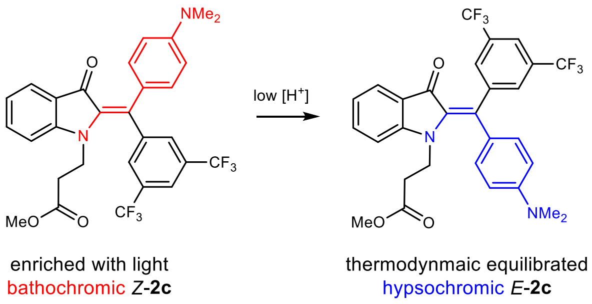

b

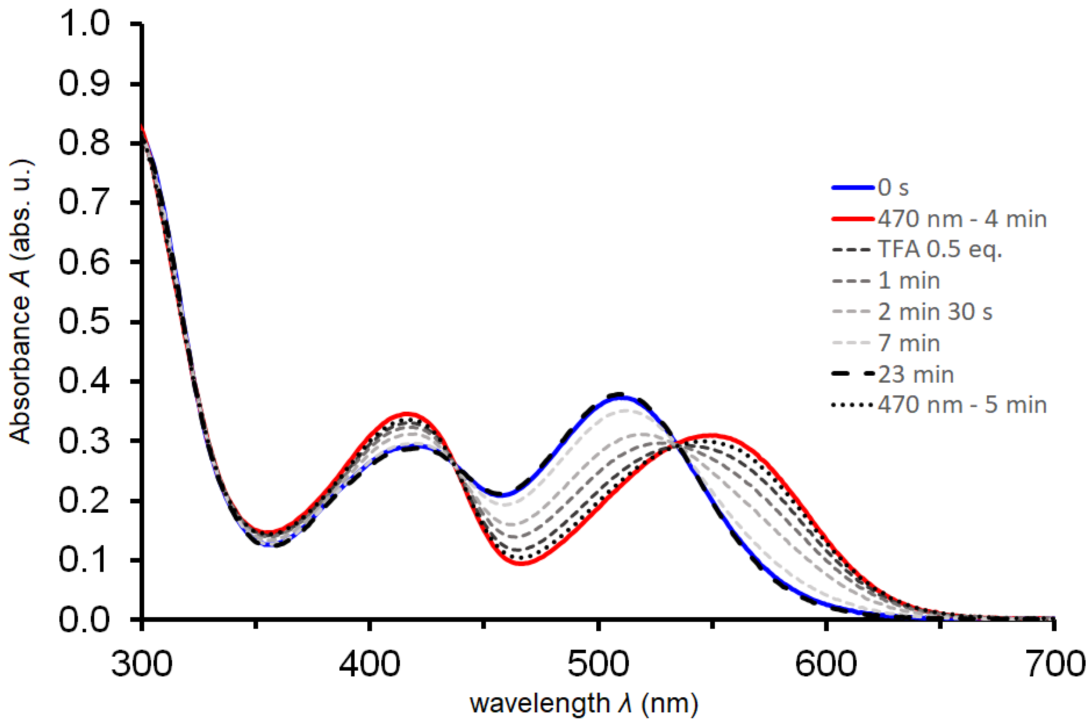

Supplementary Figure 116: a Schematic illustration of the acid induced isomerization from the bathochromic to the hypsochromic isomer of diaryl-HI **2c** (thermal equilibrium *E* = 87%, *Z* = 13%; hypsochromic pss: *E* = 85%; bathochromic pss: *Z* = 92%). **b** Absorption spectra of diaryl-HI **2c** in toluene solution at 23 °C recorded after irradiation with 625 nm light (hypsochromic *E*-**2b** enriched, blue curve) and after irradiation with 470 nm light (bathochromic *Z*-**2c** enriched, red curve). Subsequent addition of 0.5 equiv. TFA leads to formation of the hypsochromic isomer *E*-**2c** within 26 min. Acid induced thermal isomerization is accelerated compared to thermal isomerization of neutral **2c**. Source data are provided as Source Data File.

a

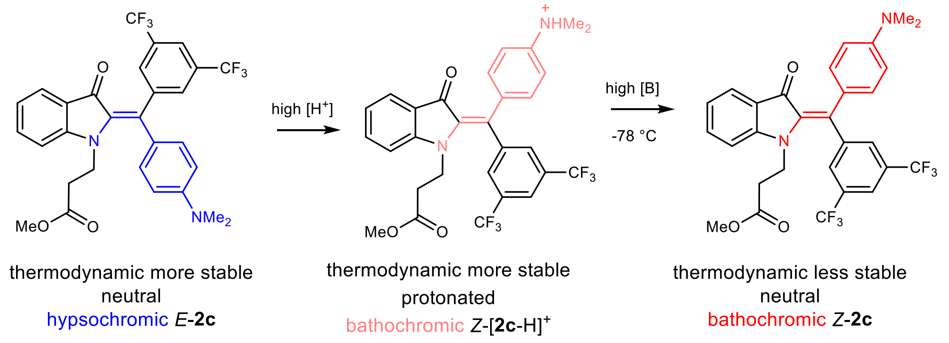

b

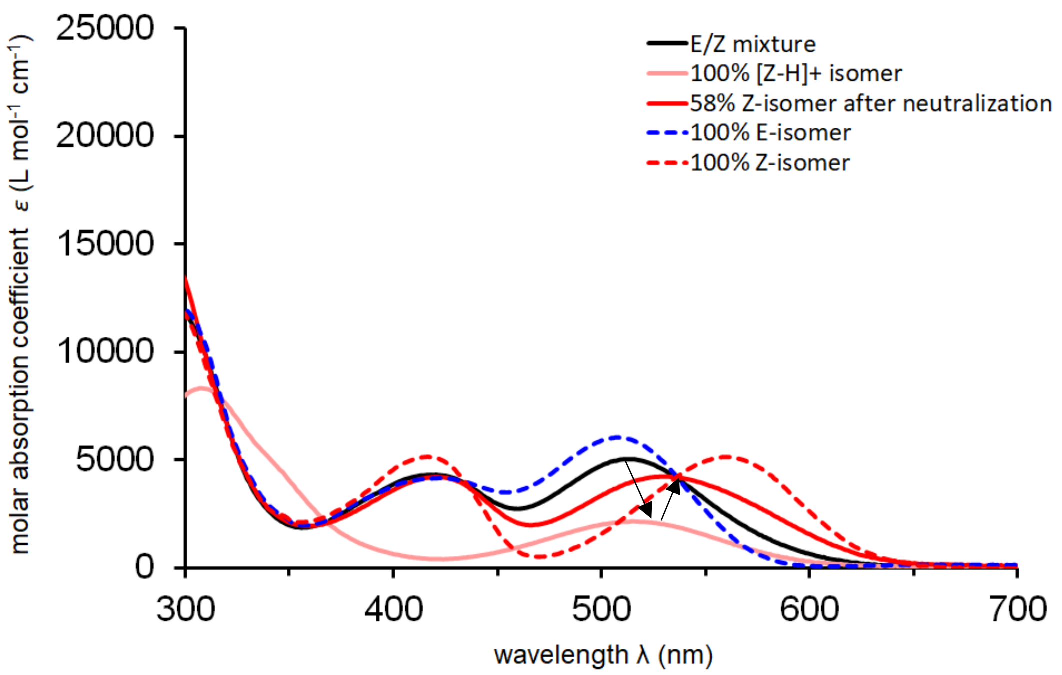

Supplementary Figure 117: Acid/base induced enrichment of thermodynamically less stable *Z*-**2c** isomer. Enrichment of *Z*-**2c** is possible after addition of a large excess of TFA to a *E*/*Z*-**2c** mixture and subsequent neutralization with triethylamine. **a** Schematic illustration of acid and base induced isomerization of **2c**. **b** To a *E*/*Z*-**2c** mixture (black curve) in toluene, concentrated TFA (5,000 equiv., salmon curve) was added at 23 °C. The solution containing newly formed *Z*-[**2c**-H]$^+$ was then cooled to –78 °C and triethylamine (10,000 equiv., red curve) was added. The solution was allowed to reach 23 °C under fast shaking to enrich the *Z*-**2c** isomer (58%). Spectra were scaled to the known isosbestic points of the previously determined molar absorption coefficients of the pure *E* and *Z* isomers (100% *E*-isomer, 100% *Z*-isomer, dashed blue and red curve). Previous acid/base experiments show, that the isosbestic points do not change their position in the x-axis after acid/base treatment but change their position in the y-axis more or less pronounced correlated to dilution effects. The individual isomer content after neutralization with base was determined according to Supplementary Note 4 via Equation (3) or Equation (4) with the constraints that $x_{E+}^{E} + x_{E+}^{Z} = 1$ or $x_{Z+}^{E} + x_{Z+}^{Z} = 1$. Source data are provided as Source Data File.

8.6 Acid induced isomerization of diaryl-HI 3a

a

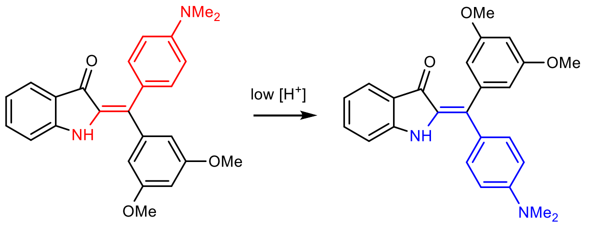

b

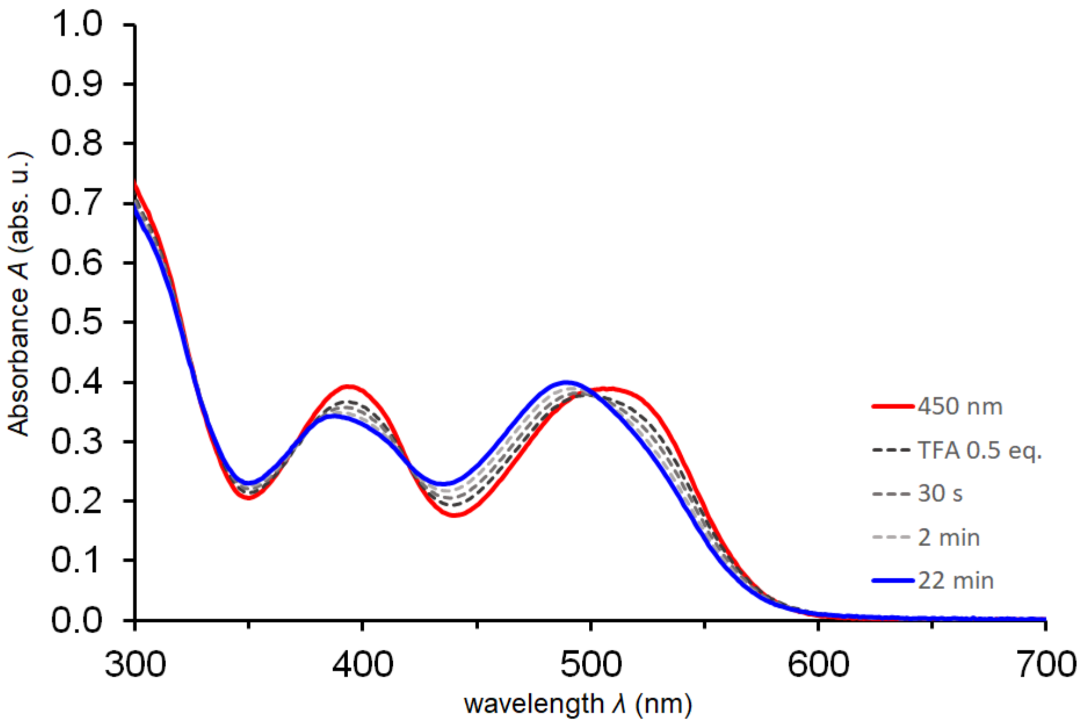

Supplementary Figure 118: a Schematic illustration of the acid induced isomerization from the bathochromic to the hypsochromic isomer of diaryl-HI **3a** (thermal equilibrium E = 47%, Z = 53%; hypsochromic pss: E = 50%; bathochromic pss: Z = 53%). **b** Absorption spectra of diaryl-HI **3a** in toluene solution at 23 °C recorded after irradiation with 450 nm light (bathochromic *Z*-**3a** enriched, red curve). Subsequent addition of 0.5 equiv. TFA leads to formation of the hypsochromic isomer *E*-**3a** within 22 min. Acid induced thermal isomerization is accelerated compared to thermal isomerization of neutral **3a**. Source data are provided as Source Data File.

8.7 Acid induced isomerization of diaryl-HI 3b

a

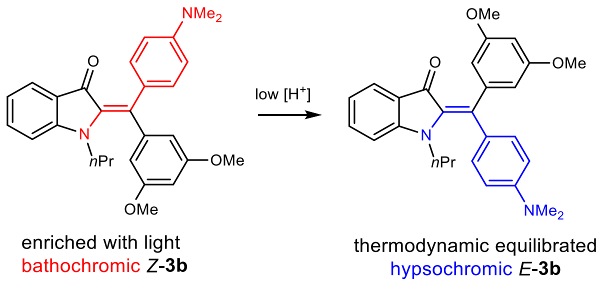

b

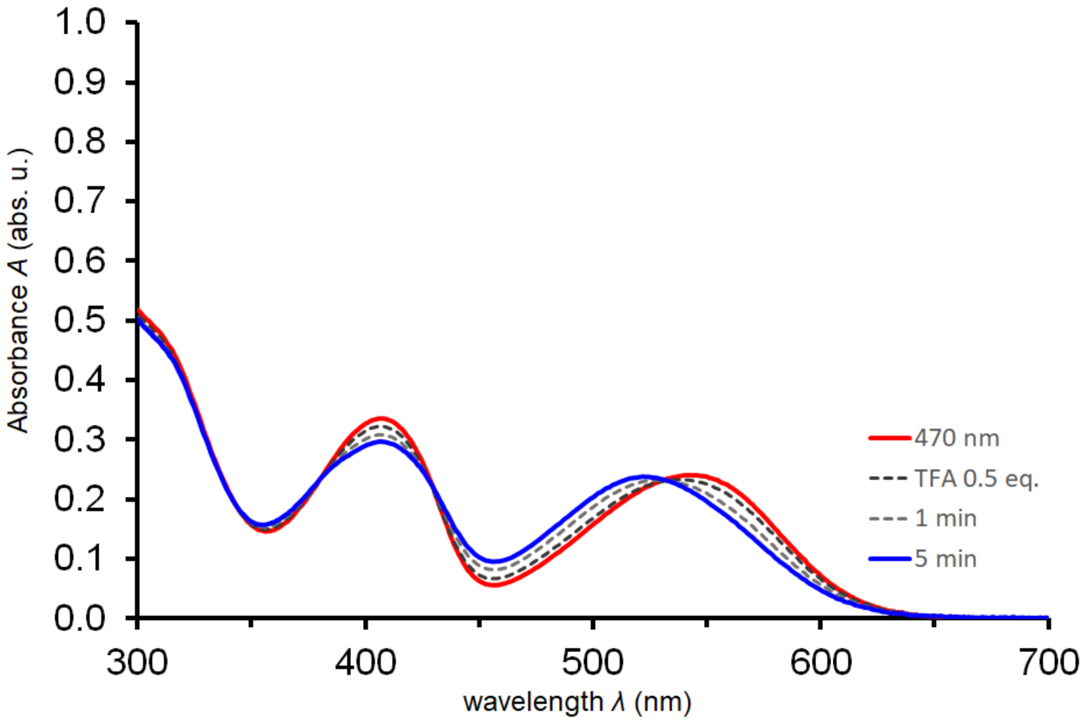

Supplementary Figure 119: a Schematic illustration of the acid induced isomerization from the bathochromic to the hypsochromic isomer of diaryl-HI **3b** (thermal equilibrium *E* = 46%, *Z* = 55%; hypsochromic pss: *E* = 62%; bathochromic pss: *Z* = 92%). **b** Absorption spectra of diaryl-HI **3b** in toluene solution at 23 °C recorded after irradiation with 470 nm light (bathochromic *Z*-**3b** enriched, red curve). Subsequent addition of 0.5 equiv. TFA leads to formation of the hypsochromic isomer *E*-**3b** within 13 min. Acid induced thermal isomerization is accelerated compared to thermal isomerization of neutral **3b**. Source data are provided as Source Data File.

a

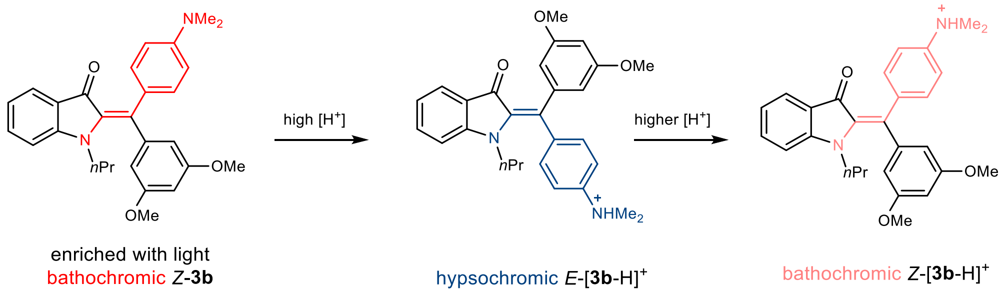

b

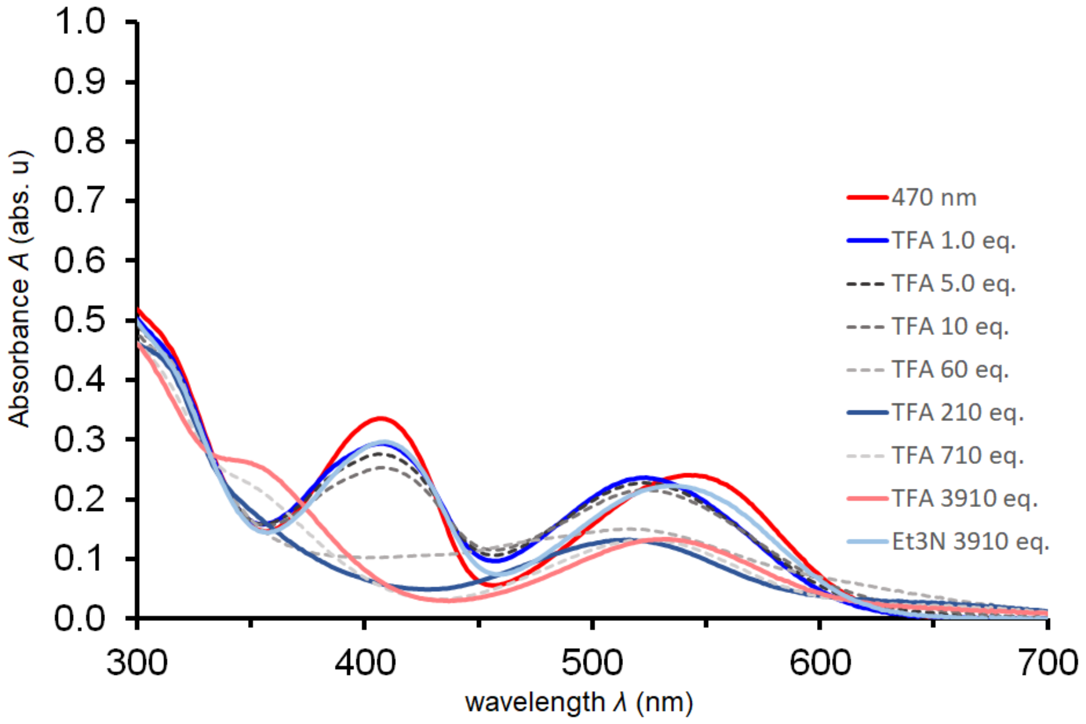

Supplementary Figure 120: a Schematic illustration of the acid/based induced isomerizations of diaryl-HI **3b**. Starting from the bathochromic *Z*-isomer the protonated hypsochromic *E*-[**3b**-H]$^+$ isomer and protonated bathochromic *Z*-[**3b**-H]$^+$ isomer can be obtained (thermal equilibrium *E* = 46%, *Z* = 55%; hypsochromic pss: *E* = 62%; bathochromic pss: *Z* = 92%). **b** Absorption spectra of diaryl-HI **3b** in toluene solution at 23 °C recorded after irradiation with 470 nm light (bathochromic *Z*-**3b** enriched, blue curve) and with 625 nm light (hypsochromic *E*-**3b** enriched, blue curve). Subsequent addition of up to 10 equiv. TFA does not lead to formation of appreciable amounts of the bathochromic isomer. After addition of 60 to 3,910 equiv. TFA two new protonated species are formed, first the third species with hypsochromic shifted absorbance (cyano trace) and then the fourth species with slightly bathochromic shift (salmon red trace), which were assigned to the protonated *E*-[**3b**-H]$^+$ and *Z*-[**3b**-H]$^+$ isomers of **3b**, respectively. After neutralization with NEt_3, the absorption spectrum of the neutral thermodynamically more stable bathochromic *Z*-isomer of diaryl-HI **3b** is obtained (light blue curve). Thus, adding a small amount of acid leads to isomerization from *Z* to *E* isomer, whereas adding a large amount of acid induces enrichment of the bathochromic *Z*-[**3b**-H]$^+$ species. Both protonated species *E*-[**3b**-H]$^+$ and *Z*-[**3b**-H]$^+$ are spectroscopically distinct. Source data are provided as Source Data File.

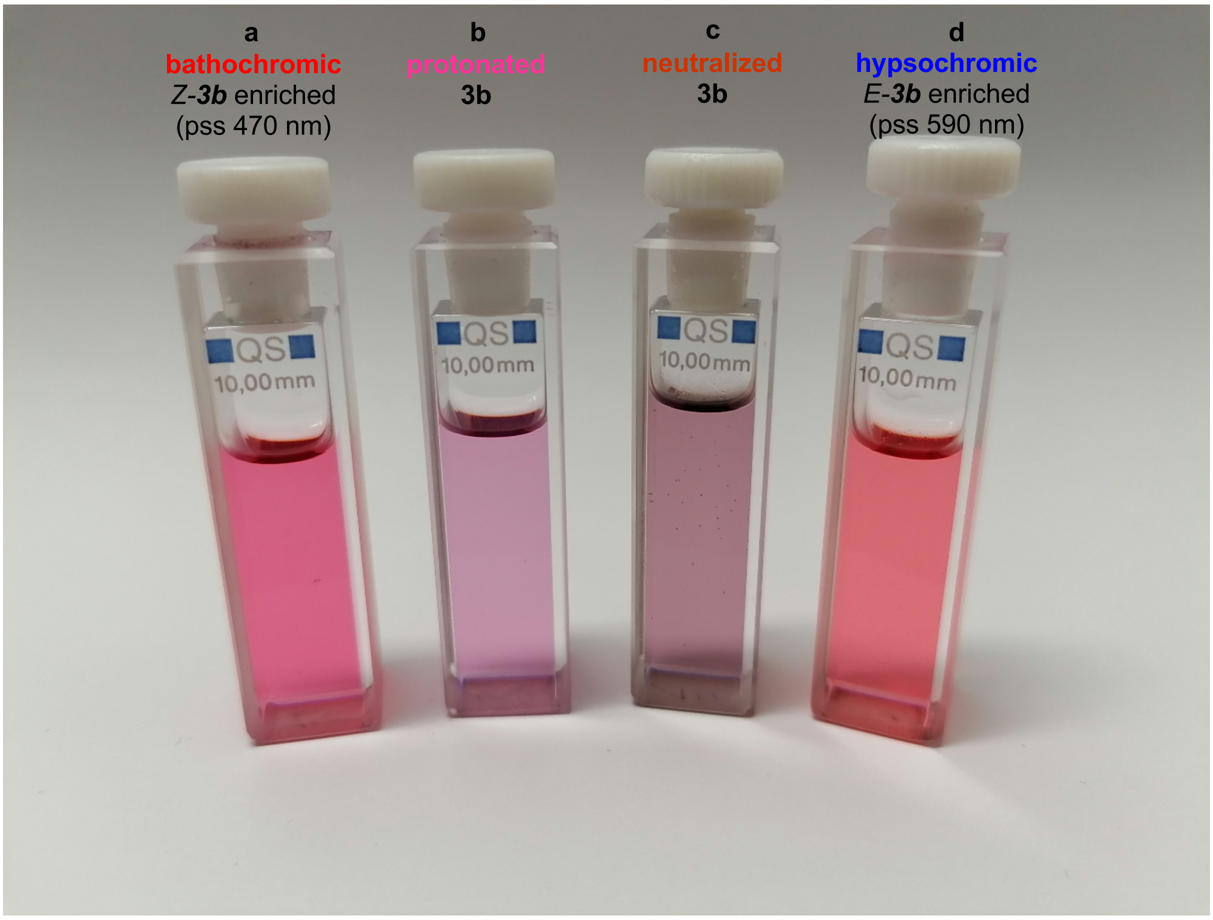

Supplementary Figure 121: Photograph of four diaryl-HI **3b** solutions of same concentrations in toluene at 23 °C. **a** Diaryl-HI **3b** solution was irradiated with 470 nm light until reaching the pss to enrich the bathochromic *Z*-**3b** isomer. **b** *Z*-**3b** solution mixed with excess of TFA. **c** *Z*-**3b** solution mixed with excess of TFA followed by neutralization with NEt_3. **d** Diaryl-HI **3b** solution irradiated with 590 nm light until reaching the pss to enrich the hypsochromic *E*-**3b** isomer. Source data are provided as Source Data File.

8.8 Acid induced isomerization of diaryl-HI 3c

a

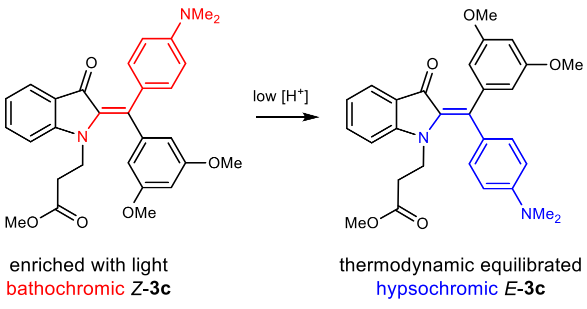

b

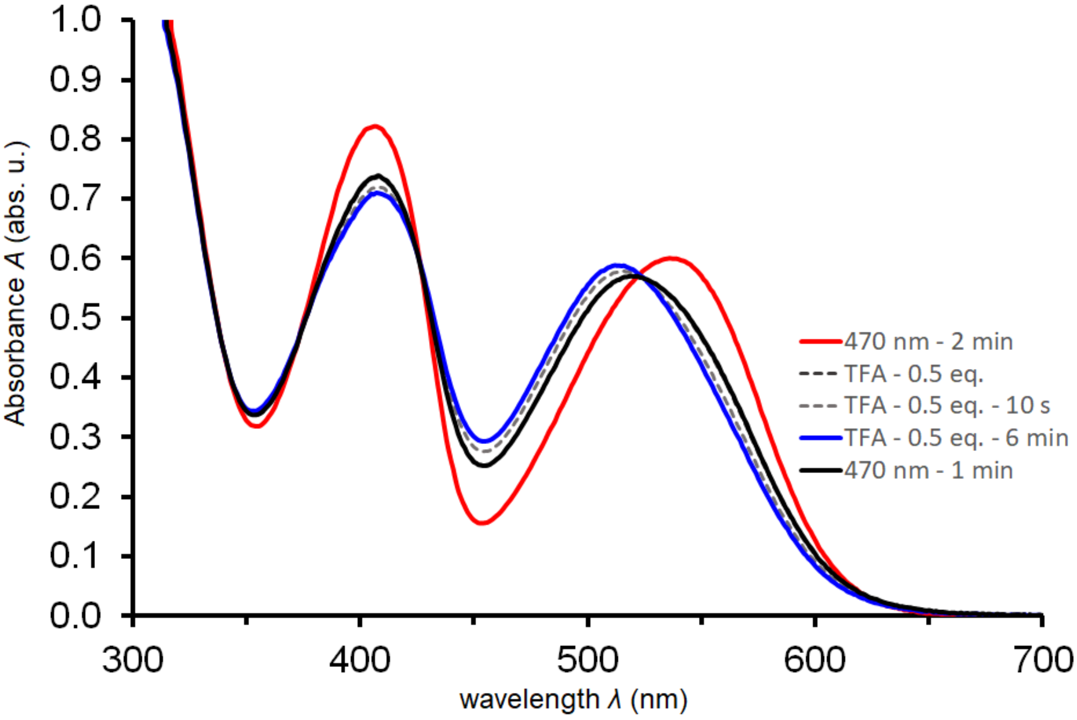

Supplementary Figure 122: a Schematic illustration of the acid induced isomerization from the bathochromic to the hypsochromic isomer of diaryl-HI **3c** (thermal equilibrium *E* = 48%, *Z* = 52%; hypsochromic pss: *E* = 46%; bathochromic pss: *Z* = 72%). **b** Absorption spectra of diaryl-HI **3c** in toluene solution at 23 °C recorded after irradiation with 470 nm light (bathochromic *Z*-**3c** enriched, red curve). Subsequent addition of 0.5 equiv. TFA (grey dotted curves) leads to formation of the hypsochromic isomer *E*-**3c** within 6 min (blue curve). Acid induced thermal isomerization is accelerated compared to thermal isomerization of neutral **3c**, addressability with light is still possible (black curve). Source data are provided as Source Data File.

8.9 Acid induced isomerization of diaryl-HI 4b

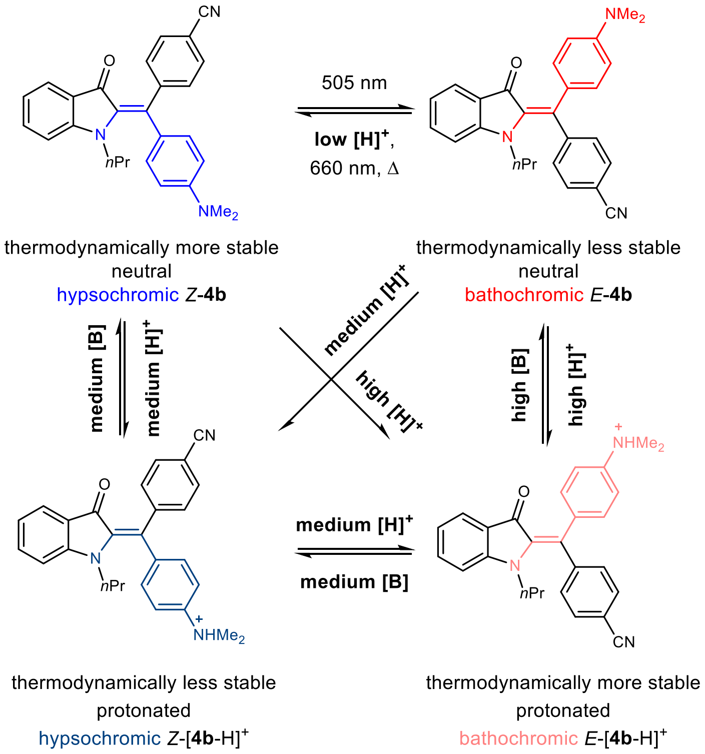

Supplementary Figure 123: Schematic illustration of the acid/base induced isomerization processes of diaryl-HI **4b** (thermal equilibrium $E = 23\%$, $Z = 77\%$; hypsochromic pss: $Z = 81\%$; bathochromic pss: $E = 89\%$; high acid concentration E-[**4b**-H]$^+$ = 91%; low acid concentration Z-[**4b**-H]$^+$ = 77%; data were measured in toluene). Source data are provided as Source Data File.

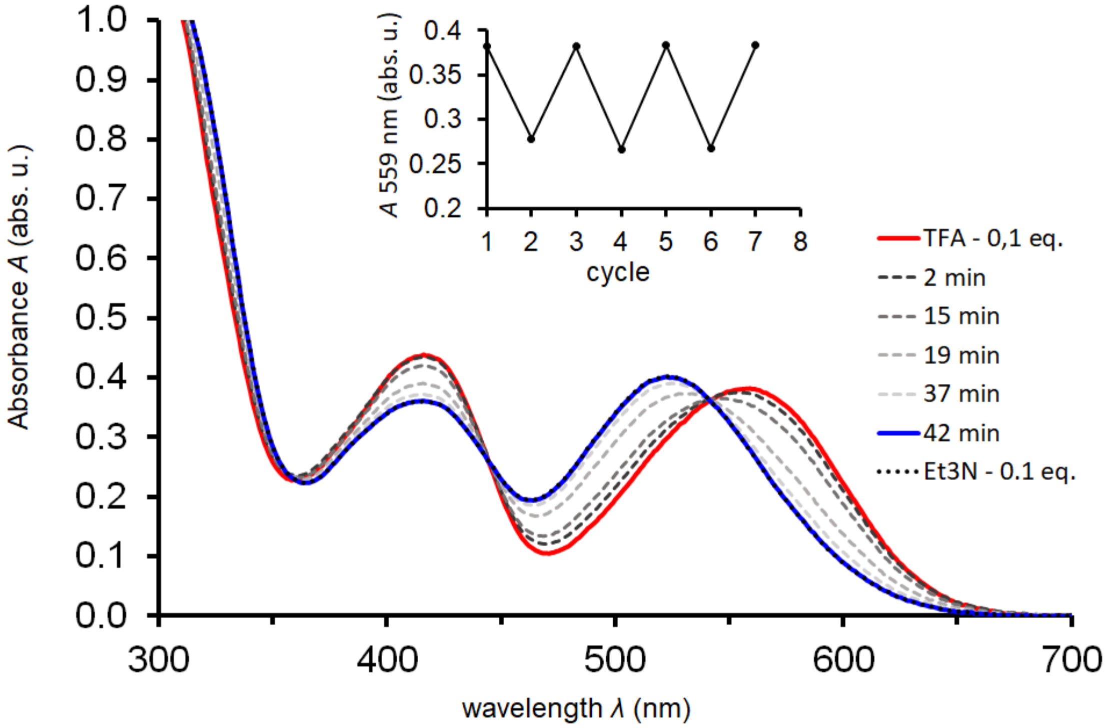

Supplementary Figure 124: Absorption spectra of diaryl-HI **4b** in toluene solution at 23 °C recorded after irradiation with 505 nm light (bathochromic *E*-**4b** enriched, red curve). Subsequent addition of 0.1 equiv. TFA (dark to light grey dotted curves) leads to formation of the hypsochromi*c* isomer *Z*-**4b** (blue curve) within 42 min. Acid induced thermal isomerization is accelerated compared to thermal isomerization of neutral **4b**. After neutralizing with NEt_3 hypsochromic *Z*-**4b** (black dotted curve) was recovered. Photoswitching with 505 nm light again enriched the bathochromic *E*-**4b** isomer and this process of acid and light driven isomerization with intermittent neutralization was repeated for several cycles (inset). Source data are provided as Source Data File.

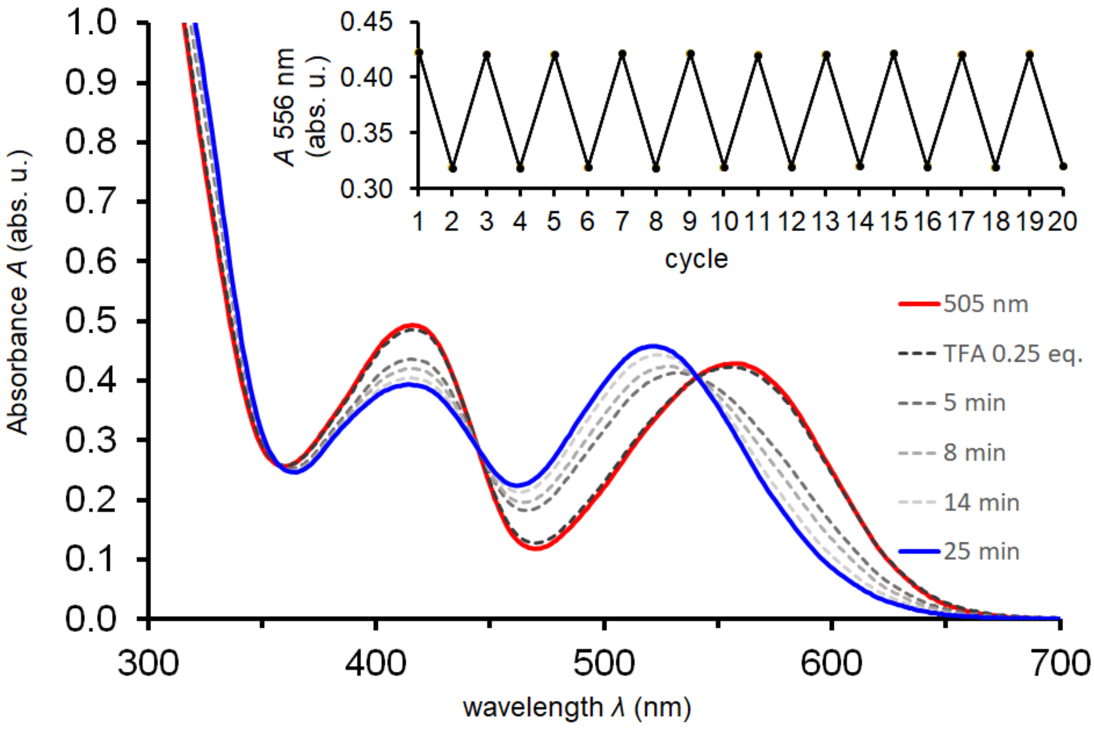

Supplementary Figure 125: Absorption spectra of diaryl-HI **4b** in toluene solution at 23 °C recorded after irradiation with 505 nm light (bathochromic *E*-**4b** enriched, red curve). Subsequent addition of 0.25 equiv. TFA (dark to light grey dotted curves) leads to formation of the hypsochromic isomer *Z*-**4b** (blue curve) within 25 min. Acid induced thermal isomerization is accelerated compared to thermal isomerization of neutral **4b**. Photoswitching with 505 nm light again enriched the bathochromic *E*-**4b** isomer in the presence of acid. The process of acid and light driven isomerization without neutralization was repeated for several cycles (inset). Source data are provided as Source Data File.

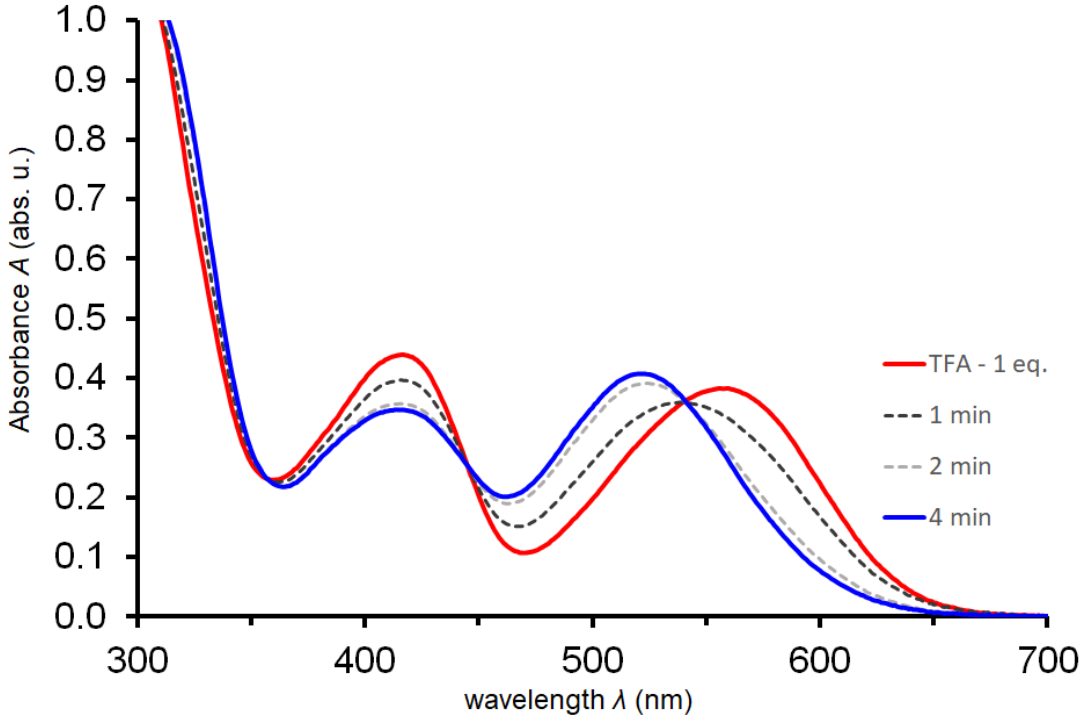

Supplementary Figure 126: Absorption spectra of diaryl-HI **4b** in toluene solution at 23 °C recorded after irradiation with 505 nm light (bathochromic *E*-**4b** enriched, red curve). Subsequent addition of 1.0 equiv. TFA dark to light grey dotted curves) leads to formation of the hypsochromic isomer *Z*-**4b** (blue curve) within 4 min. The acid induced isomerization to the hypsochromic *Z*-**4b** species is about ten times faster with 1.0 equiv. of TFA than with 0.1 equiv. of TFA (Supplementary Figure 124), which demonstrates the catalytic effect of the acid induces isomerization. Source data are provided as Source Data File.

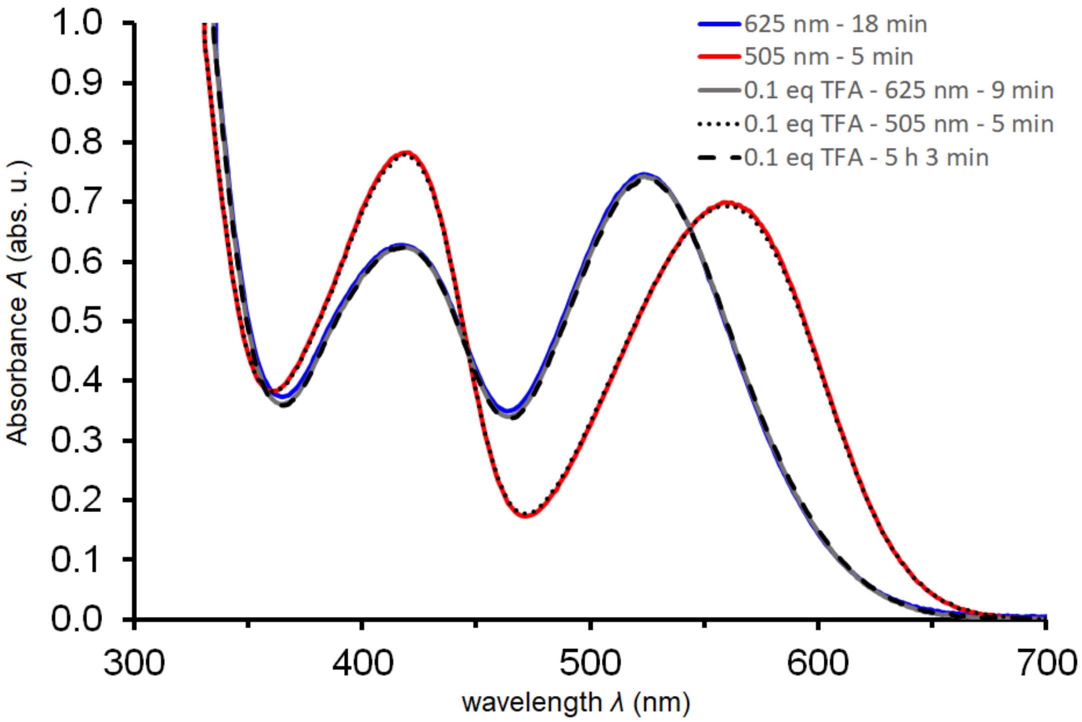

Supplementary Figure 127: Absorption spectra of diaryl-HI **4b** in toluene solution at 5 °C recorded after irradiation with 505 nm light (bathochromic *E*-**4b** enriched, red curve), 625 nm light (hypsochromic *Z*-**4b** enriched, blue curve), 505 nm / 625 nm light in presence of 0.1 equiv. of TFA, and after thermal isomerization from bathochromic to hypsochromic isomer in presence of 0.1 equiv. of TFA. The 505 nm light induced isomerization from hypsochromic *Z*-**4b** to bathochromic *E*-**4b** takes 5 min and the reverse process with light of 625 nm takes 18 min. The isomerization from hypsochromic to bathochromic isomer with 505 nm light in presence of 0.1 equiv. of TFA takes 5 min and the reverse process with light of 625 nm takes 9 min. The acid induced isomerization from bathochromic isomer to hypsochromic enriched isomer takes 5 h 3 min at 5 °C. Because of these large time differences, no thermal interference in the 625 nm light induced photoisomerizations is present. Source data are provided as Source Data File.

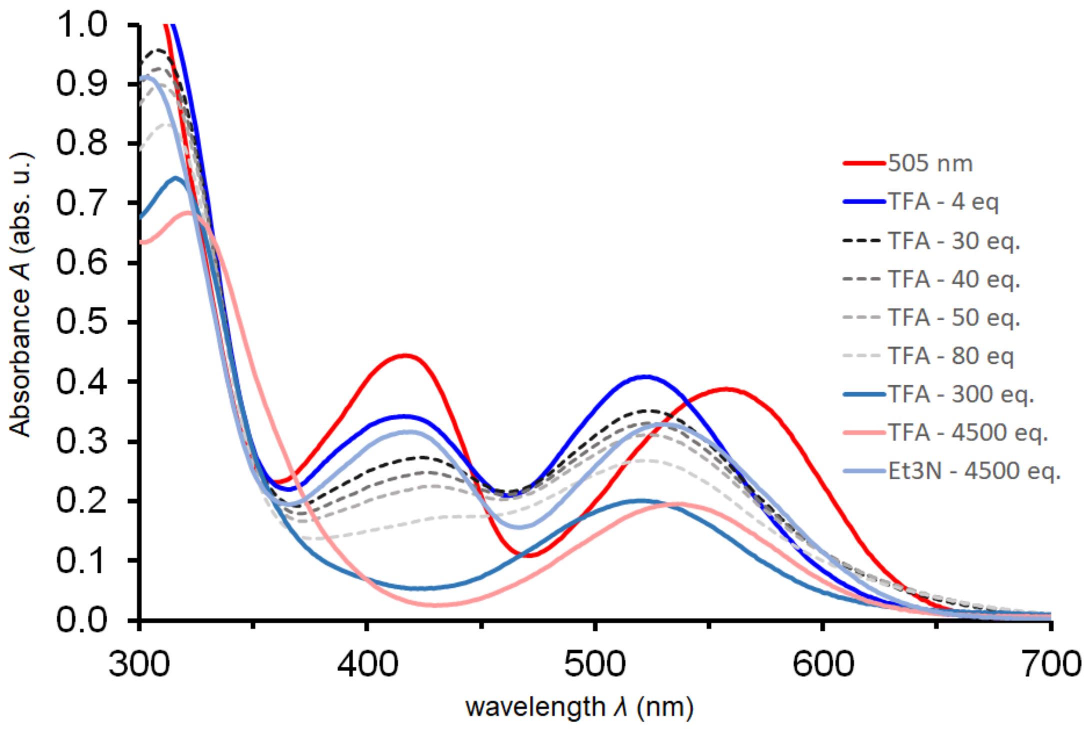

Supplementary Figure 128: Absorption spectra of diaryl-HI **4b** in toluene solution at 23 °C recorded after irradiation with 505 nm light (bathochromic *E*-**4b** enriched, red curve). Subsequent addition of up to 4.0 equiv. TFA leads to formation of the hypsochromic isomer *Z*-**4b**. After addition of 5.0 to 4,500 equiv. TFA two new protonated species are formed, first the third species with hypsochromic shifted absorbance (cyano trace) and then the fourth species with slightly bathochromic shift (salmon red trace), which were assigned to the protonated *Z*- and *E*-isomers of **4b**, respectively. After neutralization with NEt_3 at 23 °C, the absorption spectrum dominated by the neutral hypsochromic *Z*-isomer of diaryl-HI **4b** is obtained (light blue curve). Thus, adding small amounts of acid induces facile thermal *E* to *Z* isomerization of **4b** and spectroscopically distinct protonated species are formed at high acid concentrations. The latter are assigned to the thermally less stable protonated *Z*-[**4b**-H]$^+$ isomer and the thermally more stable protonated *E*-[**4b**-H]$^+$ isomer. The thermal stability of the neutral and protonated states are opposite, which is demonstrated in this experiment. Without cooling during the neutralization step with NEt_3, the finally resulting absorption spectrum is dominated by the neutral thermodynamically more stable *Z*-**4b** isomer (light blue curve). Source data are provided as Source Data File.

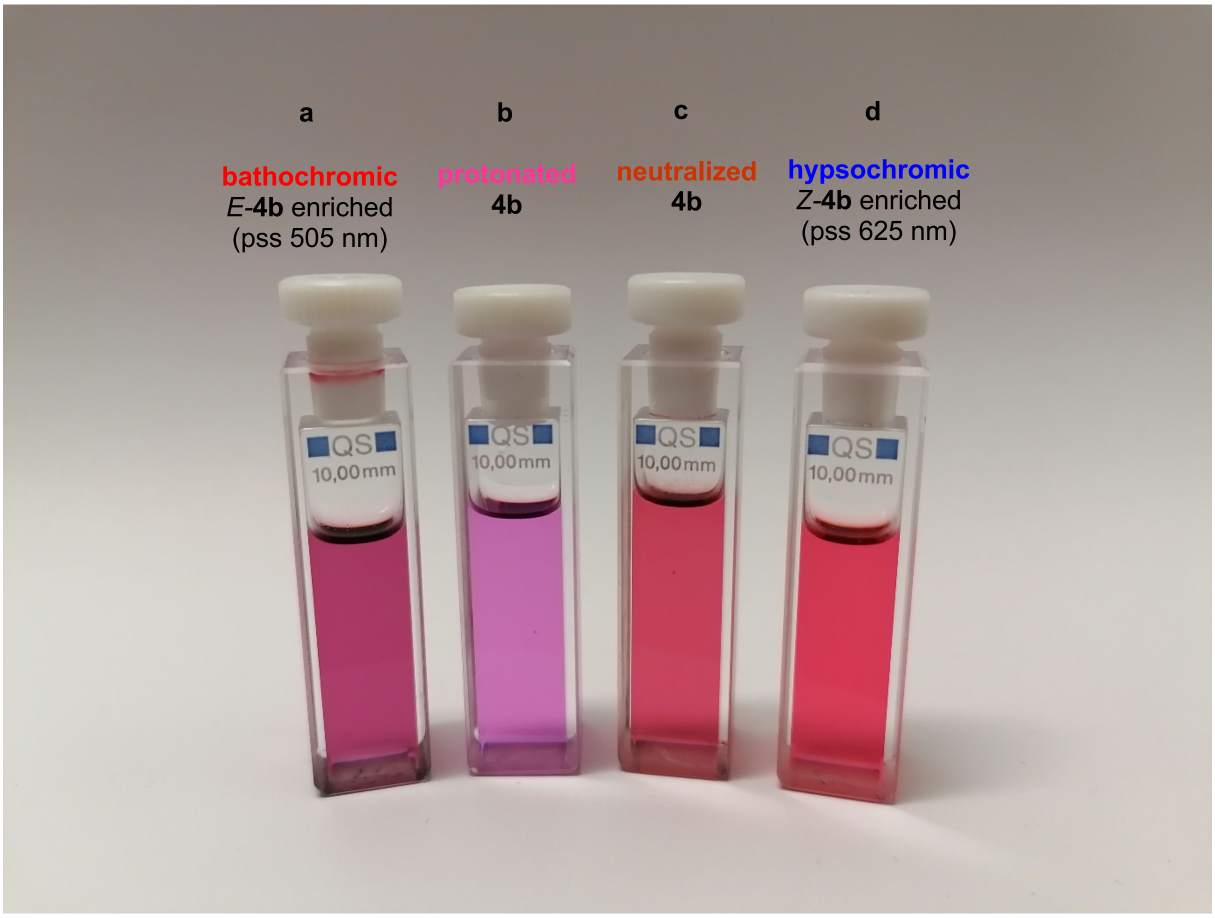

Supplementary Figure 129: Photograph of four diaryl-HI **4b** solutions of same concentrations in toluene at 23 °C. **a** Diaryl-HI **4b** solution was irradiated with 505 nm light until reaching the pss to enrich the bathochromic *E*-**4b** isomer. **b** *E*-**4b** solution mixed with excess of TFA. **c** *E*-**4b** solution mixed with excess of TFA followed by neutralization with NEt_3. **d** Diaryl-HI **4b** solution irradiated with 625 nm light until reaching the pss to enrich the hypsochromic *Z*-**4b** isomer. Source data are provided as Source Data File.

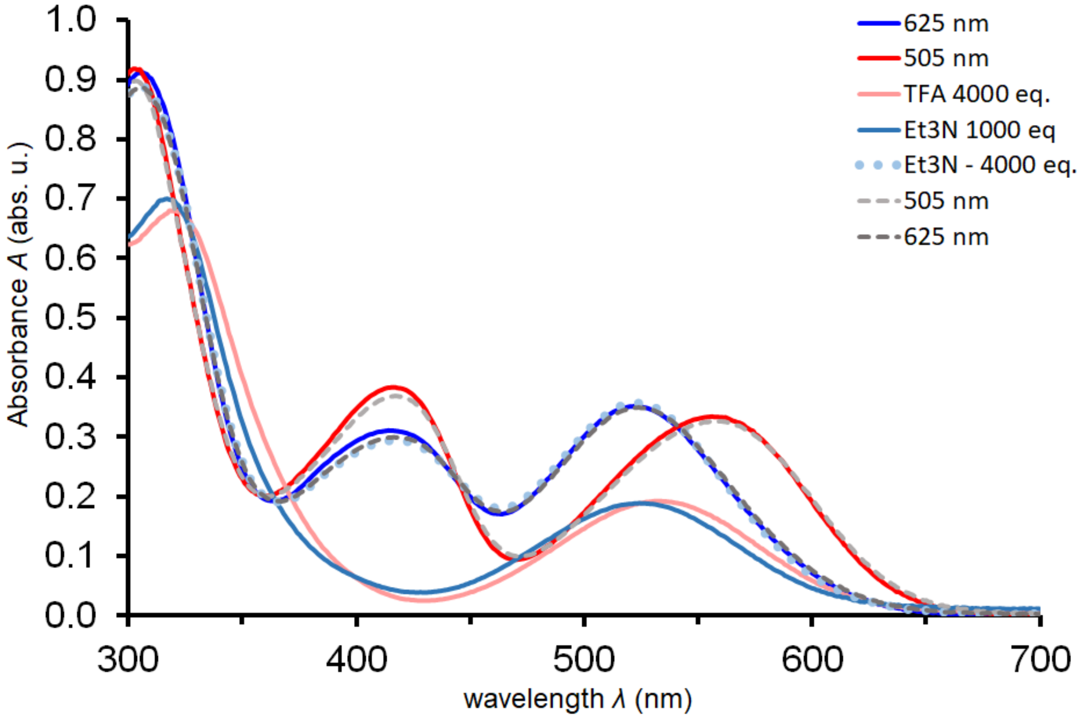

Supplementary Figure 130: Demonstration of 4 state switching of diaryl-HI **4b** in toluene solution at 23 °C. The *E*/*Z*-**4b** mixture was irradiated with light of 625 nm to the hypsochromic *Z*-**4b** pss state (blue curve). Afterwards, the hypsochromic *Z*-**4b** enriched solution was irradiated to the pss at 505 nm (bathochromic *E*-**4b** isomer, red curve). Then 4,000 equiv. of TFA were added and the thermodynamically more stable protonated *E*-[**4b**-H]$^+$ species was formed (salmon curve, fourth state). After addition of 1,000 equiv. of triethylamine, the protonated third state, the thermodynamically less stable *Z*-[**4b**-H]$^+$ species (cyan curve) was formed. Then, the *Z*-[**4b**-H]$^+$ state was neutralized with another 3,000 euiv. of triethylamine. The thermally fully equilibrated isomer content of *Z*-**4b** (light blue dotted curve) was regenerated. After neutralization, the *Z*-**4b** isomer was fully addressable with light of 505 nm and 625 nm (grey dotted curves) again. Source data are provided as Source Data File.

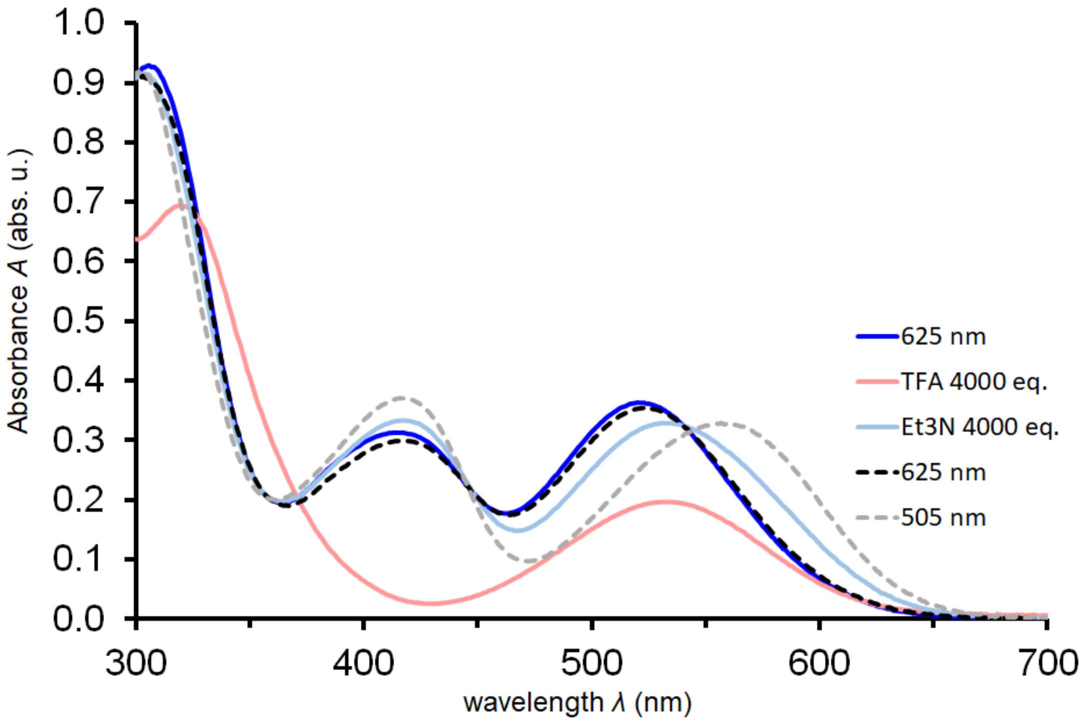

Supplementary Figure 131: Demonstration of 3 state switching of diaryl-HI **4b** in toluene solution at 23 °C. The *E*/*Z*-**4b** mixture was irradiated with light of 625 nm to the hypsochromic *Z*-**4b** pss state (blue curve). Then 4,000 equiv. of TFA were added and the protonated thermodynamically more stable *E*-[**4b**-H]$^+$ was reached (salmon curve, fourth state). Then, the *Z*-[**4b**-H]$^+$ state was neutralized with 4,000 equiv. of triethylamine at 23 °C to recover a *Z*-**4b** enriched isomer content (light blue curve). Afterwards, fully addressability with light of 505 nm and 625 nm (grey dotted curves) was retained. Source data are provided as Source Data File.

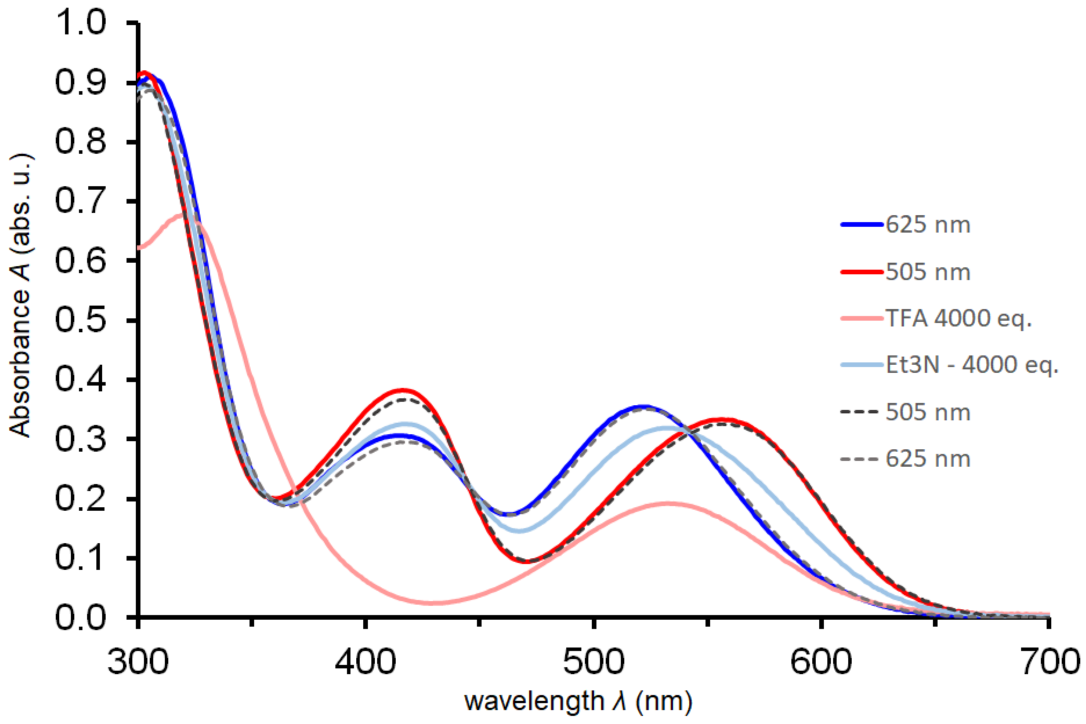

Supplementary Figure 132: Demonstration of 3 state switching of diaryl-HI **4b** in toluene solution at 23 °C. The *E*/*Z*-**4b** mixture was irradiated with light of 625 nm to the hypsochromic *Z*-**4b** pss state (blue curve), followed by irradiation with light of 505 nm to the bathochromic *E*-**4b** pss state (red curve). Then 4,000 equiv. of TFA were added and the protonated thermodynamically more stable *Z*-[**4b**-H]$^+$ species was reached (salmon curve, fourth state). Then, the *Z*-[**4b**-H]$^+$ state was neutralized with 4,000 equiv. of triethylamine to recover a *Z*-**4b** (light blue curve) enriched solution. Afterwards, fully addressability with light of 505 nm and 625 nm (grey dotted curves) was retained. Source data are provided as Source Data File.

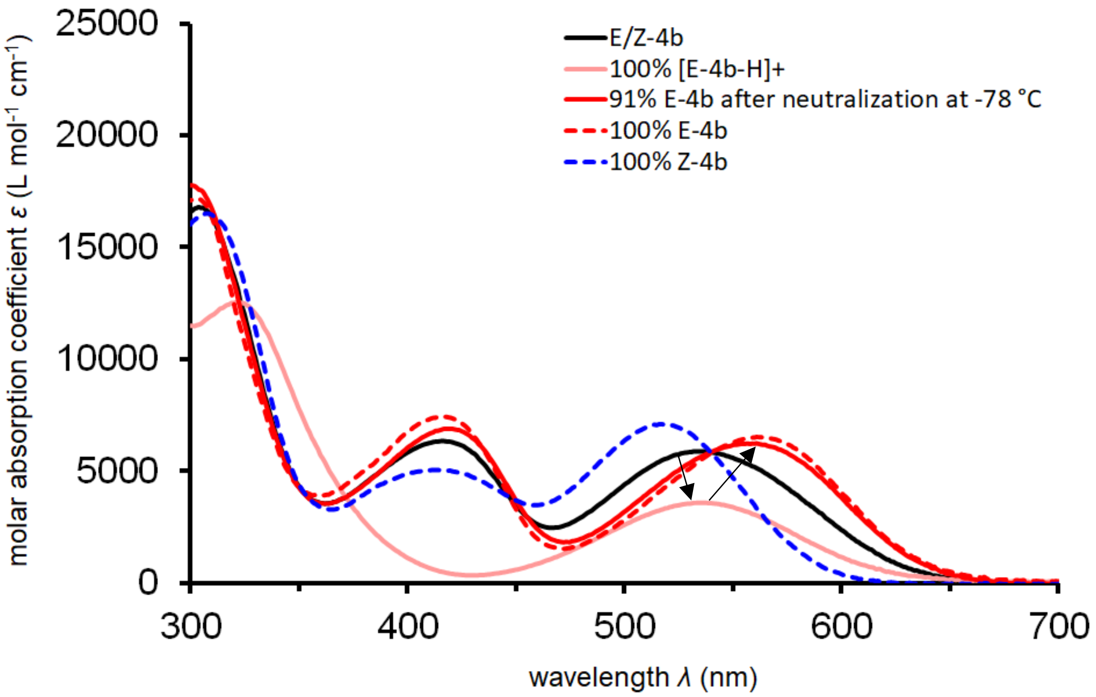

Supplementary Figure 133: Acid/base induced enrichment of the thermodynamically less stable *E*-**4b** isomer. Strong enrichment of *E*-**4b** is possible after addition of a large excess of TFA to a E/Z-**4b** mixture and subsequent neutralization with triethylamine. To a *E*/*Z*-**4b** mixture (black curve) in toluene solution, concentrated TFA (5,000 equiv.) was added at 23 °C. The solution containing newly formed *E*-[**4b**-H]$^+$ (salmon curve) was then cooled to –78 °C and triethylamine (5,000 equiv.) was added. The solution was allowed to reach 23 °C under fast shaking resulting in strongly enriched *E*-**4b** isomer (91%, red curve). Spectra were scaled to the known isosbestic points of the previously determined molar absorption coefficients of the pure *E* and *Z* isomers (100% *E*-isomer, 100% *Z*-isomer, dotted red and blue curves). Previous acid/base experiments show, that the isosbestic points do not change their position on the x-axis after acid/base treatment but change their position on the y-axis more or less pronounced correlated to dilution effects. The individual isomer content after neutralization with base was determined according to Supplementary Note 4 via (3) or (4) with the constraints that $x_{E+}^{E} + x_{E+}^{Z} = 1$ or $x_{Z+}^{E} + x_{Z+}^{Z} = 1$. Source data are provided as Source Data File.

MS-APPI and MS-ESI mass experiments in toluene in the presence of different amounts of TFA validate, that twofold protonated HIs in the form of *E*/*Z*-[**4b**-2H]$^{2+}$ were not formed.

Supplementary Table 8: 19 HI **4b** samples in different toluene/TFA mixtures have been analyzed via APPI mass spectrometry at 250 °C, 5.2 bar pressure, 1.2 L min^{-1} flow rate and positive ion detection mode. The amount of TFA was increased in every consecutive measurement. The HI concentration was kept constant for each measurement (2.22 × 10^{-5} M).

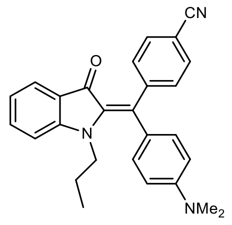

Chemical Formula: $C_{27}H_{25}N_3O$

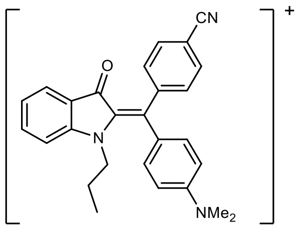

Chemical Formula: $C_{27}H_{25}N_3O^+$

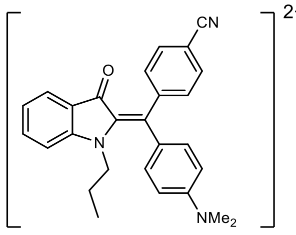

Chemical Formula: $C_{27}H_{25}N_3O^{2+}$

exp.	equiv. TFA (-)	TFA/toluene (vol%)	[M]$^+$ [$C_{27}H_{25}N_3O$]$^+$ (m/z)	[M]$^{2+}$ [$C_{27}H_{25}N_3O$]$^{2+}$ (m/z)
**setpoin**	**-**	**-**	**407.1998; 408.2031; 409.2065**	**203.5993; 204.1009; 204.6024**
1	0	0	407.1995; 408.2053; 409.2089	-
2	10	0.0020	407.1996; 408.2045; 409.2081	-
3	20	0.0030	407.1997; 408.2050; 409.2086	-
4	50	0.0090	407.1991; 408.2047; 409.2085	-
5	100	0.017	407.1998; 408.2055; 409.2093	-
6	200	0.034	407.1990; 408.2046; 409.2083	-
7	500	0.086	407.1996; 408.2056; 409.2092	-
8	1,000	0.17	407.1997; 408.2057; 409.2094	-
9	2,000	0.34	407.1992; 408.2052; 409.2090	-
10	3,000	0.52	407.1994	-
11	4,000	0.69	407.1991; 408.2046; 409.2084	-
12	5,000	0.86	407.1991; 408.2050; 409.2088	-
13	10,000	1.7	407.1993; 408.2050; 409.2086	-
14	100,000	17	407.1992; 408.2041; 409.2079	-
15	200,000	34	407.1995	-
16	300,000	52	407.1994	-
17	400,000	69	407.1986	-
18	500,000	86	407.1986; 408.2055; 409.2091	-
19	576,000	99	407.1985; 408.2053; 409.2091	-

Supplementary Table 9: 2 HI **4b** samples in a toluene and TFA have been analyzed via ESI mass spectrometry at 200 °C, 0.3 bar pressure, 3.5 L min^{-1} flow rate and positive ion detection mode. The HI concentration was 2.22 × 10^{-5} M in each measurement.

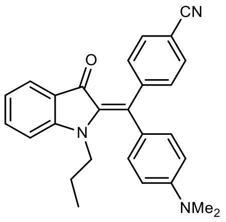

Chemical Formula: $C_{27}H_{25}N_3O$

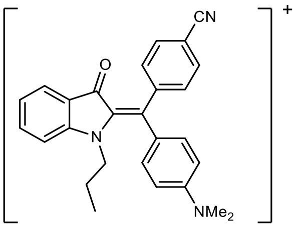

Chemical Formula: $C_{27}H_{25}N_3O^{+}$

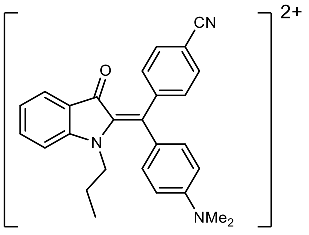

Chemical Formula: $C_{27}H_{25}N_3O^{2+}$

exp.	equiv. TFA (-)	TFA/toluene (vol%)	$[M]^{+}$ $[C_{27}H_{25}N_3O]^{+}$ (m/z)	$[M]^{2+}$ $[C_{27}H_{25}N_3O]^{2+}$ (m/z)
**setpoint**	**-**	**-**	**407.1998; 408.2031; 409.2065**	**203,5993; 204.1009; 204.6024**
1	0	0	407.1934; 408.2029; 409.2039	-
19	576,000	99	407.1965; 408.2038; 409.2073	203.6006; 204.1030; 204.6073

Supplementary Table 10: 19 HI **4b** samples in different toluene/TFA mixtures have been analyzed via APPI mass spectrometry at 250 °C, 5.2 bar pressure, 1.2 L min^{-1} flow rate and positive ion detection mode. The amount of TFA was increased in every consecutive measurement. The HI concentration was kept constant for each measurement (2.22×10^{-5} M).

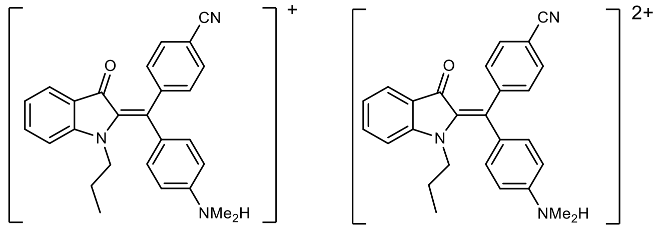

Chemical Formula: $C_{27}H_{26}N_3O^+$ Chemical Formula: $C_{27}H_{26}N_3O^{2+}$

exp.	equiv. TFA (-)	TFA/toluene (vol%)	$[M-H]^+$ $[C_{27}H_{26}N_3O]^+$ (m/z)	$[M-H]^{2+}$ $[C_{27}H_{26}N_3O]^{2+}$ (m/z)
**setpoint**	**-**	**-**	**408.2070; 409.2104; 410.2137**	**204.1032; 204.6049; 205.1066**
1	0	0	-	-
2	10	0.0020	-	-
3	20	0.0030	-	-
4	50	0.0090	-	-
5	100	0.017	-	-
6	200	0.034	-	-
7	500	0.086	-	-
8	1,000	0.17	-	-
9	2,000	0.34	-	-
10	3,000	0.52	-	-
11	4,000	0.69	-	-
12	5,000	0.86	-	-
13	10,000	1.7	-	-
14	100,000	17	-	-
15	200,000	34	-	-
16	300,000	52	-	-
17	400,000	69	-	-
18	500,000	86	-	-
19	576,000	99	-	-

Supplementary Table 11: 19 HI **4b** samples in different toluene/TFA mixtures have been analyzed via APPI mass spectrometry at 250 °C, 5.2 bar pressure, 1.2 L min^{-1} flow rate and positive ion detection mode. The amount of TFA was increased in every consecutive measurement. The HI concentration was kept constant for each measurement (2.22 × 10^{-5} M).

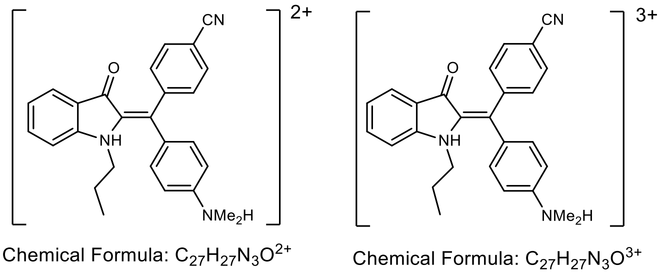

Exp.	equiv. TFA (-)	TFA/toluene (vol%)	$[M-2H]^{2+}$ $[C_{27}H_{27}N_3O]^{2+}$ (m/z)	$[M-2H]^{3+}$ $[C_{27}H_{27}N_3O]^{3+}$ (m/z)
**setpoint**	**-**	**-**	**204.6072; 205.1088; 205.6105**	**136.4046, 136.7390, 137.0735**
1	0	0	-	-
2	10	0.0020	-	-
3	20	0.0030	-	-
4	50	0.0090	-	-
5	100	0.017	-	-
6	200	0.034	-	-
7	500	0.086	-	-
8	1,000	0.17	-	-
9	2,000	0.34	-	-
10	3,000	0.52	-	-
11	4,000	0.69	-	-
12	5,000	0.86	-	-
13	10,000	1.7	-	-
14	100,000	17	-	-
15	200,000	34	-	-
16	300,000	52	-	-
17	400,000	69	-	-
18	500,000	86	-	-
19	576,000	99	-	-

An acid titration experiment with TFA-d_1 was performed with a thermally equilibrated *Z*/*E*-**4b** mixture at constant diaryl-HI concentration in toluene-d_8 at 25 °C to understand the acid/base-induced isomerization processes. The concentration of thermodynamic equilibrated Z/E-**4b** (79% *Z* : 21% *E*) was kept constant at 1.12 mM and the equivalents of TFA were raised from 0.1 to 135. By tracing the shifting signals of the *Z* and *E* isomers and integrating them it could be observed that isomerization from *Z* to *E* configuration occurs until 100% of the *E*-[**4b**-H]$^+$ species is present at high acid content. The *E*-[**4b**-H]$^+$ species was additionally validated through a comprehensive NMR analysis in TFA-d_1 solution (see further below).

Beforehand, the protons of *E*/*Z*-**4b** in toluene were assigned to the structure and a 1D ^{1}H-^{1}H NOE NMR experiment in toluene confirmed that the thermodynamic more stable neutral isomer is the *Z*-**4b** species.

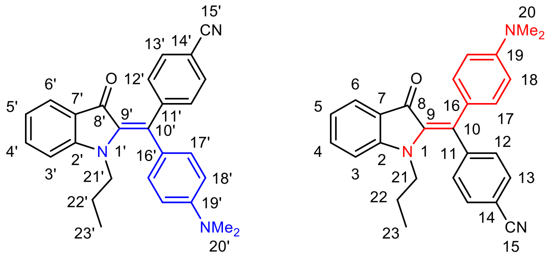

hypsochromic *Z*-**4b**
thermodynamic more stable

bathochromic *E*-**4b**
thermodynamic less stable

***Z* isomer (major isomer): ^{1}H NMR** (601 MHz, toluene-d_8, 25 °C) δ (ppm) = 7.70 – 7.67 (m, 1H, H6'), 6.94 – 6.90 (m, 2H, H17'), 6.69 – 6.63 (m, 1H, H5'), 6.60 (d, $^3J_{H-H}$ = 8.2 Hz, 1H, H3'), 6.39 – 6.34 (m, 2H, H18'), 3.27 – 3.20 (m, 2H, H21'), 2.44 (s, 6H, H20'), 1.26 – 1.17 (m, 2H, H22'), 0.46 (t, *J* = 7.4 Hz, 3H, H23'), H4', H12', and H13' probably beneath the toluene-d_8 signals.

***E* isomer (minor isomer): ^{1}H NMR** (601 MHz, toluene-d_8, 25 °C) δ (ppm) = 7.75 (dd, $^3J_{H-H}$ = 7.3 Hz, $^4J_{H-H}$ 1.2 Hz, 1H, H6), 7.18 – 7.15 (m, 2H, H13), 6.89 – 6.83 (m, 2H, H17), 6.69 – 6.63 (m, 1H, H5), 6.55 (d, $^3J_{H-H}$ = 8.2 Hz, 1H, H3), 6.48 – 6.45 (m, 2H, H18), 2.79 (t, $^3J_{H-H}$ = 7.2 Hz, 1H, H21), 2.50 (s, 6H, H20), 1.15-1.08 (m, 2H, H22), H4, and H12 probably beneath the toluene-d_8 signals, H23 probably beneath the water signal at 0.43 ppm.

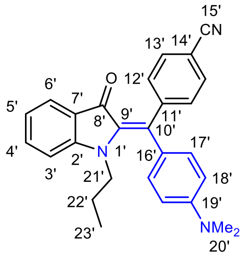

hypsochromic *Z*-**4b**
thermodynamic more stable

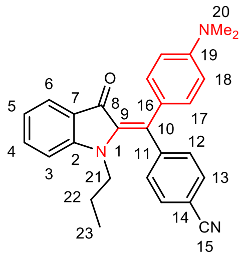

bathochromic *E*-**4b**
thermodynamic less stable

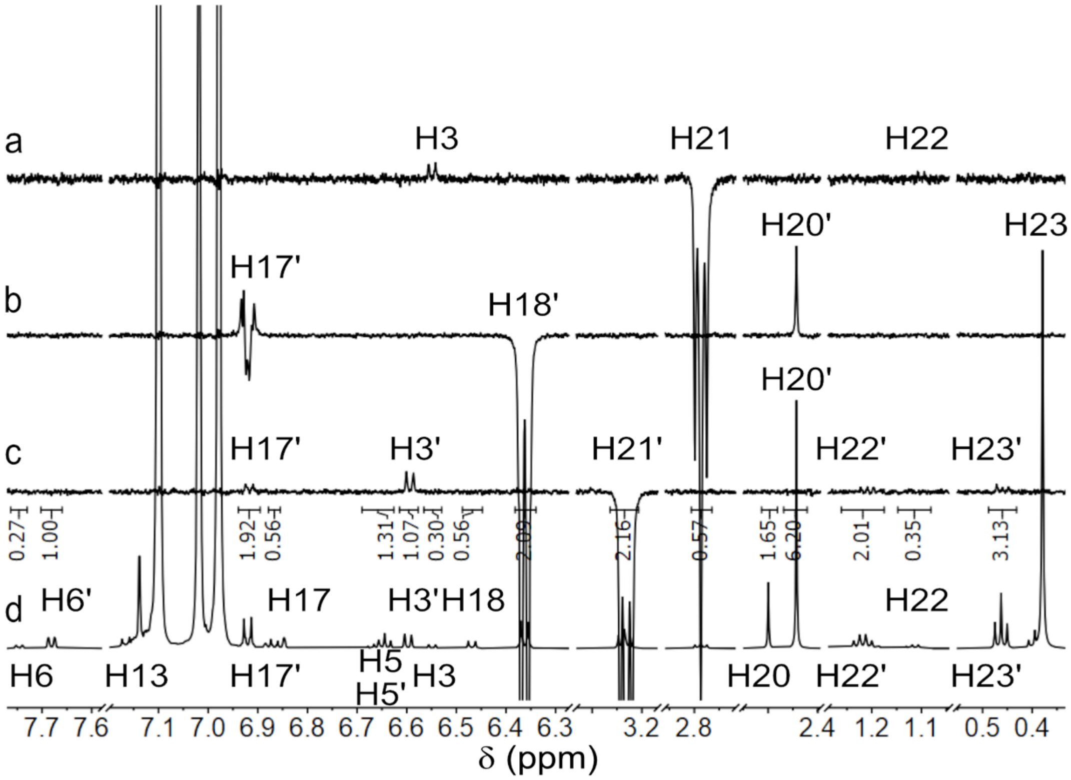

Supplementary Figure 134: 1D ^{1}H-^{1}H NOE NMR experiments (601 MHz, toluene-d_8, 25 °C) with a thermally equilibrated *E*/*Z*-**4b** mixture (*E* : *Z* = 21% : 79%) revealing *E*-**4b** to be the thermodynamically less stable isomer and *Z*-**4b** to be the thermodynamically more stable isomer. **a** Irradiating the protons at 2.79 ppm (H21 of *E*-**4b**) leads to magnetization transfer to the signals at 6.55 ppm (H3 of *E*-**4b**) and 1.15-1.08 ppm (H22 of *E*-**4b**). **b** Irradiating the protons at 6.39 – 6.34 ppm (H18' of *Z*-**4b**) leads to magnetization transfer to the signals between 6.94 – 6.90 ppm (H17' of *Z*-**4b**) and 2.44 ppm (H20' of *Z*-**4b**). **c** Irradiating the protons at 3.27 – 3.20 ppm (H21' of *Z*-**4b**) leads to magnetization transfer to the signals at 6.60 (H3' of *Z*-**4b**), 6.94 – 6.90 ppm (H17' of *Z*-**4b**), 1.26 – 1.17 ppm (H22' of *Z*-**4b**), and 0.46 ppm (H23' of *Z*-**4b**). **d** partial ^{1}H NMR spectrum of *E*/*Z*-**4b** (601 MHz, toluene-d_8, 25 °C). The protons at H21 does not show a magnetization transfer to the aromatic protons associated with the dimethylaniline carrying aromat, which confirms the *E* configuration for the thermodynamically less stable *E*-**4b** isomer. The protons of H18', H17' and H20' belong to the same spin system and therefore can be associated to the aryl substituent carrying the dimethylaniline group. There is a magnetization transfer from the protons of H21' to the protons of H17', which confirms that the major isomer (thermodynamically more stable) is the *Z*-**4b** isomer. Source data are provided as Source Data File.

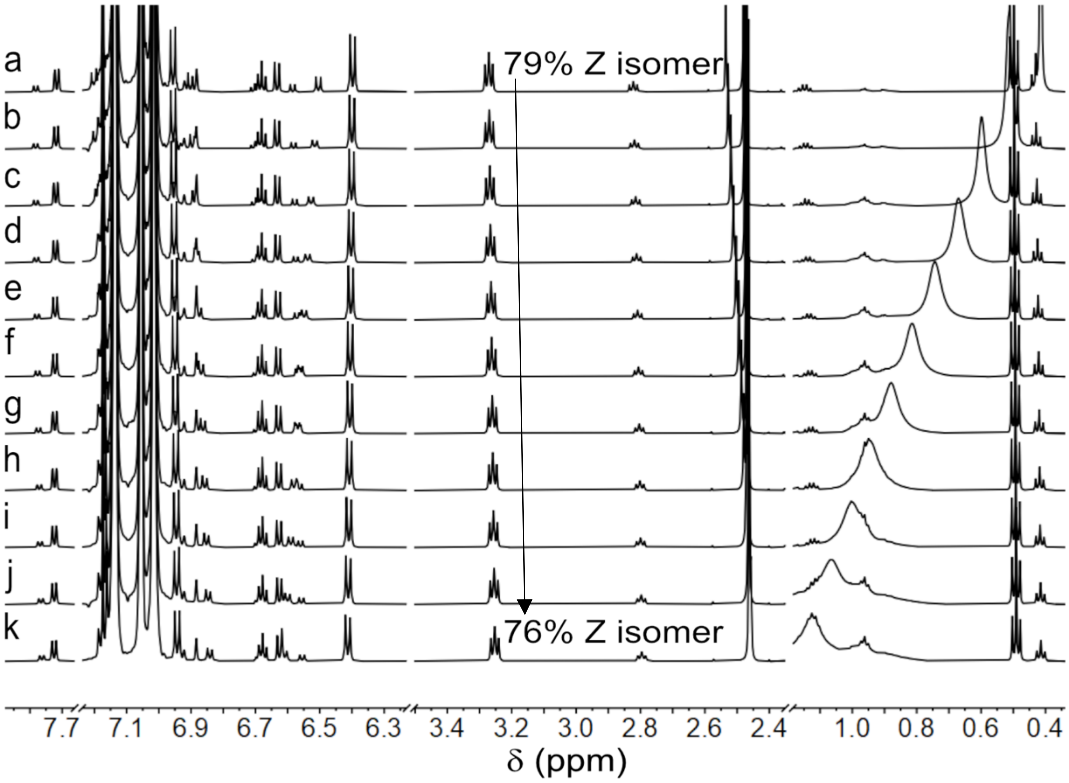

Supplementary Figure 135: ^{1}H NMR titration experiment (601 MHz, toluene-d_8, 25 °C) of thermally equilibrated *E*/*Z*-**4b** mixture (*E* : *Z* = 21% : 79%) with TFA-d_1. From a to k, the figure depicts the partial ^{1}H NMR spectra of *E*/*Z*-**4b** recorded after addition of 0 to 0.90 equiv. TFA-d_1. The concentration of **4b** was kept constant at 1.12 mM, while the TFA-d_1 concentration was increased. The amount of *Z* isomer was slightly decreasing with increasing amount of TFA-d_1. **a** 0 equiv. TFA-d_1. **b** 0.10 equiv. TFA-d_1. **c** 0.20 equiv. TFA-d_1. **d** 0.29 equiv. TFA-d_1. **e** 0.39 equiv. TFA-d_1. **f** 0.48 equiv. TFA-d_1. **g** 0.57 equiv. TFA-d_1. **h** 0.65 equiv. TFA-d_1. **i** 0.74 equiv. TFA-d_1. **j** 0.82 equiv. TFA-d_1. **k** 0.90 equiv. TFA-d_1. Source data are provided as Source Data File.

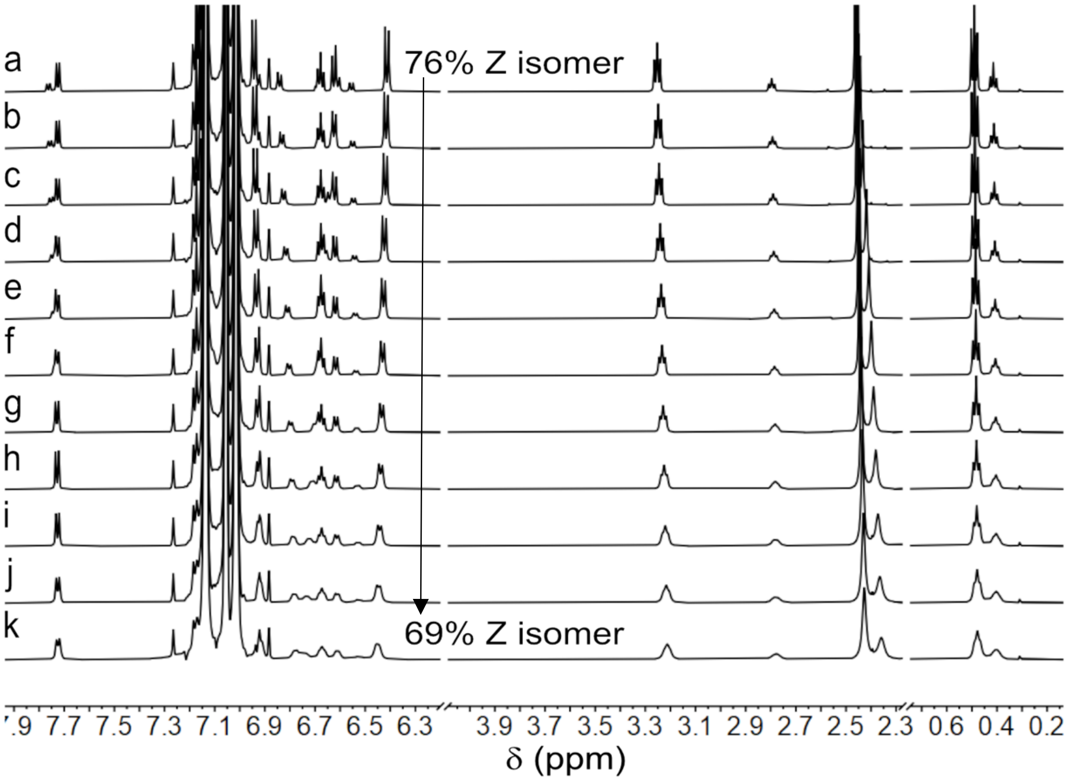

Supplementary Figure 136: ^{1}H NMR titration experiment (601 MHz, toluene-d_8, 25 °C) of *E*/*Z*-**4b** mixture (*E* : *Z* = 24% : 76%) with TFA-d_1. From a to k, the figure depicts the partial ^{1}H NMR spectra of *E*/*Z*-**4b** recorded after addition of 0.90 to 2.4 equiv. TFA-d_1. The concentration of **4b** was kept constant at 1.12 mM, while the TFA-d_1 concentration was increased. The amount of *Z* isomer was decreasing with increasing amount of TFA-d_1. **a** 0.9 equiv. TFA-d_1. **b** 1.1 equiv. TFA-d_1. **c** 1.2 equiv. TFA-d_1. **d** 1.4 equiv. TFA-d_1. **e** 1.6 equiv. TFA-d_1. **f** 1.7 equiv. TFA-d_1. **g** 1.9 equiv. TFA-d_1. **h** 2.0 equiv. TFA-d_1. **i** 2.2 equiv. TFA-d_1. **j** 2.3 equiv. TFA-d_1. **k** 2.4 equiv. TFA-d_1. Source data are provided as Source Data File.

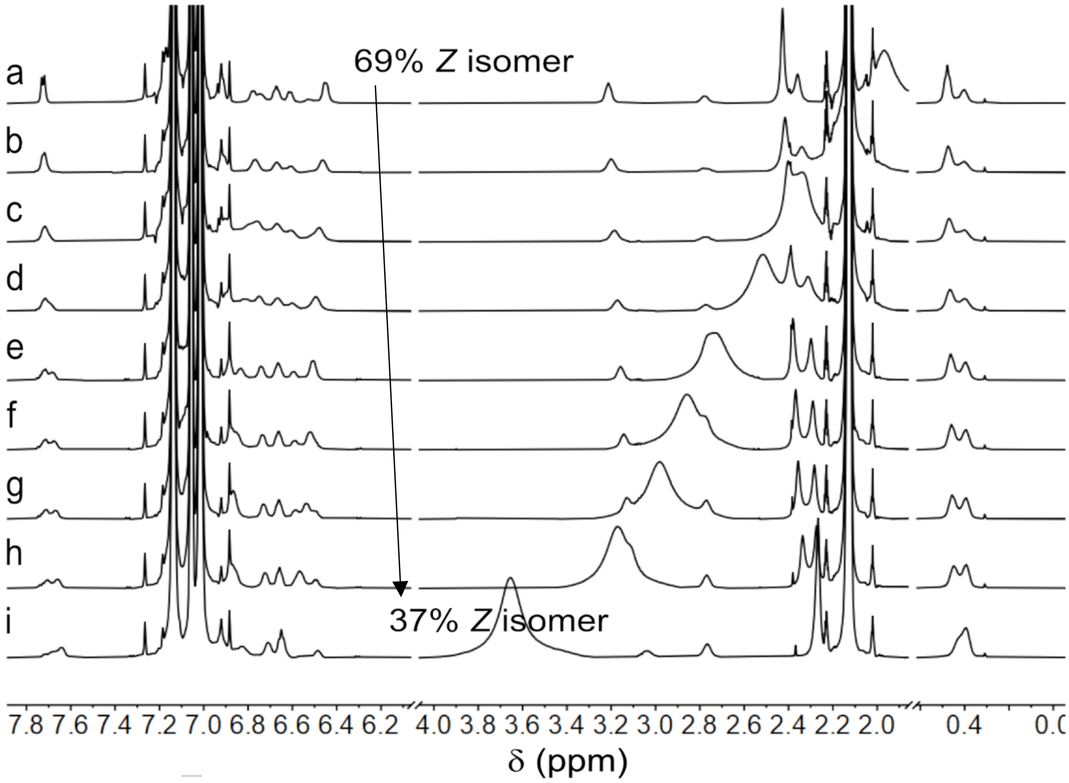

Supplementary Figure 137: ^{1}H NMR titration experiment (601 MHz, toluene-d_8, 25 °C) of *E*/*Z*-**4b** mixture (*E* : *Z* = 31% : 69%) with TFA-d_1. From a to i, the figure depicts the partial ^{1}H NMR spectra of *E*/*Z*-**4b** recorded after addition of 2.4 to 7.4 equiv. TFA-d_1. The concentration of **4b** was kept constant at 1.12 mM, while the TFA-d_1 concentration was increased. The amount of *Z* isomer was decreasing with increasing amount of TFA-d_1. **a** 2.4 equiv. TFA-d_1. **b** 2.8 equiv. TFA-d_1. **c** 3.2 equiv. TFA-d_1. **d** 3.6 equiv. TFA-d_1. **e** 3.9 equiv. TFA-d_1. **f** 4.3 equiv. TFA-d_1. **g** 4.6 equiv. TFA-d_1. **h** 5.3 equiv. TFA-d_1. **i** 7.4 equiv. TFA-d_1. Source data are provided as Source Data File.

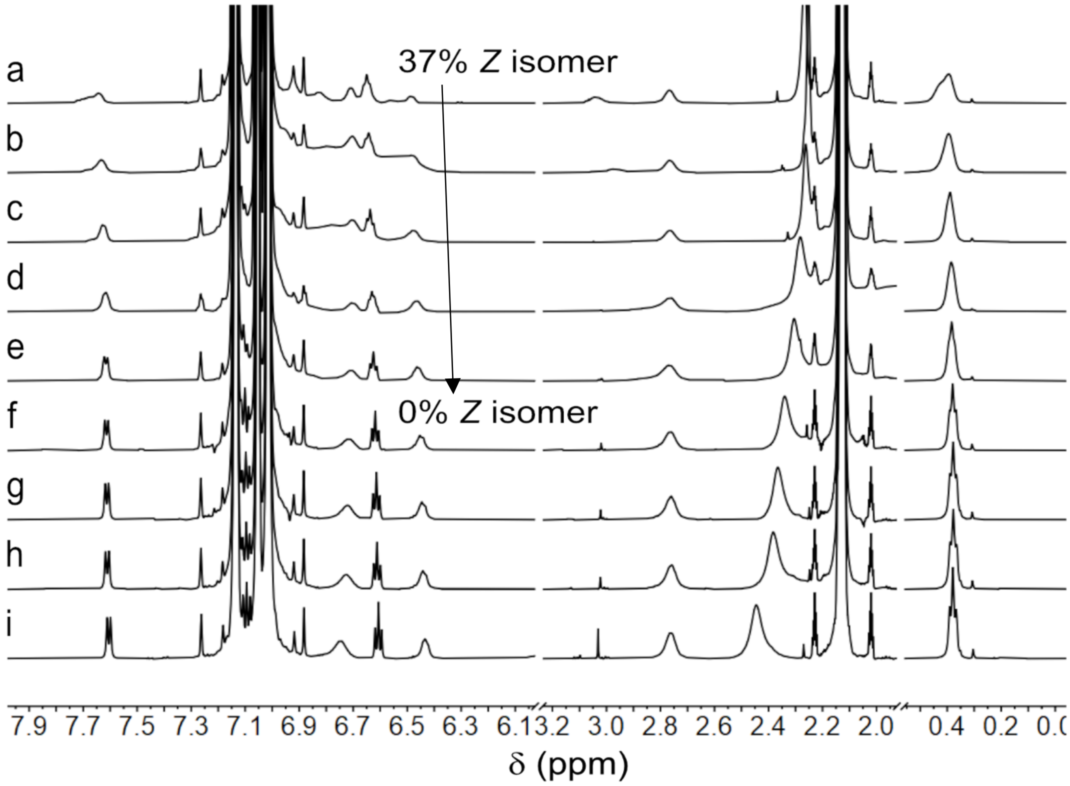

Supplementary Figure 138: ^{1}H NMR titration experiment (601 MHz, toluene-d_8, 25 °C) of *E*/*Z*-**4b** mixture (*E* : *Z* = 63% : 37%) with TFA-d_1. From a to h, partial ^{1}H NMR spectra of *E*/*Z*-**4b** are depicted, recorded after addition of 7.4 to 135 equiv. TFA-d_1. The concentration of **4b** was kept constant at 1.12 mM, while the TFA-d_1 concentration was increased. The amount of *Z* isomer was decreasing to 0% with increasing amount of TFA-d_1. **a** 7.4 equiv. TFA-d_1. **b** 11 equiv. TFA-d_1. **c** 15 equiv. TFA-d_1. **d** 22 equiv. TFA-d_1. **e** 29 equiv. TFA-d_1. **f** 43 equiv. TFA-d_1. At this point, no *Z* isomer content was present anymore. **g** 56 equiv. TFA-d_1. **h** 69 equiv. TFA-d_1. **i)** 135 equiv. TFA-d_1. Source data are provided as Source Data File.

The ^{1}H NMR titration experiment shows the enrichment of the thermodynamic more stable protonated *E*-[**4b**-H]$^+$ species after adding increasing amounts of TFA-d_1. The population of the thermodynamic less stable *Z*-[**4b**-H]$^+$ species was not obviously visible at NMR concentrations of **4b** = 1.12 mM, as Supplementary Figure 139 because of broadness of signals and smaller shifts. However, when comparing the ^{1}H NMR titration experiment with the UV/Vis titration experiments that were measured at 100 times lower concentrations (63 μM) it becomes obvious that there is a distinct population of a third state, i.e. the *Z*-[**4b**-H]$^+$ isomer. The UV/Vis protonation experiments for all measured HIs **4** directly evidence that protonation of the thermodynamic more stable neutral isomer (in this case the *Z*-**4b** isomer) occurs. This third state was also visible by the color change of the solution which is related to the loss of the absorbance band at 420 nm.

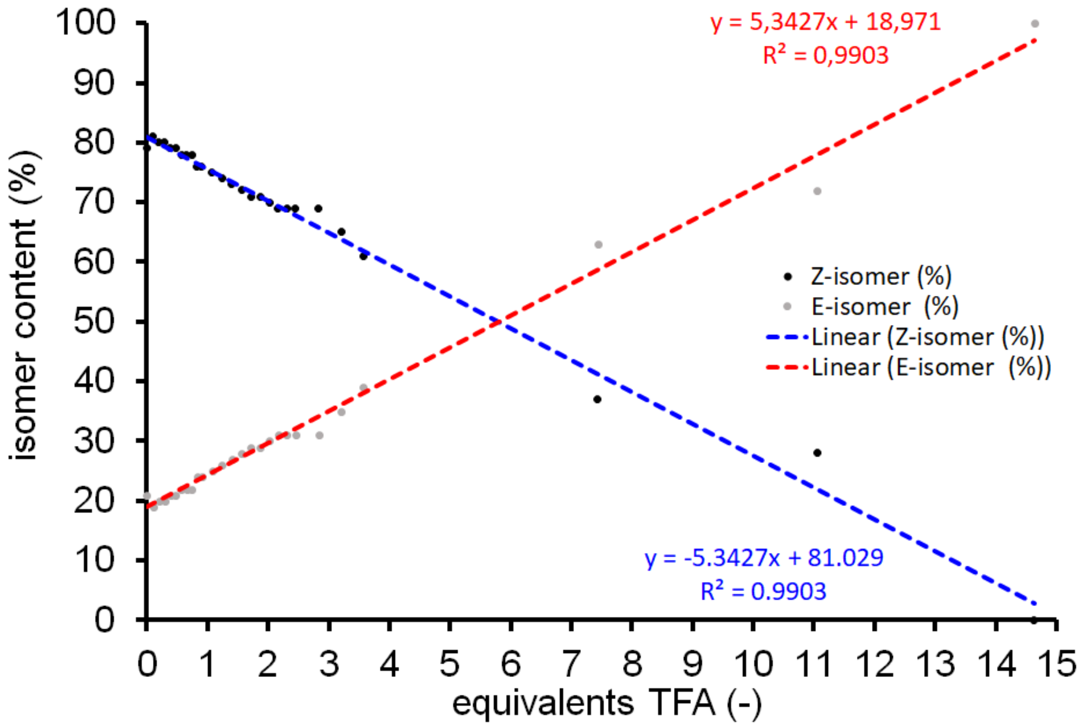

Supplementary Figure 139: Change of *E*/*Z* isomer ratio of **4b** after addition of TFA-d_1, starting with a thermally equilibrated mixture of **4b** (*E* : *Z* = 21% : 79%). Changes in the isomer composition were determined by ^{1}H NMR spectroscopy (601 MHz, toluene-d_8, 25 °C) through integration of indicative signals. Consecutive ^{1}H NMR measurements were performed at a constant concentration of **4b** (1.12 mM), while the TFA-d_1 concentration was subsequently elevated from 0 equiv. to 14.6 equiv. The measurements were completed after the *E* isomer content reached 100%. Source data are provided as Source Data File.

Diaryl-HI **4b** was dissolved in pure TFA-d_1 and analyzed with ^{1}H NMR, ^{13}C NMR, 2D ^{1}H-^{1}H COSY NMR, 2D ^{1}H-^{13}C HSQC NMR, 2D ^{1}H-^{13}C HMBC NMR, and 1D ^{1}H-^{1}H NOE NMR spectroscopy. It was found that the *E*-[**4b**-H]$^+$ species is the only species present at high acid content. Due to the fast relaxation of the magnetization of the carbon atoms, the assignments of the tertiary and quartenary carbon atoms were mostly performed through the magnetization transfer of adjacent hydrogen atoms via 2D ^{1}H-^{13}C NMR experiments.

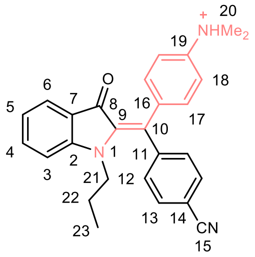

100% *E*-[4b-H]$^+$ isomer in pure TFA-d_1:

^{1}H NMR (601 MHz, TFA-d_1, referenced to THF increment at 1.73 ppm, 25 °C) δ (ppm) = 7.55 (d, $^3J_{H\text{-}H}$ = 7.9 Hz, 2H, H13), 7.43 – 7.32 (m, 4H, H4, H6, H18), 7.27 (d, $^3J_{H\text{-}H}$ = 8.0 Hz, 2H, H17), 7.19 (d, $^3J_{H\text{-}H}$ = 8.0 Hz, 2H, H12), 6.84 (d, $^3J_{H\text{-}H}$ = 8.5 Hz, 1H, H3), 6.75 (t, $^3J_{H\text{-}H}$ = 7.6 Hz, 1H, H5), 3.18 (s, 6H, H20), 3.07 (t, $^3J_{H\text{-}H}$ = 7.3 Hz, 2H, H21), 1.23 – 1.15 (m, 2H, H22), 0.35 (t, $^3J_{H\text{-}H}$ = 7.4 Hz, 3H, H23).

^{13}C NMR (151 MHz, TFA-d_1, 25 °C) δ (ppm) = 181.0 (C8), 159.5 (C2), 146.1 (C11), 144.7 (C19), 142.5 (C16), 142.4 (C4), 140.2 (C9), 136.0 (2C, C17), 134.7 (2C, C13), 133.9 (2C, C12), 127.7 (2C, C18), 124.1 (C5), 122.2 (C6), 122.0 (C7), 114.1 (C14), 113.8 (C3), 49.1 (2C, C20), 49.0 (C21), 22.1 (C22), 11.3 (C23), C10 not detected.

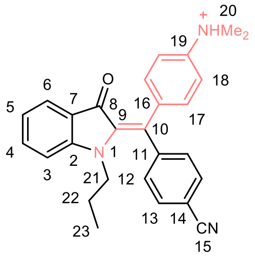

E-[**4b**-H]$^+$

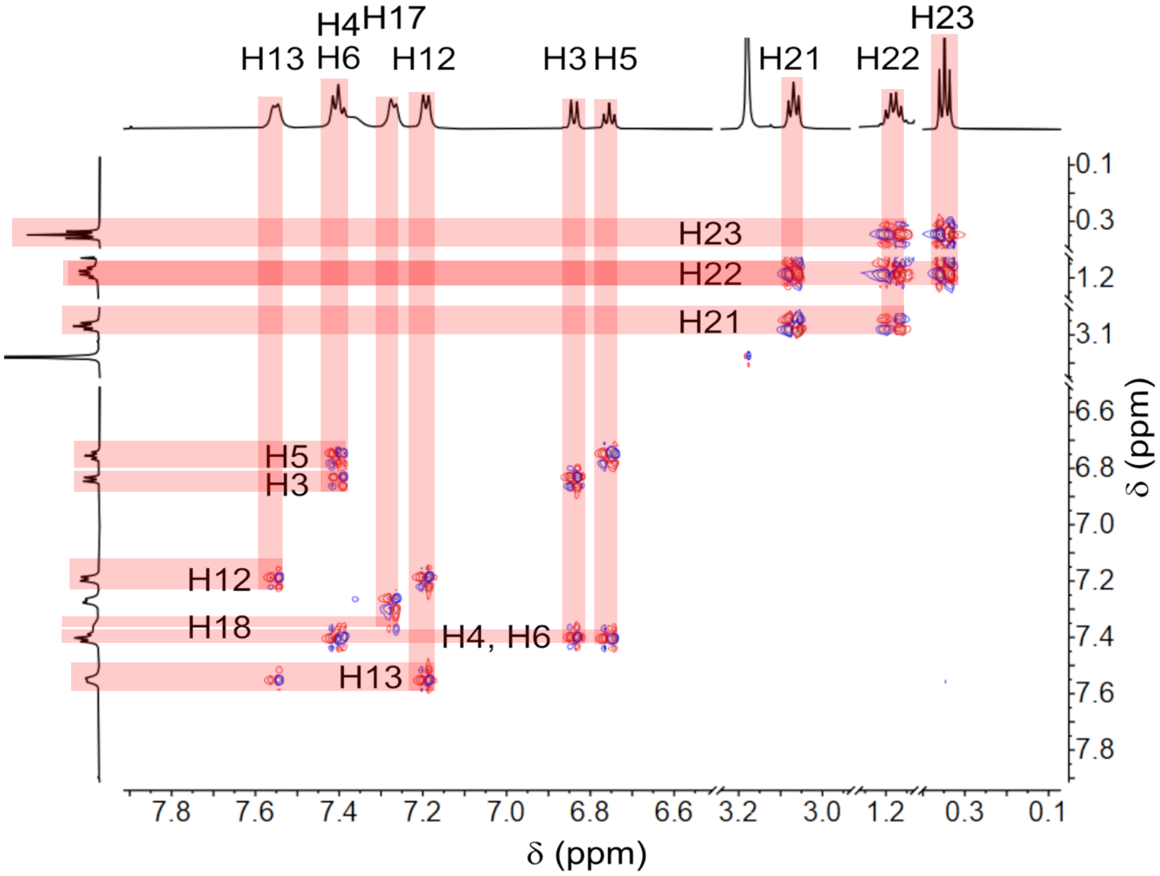

Supplementary Figure 140: Partial 2D ^{1}H-^{1}H COSY NMR spectrum (601 MHz, TFA-d_1, 25 °C) of *E*-[**4b**-H]$^+$. The cross signals between neighboring protons that show $^3J_{H\text{-}H}$ couplings confirm the structural elucidation. Source data are provided as Source Data File.

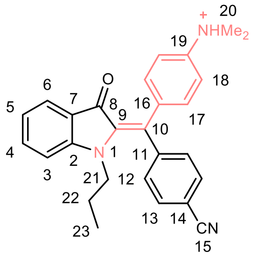

E-[**4b**-H]$^+$

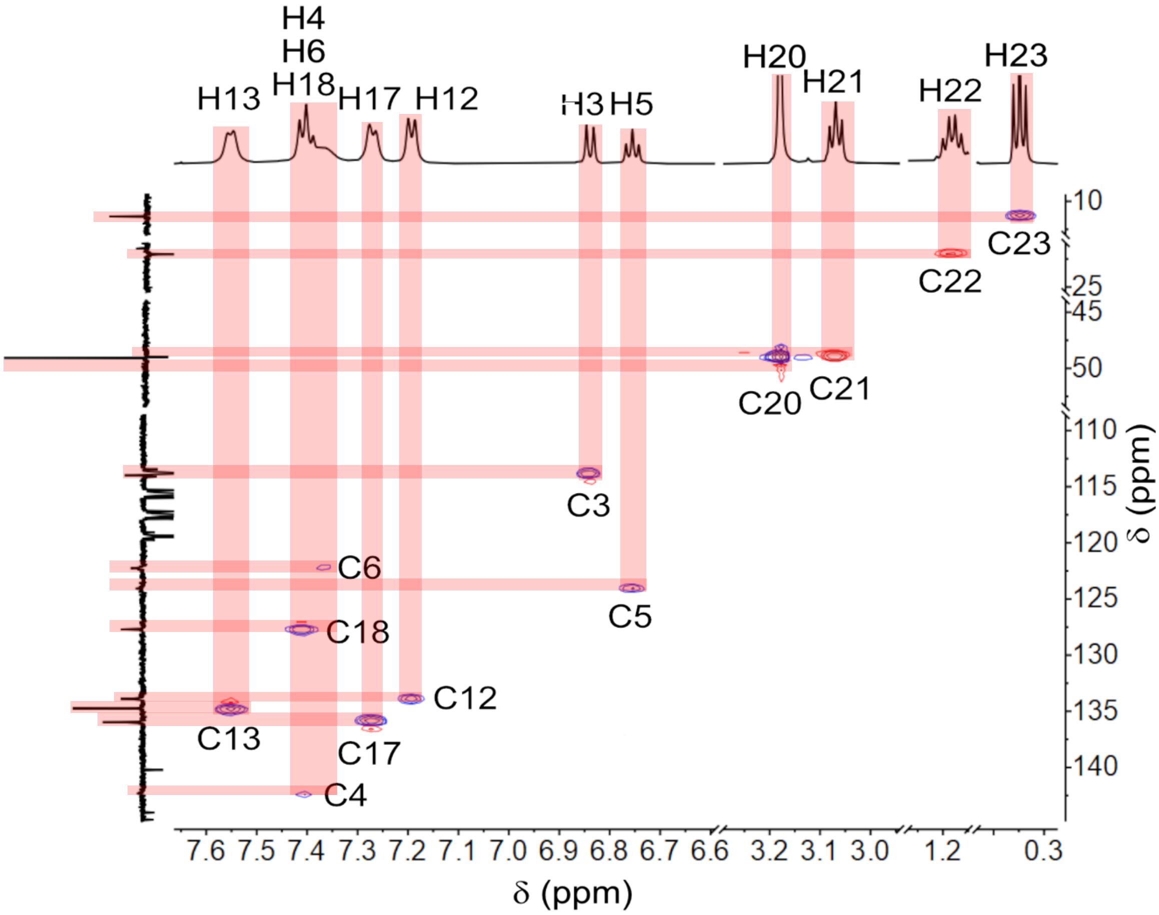

Supplementary Figure 141: Partial 2D ^{1}H-^{13}C HSQC NMR spectrum (601 MHz, TFA-d_1, 25 °C) of *E*-[**4b**-H]$^+$. The cross signals between protons and their carrying carbon atoms confirm the structural assignment. Source data are provided as Source Data File.

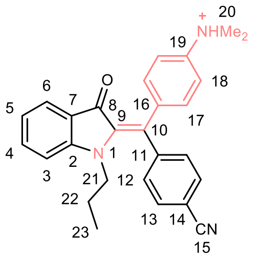

E-[**4b**-H]$^+$

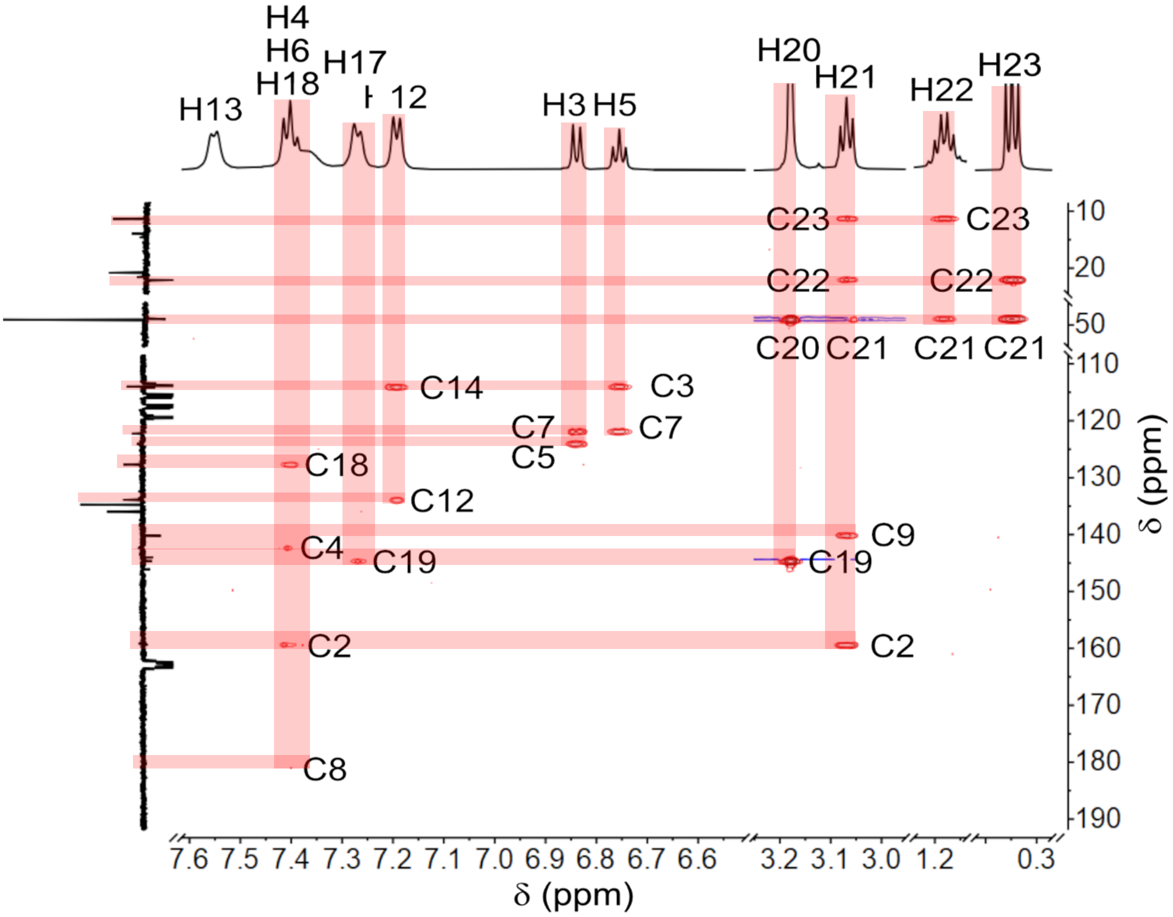

Supplementary Figure 142: Partial 2D ^{1}H-^{13}C HMBC NMR spectrum (601 MHz, TFA-d_1, 25 °C) of [*E*-**4b**-H]$^+$. The cross signals between protons and carbon atoms that show a $^3J_{H\text{-}C}$ coupling in the aromatic region and a $^{3/4}J_{H\text{-}C}$ coupling in the alkylic region confirm the structural assignment. Source data are provided as Source Data File.

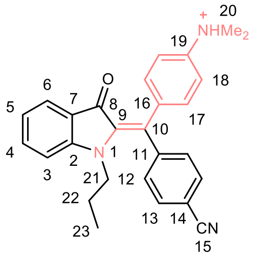

E-[**4b**-H]$^+$

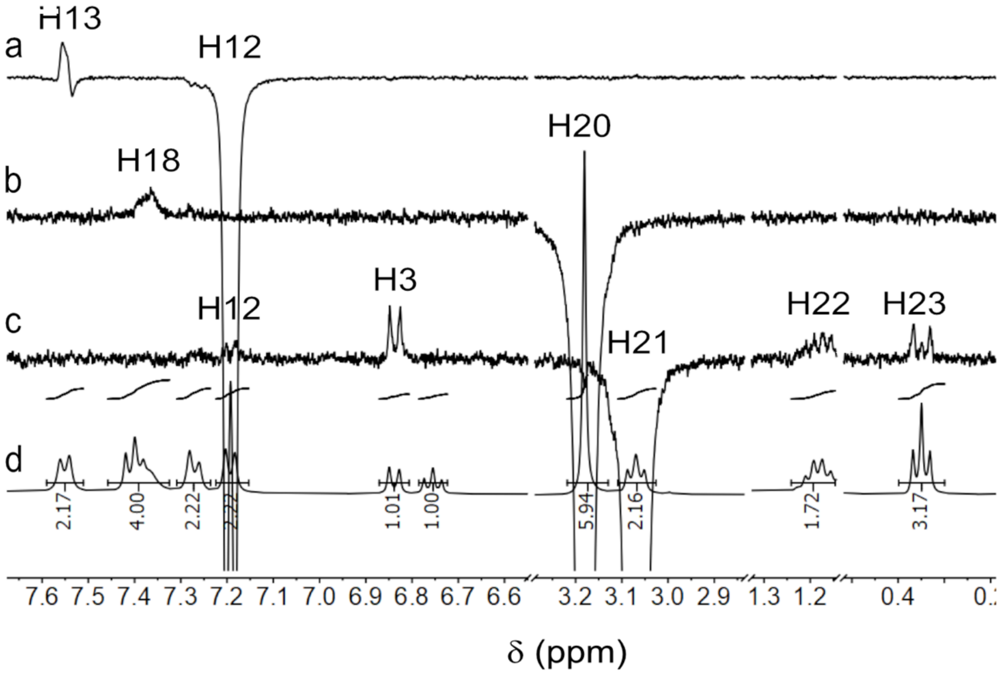

Supplementary Figure 143: 1D ^{1}H-^{1}H NOE NMR experiments with protonated *E*-[**4b**-H]$^+$ (400 MHz, TFA-*d*$_1$, 25 °C) to confirm the *E* configuration of *E*-[**4b**-H]$^+$ in TFA solution. **a)** Irradiating the protons at 7.19 ppm (H12 of *E*-[**4b**-H]$^+$) leads to magnetization transfer to the signal at 7.55 ppm (H13 of *E*-[**4b**-H]$^+$). **b)** Irradiating the protons at 3.18 ppm (H20 of *E*-**4b**-H$^+$) leads to magnetization transfer to the signal between 7.43-7.32 ppm (H18 of *E*-[**4b**-H]$^+$). **c)** Irradiating the protons at 3.07 ppm (H21 of *E*-[**4b**-H]$^+$) leads to magnetization transfer to the signals at 7.19 ppm (H12 of *E*-[**4b**-H]$^+$), 6.84 ppm (H3 of *E*-[**4b**-H]$^+$), 1.23 – 1.15 ppm (H22 of *E*-[**4b**-H]$^+$), and 0.35 ppm (H23 of *E*-[**4b**-H]$^+$). **d)** partial ^{1}H NMR spectrum of *E*-[**4b**-H]$^+$ (400 MHz, TFA-*d*$_1$, 25 °C). The aromatic protons H13 and H12 belong to the same spin system and are associated to the aryl substituent carrying the nitrile group. This aryl substituent is in proximity to the protons at H21 at the alkyl substituent, which confirms the *E* configuration. The magnetization transfer between the protons of H20 to the protons of H18 shows that they belong to the same spin system, but do not show a magnetization transfer to the protons at H21, which excludes a *Z* configuration. Source data are provided as Source Data File.

8.10 Acid induced isomerization of diaryl-HI 4c

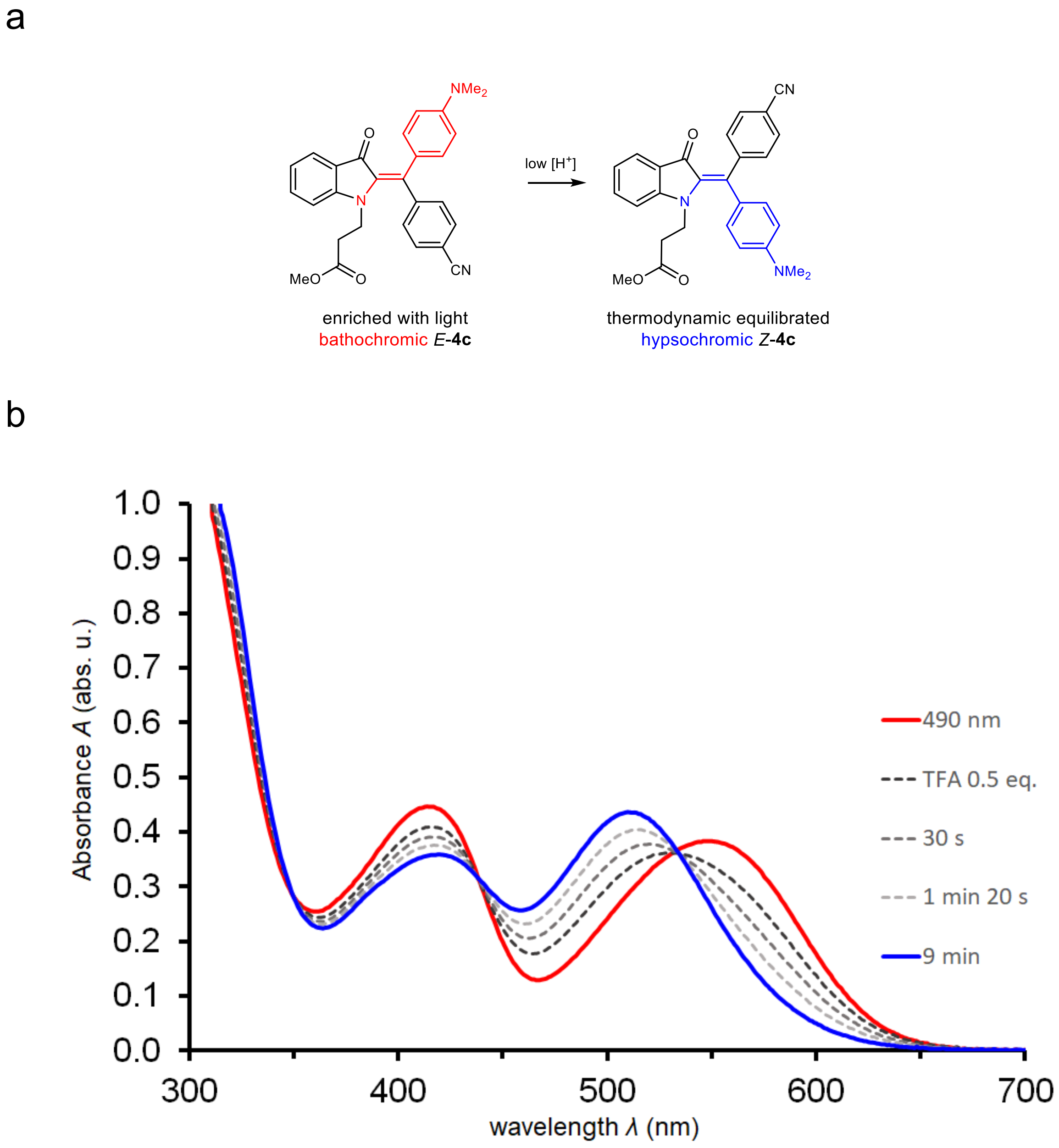

Supplementary Figure 144: a Schematic illustration of the acid induced isomerization from the bathochromic to the hypsochromic isomer of diaryl-HI **4c** (thermal equilibrium E = 26%, Z = 74%; hypsochromic pss: Z = 88%; bathochromic pss: E = 90%). **b** absorption spectra of diaryl-HI **4c** in toluene solution at 23 °C recorded after irradiation with 490 nm light (bathochromic *E*-**4c** enriched, red curve). Subsequent addition of 0.5 equiv. TFA leads to formation of the hypsochromic isomer *Z*-**4c** within 9 min. Acid induced thermal isomerization is accelerated compared to thermal isomerization of neutral **4c**. Source data are provided as Source Data File.

a

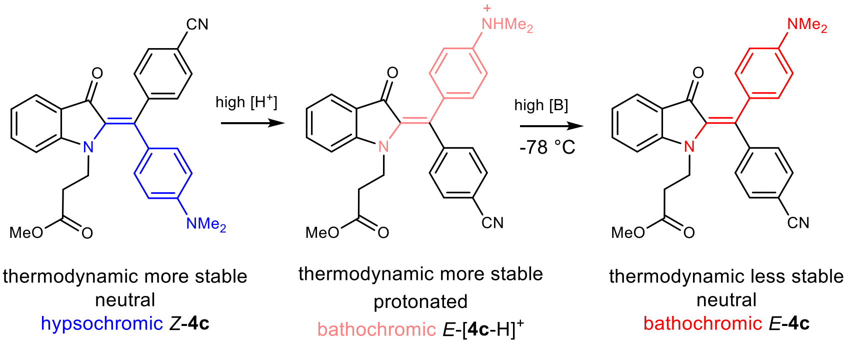

b

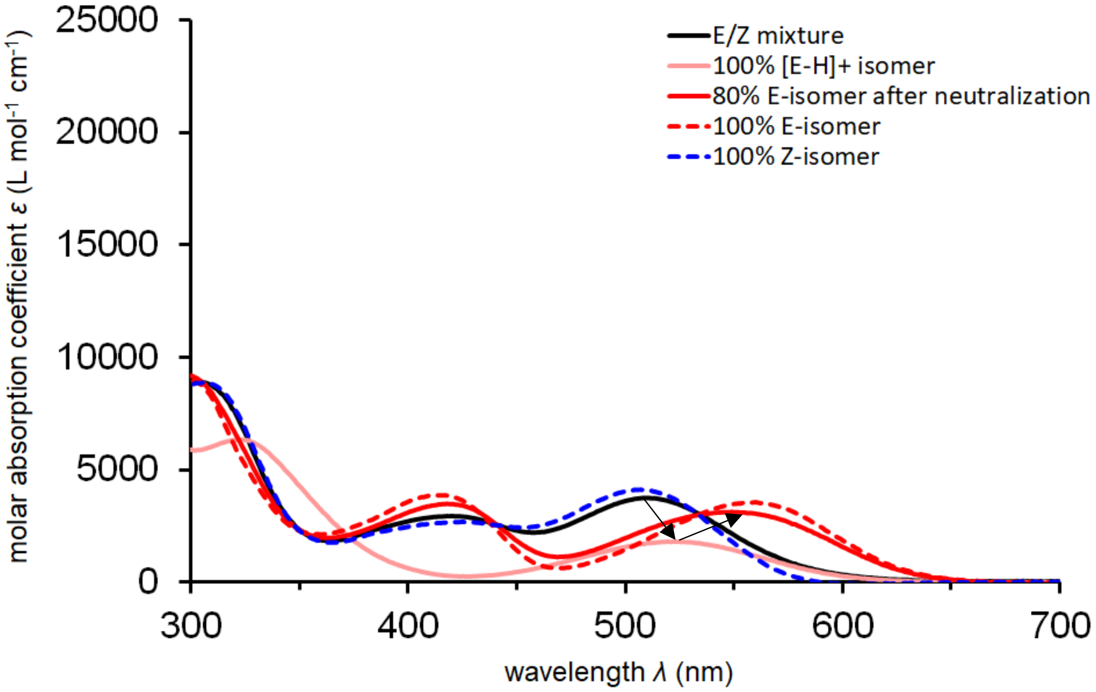

Supplementary Figure 145: Acid/base induced enrichment of the thermodynamically less stable *E*-**4c** isomer. Strong enrichment of *E*-**4c** is possible after addition of a large excess of TFA to a E/Z-**4c** mixture and subsequent neutralization with triethylamine. **a** Schematic illustration of acid and base induced isomerization of **4c**. **b** To a *E*/*Z*-**4c** mixture (black curve) in toluene solution, concentrated TFA (5,000 equiv.) was added at 23 °C. The solution containing newly formed *E*-[**4b**-H]$^+$ (salmon curve) was then cooled to –78 °C and triethylamine (5,000 equiv.) was added. The solution was allowed to reach 23 °C under fast shaking resulting in strongly enriched *E*-**4c** isomer (80%, red curve). Spectra were scaled to the known isosbestic points of the previously determined molar absorption coefficients of the pure *E* and *Z* isomers (100% *E*-isomer, 100% *Z*-isomer, dashed red and blue curves). Previous acid/base experiments show, that the isosbestic points do not change their position on the x-axis after acid/base treatment but change their position on the y-axis more or less pronounced correlated to dilution effects. The individual isomer content after neutralization with base was determined according to Supplementary Note 4 via (3) or (4) with the constraints that $x^E_{E+} + x^Z_{E+} = 1$ or $x^E_{Z+} + x^Z_{Z+} = 1$. Source data are provided as Source Data File.

Supplementary Note 9: Isomerization of diaryl-HIs within polymers

To inscribe the photochromic polymer the hypsochromic state was enriched by irradiation with 625 nm LEDs. Then an LED of suitable shorter wavelength was connected to a glass fiber and used for writing (Supplementary Figure 146 and Supplementary Figure 147). The resulting letters could be erased by red light, by sunlight, or by an acid treatment. For the latter TFA (6.5 mM, toluene) was dropped unto the polymer and quick erasing of the bathochromic inscription was observed.

Enriched isomers in the polymer are stable at 23 °C for many days in the dark. To test photostability under sunlight, the samples were stored under ambient conditions and exposed to indirect to direct sunlight until the respective pss composition under sunlight was restored (visual determination by color change).

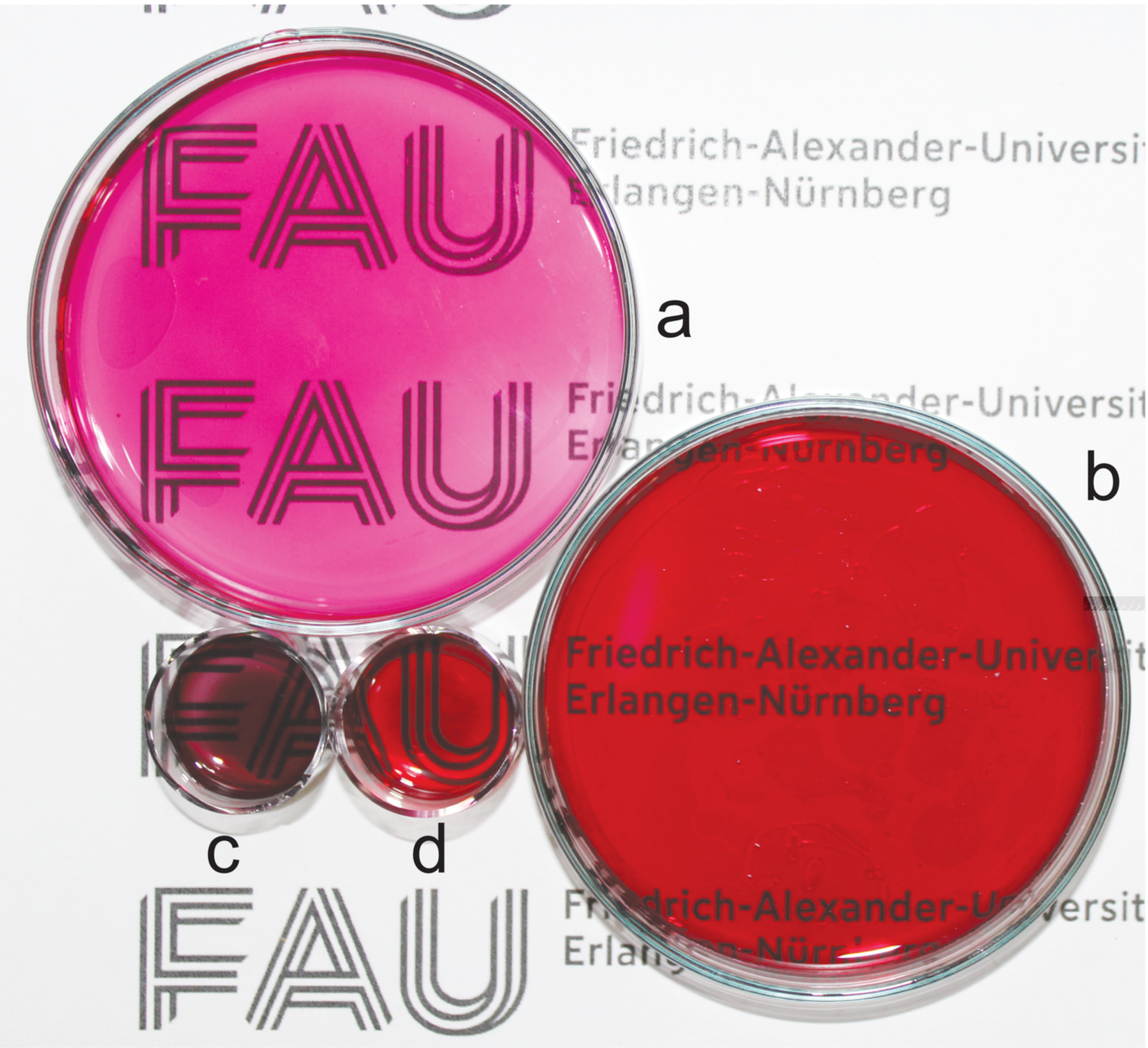

Supplementary Figure 146: Diaryl-HI polystyrene photochromic polymers are highly transparent and homogenous. Examples are shown for different isomers of the same molecule and a different range of concentrations. Petri dishes were placed unto a display showing the FAU logo. **a)** Bathochromic *Z*-**1b** (0.51 mM) embedded into a polystyrene matrix. **b)** Hypsochromic *E*-**1b** (2.4 mM) embedded into a polystyrene matrix. **c)** Bathochromic *E*-**4b** (1.6 mM) embedded into a polystyrene matrix. **d)** Hypsochromic *Z*-**4b** (1.6 mM) embedded into a polystyrene matrix. Source data are provided as Source Data File.

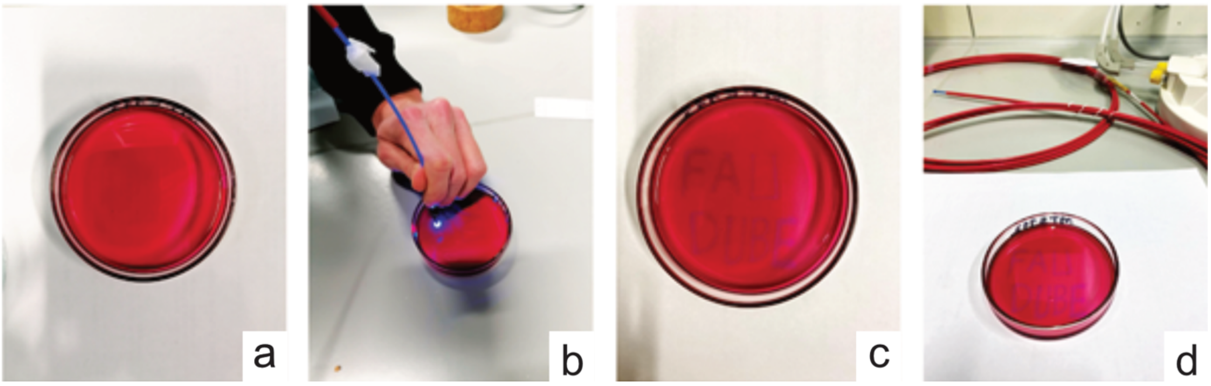

Supplementary Figure 147: Diaryl-HI **1b** (0.51 mM) was embedded into a polystyrene matrix to write the text "FAU DUBE" into the polymer with blue light. **a** Diaryl-HI **1b** was embedded into the polystyrene matrix and globally irradiated with 625 nm light. **b** The text "FAU DUBE" was written into the polymer with a 470 nm HP-LED connected to a glass fiber. **c** and **d** Depiction of the inscribed text "FAU DUBE". Source data are provided as Source Data File.

Supplementary Note 10: Crystal Structural Data

Supplementary Table 12: Crystal structural data of diaryl-HI *Z*-**1a** and diaryl-HI *Z*-**3b**.

compound	*Z*-**1a** (CCDC 2191572)	*Z*-**3b** (CCDC 2191573)
Formula	$C_{26}H_{26}N_2O$	$C_{28}H_{30}N_2O_3$
$D_{calc.}$ (g × cm^{-3})	1.238	1.251
μ (mm^{-1})	0.586	0.646
Formula Weight	382.49	442.54
Colour	clear light red	clear dark red
Shape	plate-shaped	plate-shaped
Size (mm^3)	0.16 × 0.11 × 0.04	0.16 × 0.10 × 0.03
T (K)	153.05(10)	153.00(14)
Crystal System	monoclinic	monoclinic
Space Group	$P2_1/c$	$P2_1/c$
a (Å)	13.8068(4)	17.3406(5)
b (Å)	12.4035(3)	10.2180(2)
c (Å)	12.2369(3)	13.8422(3)
a (°)	90	90
b (°)	101.701(3)	106.615(3)
g (°)	90	90
V (Å^3)	2052.05(9)	2350.24(10)
Z	4	4
Z'	1	1
Wavelength (Å)	1.54184	1.54184
Radiation type	Cu K_a	Cu K_a
θ_{min} (°)	3.269	2.659
θ_{max} (°)	71.504	71.320
Measured Refl's.	7555	9099
Indep't Refl's	3865	4454
Refl's I≥2 s(I)	3033	3652
R_{int}	0.0392	0.0213
Parameters	267	304
Restraints	0	0
Largest Peak	0.363	0.247
Deepest Hole	-0.333	-0.246
GooF	1.045	1.050
wR_2 (all data)	0.1537	0.1177
wR_2	0.1392	0.1082
R_1 (all data)	0.0659	0.0536
R_1	0.0528	0.0421

Supplementary References

[1] U. Megerle, R. Lechner, B. König, E. Riedle, *Photochemical & photobiological sciences : Official journal of the European Photochemistry Association and the European Society for Photobiology* 2010, *9*, 1400.
